# Crystallography in school

**DOI:** 10.1107/S1600576725007459

**Published:** 2025-09-12

**Authors:** Erhard Irmer

**Affiliations:** a XLAB – Göttingen Experimental Laboratory for Young People Georg-August-Universität Göttingen Justus-von-Liebig-Weg 8 37077Göttingen Germany; Wilfrid Laurier University, Waterloo, Ontario, Canada

**Keywords:** crystallography in school, database structures, structure determination, teaching

## Abstract

The study of crystallography and the introduction to X-ray structure analysis are generally regarded as tasks for universities and, even then, usually only in graduate studies. However, analysis of crystal structures in science classes at high schools offers a wide range of opportunities for illustrating and improving understanding of fundamental structural chemistry concepts. This article attempts to share some experiences with crystallographers who want to work with high school students on crystallographic topics. After presenting some preconditions for introducing students to crystallographic topics, such as the curricular situation, two levels of didactical reduction are suggested. Examples of the use of database structures from the Protein Data Bank and the Teaching Subset of the Cambridge Structural Database in chemistry lessons are presented. In the Göttingen experimental laboratory for young people, XLAB, high school students can carry out the essential steps of structure determination, structure solution and refinement of X-ray diffraction data themselves, using aspirin or citric acid as examples. Finally, a network scheme for promoting crystallographic topics in the classroom is proposed.

## Abstract 2

Preconditions and applications for teaching crystallography in high school chemistry classes are presented.

## 1. Introduction

The study of crystallography is usually associated with university and therefore more with graduate studies. There are a number of activities to familiarize the broader public with crystallographic topics [see *e.g.* Murray *et al.* (2024 ▸) and Schimpf *et al.* (2023 ▸); activities at the IUCr 2023 Congress in Melbourne, *e.g.* Zheng *et al.* (2025 ▸); or examples from the 2024 ACA Transactions Symposium, *e.g.* Bou-Nader *et al.* (2025 ▸) and Kaduk (2025 ▸)]. Especially for undergraduate students there are a large number of activities presented in the literature, for example by Zheng & Campbell (2018 ▸) and Caro *et al.* (2023 ▸). A complete overview of the literature on this subject would go beyond the scope of this publication. The General Interest Group ‘Education in crystallography’ (GIG-03) of the European Crystallographic Association (ECA) launched an initiative in 2023 to make crystallographic topics accessible to children and young people of school age. In August 2024 a workshop, ‘Crystallography in school’, was held as a satellite meeting to the European Crystallographic Meeting ECM34 in Padova (Italy), at which best-practice examples were presented in a series of lectures; the abstracts give an idea of the bandwidth of topics (Bacchi, 2024 ▸; Galli *et al.*, 2024 ▸; Gimondi *et al.*, 2024 ▸; Graw, 2024 ▸; Gupta, 2024 ▸; Ienco *et al.*, 2024 ▸; Irmer, 2024 ▸).

This article primarily addresses crystallographers and will focus on the application of crystallographic aspects in the context of school teaching, concentrating on examples from chemistry lessons for high school students that the author has carried out over more than two decades. Therefore, the applications do not follow a carefully developed concept but rather reflect how new aspects of crystallography were gradually discovered and explored in the classroom. The basic idea was to find low-threshold approaches to crystallographic topics that would prove useful in working out classical concepts of structural chemistry.

The applications of database structures originate from chemistry lessons in upper school courses at the Otto-Hahn-Gymnasium in Göttingen. The applications for structure solution and refinement were carried out as part of courses for high school students at the XLAB student laboratory at the Georg-August-Universität Göttingen. The XLAB experimental laboratory for young people (XLAB, 2025 ▸) is one of the largest student laboratories in Europe with around 10000 participants per year in the fields of physics, chemistry, biology and computer science/mathematics and offers courses for school classes as well as school holiday camps and teacher training events.

## 2. Preconditions for teaching crystallography at school: a spotlight

**Figure 1:**
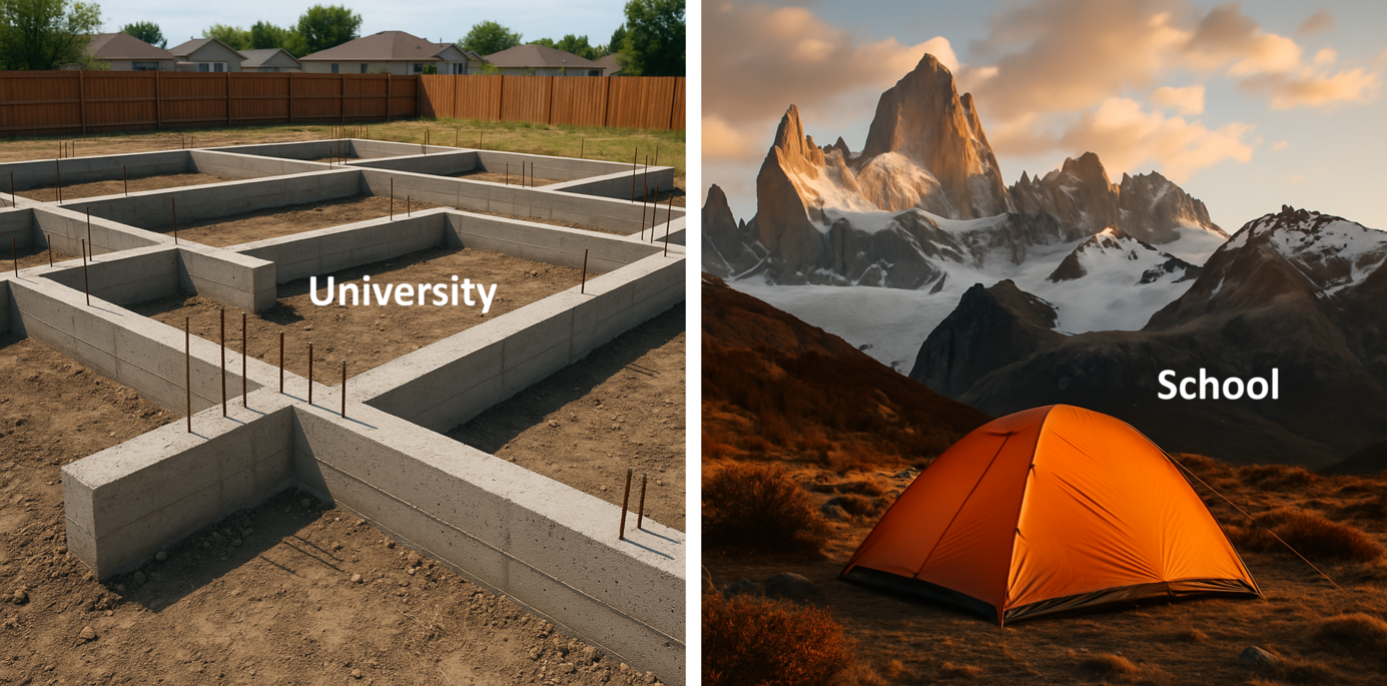
Analogy for teaching crystallography at university and at school [*Concrete building foundations on a construction site* and *Tent in front of a mountain landscape at sunset* (AI-generated images). ChatGPT: https://chat.openai.com/].

At this point, I would like to concentrate on two key aspects that, in my experience, are particularly important for crystallographers when working with students. Compared with the teaching of crystallography at university, a completely different perspective and approach is required at school. At university, solid foundations must be laid in the undergraduate studies on which a stable building (knowledge in crystallography) can later be erected. At school, students have to be introduced to a new, fascinating field of science. To stay with the analogy, a lightweight tent that can be erected quickly is sufficient to take a ‘trip’ into crystallography (Fig. 1 ▸).

**Figure 2:**
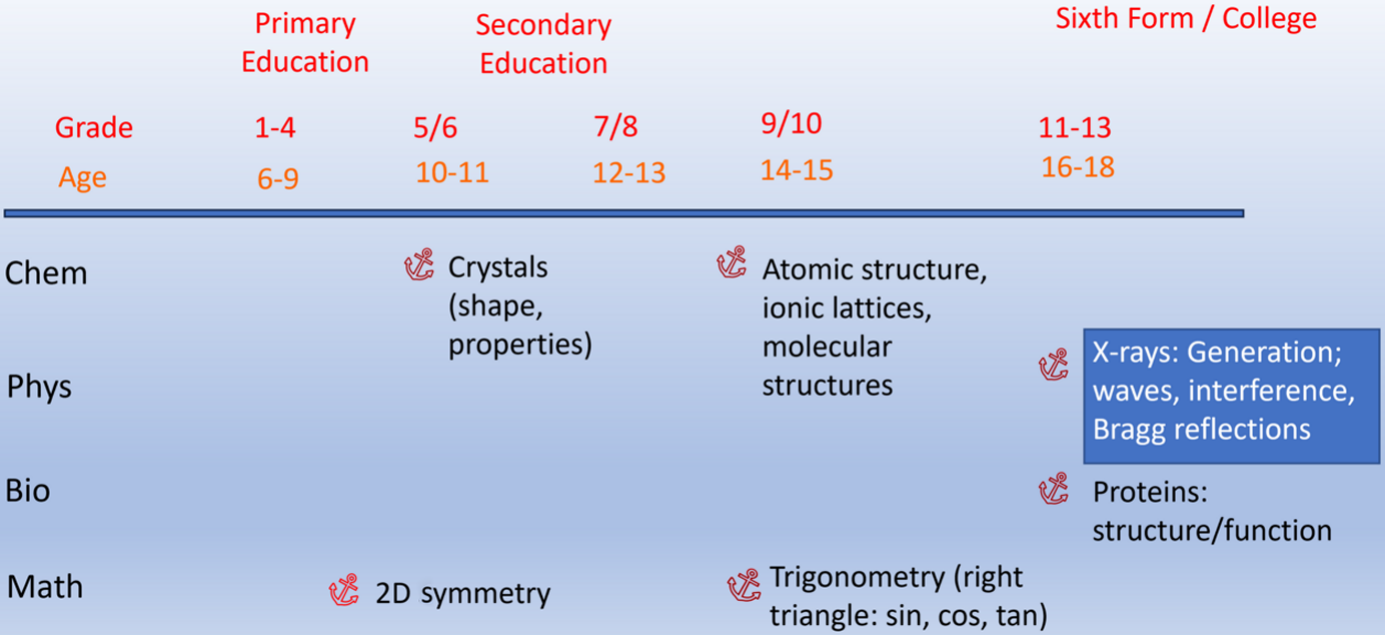
Anchor points for crystallographic topics in the STEM curricula of Lower Saxony, Germany (NLQ, 2025 ▸).

Crystallographers are often unsure about which aspects of school knowledge they can link to with their explanations or activities. In most German curricula for chemistry lessons crystallographic topics are not even mentioned explicitly. However, there are a whole series of anchor points in the STEM (science, technology, engineering and mathematics) curricula where crystallographic topics can be linked. This can be seen in the overview in Fig. 2 ▸ using the example of the curricula in chemistry, physics, biology and mathematics in Lower Saxony, Germany (NLQ, 2025 ▸). Even in primary school mathematics lessons, pupils can be introduced to plane symmetry in a playful way. In chemistry lessons in grades 5 and 6, pupils learn about crystals and how to characterize them using salt-like substances. However, understanding of the structure of ionic and molecular crystals is only possible when the structure of atoms and chemical bonding (ionic and covalent) are dealt with in grades 9 and 10. The mathematical knowledge of trigonometry required to understand Bragg reflection is acquired in year 9/10. For biology lessons, crystallographic topics can be dealt with as part of the topic ‘structure and function of proteins’. It is particularly important for the treatment of structure determination methods that knowledge of diffraction phenomena, *e.g.* Bragg reflection or interference, cannot be assumed, as these topics are only dealt with in physics lessons at the college level (year 11–13) and many pupils no longer have to take physics in the sixth form.

## 3. Didactical reduction of the theory

The physically and mathematically demanding theoretical fundamentals of crystallography are often regarded as a particularly serious obstacle to getting started with X-ray structure analysis. When working with school students, the division into two levels of didactical reduction has proved useful. A very basic level is sufficient as an introduction to the method and as a basis for working with ‘finished’ crystal structures, while some additional knowledge (advanced level) is required to understand the structure solution and refinement process.

**Figure 3:**
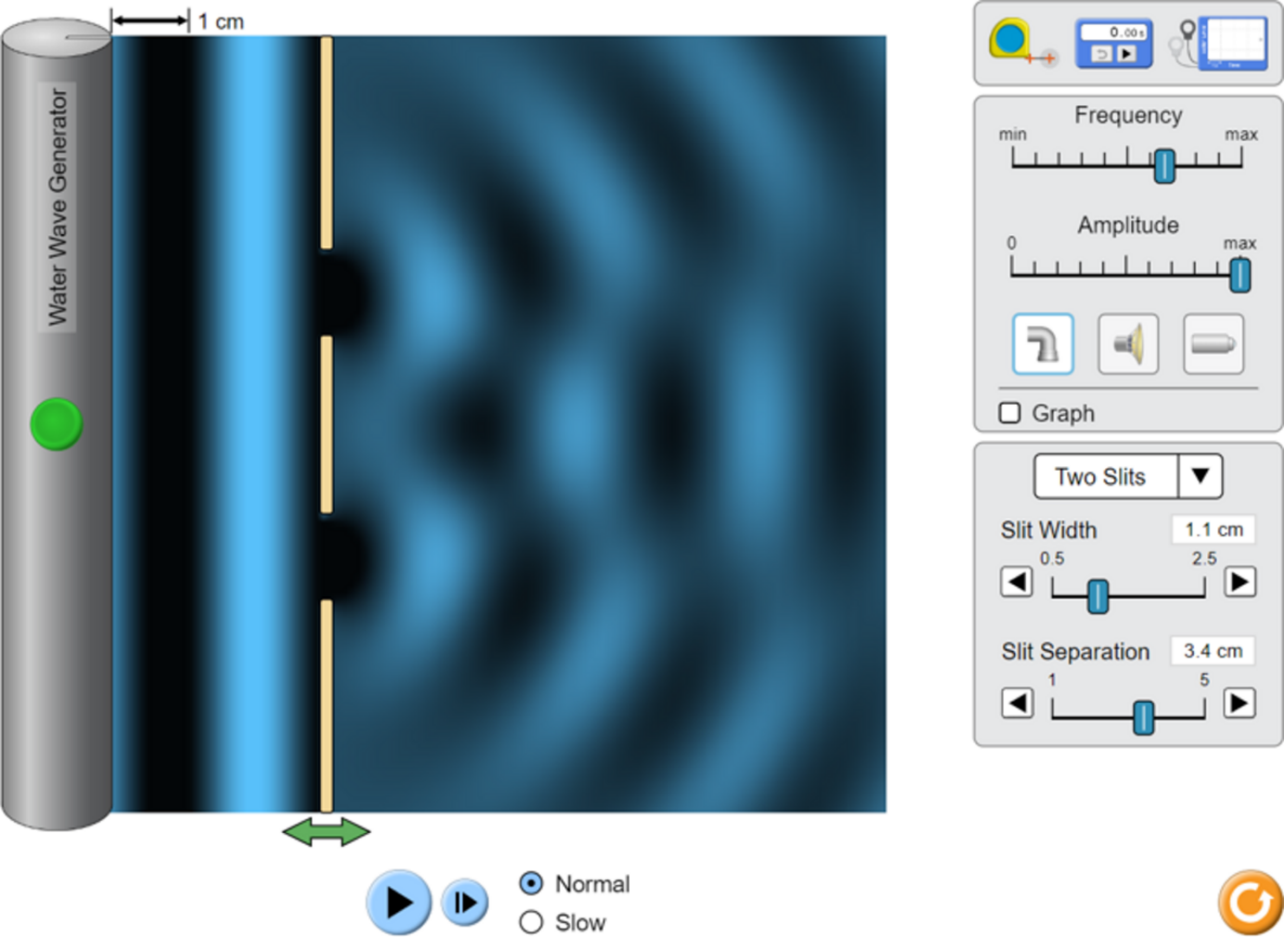
The most basic level of didactical reduction of the theory of X-ray diffraction: interference of water waves at a double slit. Simulation by PhET Interactive Simulations, University of Colorado Boulder, licenced under CC-BY-4.0 (https://phet.colorado.edu).

**Figure 4:**
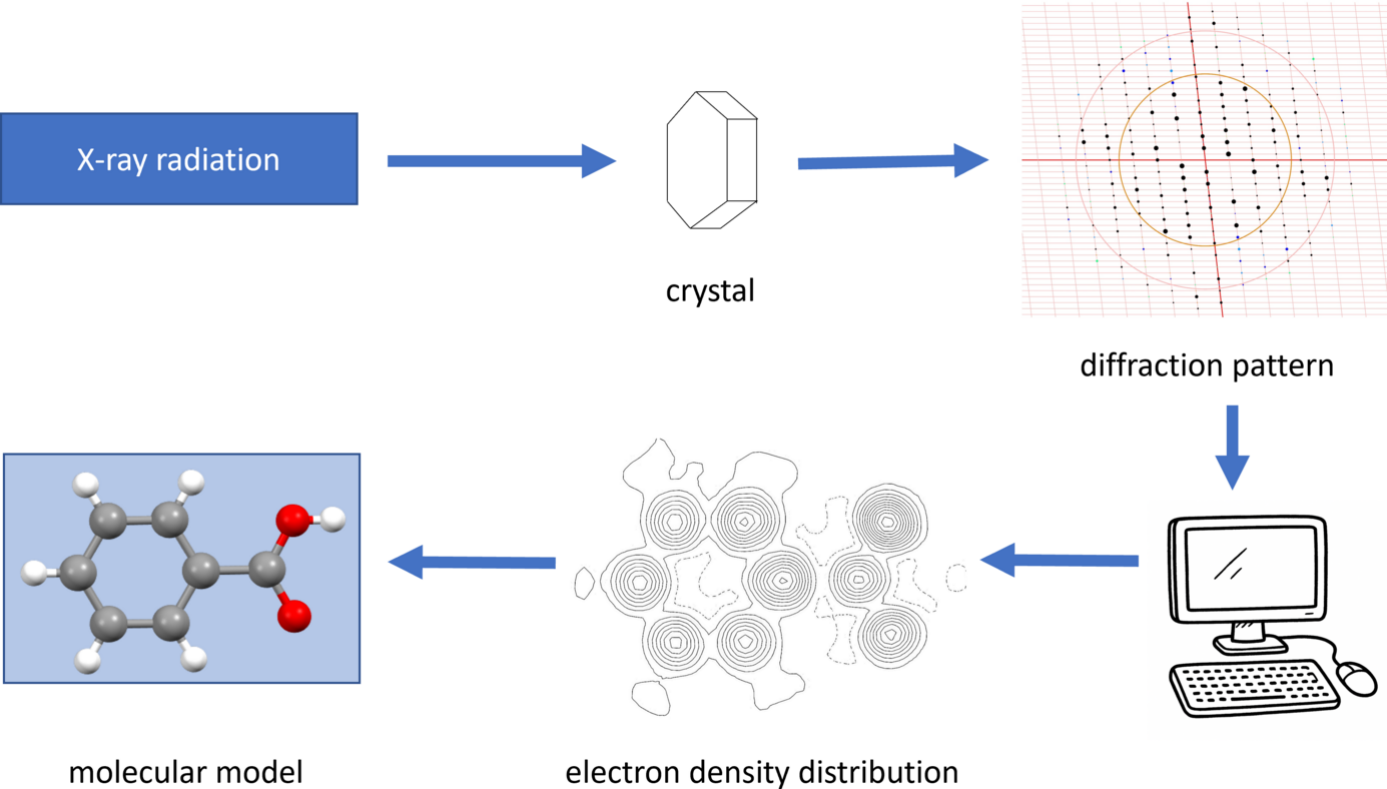
The most basic level of didactical reduction of the theory of X-ray diffraction: scheme of the X-ray structure determination process.

For reasons of vividness, it is recommended to start with the diffraction experiment. It is ideal if this can be combined with a visit to an X-ray structure analysis department, where the students can get to know the equipment directly. Since, as shown above (Section 2), the students may not yet have any knowledge of diffraction, this phenomenon must be introduced first. It has proved helpful to use the diffraction of water waves at a double slit as an explanation. It can be demonstrated experimentally or with the help of simulations (Fig. 3 ▸; PhET, 2025*a* ▸) that the diffraction pattern of the interfering waves can be used to deduce the arrangement at which the diffraction takes place, even if this arrangement is not visible. It also becomes clear that a regular diffraction pattern can only be obtained if the slits are arranged regularly. Alternatively or additionally, diffraction phenomena of laser pointer light on optical grids can be used (Chayanun *et al.*, 2022 ▸). A disadvantage is that, in this case, the wave character of the light must be postulated, while, in fact, it can only be deduced by the diffraction phenomena. In the X-ray diffraction experiment, the X-ray diffraction pattern produced by the crystal can be used to draw conclusions about the arrangement of the electrons of the atoms arranged regularly in the crystal structure. From the electron density distribution atomic positions can be obtained and represented in a classical molecular model (Fig. 4 ▸).

If the students are expected to solve and refine crystal structures themselves, as described below (Section 4.2), it must be explained why the determination of the electron density is not as trivial as it may appear in the basic description. The illustrative representation of Bragg reflection and the phase problem is standard knowledge in any university introduction to crystallography (*e.g.* Massa, 2016 ▸) and does not need to be explained further here. The supporting information contains *PowerPoint* slides that illustrate this topic. The details of solving the structure using direct methods cannot be discussed with students; as a simplified explanation, it is explained that starting sets of randomly selected phase sets are tried out until a chemically meaningful result is obtained. Once a reasonably sensible structural model is obtained, it is possible to compare the calculated intensities of the diffraction maxima with the measured intensities and to improve the model so that the agreement with the experiment becomes better and better. The procedure for a linear regression is suitable for illustrating this least-squares-refinement strategy [see supporting information and PhET (2025 ▸*b*)].

By starting with the diffraction experiment, the topic of symmetry in the crystal structure can be avoided at the beginning. Experience has shown that the understanding of 3D symmetry often causes considerable difficulties for learners. For this reason, it is also a good idea to first look at organic compounds, in which the molecules often have no or only simple symmetry elements. Once the students have gained basic experience with the method, they can ‘discover’ symmetry elements in the ‘finished’ crystal structures and move from a simple inversion centre, for example in the structure of octane, to more complex symmetry relationships between or within the molecules.

The vividness of the results also argues in favour of starting X-ray structure analysis with single crystals. Although diffraction experiments on powder are easier to perform and can be carried out with equipment suitable for schools, the clarity of the results is much lower compared with single-crystal structure analysis, even if there are nice applications in substance characterization [*e.g.* Kaduk (2025 ▸) or Iqbal *et al.* (2024 ▸)].

## 4. Applications of crystallography in school

### 4.1. Use of database crystal structures

#### 4.1.1. Protein structures from the PDB

**Figure 5:**
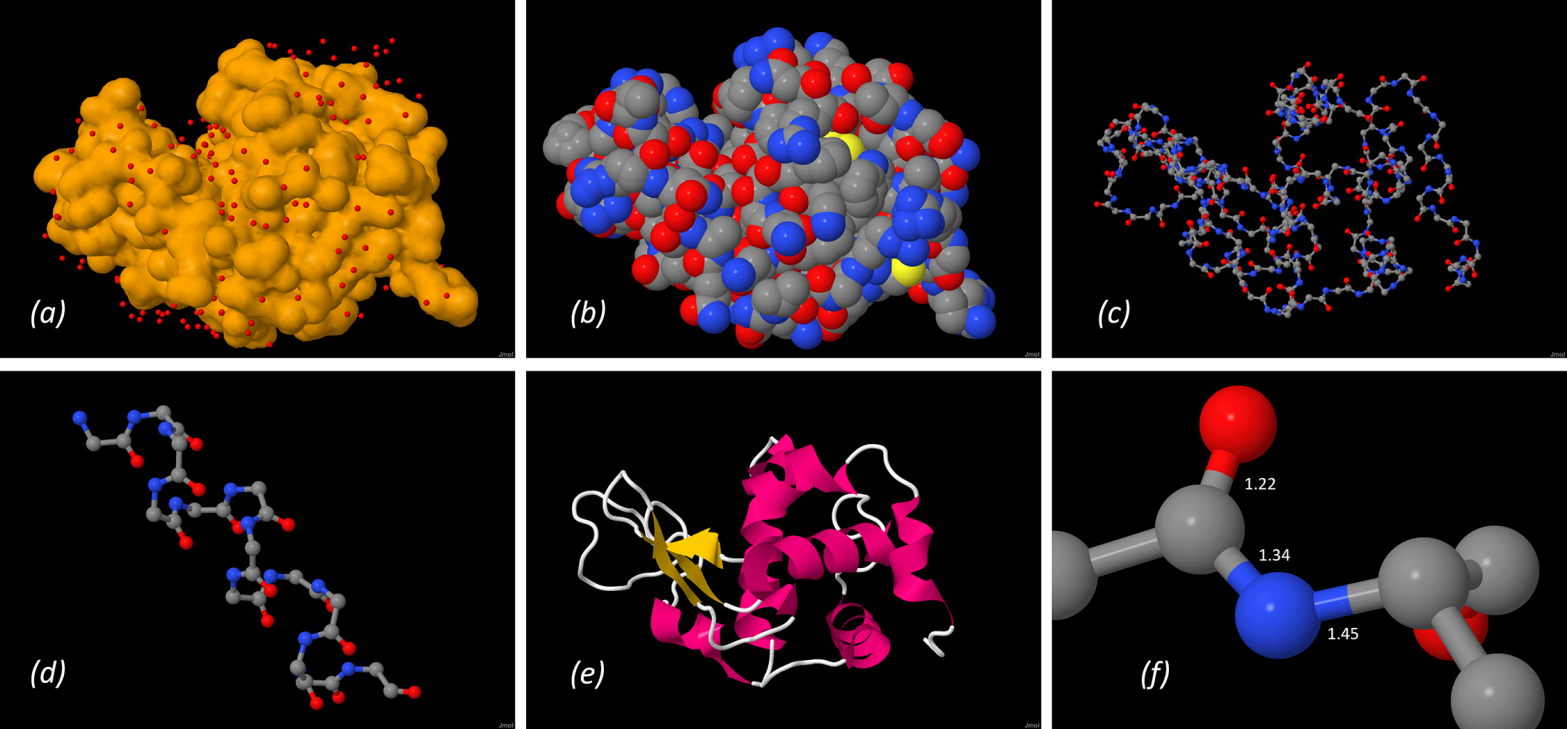
From protein to amino acid – structure of lysozyme (*Jmol* representations from PDB entry 1iee): (*a*) surface of the protein with water molecules, (*b*) surface with atom types, (*c*) backbone of the protein, (*d*) α-helix secondary structure element, (*e*) cartoon representation and (*f*) peptide bond with bond lengths (Å).

The RCSB Protein Data Bank (PDB) (Berman, 2000 ▸) was one of the first crystallographic databases whose contents were freely accessible. The structures were also made usable from a didactical point of view at an early stage, for example through prepared accompanying material on important structures. A teaching course was successfully tried out by the author, in which school students were sent on a ‘journey of discovery’ through the structure of the protein lysozyme with the help of a worksheet (Irmer, 2011 ▸). Under the motto ‘From protein to amino acid’, the structure of a high-resolution protein structure (PDB entry 1iee; Sauter *et al.*, 2001 ▸) was investigated with the help of the molecule viewer *Jmol* (Hanson, 2010 ▸), starting with the surface of the protein and going into more and more detail until the peptide bond was examined (Fig. 5 ▸). This allows the tertiary structure to be determined, the active centre to be discovered, and statements to be made about the polarity via the water molecules bound to the surface or atom types on the surface. Students can explore the chain structure of the protein backbone, discover secondary structure elements such as α-helices and β-sheets, identify amino acids on the basis of their side chains, and learn about the steric characteristics of the peptide bond. During the Covid pandemic, this topic was also used in the XLAB as an online student course.

Other topics can be covered well in chemistry lessons with the help of PDB database structures, *e.g.* the structure and function of haemoglobin (RCSB, 2025*a* ▸). The sections from the structure in the ‘Molecule of the Month’ area of the PDB website are helpful here. Further materials that can be used in school can be found on the PDB website in the ‘PDB-101’ section (RCSB, 2025 ▸*b*). An example of a combination of protein structure determination with the use of the PDB is given by Fox *et al.* (2023 ▸).

#### 4.1.2. Crystal structures from the CSD Teaching Subset

**Figure 6:**
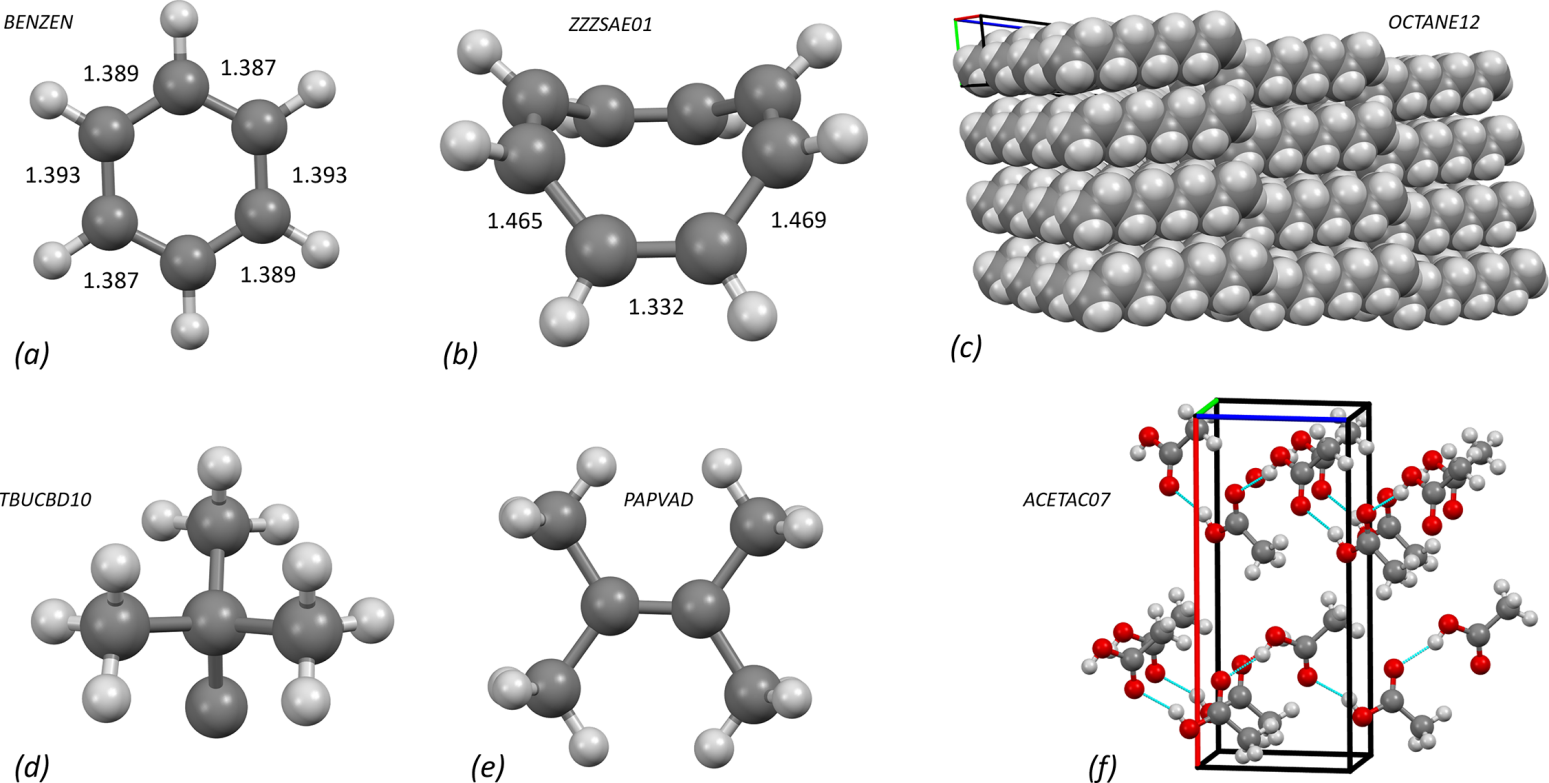
Applications of the CSD Teaching Subset, generated using *Mercury* (Macrae *et al.*, 2020 ▸): structures of (*a*) benzene and (*b*) cyclo­octatetra­ene with C—C distances (Å); (*c*) packing of *n*-octane, (*d*) *sp*^3^ and (*e*) *sp*^2^ carbon geometry; (*f*) acetic acid packing with hydrogen bonds.

A series of articles in *Journal of Chemical Education* in 2010 (Battle *et al.*, 2010*a* ▸; Battle *et al.*, 2010*b* ▸) and 2011 (Battle *et al.*, 2011*a* ▸; Battle *et al.*, 2011*b* ▸) familiarized teachers with the Teaching Subset of the Cambridge Crystallographic Structural Database (CSD) (Battle *et al.*, 2010*c* ▸). I have had good experiences using CSD structures for a number of topics in school lessons (Fig. 6 ▸). For example, the structure of benzene (refcode BENZEN; Bacon *et al.*, 1964 ▸) and its aromatic character can be demonstrated very well by comparing the bond lengths and angles with a non-aromatic molecule such as cyclo­octatetra­ene (refcode ZZZSAE01; Claus & Krüger, 1988 ▸). The comparison of molecular geometries of simple carbon compounds [*e.g.* refcodes TBUCBD10 (Irngartinger & Nixdorf, 1983 ▸), PAPVAD (Boese *et al.*, 1992 ▸) and JUFDUJ (Mootz & Deeg, 1992 ▸)] with those predicted by the VSEPR model can be extended for more complex geometries by considering organometallic compounds. It is important that the geometries are not verified on hydrogen atoms, as these are usually refined with constraints or restraints in idealized geometries. Crystal structures can also be used to investigate the interactions between molecules, since the most energetically favourable conformations in the solid state are ‘frozen’ in the crystal structure (*e.g.n*-octane, refcode OCTANE12; Boese *et al.*, 1999*a* ▸). The representation of crystal packing makes it very clear why these molecules can form strong London forces. Crystal structures can also be helpful for a deeper understanding of hydrogen bonds, as the strength of hydrogen bonds can be easily estimated from the distances and angles. In addition, misunderstandings – some of which are also represented in textbooks – can be eliminated: The comparatively high melting point of acetic acid cannot be explained by the presence of dimers. A view of the packing in the structure of acetic acid (refcode ACETAC07; Boese *et al.*, 1999*b* ▸) shows that the molecules in the crystal are linked in chains by hydrogen bridges. Dimers actually only occur in the gas phase (Socha & Dračínský, 2020 ▸).

A large number of teaching materials have also been published on the CSD website, which can also be used in schools (CCDC, 2025*a* ▸; see also Abourahma, 2024 ▸). A licence is required to use the complete database with all search and evaluation functions, but access to the Teaching Subset is free of charge (CCDC, 2025 ▸*b*). In the free version of the *Mercury* molecule viewer (Macrae *et al.*, 2020 ▸), the Teaching Subset is also automatically installed. Otherwise, a search filter on the web interface *WebCSD* (Thomas *et al.*, 2010 ▸) or a categorized compilation of the structures in an *Excel* file enables access to the subset. A compilation of structures particularly suitable for teaching chemistry in schools, categorized by substance class and enabling direct access to the structures via either *WebCSD* or *Mercury*, is available as supporting information. However, non-licenced users of the CSD can also download the CIF of a structure not contained in the Teaching Subset via the web interface, for example by searching for a compound name. Further, purely inorganic, freely accessible structural data can be found in the Crystallography Open Database (Gražulis *et al.*, 2009 ▸).

### 4.2. Crystal structure solution and refinement by students

For about two years now, encouraged by the work of students with database structures and by examples from the literature on working with high school students and undergraduate students (Bazley *et al.*, 2018 ▸; Abrahams *et al.*, 2023 ▸; Beauparlant *et al.*, 2023 ▸), we have gained initial experience at XLAB with students solving and refining structures themselves using diffraction data provided. This is embedded in existing XLAB student courses, for example on the everyday topics of producing aspirin, isolating caffeine from tea or elucidating the structure of citric acid (XLAB, 2025 ▸). In this way, the method of X-ray structure analysis is always com­bined with practical work in a classical chemistry laboratory.

#### 4.2.1. Example: XLAB course ’Aspirin – synthesis and analysis’

As a concrete example of a course in which the students solve and refine a structure themselves, the one-day XLAB course ‘Aspirin – synthesis and analysis’ (XLAB, 2025 ▸) is presented. After the welcome, a safety briefing and a short introductory lecture, the students synthesize aspirin in the laboratory by esterification of salicylic acid with acetic anhydride and purify the crude product by recrystallization in an ethanol–water mixture. The still-wet fine product is then dried in a drying oven over lunchtime. In the traditional aspirin course, in the afternoon the students learn about the detection reaction for salicylic acid with iron(III) chloride solution and use this test quantitatively to determine any salicylic acid still present by UV–Vis spectroscopy.

**Figure 7:**
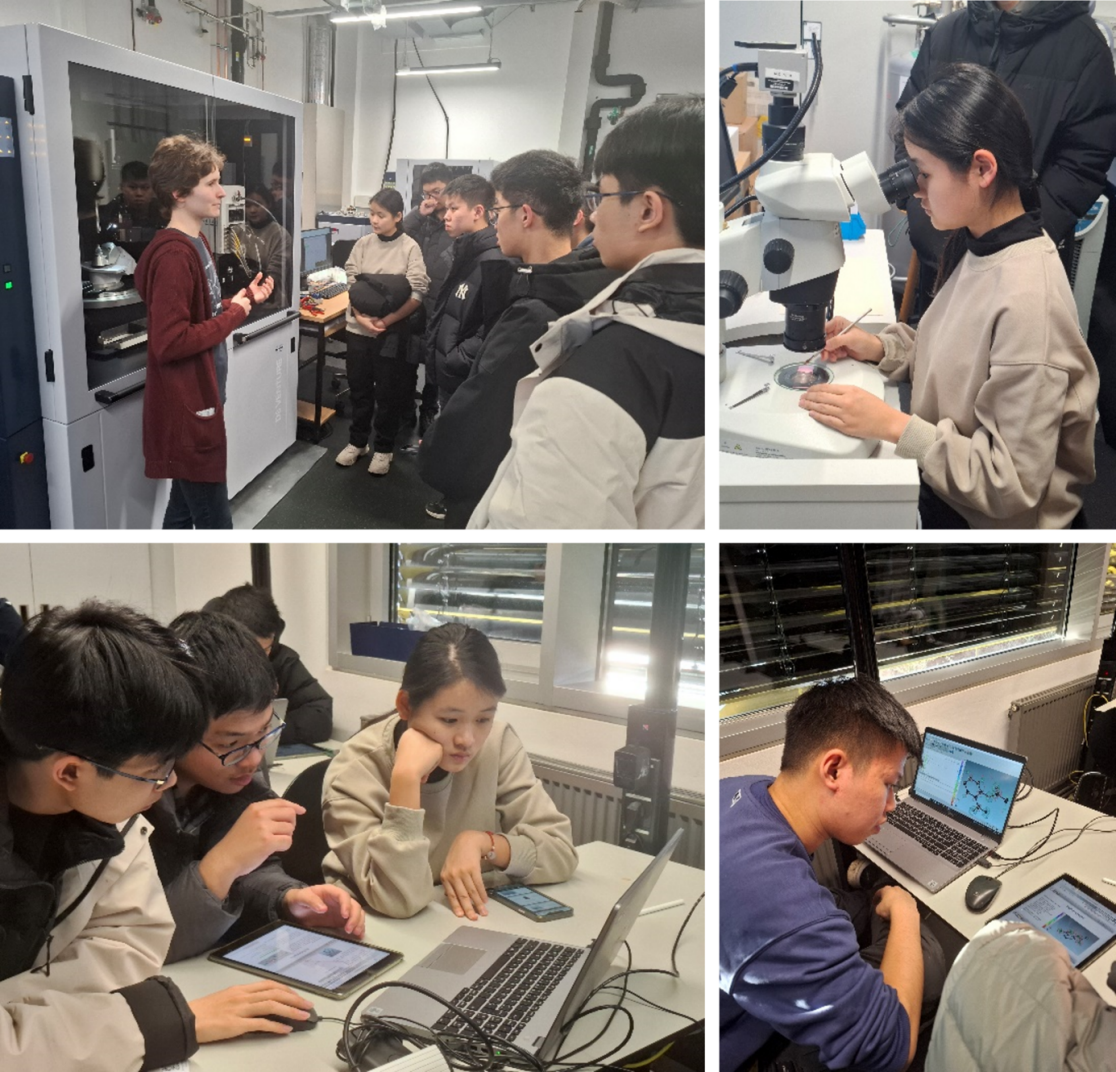
International students analysing aspirin crystals in an XLAB course: visit to the X-ray laboratory (top), solving and refining the structure (bottom). Photographs: E. Irmer, with permission of the participants.

In the alternative aspirin course, in the afternoon X-ray structure analysis is introduced as an analytical method for structure determination. The students receive an introduction to the method of X-ray structure analysis at the ‘basic level’ in the XLAB (see Section 3 and the supporting information: *PowerPoint* slides for the basic level of didactical reduction). Then we go to the Department of X-ray Structure Analysis in the Faculty of Chemistry, where a PhD student or technical employee shows the students the criteria for selecting a suitable crystal under the polarization microscope using the sample of the recrystallized substance that they have brought with them and – if the measuring situation allows – also starts a measurement. The setup of a diffractometer is explained and the generation of X-rays is discussed [Fig. 7 ▸(*a*)]. It is particularly motivating when the students can search for suitable crystals under the microscope themselves and try to place them on the pin of a goniometer head [Fig. 7 ▸(*b*)]. One problem here is that larger groups may have to be split up because there is not much space available in the diffractometer laboratory. Back in the XLAB, after a 10 min walk, the students are given a more in-depth introduction to structure determination (Section 3 and supporting information:*PowerPoint* slides for the advanced level of didactical reduction), where the phase problem, structure solution and the least-squares method are discussed.

A step-by-step tutorial then guides the students through the structure determination of acetyl­salicylic acid (Fig. 7 ▸). The students work on a dataset that was measured in Professor D. Stalke’s research group on a crystal of aspirin synthesized by students. In addition to the *hkl* file with the reflection intensities, the students receive an ins file that already contains the cell parameters and symmetry information. In this way, we avoid the very complex topic of space-group assignment, as explained above.

The tutorial is made available to the students on a tablet computer, while the structure determination is carried out on a laptop. The students can work through this tutorial independently at their own pace in groups of two or three. In principle, the step-by-step tutorial is self-explanatory, but the supervisor is available to answer questions and provide assistance. The illustrated step-by-step instructions (see the supporting information) are intentionally designed so that the students do not just have to follow instructions, but instead questions or impulses are inserted to encourage them to participate and try things out for themselves. For example, during the refinement students are encouraged to make a wrong atom assignment (refine oxygen atoms as carbon) in order to experience the effects on the displacement parameters. They notice that the displacement parameters are significantly smaller for the ‘wrong’ atoms and that there is positive residual density around these atoms. This is a good opportunity to discuss the meaning of the displacement parameters with the students. A discussion may also start when the students reach a point where they do not get further by themselves. This may happen when they had assigned an atom type to a *Q* peak near an existing atom instead of changing the atom type or when they try to refine a wrongly assigned atom anisotropically. At the end, the students analyse the bonding properties in the structural model and the packing of the molecules in the crystal structure.

Enabling students to carry out structure solution and refinement themselves is only possible because user-friendly graphical user interfaces (GUIs) are available. We started with *OLEX2* (Dolomanov *et al.*, 2009 ▸) because this GUI is freely available and has a ‘modern’ interface structure that students get used to quickly. The installation includes all components needed (charge-flipping algorithm for structure solution and *olex2.refine*) and is quite straightforward even for non-specialists. Then Christian Hübschle provided us with a version of *ShelXle* (Hübschle *et al.*, 2011 ▸) specially adapted to our needs, where all components [*SHELXS* (Sheldrick, 2008 ▸) and *SHELXL* (Sheldrick, 2015*b* ▸)] can be installed in one step too. Supervisors and students particularly like the clear interface of this *ShelXle XLAB Edition*, which is reduced to the essentials, with the buttons arranged from left to right in accordance with the structure determination process. The colour-coded intensity of the *Q* peaks in *ShelXle* has proved very helpful when working with students. But both GUIs are comparable in terms of usability. We provide customized step-by-step tutorials for both GUIs (see the supporting information) so the supervisor can decide which version they are familiar with or which one they want to use.

For structure solution in the *ShelXle* XLAB Edition, intentionally *SHELXS* is used and not *SHELXT* (Sheldrick, 2015*a* ▸) because one challenge is that the students have to assign the atom types to the electron density peaks by themselves. In this way, chemical knowledge about the bonding properties of certain atom types in organic molecules can be practised and verified by the results in the refinement step. Alternatively, the *OLEX2* charge-flipping method gives similar results.

Following the determination of the crystal structure of acetyl­salicylic acid, the students can – if there is still time – determine up to two other structures of compounds relevant to everyday life. They have to use the knowledge acquired in the tutorial without the need for explicit instructions. Unlike with aspirin, they do not know the molecular structure beforehand but only receive the gross formula of the molecule as information. We use datasets from paracetamol, which allows a comparison with aspirin, and from ascorbic acid (vitamin C), where the structure determination is complicated by two molecules in the asymmetric unit. If interest is aroused, the students can also receive further datasets and the computer programs, which they can work on independently at home.

#### 4.2.2. Benefit for chemistry lessons in school

By solving and refining structures, fundamental knowledge of structural chemistry can be made familiar to students through their own experience. Some of the results may sound trivial to the experienced chemist: molecules have a three-dimensional structure. Inexperienced school students have to be made aware of this again and again, because in everyday school life they usually work with two-dimensional representations of molecules, *e.g.* Lewis formulae. Secondly, understanding of the nature of a chemical bond can be trained: chemical bonds are characterized by atomic distances and geometries, not by ‘dashes in a formula’. Practising such typical geometries helps with setting up Lewis formulae and with many concepts based on the three-dimensional structure of molecules, such as polarity and intermolecular forces, delocalization and aromaticity, stereoisomerism, or steric effects in reactions. The students can make their own assumptions when categorizing atom types according to the structure solution and check these themselves when refining them using the experimental data. Finally, the students realize that the result of the X-ray structure analysis is always a structural *model*, but a model with a certain agreement with experimental data. Appropriate and critical handling of models is a basic competence in chemistry lessons, because every representation at the molecular level leads the students to the model level. Producing models by students is to be regarded as the highest level of modelling activities (Gilbert, 2004 ▸).

## 5. Next steps

‘Crystallography in school’ is a project in progress. Even though the response from students, particularly with regard to the courses on X-ray structure analysis, has been very positive, the materials and computer programs on crystallography for students at school require further development. Empirical evaluation is an urgent task for the future. In addition, we are currently looking for ways to familiarize students with hands-on crystal selection, even if the diffractometer laboratory is not accessible at the time. To this purpose, we are using a low-cost digital polarization microscope and are currently developing a hands-on functional model for a diffractometer.

**Figure 8:**
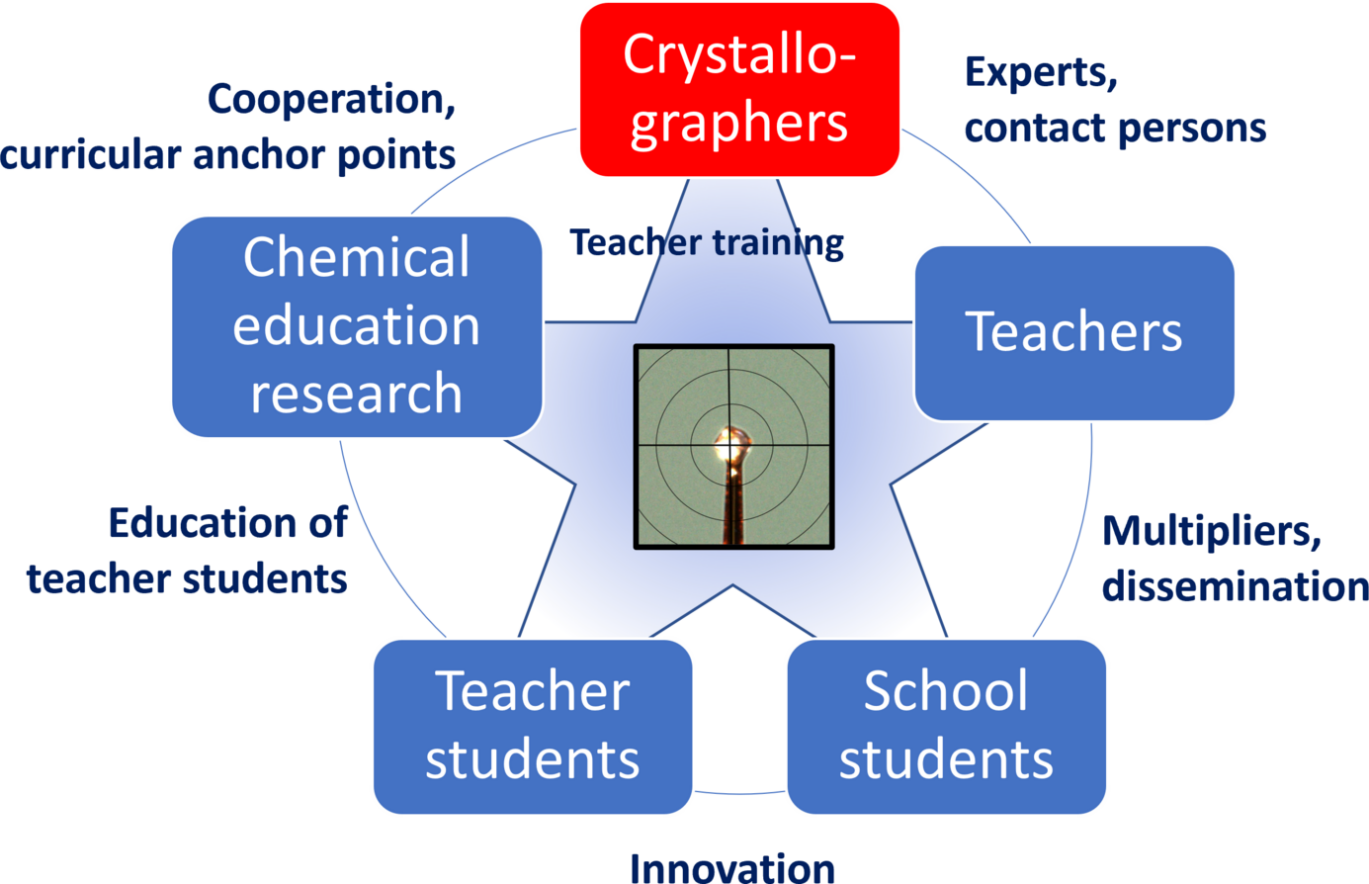
Network scheme for promoting crystallographic topics in the classroom.

In order to transport crystallographic topics into school, it seems problematic to rely only on crystallographers introducing as many school students as possible to the method. The first possible disseminators (Fig. 8 ▸) are the teachers, who can be introduced to crystallographic topics and then pass on this knowledge to the students. Particular attention should be paid to the training of science education students who are studying to become teachers. These ‘teacher students’ can bring innovations into schools and should be familiarized with the method of X-ray structure analysis and the opportunities this method offers in STEM education during their studies. Chemical education researchers can provide support in this respect by liaising with the teacher students on the one hand, and on the other hand by working with them on the development of teaching materials and methods.

A one-and-a-half-day workshop ‘Crystallography in school’ as a satellite meeting at ECM35 in Poznań (Poland) will be a further step towards this networking. The workshop will be located at St John Cantius school, former Friedrich Wilhelm Gymnasium, where the young Max von Laue was a pupil from 1887 to 1891. It will not only be aimed at crystallographers but also involve teachers and school students.

## Supplementary Material

Instructions for the use of the materials. DOI: 10.1107/S1600576725007459/dv5027sup1.pdf

Quick guide for Jmol with lysozyme (Word file) and pdb entry 1IEE.pdb. DOI: 10.1107/S1600576725007459/dv5027sup2.zip

CSD teaching subset sorted by substance classes - a selection of school-relevant organic structures and installation notes (in English and German). DOI: 10.1107/S1600576725007459/dv5027sup3.zip

PowerPoint slides for the basic level of the didactical reduction of the theory. DOI: 10.1107/S1600576725007459/dv5027sup4.pptx

PowerPoint slides for the advanced level of didactical reduction of the theory. DOI: 10.1107/S1600576725007459/dv5027sup5.pptx

Step-by-step-Guide Aspirin OLEX2. DOI: 10.1107/S1600576725007459/dv5027sup6.pdf

Step-by-step-Guide Aspirin ShelXle. DOI: 10.1107/S1600576725007459/dv5027sup7.pdf

X-ray data sets for aspirin, paracetamol and ascorbic acid with instructions. DOI: 10.1107/S1600576725007459/dv5027sup8.zip

PDF of PowerPoint slides for the basic level of the didactical reduction of the theory. DOI: 10.1107/S1600576725007459/dv5027sup9.pdf

PDF of PowerPoint slides for the advanced level of didactical reduction of the theory. DOI: 10.1107/S1600576725007459/dv5027sup10.pdf

## Data Availability

The supporting information contains the following material: instructions for the use of the materials in the supporting information; the *PowerPoint* slides for the basic and advanced level of theory; the worksheet for analysing the lysozyme structure 1iee and the corresponding PDB file; the CSD Teaching Subset sorted by substance classes; the step-by-step tutorials for the aspirin course with *ShelXle* and *OLEX2*; and the aspirin, paracetamol and ascorbic acid datasets.
