# Supplementary material for: Crystallography in school
Source: J Appl Crystallogr. 2025 Sep 12;58(Pt 5):1802–9. doi: 10.1107/S1600576725007459 (PMC12502877; doi:10.1107/S1600576725007459)
Supplement: Supplementary file 1 [file j-58-01802-sup1.pdf]

Supporting information

S1. Overview

The supporting information includes the following materials:

- This Word document containing instructions how to use the materials
- A zipped archive with the Quick guide for Jmol with lysozyme (Word file) and the PDB entry 1IEE.pdb
- A zipped archive with the CSD teaching subset sorted by substance classes – A selection of school-relevant organic structures, together with installation notes
- A PowerPoint document with PowerPoint Slides for the basic level of didactic reduction of the theory
- A PowerPoint document with PowerPoint Slides for the advanced level of didactic reduction of the theory
- A pdf document with the step-by-step-guide for the aspirin structure with *OLEX2*
- A pdf document with the step-by-step-guide for the aspirin structure with *ShelXle*
- A zipped archive with the X-ray data sets for aspirin, paracetamol and ascorbic acid with instructions

S2. Instructions how to use the materials – some ideas

S2.1. Protein structures from the PDB

The molecular viewer Jmol can be downloaded at https://jmol.sourceforge.net/download/. The pdb file for the structure of hen egg white lysozyme 1IEE (Sauter *et al.*, 2001) is found in the zip archive. This activity aims at high school students when learning about proteins and amino acids in the chemistry class but also works well with biology students in high school. Exploring the structure of this small protein needs about 90 minutes, starting with a presentation of the basic level of didactic reduction of the theory of X-ray structure determination (about 20 minutes). After that the students explore the structure of lysozyme following the instructions in the worksheet ‘Quick guide for Jmol with lysozyme’. The students are encouraged to ‘play around’ with the structure. Alternatively, the properties of the protein can be presented by the instructor and after that the students try to transfer their knowledge by testing out other proteins. The ‘Molecule of the Month’ area of the PDB website can be a very good starting point for individual research.

IMPORTANT: this document contains embedded data - to preserve data integrity, please ensure where possible that the IUCr Word tools (available from http://journals.iucr.org/services/docxtemplate/) are installed when editing this document.

S2.2. Crystal structures from the CSD Teaching Subset

For the installation of the CSD Teaching Subset categorised by substance classes please follow the installation notes which are provided in a German and an English version included in the zip archive. After installation there is direct access to about 100 organic structures sorted by school-relevant substance classes via a pdf document. The structures can be opened in WebCSD or directly by Mercury (downloadable via https://www.ccdc.cam.ac.uk/solutions/software/free-mercury/). For this reason the cif files of the structures are included in the zip file. The folder structure in the zip file needs to be kept unchanged when unzipping the archive.

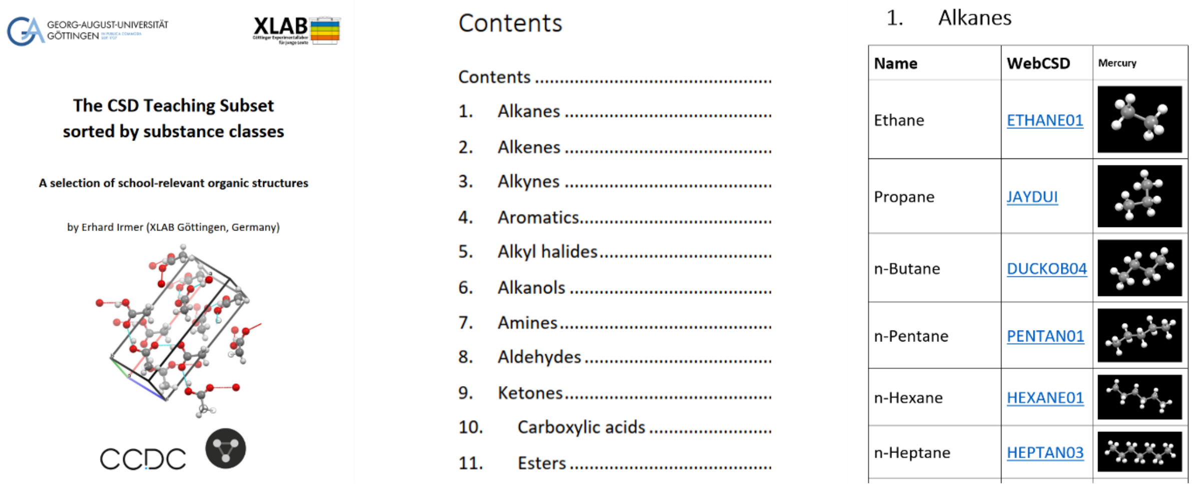

Figure S1 Excerpts from the CSD Teaching Subset sorted by substance classes. The structures can be accessed by clicking on the link (WebCSD) or the 3D model (Mercury).

Use of the CSD Teaching Subset is possible when students have learned about molecular compounds (covalent bonding, Lewis formulae). Again, an introduction into the theory of X-ray structure determination on a basic level is necessary (see above). Good experience was made with introducing the students to the use of Mercury on a simple example (e.g. aspirin; there is a tutorial for this structure in the 'tutorials' folder of the zip archive) and then using the structure viewer every time a new substance class or a new concept (e.g. aromaticity, H-bonding, …) is introduced in the chemistry lessons. After a while, it will be completely normal for the students to look on the structure of the molecules via Mercury. If the students bring their own electronic device (laptop, tablet or mobile phone) accessing the structure via WebCSD is easier and doesn't need the use of a computer with Mercury installed. Examples for tutorials in German (VSEPR model, H-bonding, Aromaticity) can be found in the zip archive in the folder 'Tutorials'. The tutorials can be also used by the students for individual training at home.

S2.3. Crystal structure solution and refinement by students

The XLAB course 'Aspirin – synthesis and analysis' typically lasts from 9 am to 5 pm with one hour lunch break. The students are from high school chemistry classes. The number of participants is limited to 20 due to the space in the laboratory. Normally, groups are smaller (12-16 persons). As described in the article, in the morning the students synthesise and recrystallise acetylsalicic acid (aspirin). During the lunch break (12:30 – 13:30) the samples are dried in a drying oven.

The presentation of the theory is split up in two parts. Before going to the diffraction lab, the students learn about the basics (basic level of the didactical reduction of the theory) in the seminar room of XLAB via a PowerPoint presentation. In the Department of X-ray structure analysis in the Faculty of Chemistry I hand over the group to a PhD student working there. He or she explains how X-rays are generated, the setup of the diffractometer and introduces them into the selection of a suitable crystal. The students can try to find a crystal under the polarisation microscope and place it on the loop of a pin. To avoid boring waiting time, a second microscope may be used. If the group is very large (more than 12 participants) the group has to be split up guided through the lab by another instructor. Alternatively, one part of the group has to get the advanced level theory before visiting the diffraction lab. If the diffractometers aren't used for regular measurements a crystal will be put on the machine and a few frames with reflections will be measured. Alternatively, some prepared frames could be shown on the computer.

Back at XLAB, the students get the presentation about the advanced level of theory. For the determination of the aspirin structure following the step-by-step tutorial about 60 – 90 minutes are needed. The students are provided in groups of two or three with a laptop with installed *ShelXle XLAB Edition* or *OLEX2* and an iPad where they find the step-by-step tutorial. On the desktop of the laptop, they find an ins and an hkl file for the aspirin structure in a corresponding folder.

OLEX2 can be downloaded for free at https://www.olexsys.org/olex2/.

The *ShelXle XLAB Edition*, which will also install *SHELXS* and *SHELXL*, is available from the author Christian Hübschle; please send an email to: erhard.irmer@chemie.uni-goettingen.de.

If students are fast or if more time is available, the students can work on further structures. In these cases, the students don't know the structure but get only the sum formula and they have to use the knowledge they gained by doing the tutorial to determine these unknown structures. In the case of the paracetamol structure (structure 2 in the folder 'further structures'), the challenge is to distinguish not only between carbon and oxygen atoms but also finding the nitrogen atom. Ascorbic acid (structure 3) is even a little bit more difficult because there are 2 molecules in the asymmetric unit and the solution by direct methods often doesn't give both molecules completely from the beginning.
