# Supplementary material for: Crystallography in school
Source: J Appl Crystallogr. 2025 Sep 12;58(Pt 5):1802–9. doi: 10.1107/S1600576725007459 (PMC12502877; doi:10.1107/S1600576725007459)
Supplement: Supplementary file 2 [file j-58-01802-sup2.zip › Quick guide Jmol with lysozyme.docx]

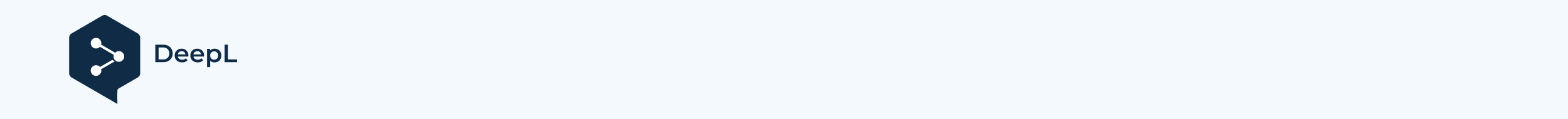


Subscribe to DeepL Pro to edit this document.
Visit [www.DeepL.com/pro](https://www.deepl.com/pro?cta=edit-document) for more information.

Quick guide (Jmol) for the
"Journey of discovery through the structure of the lysozyme"

1. **Search for a suitable structure of the lysozyme**:
   If you already know the pdb code, enter it in the field to the right of the "PDB-ID or text" field and then click on "Search". You can also enter the name of the protein ("lysozyme").
   Select the most current structure "3IJU" from the list at the time the manual was created. However, you can also use other structures (alternatively you can also use "1IEE").
   There are now two options:
   a) Direct viewing in the 3D viewer of the browser or
   b) Viewing in the standalone version of the Jmol program
2. **Call up the Jmol program:**
   a) Click on "View in 3D": A new window opens (agree to the browser's security prompt if necessary).
   b) Download the selected structure to your computer ("Download files" at the top right of the window next to the PDB code, "PDB File (Text)", specify a storage location).
   Now start the Jmol program (in the OHG suite (sunflower symbol): "Subjects - Chemistry - Jmol Portable Launcher"). Open the downloaded pdb file by navigating to the folder with the saved structural data under "File - Open en".
3. **Viewing with** Jmol
   In the window with the structure display in the cartoon view, you can now start by playing around: Rotate, move, zoom (see Tab. 1 for commands). If in doubt, use the "house symbol" to return to the starting position or simply reload the files.

Table 1: Overview of the basic control of Jmol

| **Button(s)** | **Movement** |
| --- | --- |
| left mouse button | Free rotation (around the center of gravity) |
| right mouse button | Calling up the selection and function menu |
| Mouse wheel | Zoom |
| right mouse button + CTRL or CTRL | Shift in the monitor level |
| left mouse button + SHIFT or ⇧ | Rotation in the screen plane + zoom |

1. We are looking at the **"surface"** of the lysozyme:
   Right-click in the image to open a context menu. Select "Surfaces - van der Waals surface" and then "Surfaces - Make opaque". Rotate the molecule and explore the surface structure, find the active pocket. Remove the surface via "Surfaces - Off".
2. **From the tertiary structure to the atomic level model**:
   Follow the instructions given (Tab. 2). Right-click on the image to open the context menu where you will find the other commands. Always try to keep in mind what you are looking at.

Tab. 2

1. Context menu → Selection→ Protein→ All
2. Context menu→ Selection→ Protein→ Primary structure
3. Context menu→ Selection→ Show selection only
4. Context menu→ Style→ Scheme→ Rods
5. Context menu→ Color→ Atoms→ according to scheme → Secondary structure
6. Context menu→ Selection→ Protein→ everything
7. Context menu→ Style→ Scheme→ Rods
8. Context menu→ Color→ Atoms→ by scheme→ Secondary structure
9. Context menu→ Color→ Atoms→ according to scheme → Element (CPK)
10. Examination of the **secondary structure elements**:
    The commands that you enter via the context menu can also be executed using the scripting language implemented in Jmol. To do this, use "File - Console" to open a separate window in which you can enter commands.

Tab. 3: Step-by-step representation of the first α-helix in the lysozyme including the hydrogen bonds using the script language in Jmol.

| Command | Action |
| --- | --- |
| restrict 3-16 and backbone | Select and show only the main chain atoms of the amino acids 3-16 |
| center 3-16 | Centering on this section |
| zoom 300 | Enlargement to 300% (not absolutely necessary) |
| wireframe 0.15 | Representation of the bonds between the atoms |
| spacefill 0.45 | Representation of atoms as spheres |
| color cpk | Coloring the atoms according to the elements |
| cartoon off | Hiding the helix structure |
| calculate hbonds | Calculation of hydrogen bonds |
| hbonds 50 | Representation of hydrogen bonds |
| color hbonds white | Coloring the hydrogen bonds |

Proceed in the same way by exploring the structure of the β-folder area 42-60

1. And now the most important thing: Try out all possible settings (either via the main menu or via the context menu (right-click in the image).

*This quick guide follows the procedure in the article:
T. Pape, The third dimension - Spatial representation of protein structures on the computer. PdN-ChiS* ***60*** *(2), 2011, pp. 23-27*
