# Supplementary material for: Crystallography in school
Source: J Appl Crystallogr. 2025 Sep 12;58(Pt 5):1802–9. doi: 10.1107/S1600576725007459 (PMC12502877; doi:10.1107/S1600576725007459)
Supplement: Supplementary file 3 [file j-58-01802-sup3.zip › Installationshinweise CSD_Teaching_Materials_EI.docx]

**CSD Teaching Materials**

**Installationshinweise**

Entpacken Sie die „CSD_Teaching_Materials_EI.zip“-Datei an einen gewünschten Ort auf Ihrem Rechner. Ändern Sie bitte danach die Ordnerstruktur nicht, da die Verweise zwischen der Liste an Teaching-Subset-Strukturen und den Dateien in den anderen Ordnern sonst nicht mehr funktionieren.

Ein direktes Aufrufen der Struktur in Mercury ist nur möglich, wenn die Datei-Endung „cif“ mit dem Programm Mercury verknüpft ist. Bei der Installation von Mercury wird normalerweise diese Verknüpfung erstellt. Ansonsten muss die Verknüpfung selbst gesetzt werden: Bei Rechtsklick auf eine *.cif-Datei wählen Sie „Öffnen mit…“ aus, suchen Sie unter „Weitere Apps“ das Programm „Mercury“ und setzen eine Haken bei „Immer diese App zum Öffnen von .cif-Dateien verwenden“.
