# Supplementary material for: Crystallography in school
Source: J Appl Crystallogr. 2025 Sep 12;58(Pt 5):1802–9. doi: 10.1107/S1600576725007459 (PMC12502877; doi:10.1107/S1600576725007459)
Supplement: Supplementary file 3 [file j-58-01802-sup3.zip › Teaching Subset nach Stoffklassen.docx]

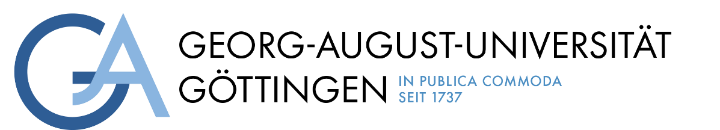

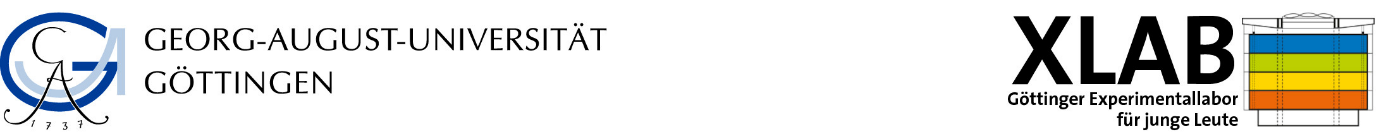


**Der CSD Teaching Subset
nach Stoffklassen sortiert**

**Eine Auswahl von schulrelevanten Strukturen**

von Erhard Irmer (XLAB Göttingen, Germany)

**
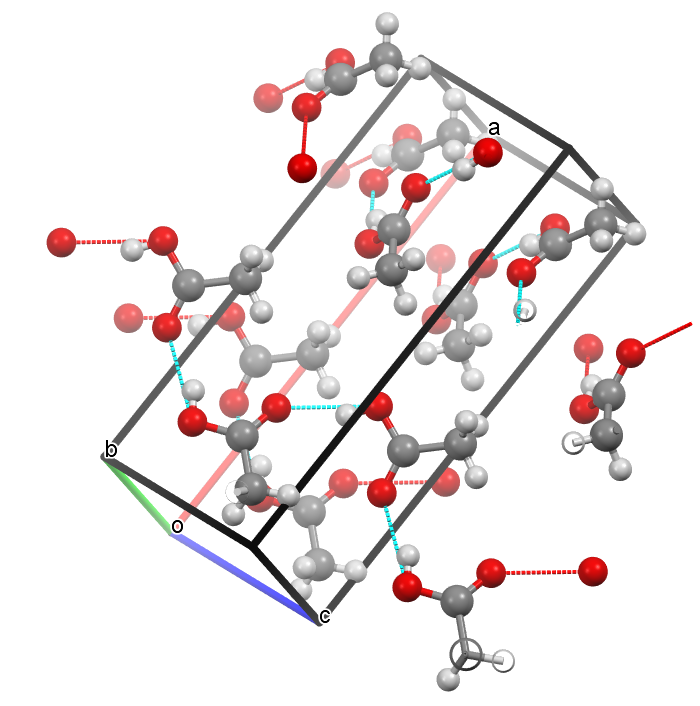
**


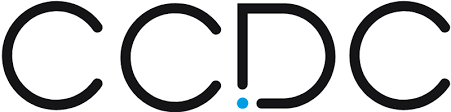

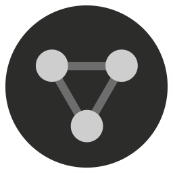


# Inhalt

[Inhalt 2](#_Toc142922271)

[1. Alkane 3](#_Toc142922272)

[2. Alkene 4](#_Toc142922273)

[3. Alkine 5](#_Toc142922274)

[4. Aromaten 5](#_Toc142922275)

[5. Halogenkohlenwasserstoffe 7](#_Toc142922276)

[6. Alkanole 8](#_Toc142922277)

[7. Amine 9](#_Toc142922278)

[8. Aldehyde 10](#_Toc142922279)

[9. Ketone 10](#_Toc142922280)

[10. Carbonsäuren 11](#_Toc142922281)

[11. Ester 12](#_Toc142922282)

[12. Aminosäuren 12](#_Toc142922283)

[13. Peptide 15](#_Toc142922284)

[14. Kohlenhydrate 15](#_Toc142922285)

[15. Vitamine 16](#_Toc142922286)

[16. Naturstoffe 16](#_Toc142922287)

[17. Polymere 17](#_Toc142922288)

[Alphabetisches Inhaltsverzeichnis 18](#_Toc142922289)

# Alkane

| **Name** | **Refcode** | **Mercury** |
| --- | --- | --- |
| Ethan | [ETHANE01](CIF/Alkanes/ETHANE01.cif) | [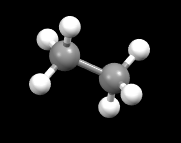](CIF/Alkanes/ETHANE01.cif) |
| Propan | [JAYDUI](CIF/Alkanes/JAYDUI.cif) | [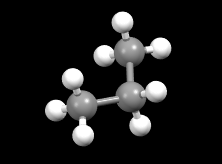](CIF/Alkanes/JAYDUI.cif) |
| n-Butan | [DUCKOB04](CIF/Alkanes/DUCKOB04.cif) | [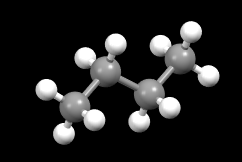](CIF/Alkanes/DUCKOB04.cif) |
| n-Pentan | [PENTAN01](CIF/Alkanes/PENTAN01.cif) | [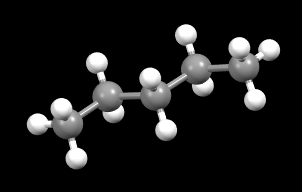](CIF/Alkanes/PENTAN01.cif) |
| n-Hexan | [HEXANE01](CIF/Alkanes/HEXANE01.cif) | [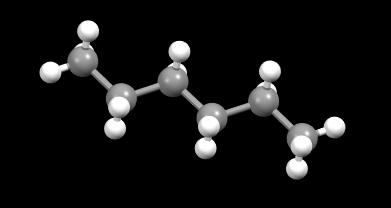](CIF/Alkanes/HEXANE01.cif) |
| n-Heptan | [HEPTAN03](CIF/Alkanes/HEPTAN03.cif) | [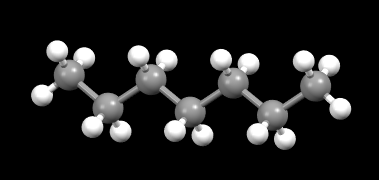](CIF/Alkanes/HEPTAN03.cif) |
| n-Octan | [OCTANE12](CIF/Alkanes/OCTANE12.cif) | [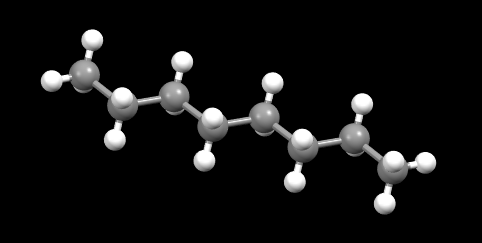](CIF/Alkanes/OCTANE12.cif) |
| Cyclopropan | [QQQCIS01](CIF/Alkanes/QQQCIS01.cif) | [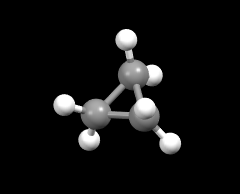](CIF/Alkanes/QQQCIS01.cif) |
| Cyclobutan | [ZZZWEO02](CIF/Alkanes/ZZZWEO02.cif) | [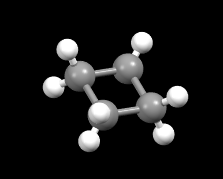](CIF/Alkanes/ZZZWEO02.cif) |
| Cyclohexan | [CYCHEX](CIF/Alkanes/CYCHEX.cif) | [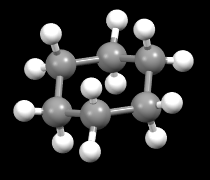](CIF/Alkanes/CYCHEX.cif) |

# Alkene

| **Name** | **Refcode** | **Mercury** |
| --- | --- | --- |
| Ethen | [ETHLEN10](CIF/Alkenes/ETHLEN10.cif) | [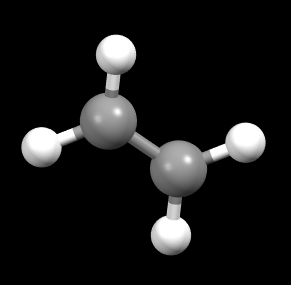](CIF/Alkenes/ETHLEN10.cif) |
| Tetramethylethen | [PAPVAD](CIF/Alkenes/PAPVAD.cif) | [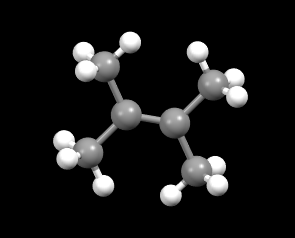](CIF/Alkenes/PAPVAD.cif) |
| 1,7-Octadien | [XOMHUC](CIF/Alkenes/XOMHUC.cif) | [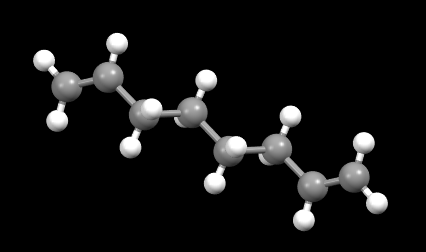](CIF/Alkenes/XOMHUC.cif) |
| 1,9-Decadien | [XOMJAK](CIF/Alkenes/XOMJAK.cif) | [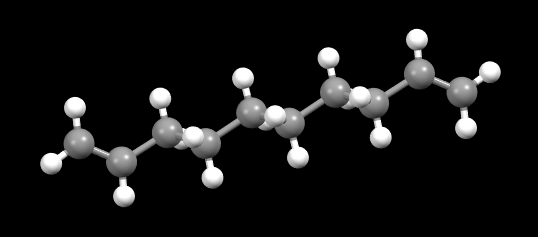](CIF/Alkenes/XOMJAK.cif) |
| Cyclohexen | [COVJON](CIF/Alkenes/COVJON.cif) | [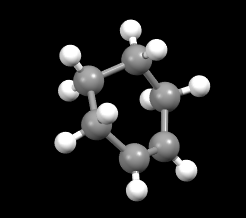](CIF/Alkenes/COVJON.cif) |
| Cyclo-octatetraen | [ZZZSAE01](CIF/Alkenes/ZZZSAE01.cif) | [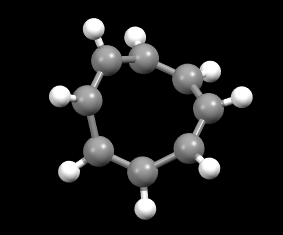](CIF/Alkenes/ZZZSAE01.cif) |

# Alkine

| **Name** | **Refcode** | **Mercury** |
| --- | --- | --- |
| Ethin, Acetylen | [ACETYL03](CIF/Alkynes/ACETYL03.cif) | [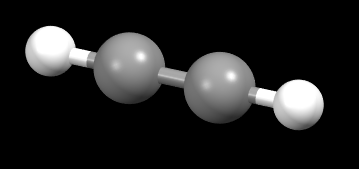](CIF/Alkynes/ACETYL03.cif) |
| But-2-in Hydrogenchlorid | [JUFDUJ](CIF/Alkynes/JUFDUJ.cif) | [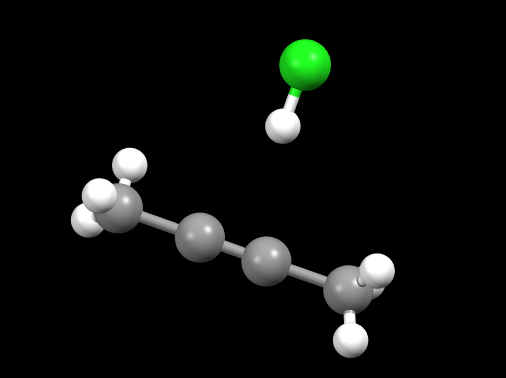](CIF/Alkynes/JUFDUJ.cif) |
| Octa-2,4,6-triin | [OCTRNE](CIF/Alkynes/OCTRNE.cif) | [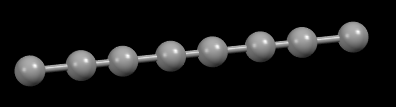](CIF/Alkynes/OCTRNE.cif) |
| Cyanoacetlyen | [CAACTY](CIF/Alkynes/CAACTY.cif) | [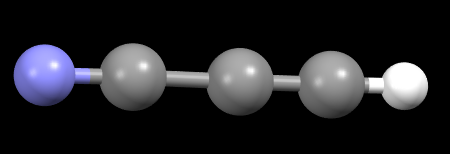](CIF/Alkynes/CAACTY.cif) |
| 1,7-Octadiin | [XOMJEO](CIF/Alkynes/XOMJEO.cif) | [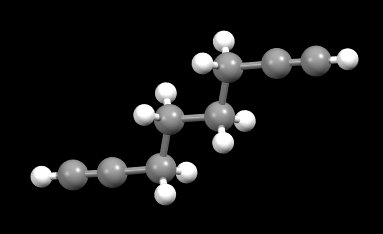](CIF/Alkynes/XOMJEO.cif) |

# Aromaten

| **Name** | **Refcode** | **Mercury** |
| --- | --- | --- |
| Benzol (neutron diffraction) | [BENZEN](CIF/Aromatics/BENZEN.cif) | [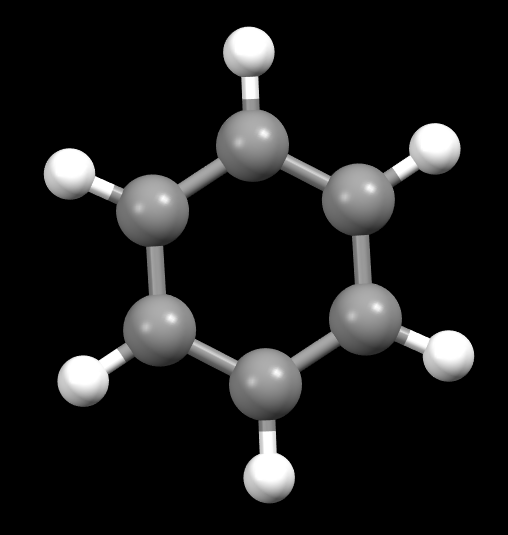](CIF/Aromatics/BENZEN.cif) |
| Benzol | [BENZEN02](CIF/Aromatics/BENZEN02.cif) | [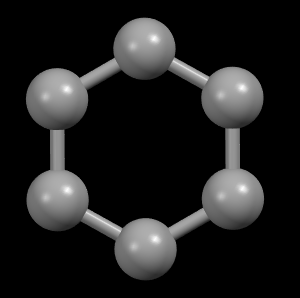](CIF/Aromatics/BENZEN02.cif) |
| Toluol | [TOLUEN](CIF/Aromatics/TOLUEN.cif) | [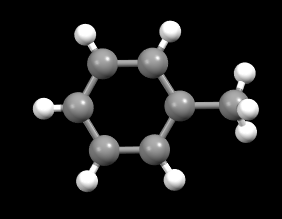](CIF/Aromatics/TOLUEN.cif) |
| Phenol | [PHENOL03](CIF/Aromatics/PHENOL03.cif) | [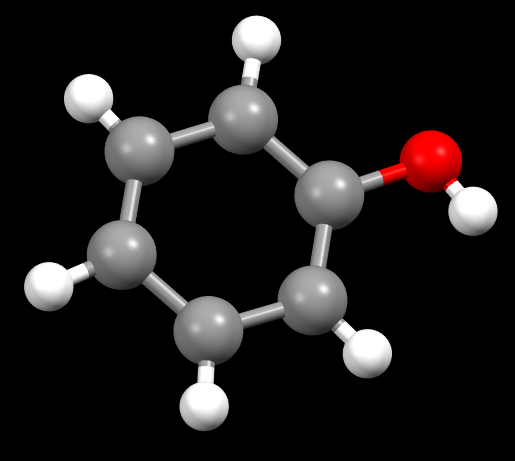](CIF/Aromatics/PHENOL03.cif) |
| Catecholin, 2-Hydroxyphenol | [CATCOL13](CIF/Aromatics/CATCOL13.cif) | [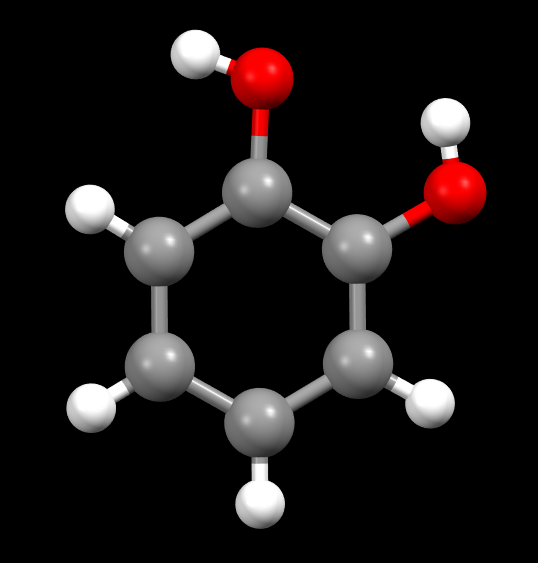](CIF/Aromatics/CATCOL13.cif) |
| 2-Amino-5-nitrophenol | [AMNPHA](CIF/Aromatics/AMNPHA.cif) | [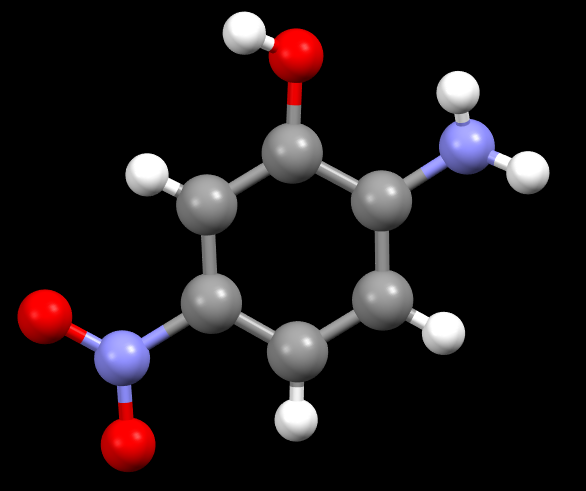](CIF/Aromatics/AMNPHA.cif) |
| 1,3,5-Trinitrobenzol | [TNBENZ12](CIF/Aromatics/TNBENZ12.cif) | [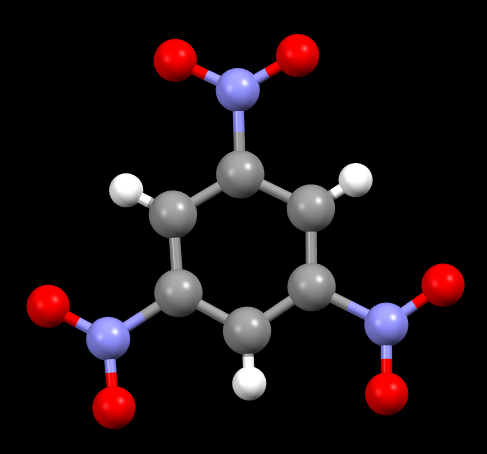](CIF/Aromatics/TNBENZ12.cif) |
| 2,4,6-Trinitrotoluol | [ZZZMUC01](CIF/Aromatics/ZZZMUC01.cif) | [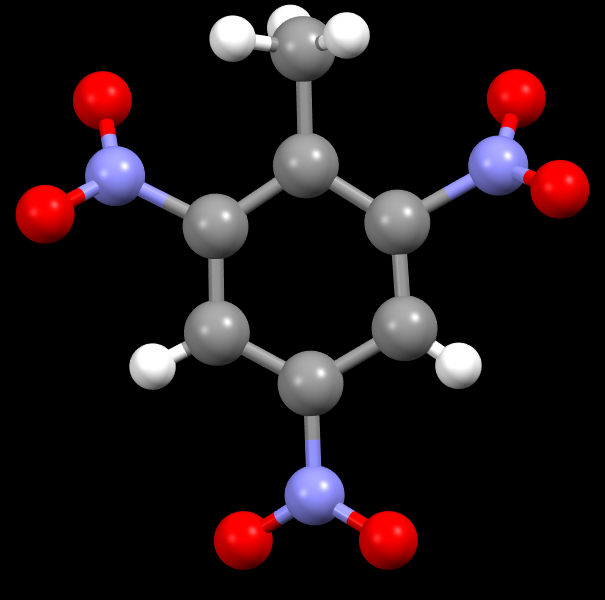](CIF/Aromatics/ZZZMUC01.cif) |
| Hexaaminobenzol | [ZZZWOU01](CIF/Aromatics/ZZZWOU01.cif) | [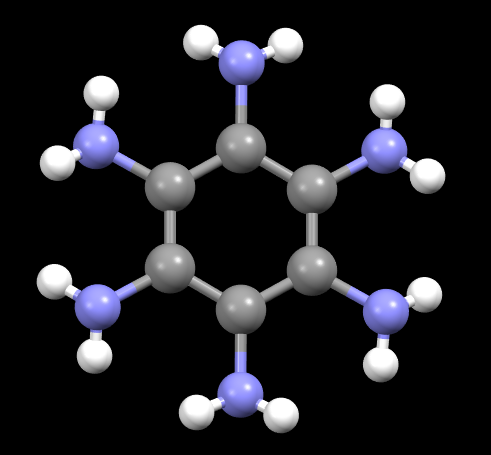](CIF/Aromatics/ZZZWOU01.cif) |
| Benzonitril | [BZONTR](CIF/Aromatics/BZONTR.cif) | [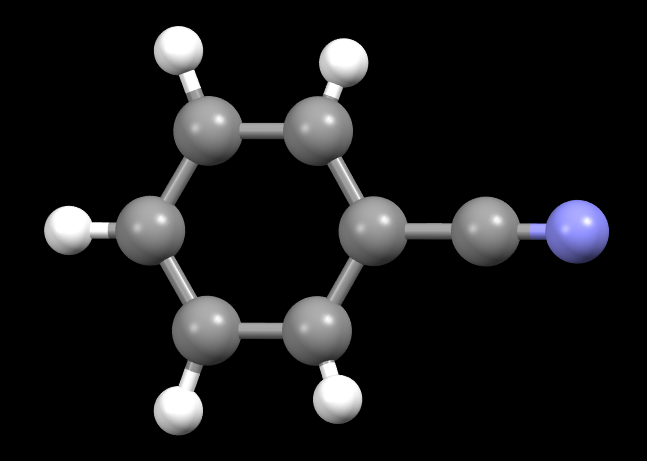](CIF/Aromatics/BZONTR.cif) |
| 5-Brom-1,3-dichlor-2-iod-benzol | [ACEPOO](CIF/Aromatics/ACEPOO.cif) | [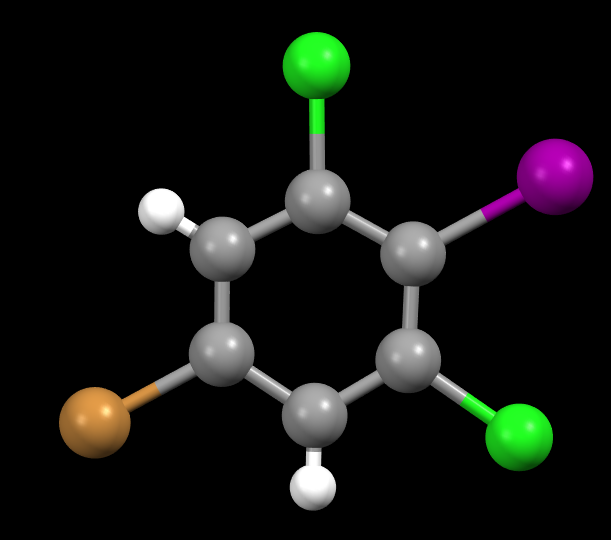](CIF/Aromatics/ACEPOO.cif) |
| Naphthol | [NAPHTA12](CIF/Aromatics/NAPHTA12.cif) | [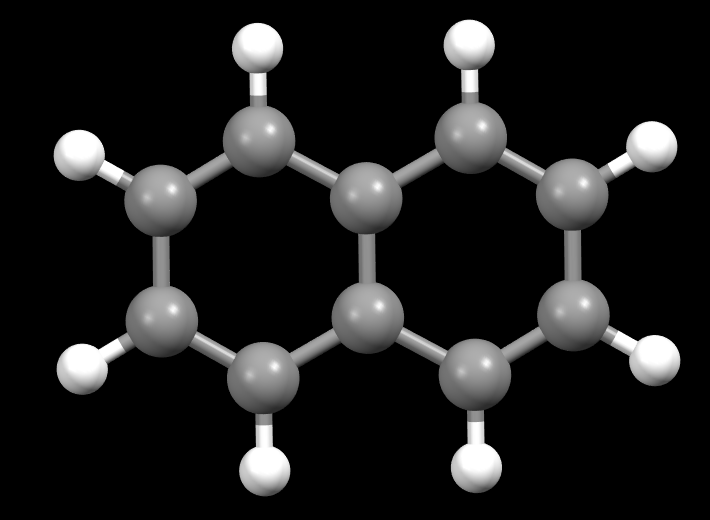](CIF/Aromatics/NAPHTA12.cif) |
| Anthrachinon | [ANTQUO08](CIF/Aromatics/ANTQUO08.cif) | [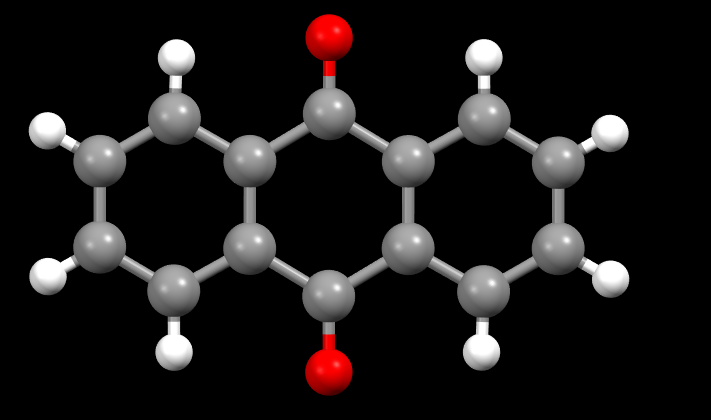](CIF/Aromatics/ANTQUO08.cif) |
| (16)Annulen | [ANNULE01](CIF/Aromatics/ANNULE01.cif) | [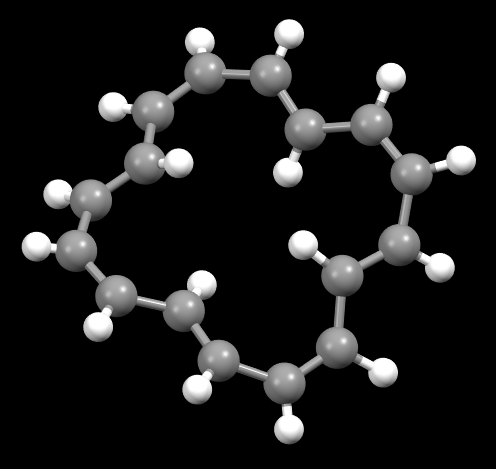](CIF/Aromatics/ANNULE01.cif) |
| (18)Annulen | [ANULEN](CIF/Aromatics/ANULEN.cif) | [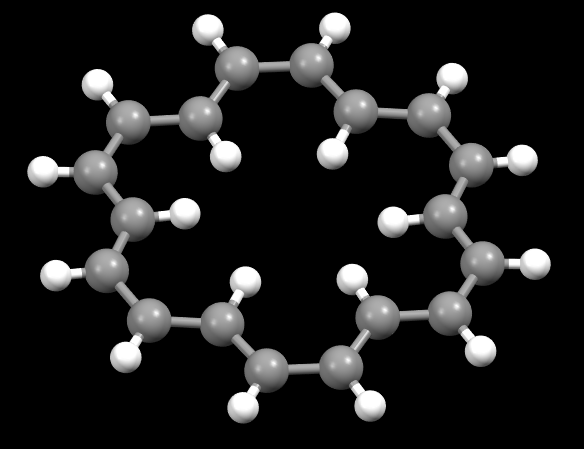](CIF/Aromatics/ANULEN.cif) |

# Halogenkohlenwasserstoffe

| **Name** | **Refcode** | **Mercury** |
| --- | --- | --- |
| Chlormethan | [CLMETH](CIF/Alkyl_halides/CLMETH.cif) | [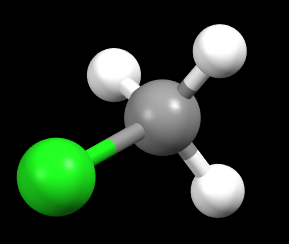](CIF/Alkyl_halides/CLMETH.cif) |
| Diiodmethan | [DIMETH03](CIF/Alkyl_halides/DIMETH03.cif) | [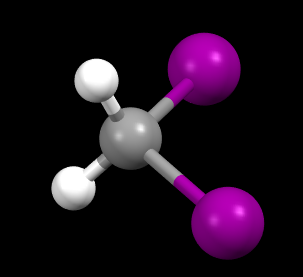](CIF/Alkyl_halides/DIMETH03.cif) |
| Brommethan | [MBRMET10](CIF/Alkyl_halides/MBRMET10.cif) | [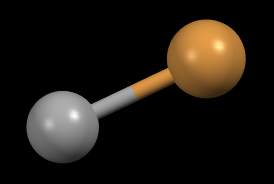](CIF/Alkyl_halides/MBRMET10.cif) |
| Iodmethan | [MIMETH10](CIF/Alkyl_halides/MIMETH10.cif) | [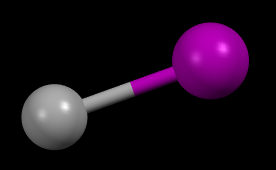](CIF/Alkyl_halides/MIMETH10.cif) |
| Tetraiodmethan | [ZZZKDW01](CIF/Alkyl_halides/ZZZKDW01.cif) | [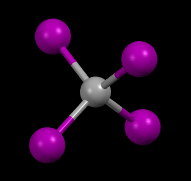](CIF/Alkyl_halides/ZZZKDW01.cif) |
| Dichlormethan (solvent) | [BEJKUW](CIF/Alkyl_halides/BEJKUW.cif) | [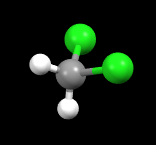](CIF/Alkyl_halides/BEJKUW.cif) |
| Dibromhexafluorpropan | [BOCGAB](CIF/Alkyl_halides/BOCGAB.cif) | [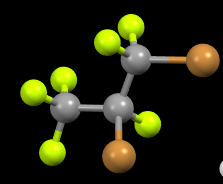](CIF/Alkyl_halides/BOCGAB.cif) |

# Alkanole

| **Name** | **Refcode** | **Mercury** |
| --- | --- | --- |
| Methanol | [METHOL](CIF/Alkanols/METHOL.cif) | [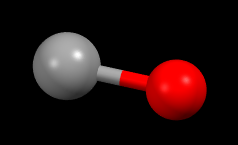](CIF/Alkanols/METHOL.cif) |
| Ethanol | [ETANOL](/CIF/Alkanols/ETANOL.cif) | [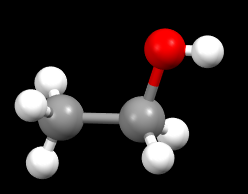](CIF/Alkanols/ETANOL.cif) |
| Propan-1-ol (solvent) | [VENVAM](CIF/Alkanols/VENVAM.cif) | [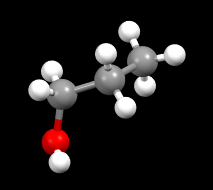](CIF/Alkanols/VENVAM.cif) |
| Isopropanol (solvent) | [ABALEV](CIF/Alkanols/ABALEV.cif) | [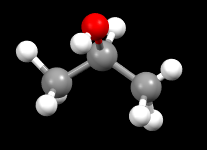](CIF/Alkanols/ABALEV.cif) |
| Butan-2-ol (solvent) | [AVEPIB](CIF/Alkanols/ABALEV.cif) | [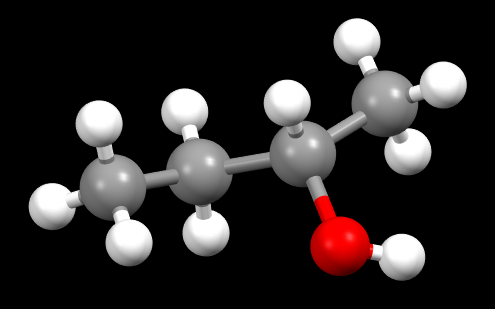](CIF/Alkanols/ABALEV.cif) |

# Amine

| **Name** | **Refcode** | **Mercury** |
| --- | --- | --- |
| 1,2-Diaminoethan | [ETDIAM12](CIF/Amines/ETDIAM12.cif) | [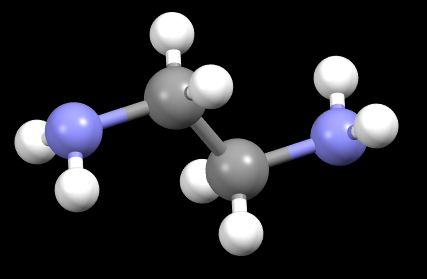](CIF/Amines/ETDIAM12.cif) |
| Methylamin | [METAMI](CIF/Amines/METAMI.cif) | [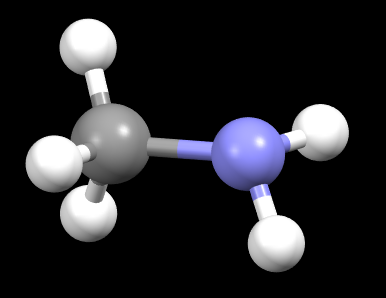](CIF/Amines/METAMI.cif) |
| Anilin | [BAZGOY](CIF/Amines/BAZGOY.cif) | [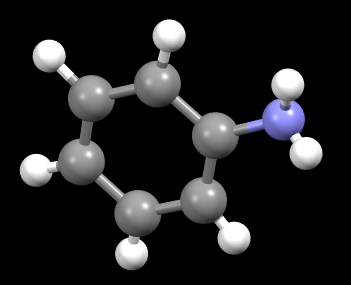](CIF/Amines/BAZGOY.cif) |
| Harnstoff | [UREAXX](CIF/Amines/UREAXX.cif) | [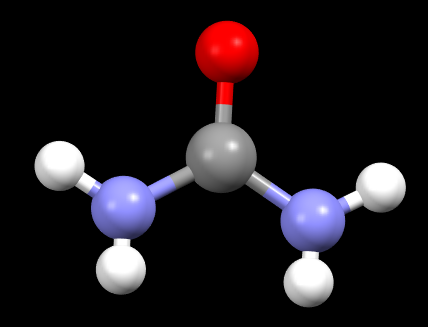](CIF/Amines/UREAXX.cif) |
| Trimethylamin | [CEKGUU01](CIF/Amines/CEKGUU01.cif) | [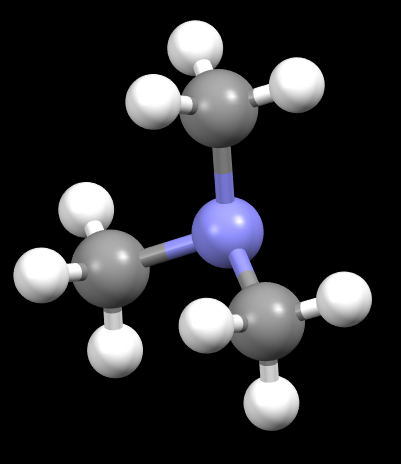](CIF/Amines/CEKGUU01.cif) |

# Aldehyde

| **Name** | **Refcode** | **Mercury** |
| --- | --- | --- |
| Formaldehyd | [GURNEN](CIF/Aldehydes/GURNEN.cif) | [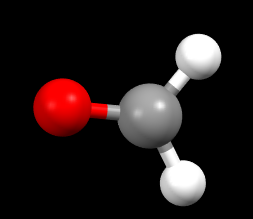](CIF/Aldehydes/GURNEN.cif) |

# Ketone

| **Name** | **Refcode** | **Mercury** |
| --- | --- | --- |
| Aceton | [HIXHIF05](CIF/Ketones/HIXHIF05.cif) | [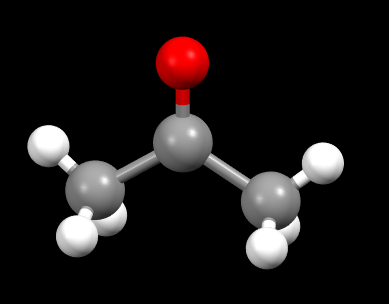](CIF/Ketones/HIXHIF05.cif) |
| Anthrachinon | [ANTQUO08](CIF/Ketones/ANTQUO08.cif) | [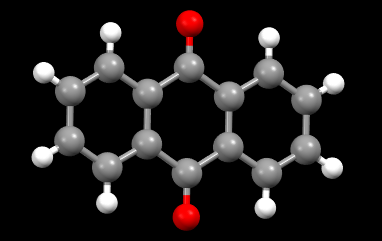](CIF/Ketones/ANTQUO08.cif) |
| Benzophenon | [BPHENO03](CIF/Ketones/BPHENO03.cif) | [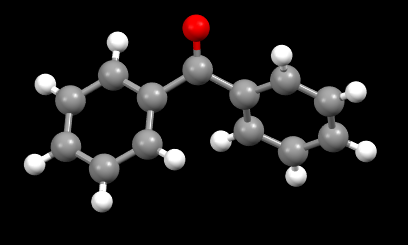](CIF/Ketones/BPHENO03.cif) |

# Carbonsäuren

| **Name** | **Refcode** | **Mercury** |
| --- | --- | --- |
| Ameisensäure, Methansäure | [FORMAC01](CIF/Carboxylic_acids/FORMAC01.cif) | [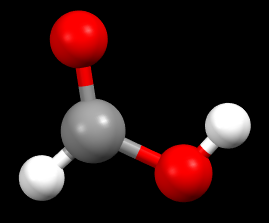](CIF/Carboxylic_acids/FORMAC01.cif) |
| Essigsäure, Ethansäure | [ACETAC07](CIF/Carboxylic_acids/ACETAC07.cif) | [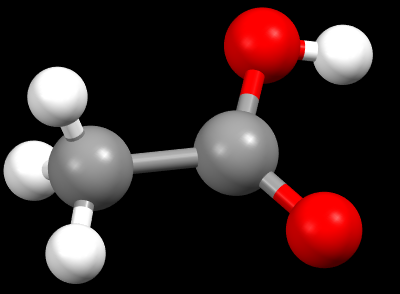](CIF/Carboxylic_acids/ACETAC07.cif) |
| Benzoesäure | [BENZAC02](CIF/Carboxylic_acids/BENZAC02.cif) | [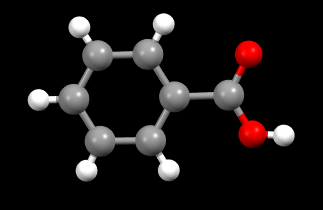](CIF/Carboxylic_acids/BENZAC02.cif) |
| Prop-2-en-säure | [ACRLAC02](CIF/Carboxylic_acids/ACRLAC02.cif) | [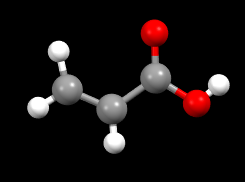](CIF/Carboxylic_acids/ACRLAC02.cif) |
| Adipinsäure | [ADIPAC04](CIF/Carboxylic_acids/ADIPAC04.cif) | [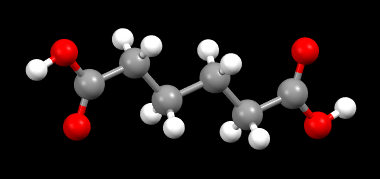](CIF/Carboxylic_acids/ADIPAC04.cif) |
| L-(+)-Milch | [YILLAG](CIF/Carboxylic_acids/YILLAG.cif) | [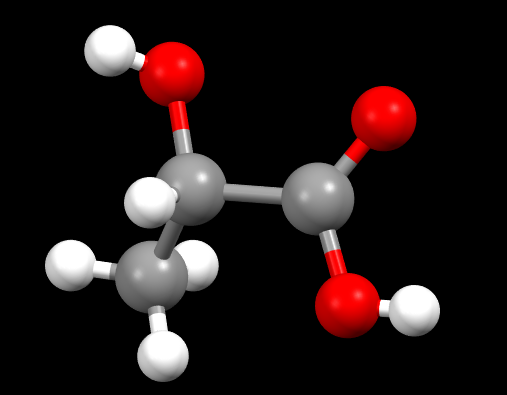](CIF/Carboxylic_acids/YILLAG.cif) |
| Citronensäure | [CITRAC10](CIF/Carboxylic_acids/CITRAC10.cif) | [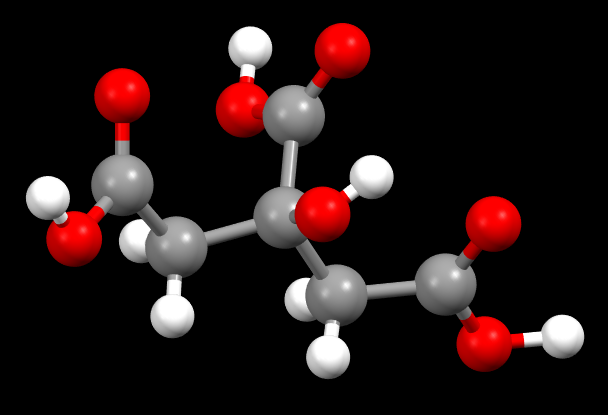](CIF/Carboxylic_acids/CITRAC10.cif) |
| Citronensäure Monohydrat | [CITARC](CIF/Carboxylic_acids/CITARC.cif) | [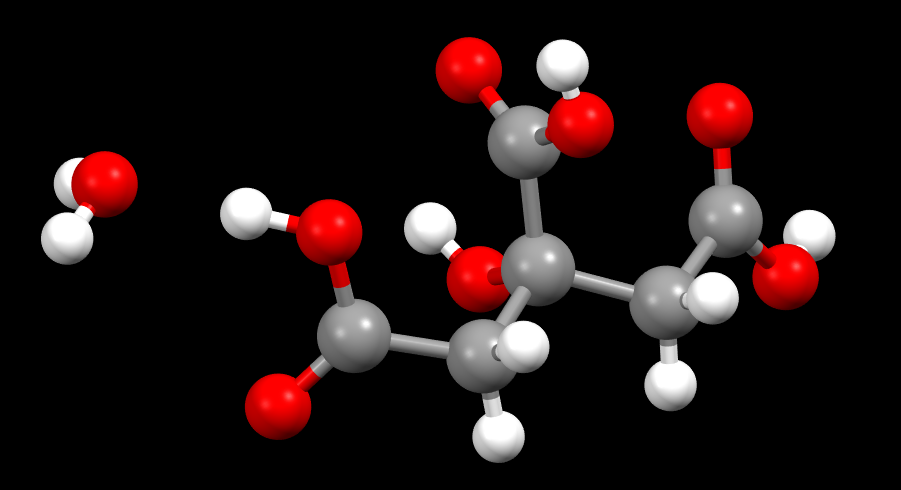](CIF/Carboxylic_acids/CITARC.cif) |
| Fumarsäure | [FUMAAC01](CIF/Carboxylic_acids/FUMAAC01.cif) | [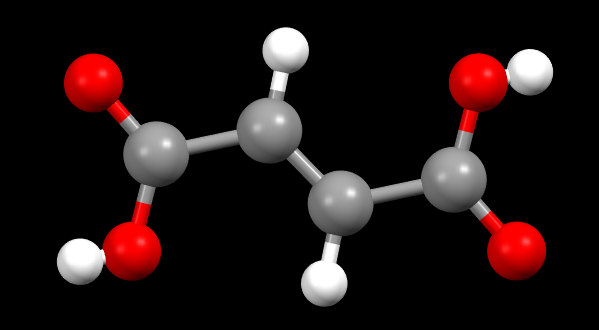](CIF/Carboxylic_acids/FUMAAC01.cif) |
| Monofluoressigsäure | [FACETC10](CIF/Carboxylic_acids/FACETC10.cif) | [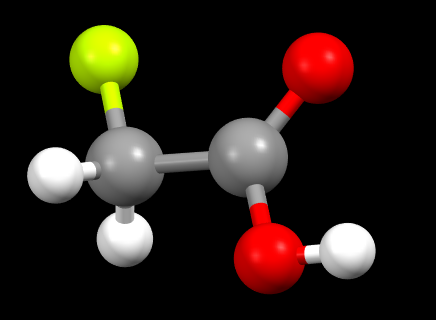](CIF/Carboxylic_acids/FACETC10.cif) |
| Natriumacetat | [BOPKOG1](CIF/Carboxylic_acids/BOPKOG10.cif)0 | [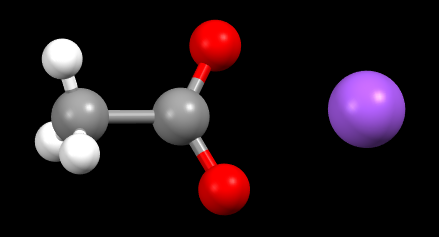](CIF/Carboxylic_acids/BOPKOG10.cif) |
| Calciumformiat | [CAFORM05](CIF/Carboxylic_acids/CAFORM05.cif) | [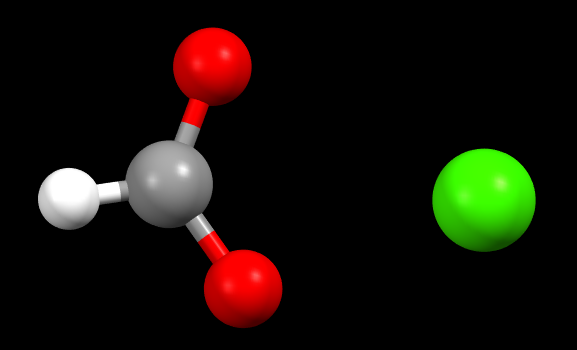](CIF/Carboxylic_acids/CAFORM05.cif) |

# Ester

| **Name** | **Refcode** | **Mercury** |
| --- | --- | --- |
| Methylacetat | [BAHSUY](CIF/Esters/BAHSUY.cif) | [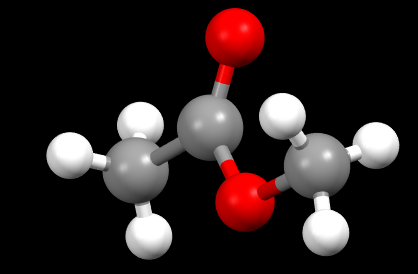](CIF/Esters/BAHSUY.cif) |
| Ethylpropionat | [YARZUN03](CIF/Esters/YARZUN03.cif) | [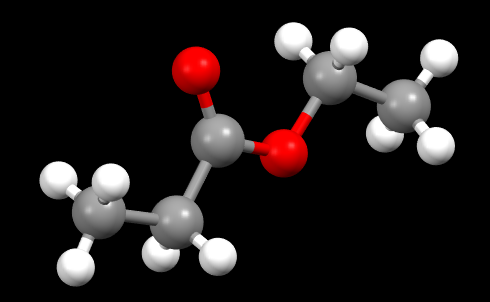](CIF/Esters/YARZUN03.cif) |

# Aminosäuren

| **Name** | **Refcode** | **Mercury** |
| --- | --- | --- |
| Glycin | [GLYCIN](CIF/Amino_acids/GLYCIN.cif) | [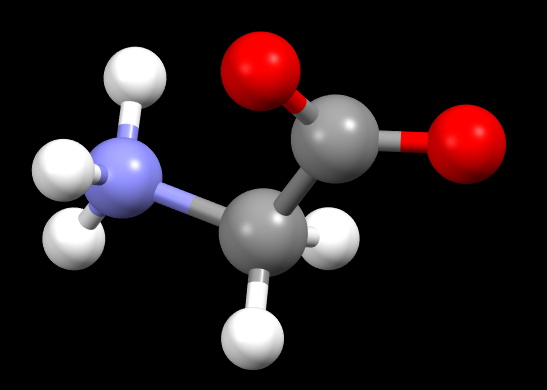](CIF/Amino_acids/GLYCIN.cif) |
| D-Alanin | [ALUCAL05](CIF/Amino_acids/ALUCAL05.cif) | [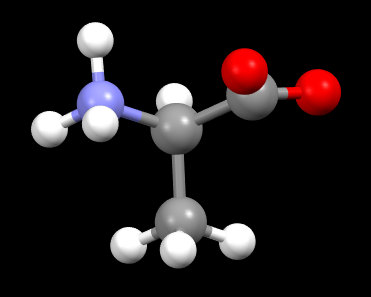](CIF/Amino_acids/ALUCAL05.cif) |
| L-Alanin | [LALNIN23](CIF/Amino_acids/LALNIN23.cif) | [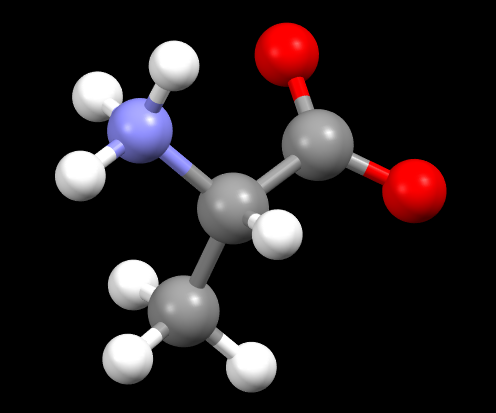](CIF/Amino_acids/LALNIN23.cif) |
| DL-Valin | [VALIDL](CIF/Amino_acids/VALIDL.cif) | [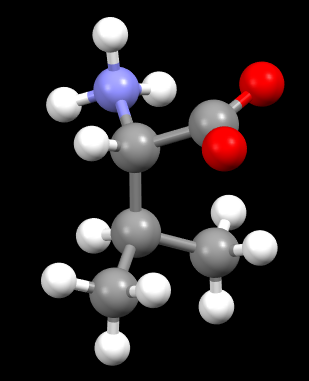](/CIF/Amino_acids/VALIDL.cif) |
| L-Prolin | [PROLIN](CIF/Amino_acids/PROLIN.cif) | [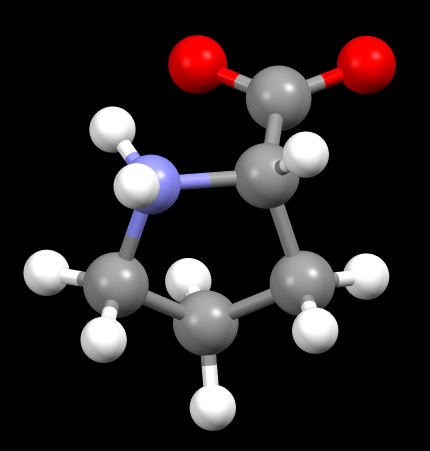](CIF/Amino_acids/PROLIN.cif) |
| L-Glutaminsäure | [LGLUAC01](CIF/Amino_acids/LGLUAC01.cif) | [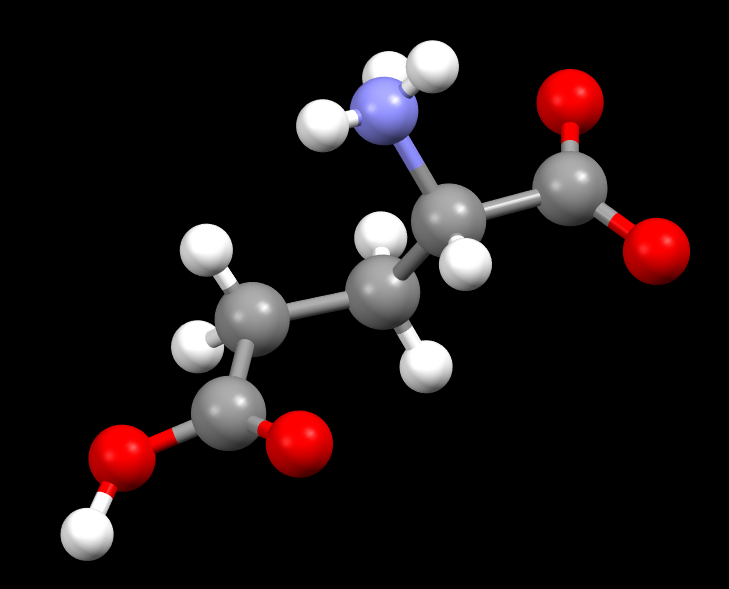](CIF/Amino_acids/LGLUAC01.cif) |
| L-Serin | [LSERIN01](CIF/Amino_acids/LSERIN01.cif) | [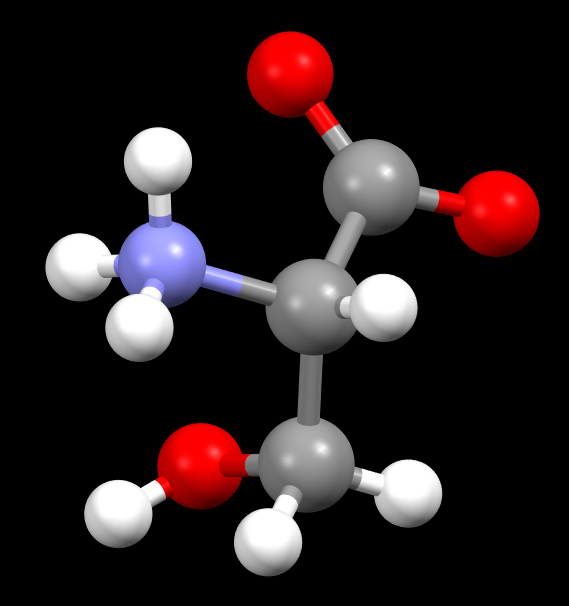](CIF/Amino_acids/LSERIN01.cif) |
| L-Threonie | [LTHREO01](CIF/Amino_acids/LTHREO01.cif) | [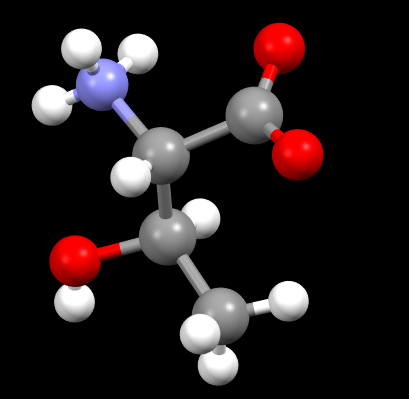](CIF/Amino_acids/LTHREO01.cif) |
| L-Tyrosin | [LTYROS10](CIF/Amino_acids/LTYROS10.cif) | [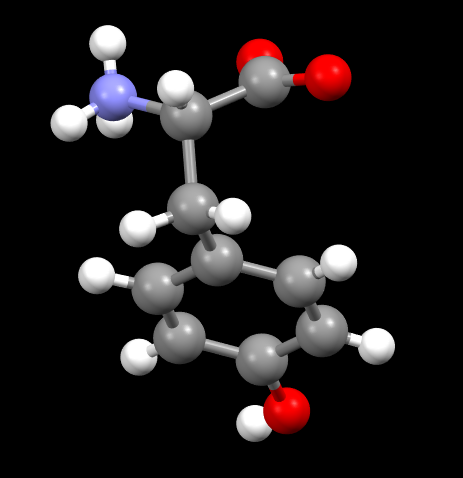](CIF/Amino_acids/LTYROS10.cif) |
| L-Cystin | [LCYSTI10](CIF/Amino_acids/LCYSTI10.cif) | [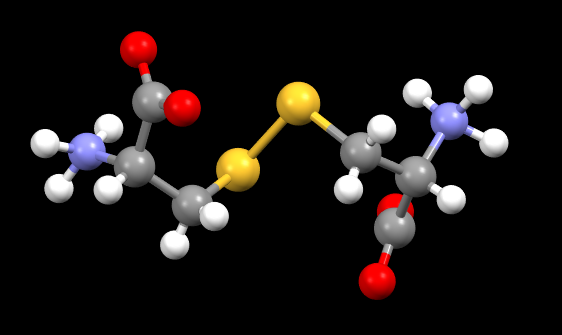](CIF/Amino_acids/LCYSTI10.cif) |
| L-Cystein | [LCYSTN22](CIF/Amino_acids/LCYSTN22.cif) | [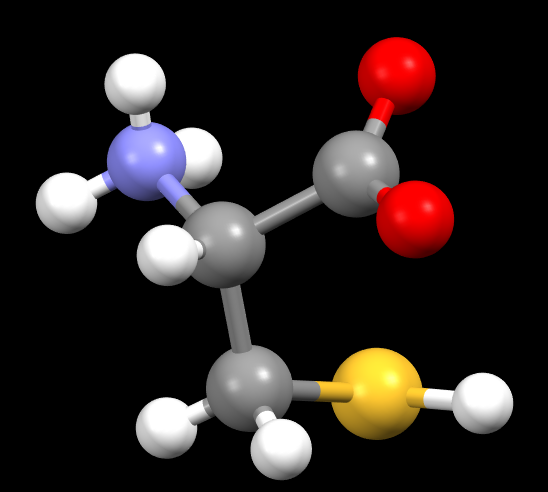](CIF/Amino_acids/LCYSTN22.cif) |
| DL-Methionin | [DLMETA05](CIF/Amino_acids/DLMETA05.cif) | [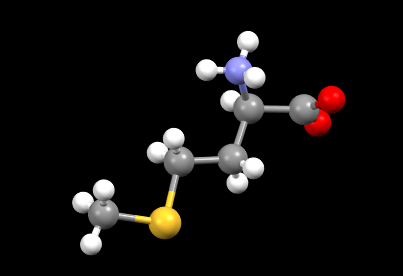](CIF/Amino_acids/DLMETA05.cif) |
| L-Glutamin | [GLUTAM01](CIF/Amino_acids/GLUTAM01.cif) | [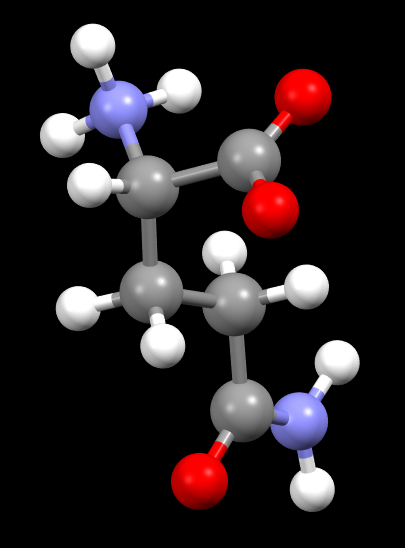](CIF/Amino_acids/GLUTAM01.cif) |
| L-Asparagin Monohydrat | [ASPARM08](CIF/Amino_acids/ASPARM08.cif) | [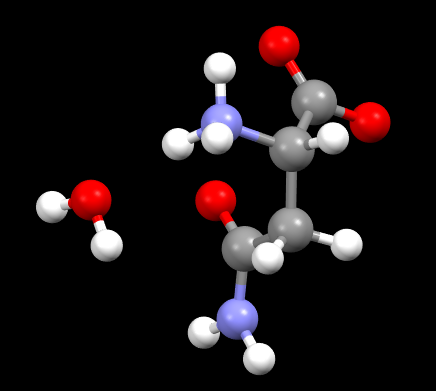](CIF/Amino_acids/ASPARM08.cif) |
| DL-Arginin Dihydrat | [WIJNEI](CIF/Amino_acids/WIJNEI.cif) | [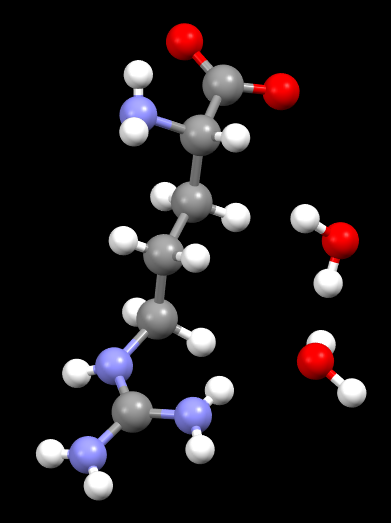](CIF/Amino_acids/WIJNEI.cif) |

# Peptide

| **Name** | **Refcode** | **Mercury** |
| --- | --- | --- |
| L-Alanyl-L-Alanin | [ALALHC](CIF/Peptides/ALALHC.cif) | [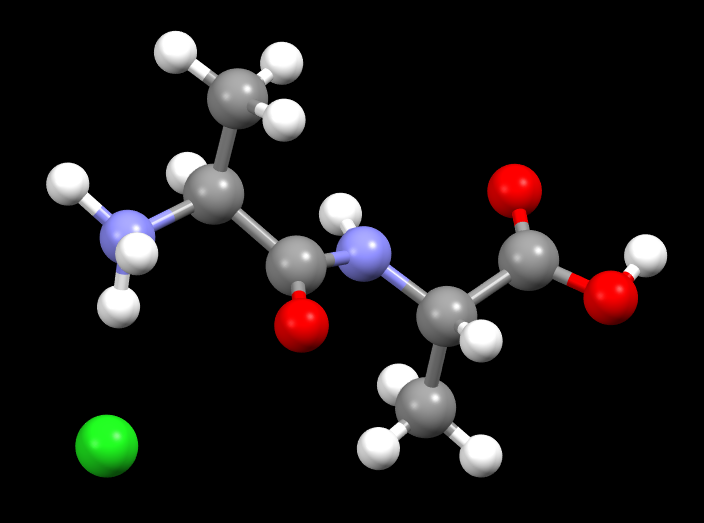](CIF/Peptides/ALALHC.cif) |

# Kohlenhydrate

| **Name** | **Refcode** | **Mercury** |
| --- | --- | --- |
| alpha-D-Glucose | [GLUCSA](CIF/Carbohydrates/GLUCSA.cif) | [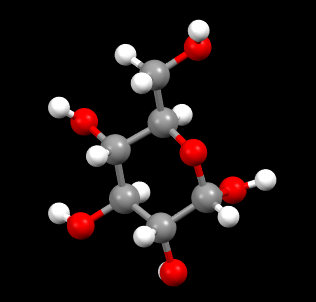](CIF/Carbohydrates/GLUCSA.cif) |
| beta-D-Glucose | [GLUCSE02](CIF/Carbohydrates/GLUCSE02.cif) | [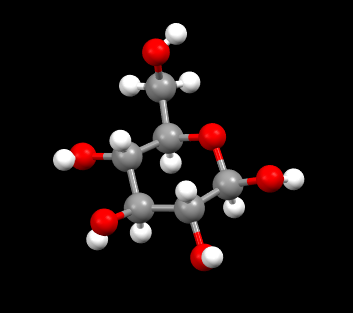](CIF/Carbohydrates/GLUCSE02.cif) |
| beta-D-Fructose | [FRUCTO11](CIF/Carbohydrates/FRUCTO11.cif) | [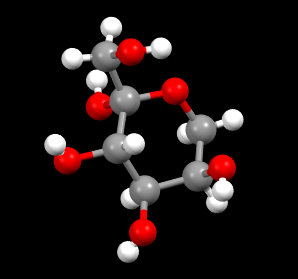](CIF/Carbohydrates/FRUCTO11.cif) |
| Saccharose | [SUCROS01](CIF/Carbohydrates/SUCROS01.cif) | [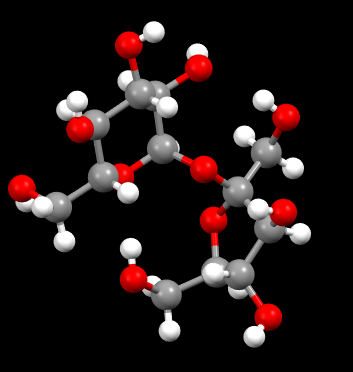](CIF/Carbohydrates/SUCROS01.cif) |
| beta-L-Arabinose | [ABINOS](CIF/Carbohydrates/ABINOS.cif) | [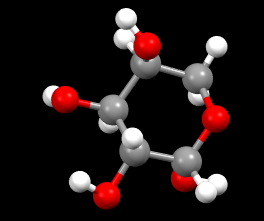](CIF/Carbohydrates/ABINOS.cif) |

# Vitamine

| **Name** | **Refcode** | **Mercury** |
| --- | --- | --- |
| Vitamin A säure | [VITAAC01](CIF/Vitamins/VITAAC01.cif) | [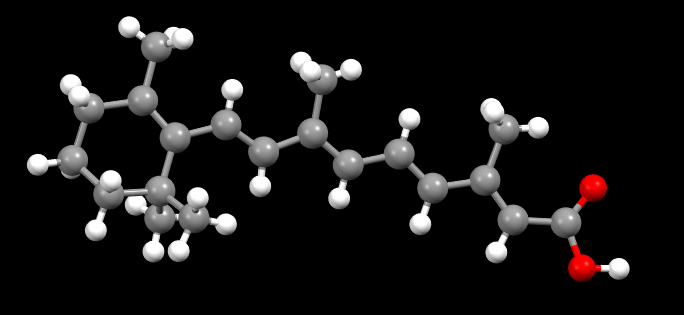](CIF/Vitamins/VITAAC01.cif) |
| Vitamin C | [LASCAC02](CIF/Vitamins/LASCAC02.cif) | [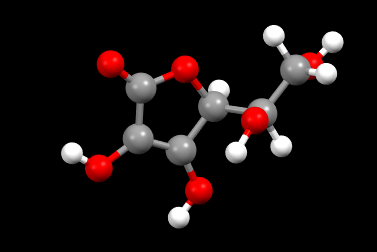](CIF/Vitamins/LASCAC02.cif) |

# Naturstoffe

| **Name** | **Refcode** | **Mercury** |
| --- | --- | --- |
| (-)Adrenalin | [ADRENL](CIF/Natural_products/ADRENL.cif) | [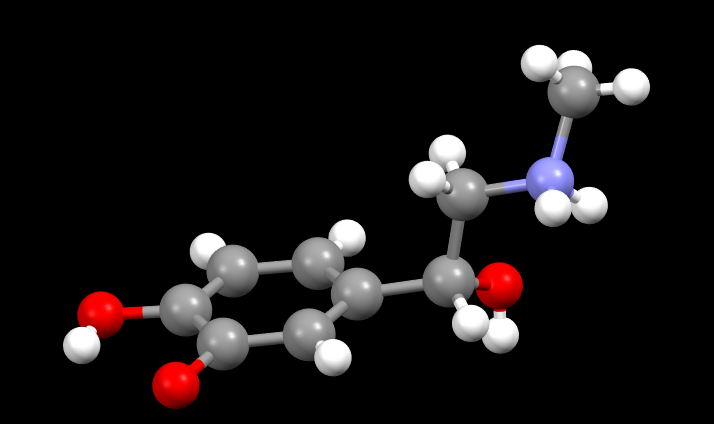](CIF/Natural_products/ADRENL.cif) |
| Adenosin | [ADENOS10](CIF/Natural_products/ADENOS10.cif) |  |
| Aspirin, Acetylsalicylsäure | [ACSALA01](CIF/Natural_products/ACSALA01.cif) |  |
| Coffein Monohydrat | [CAFINE](CIF/Natural_products/CAFINE.cif) |  |
| beta-Carotin | [CARTEN02](CIF/Natural_products/CARTEN02.cif) |  |

# Polymere

| **Name** | **Refcode** | **Mercury** |
| --- | --- | --- |
| Poly(ethen) | [QUILHUO01](CIF/Polymers/QILHUO01.cif) |  |
| Poly(propen) | [SUSJIZ](CIF/Polymers/SUSJIZ.cif) |  |
| Poly(1-buten) | [LEJKIU](CIF/Polymers/LEJKIU.cif) |  |
| Poly(styrol) | [SUSKOG](CIF/Polymers/SUSKOG.cif) |  |

## Alphabetisches Inhaltsverzeichnis

(16)Annulen 7

(18)Annulen 7

1,3,5-Trinitrobenzol 6

2,4,6-Trinitrotoluol 6

2-Amino-5-nitrophenol 6

2-Hydroxyphenol 6

5-Brom-1,3-dichlor-2-iod-benzol 7

Aceton 10

Acetylen 5

Acetylsalicylsäure 16

Adenosin 16

Adipinsäure 11

Adrenalin 16

Alanin 12, 13

alpha-D-Glucose 15

Ameisensäure 11

Anilin 9

Annulen 7

Anthrachinon 7, 10

Arabinose 15

Arginin 14

Asparagin Monohydrat 14

Aspirin 16

Benzoesäure 11

Benzol 5

Benzonitril 6

Benzophenon 10

beta-Carotin 16

beta-D-Fructose 15

beta-D-Glucose 15

beta-L-Arabinose 15

Brommethan 8

But-2-in 5

Butan 3

Butan-2-ol 9

Calciumformiat 12

Carotin 16

Catecholin 6

Chlormethan 7

Citronensäure 11

Citronensäure Monohydrat 11

Coffein Monohydrat 16

Cyanoacetlyen 5

Cyclobutan 3

Cyclohexan 4

Cyclohexen 4

Cyclo-octatetraen 4

Cyclopropan 3

Cystein 14

Cystin 14

D-Alanin 12

Decadien 4

Diaminoethan 9

Dibromhexafluorpropan 8

Dichlormethan 8

Diiodmethan 7

DL-Arginin Dihydrat 14

DL-Methionin 14

DL-Valin 13

Essigsäure 11

Ethan 3

Ethanol 8

Ethansäure 11

Ethen 4

Ethin 5

Ethylpropionat 12

Formaldehyd 10

Fructose 15

Fumarsäure 11

Glucose 15

Glutamin 14

Glutaminsäure 13

Glycin 12

Harnstoff 9

Heptan 3

Hexaaminobenzol 6

Hexan 3

Hydroxyphenol 6

Iodmethan 8

Isopropanol 9

L-(+)-Milchsäure 11

L-Alanin 13

L-Alanyl-L-Alanin 15

L-Asparagin Monohydrat 14

L-Cystein 14

L-Cystin 14

L-Glutamin 14

L-Glutaminsäure 13

L-Prolin 13

L-Serie 13

L-Threonie 13

L-Tyrosin 13

Methanol 8

Methansäure 11

Methionin 14

Methylacetat 12

Methylamin 9

Monofluoressigsäure 12

Naphthol 7

Natriumacetat 12

Octa-2,4,6-triin 5

Octadien 4

Octadiin 5

Octan 3

Pentan 3

Phenol 6

Poly(1-buten) 17

Poly(ethen) 17

Poly(propen) 17

Poly(styrol) 17

Prolin 13

Prop-2-en-säure 11

Propan 3

Propan-1-ol 9

Saccharose 15

Serie 13

Tetraiodmethan 8

Tetramethylethen 4

Threonie 13

Toluol 5

Trimethylamin 10

Trinitrobenzol 6

Trinitrotoluol 6

Tyrosin 13

Valin 13

Vitamin A säure 16

Vitamin C 16
