# Supplementary material for: Crystallography in school
Source: J Appl Crystallogr. 2025 Sep 12;58(Pt 5):1802–9. doi: 10.1107/S1600576725007459 (PMC12502877; doi:10.1107/S1600576725007459)
Supplement: Supplementary file 3 [file j-58-01802-sup3.zip › Teaching Subset nach Stoffklassen.pdf]

Der CSD Teaching Subset nach Stoffklassen sortiert

Eine Auswahl von schulrelevanten Strukturen

von Erhard Irmer (XLAB Göttingen, Germany)

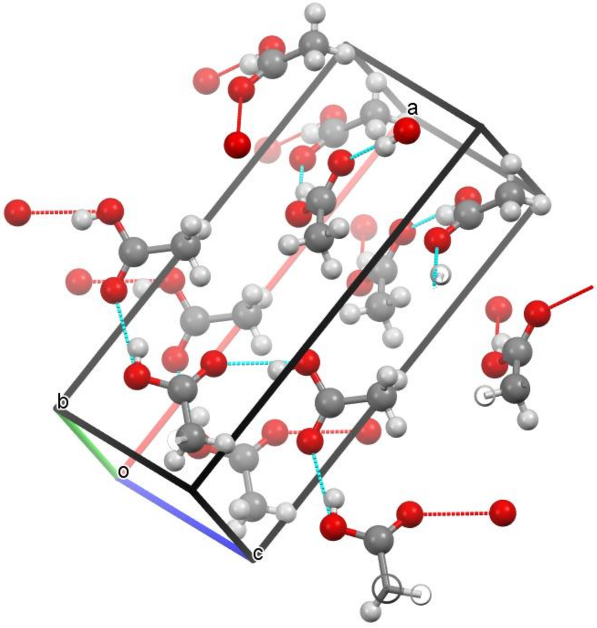

Inhalt

Inhalt 2
1. Alkane 3
2. Alkene 4
3. Alkine 5
4. Aromaten 5
5. Halogenkohlenwasserstoffe 7
6. Alkanole 8
7. Amine 9
8. Aldehyde 10
9. Ketone 10
10. Carbonsäuren 11
11. Ester 12
12. Aminosäuren 12
13. Peptide 15
14. Kohlenhydrate 15
15. Vitamine 16
16. Naturstoffe 16
17. Polymere 17
Alphabetisches Inhaltsverzeichnis 18

1. Alkane

Name	Refcode	Mercury
Ethan	ETHANE01	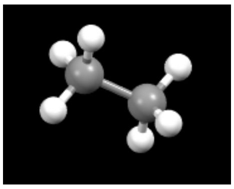
Propan	JAYDUI	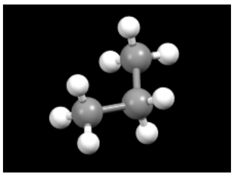
n-Butan	DUCKOB04	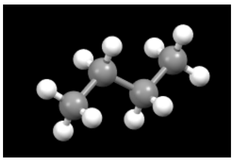
n-Pentan	PENTAN01	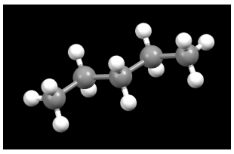
n-Hexan	HEXANE01	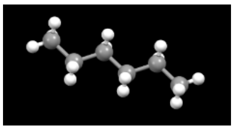
n-Heptan	HEPTAN03	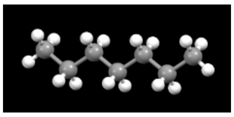
n-Octan	OCTANE12	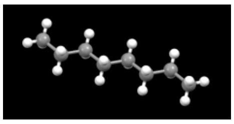
Cyclopropan	QQQCIS01	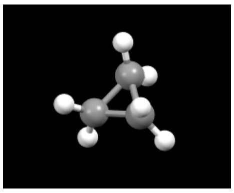
Cyclobutan	ZZZWEO02	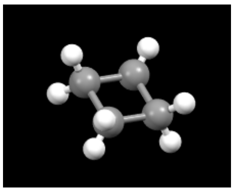

Cyclohexan	CYCHEX	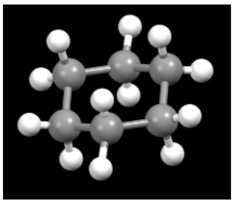

2. Alkene

Name	Refcode	Mercury
Ethen	ETHLEN10	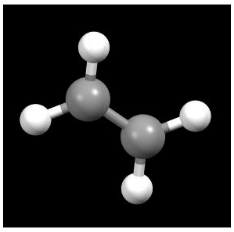
Tetramethylethen	PAPVAD	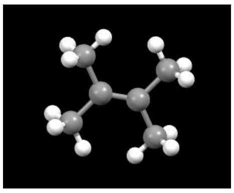
1,7-Octadien	XOMHUC	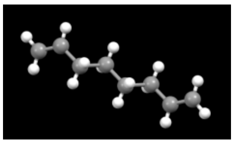
1,9-Decadien	XOMJAK	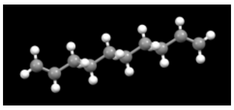
Cyclohexen	COVJON	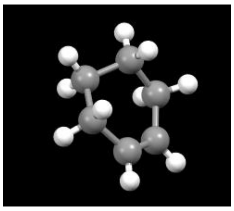
Cyclo-octatetraen	ZZZSAE01	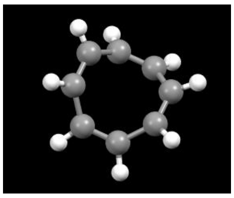

3. Alkine

**Name**	**Refcode**	**Mercury**
Ethin, Acetylen	ACETYL03	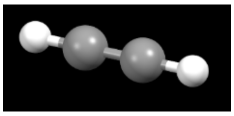
But-2-in Hydrogenchlorid	JUFDUJ	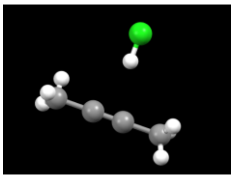
Octa-2,4,6-triin	OCTRNE	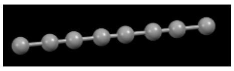
Cyanoacetlyen	CAACTY	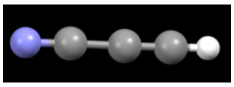
1,7-Octadiin	XOMJEO	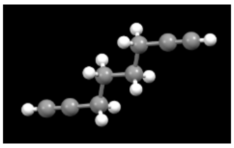

4. Aromaten

**Name**	**Refcode**	**Mercury**
Benzol (neutron diffraction)	BENZEN	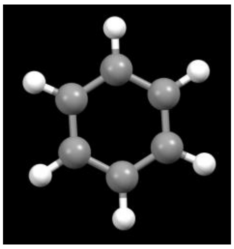
Benzol	BENZEN02	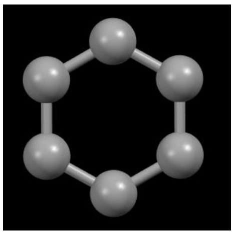
Toluol	TOLUEN	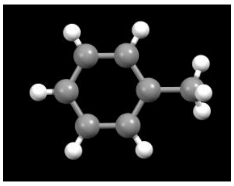

Phenol	PHENOL03	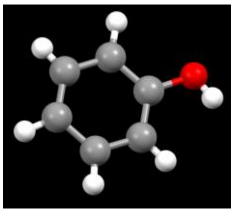
Catecholin, 2-Hydroxyphenol	CATCOL13	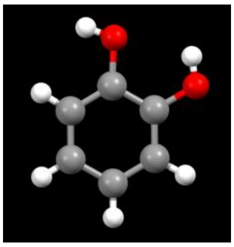
2-Amino-5-nitrophenol	AMNPHA	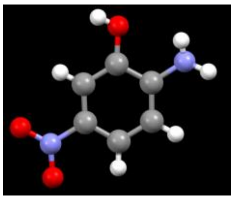
1,3,5-Trinitrobenzol	TNBENZ12	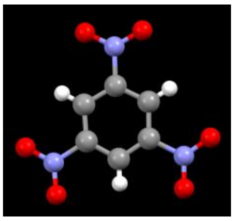
2,4,6-Trinitrotoluol	ZZZMUC01	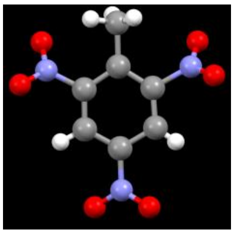
Hexaaminobenzol	ZZZWOU01	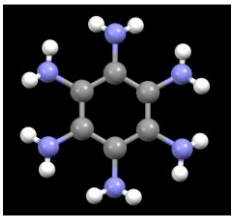
Benzonitril	BZONTR	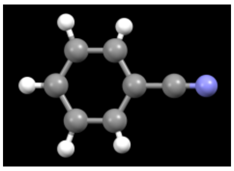

5-Brom-1,3-dichlor-2-iod-benzol	ACEPOO	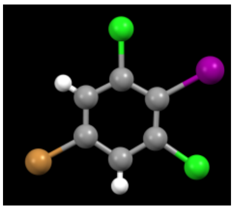
Naphthol	NAPHTA12	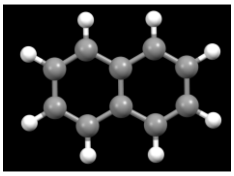
Anthrachinon	ANTQUO08	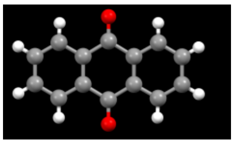
(16)Annulen	ANNULE01	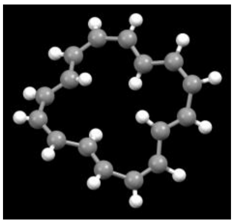
(18)Annulen	ANULEN	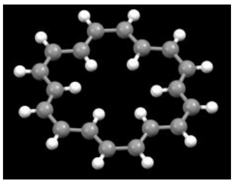

5. Halogenkohlenwasserstoffe

**Name**	**Refcode**	**Mercury**
Chlormethan	CLMETH	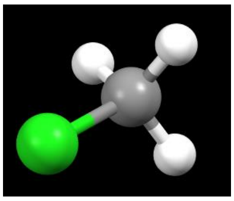
Diiodmethan	DIMETH03	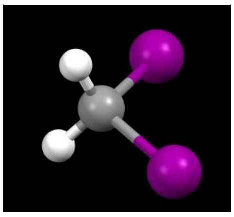

Brommethan	MBRMET10	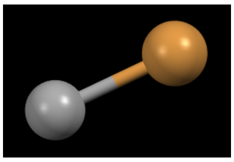
Iodmethan	MIMETH10	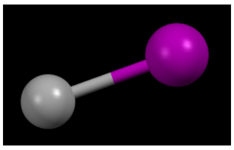
Tetraiodmethan	ZZZKDW01	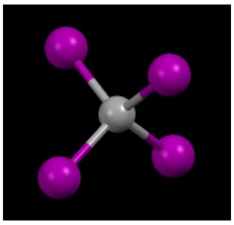
Dichlormethan (solvent)	BEJKUW	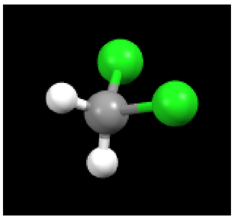
Dibromhexafluorpropan	BOCGAB	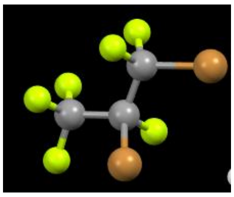

6. Alkanole

**Name**	**Refcode**	**Mercury**
Methanol	METHOL	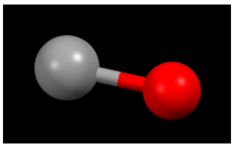
Ethanol	ETANOL	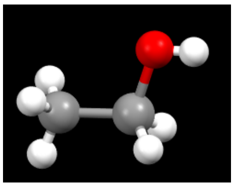

Propan-1-ol (solvent)	VENVAM	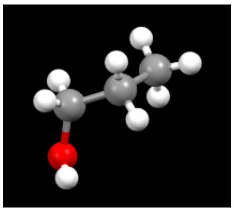
Isopropanol (solvent)	ABALEV	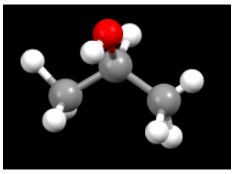
Butan-2-ol (solvent)	AVEPIB	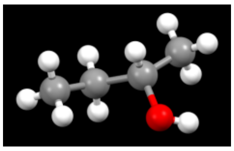

7. Amine

**Name**	**Refcode**	**Mercury**
1,2-Diaminoethan	ETDIAM12	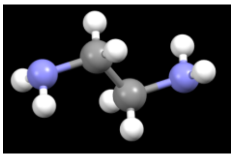
Methylamin	METAMI	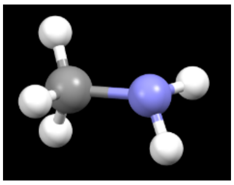
Anilin	BAZGOY	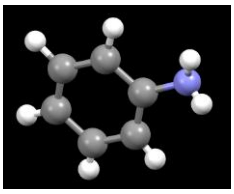
Harnstoff	UREAXX	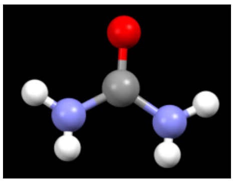

Trimethylamin	CEKGUU01	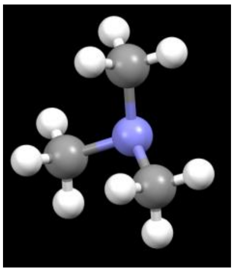

8. Aldehyde

**Name**	**Refcode**	**Mercury**
Formaldehyd	GURNEN	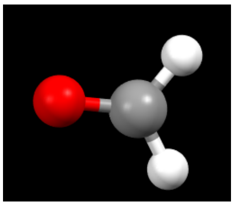

9. Ketone

**Name**	**Refcode**	**Mercury**
Aceton	HIXHIF05	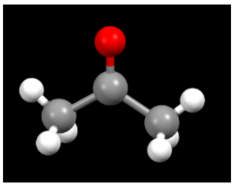
Anthrachinon	ANTQUO08	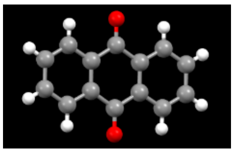
Benzophenon	BPHENO03	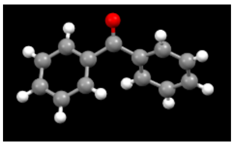

10. Carbonsäuren

**Name**	**Refcode**	**Mercury**
Ameisensäure, Methansäure	FORMAC01	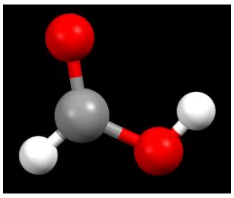
Essigsäure, Ethansäure	ACETAC07	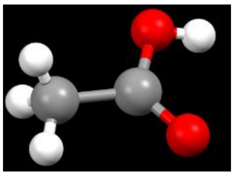
Benzoesäure	BENZAC02	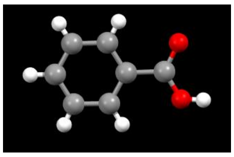
Prop-2-en-säure	ACRLAC02	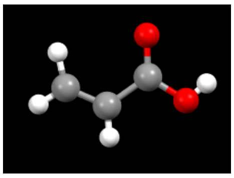
Adipinsäure	ADIPAC04	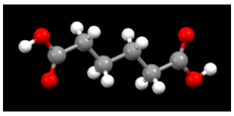
L-(+)-Milch	YILLAG	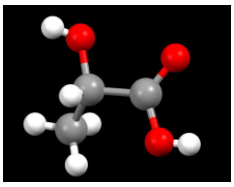
Citronensäure	CITRAC10	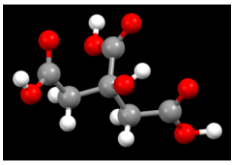
Citronensäure Monohydrat	CITARC	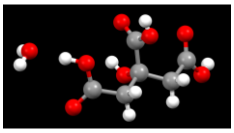
Fumarsäure	FUMAAC01	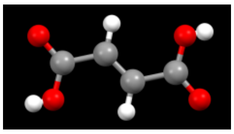

Monofluoressigsäure	FACETC10	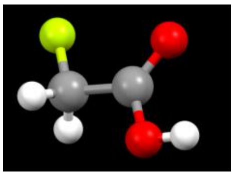
Natriumacetat	BOPKOG10	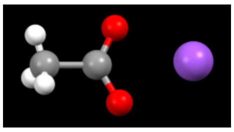
Calciumformiat	CAFORM05	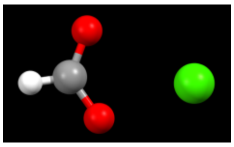

11. Ester

**Name**	**Refcode**	**Mercury**
Methylacetat	BAHSUY	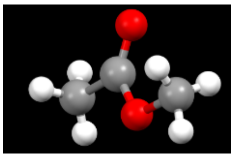
Ethylpropionat	YARZUN03	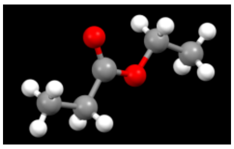

12. Aminosäuren

**Name**	**Refcode**	**Mercury**
Glycin	GLYCIN	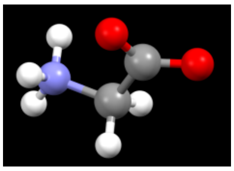
D-Alanin	ALUCAL05	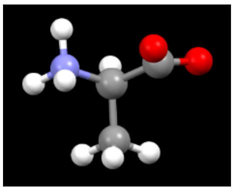

L-Alanin	LALNIN23	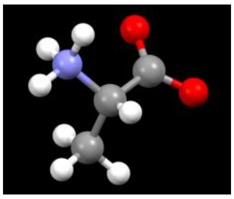
DL-Valin	VALIDL	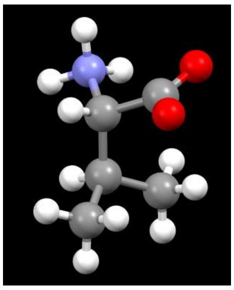
L-Prolin	PROLIN	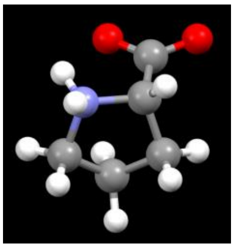
L-Glutaminsäure	LGLUAC01	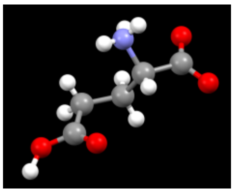
L-Serin	LSERIN01	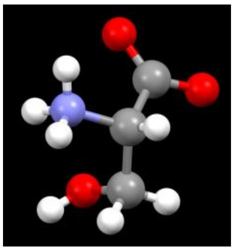
L-Threonie	LTHREO01	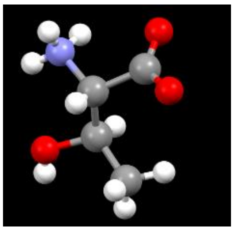
L-Tyrosin	LTYROS10	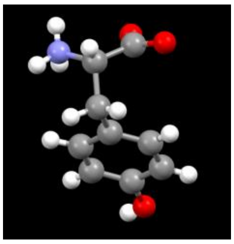

L-Cystin	LCYSTI10	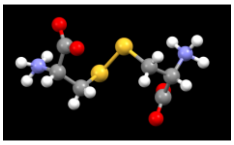
L-Cystein	LCYSTN22	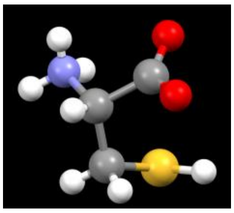
DL-Methionin	DLMETA05	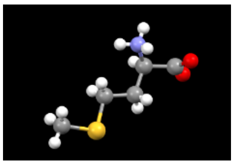
L-Glutamin	GLUTAM01	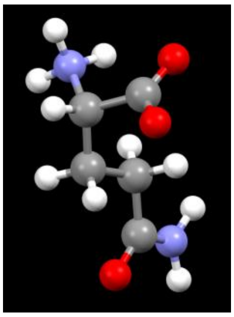
L-Asparagin Monohydrat	ASPARM08	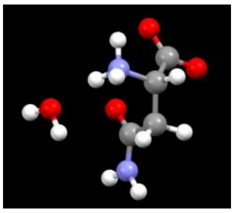
DL-Arginin Dihydrat	WIJNEI	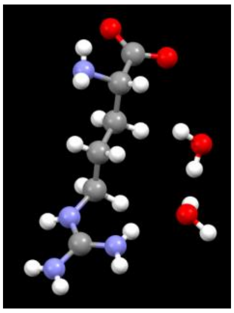

13. Peptide

Name	Refcode	Mercury
L-Alanyl-L-Alanin	ALALHC	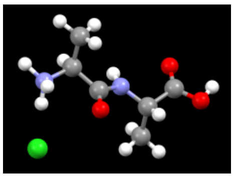

14. Kohlenhydrate

Name	Refcode	Mercury
alpha-D-Glucose	GLUCSA	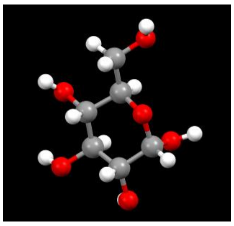
beta-D-Glucose	GLUCSE02	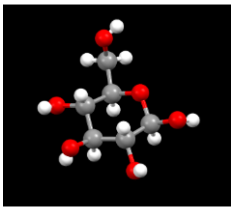
beta-D-Fructose	FRUCTO11	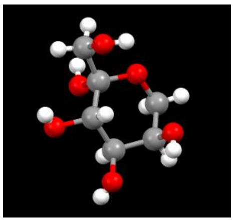
Saccharose	SUCROS01	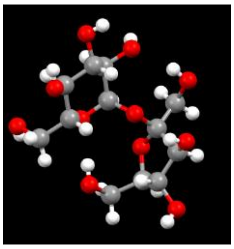
beta-L-Arabinose	ABINOS	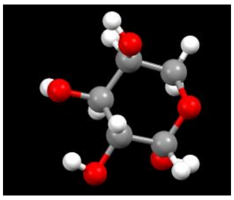

15. Vitamine

Name	Refcode	Mercury
Vitamin A säure	VITAAC01	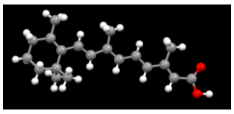
Vitamin C	LASCAC02	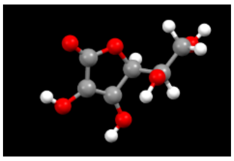

16. Naturstoffe

Name	Refcode	Mercury
(-)Adrenalin	ADRENL	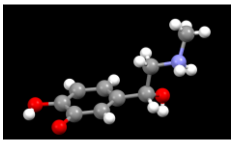
Adenosin	ADENOS10	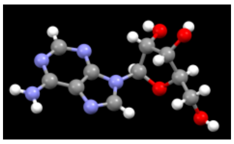
Aspirin, Acetylsalicylsäure	ACSALA01	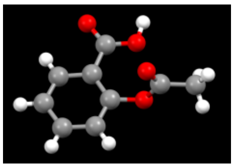
Coffein Monohydrat	CAFINE	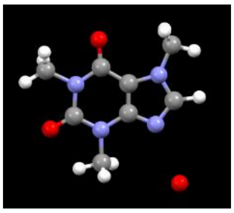
beta-Carotin	CARTEN02	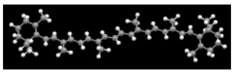

17. Polymere

Name	Refcode	Mercury
Poly(ethen)	QUILHUO01	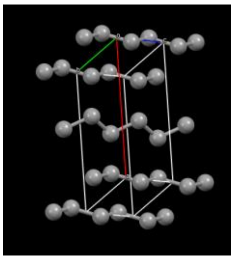
Poly(propen)	SUSJIZ	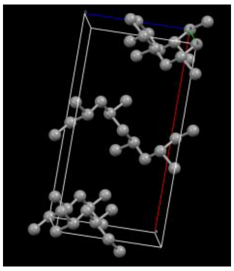
Poly(1-buten)	LEJKIU	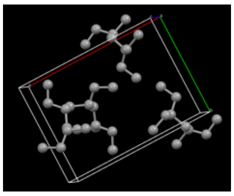
Poly(styrol)	SUSKOG	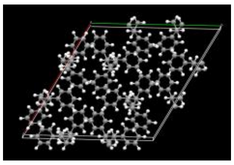

Alphabetisches Inhaltsverzeichnis

(16)Annulen....7
(18)Annulen....7
1,3,5-Trinitrobenzol....6
2,4,6-Trinitrotoluol....6
2-Amino-5-nitrophenol....6
2-Hydroxyphenol....6
5-Brom-1,3-dichlor-2-iod-benzol....7
Aceton....10
Acetylen....5
Acetylsalicylsäure....16
Adenosin....16
Adipinsäure....11
Adrenalin....16
Alanin....12, 13
alpha-D-Glucose....15
Ameisensäure....11
Anilin....9
Annulen....7
Anthrachinon....7, 10
Arabinose....15
Arginin....14
Asparagin Monohydrat....14
Aspirin....16
Benzoesäure....11
Benzol....5
Benzonitril....6
Benzophenon....10
beta-Carotin....16
beta-D-Fructose....15
beta-D-Glucose....15
beta-L-Arabinose....15
Brommethan....8
But-2-in....5
Butan....3
Butan-2-ol....9
Calciumformiat....12
Carotin....16
Catecholin....6
Chlormethan....7
Citronensäure....11
Citronensäure Monohydrat....11
Coffein Monohydrat....16
Cyanoacetlyen....5
Cyclobutan....3
Cyclohexan....4
Cyclohexen....4
Cyclo-octatetraen....4
Cyclopropan....3
Cystein....14
Cystin....14
D-Alanin....12
Decadien....4
Diaminoethan....9
Dibromhexafluorpropan....8
Dichlormethan....8
Diiodmethan....7
DL-Arginin Dihydrat....14
DL-Methionin....14
DL-Valin....13
Essigsäure....11
Ethan....3
Ethanol....8
Ethansäure....11
Ethen....4
Ethin....5
Ethylpropionat....12
Formaldehyd....10
Fructose....15
Fumarsäure....11
Glucose....15
Glutamin....14
Glutaminsäure....13
Glycin....12
Harnstoff....9
Heptan....3
Hexaaminobenzol....6
Hexan....3
Hydroxyphenol....6
Iodmethan....8
Isopropanol....9
L-(+)-Milchsäure....11
L-Alanin....13
L-Alanyl-L-Alanin....15
L-Asparagin Monohydrat....14
L-Cystein....14
L-Cystin....14
L-Glutamin....14
L-Glutaminsäure....13
L-Prolin....13
L-Serie....13
L-Threonie....13
L-Tyrosin....13
Methanol....8
Methansäure....11
Methionin....14
Methylacetat....12

Methylamin 9
Monofluoressigsäure....................................12
Naphthol...7
Natriumacetat 12
Octa-2,4,6-triin 5
Octadien 4
Octadiin 5
Octan 3
Pentan 3
Phenol..6
Poly(1-buten)..17
Poly(ethen) 17
Poly(propen)...17
Poly(styrol) 17
Prolin 13
Prop-2-en-säure..11
Propan... 3
Propan-1-ol 9
Saccharose 15
Serie 13
Tetraiodmethan 8
Tetramethylethen.. 4
Threonie.. 13
Toluol 5
Trimethylamin.. 10
Trinitrobenzol 6
Trinitrotoluol.. 6
Tyrosin 13
Valin 13
Vitamin A säure... 16
Vitamin C... 16
