# Supplementary material for: Crystallography in school
Source: J Appl Crystallogr. 2025 Sep 12;58(Pt 5):1802–9. doi: 10.1107/S1600576725007459 (PMC12502877; doi:10.1107/S1600576725007459)
Supplement: Supplementary file 3 [file j-58-01802-sup3.zip › Teaching Subset substance classes.docx]

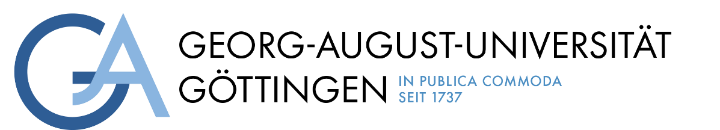

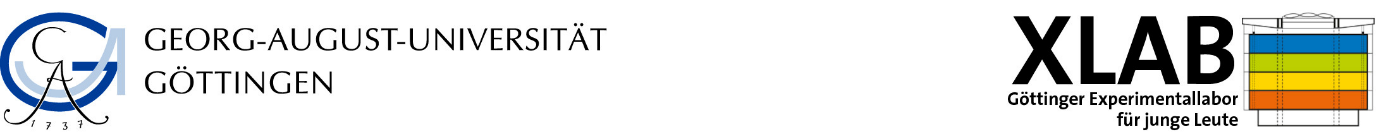


**The CSD Teaching Subset
sorted by substance classes**

**A selection of school-relevant organic structures**

by Erhard Irmer (XLAB Göttingen, Germany)

**
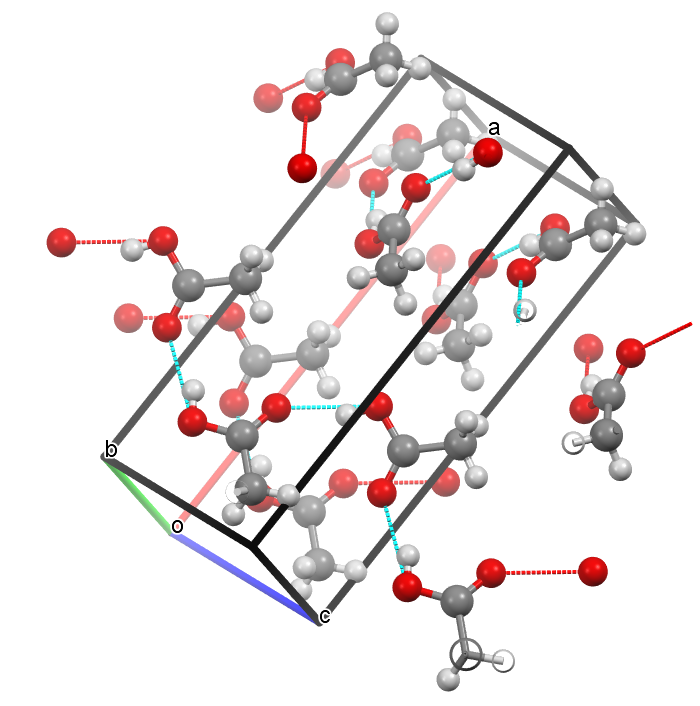
**


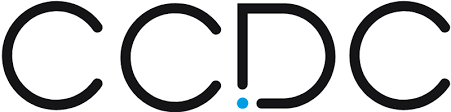

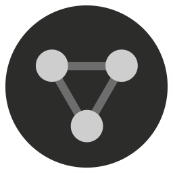


# Contents

[Contents 2](#_Toc142916883)

[1. Alkanes 3](#_Toc142916884)

[2. Alkenes 4](#_Toc142916885)

[3. Alkynes 5](#_Toc142916886)

[4. Aromatics 5](#_Toc142916887)

[5. Alkyl halides 7](#_Toc142916888)

[6. Alkanols 8](#_Toc142916889)

[7. Amines 9](#_Toc142916890)

[8. Aldehydes 10](#_Toc142916891)

[9. Ketones 10](#_Toc142916892)

[10. Carboxylic acids 11](#_Toc142916893)

[11. Esters 12](#_Toc142916894)

[12. Amino acids 12](#_Toc142916895)

[13. Peptides 15](#_Toc142916896)

[14. Carbohydrates 15](#_Toc142916897)

[15. Vitamins 16](#_Toc142916898)

[16. Natural products 16](#_Toc142916899)

[17. Polymers 17](#_Toc142916900)

[Alphabetical Index 18](#_Toc142916901)

# Alkanes

| **Name** | **WebCSD** | **Mercury** |
| --- | --- | --- |
| Ethane | [ETHANE01](https://www.ccdc.cam.ac.uk/structures/Search?Ccdcid=ethane01&DatabaseToSearch=Published) | [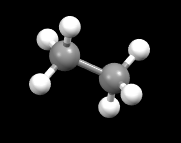](CIF/Alkanes/ETHANE01.cif) |
| Propane | [JAYDUI](https://www.ccdc.cam.ac.uk/structures/Search?Ccdcid=jaydui&DatabaseToSearch=Published) | [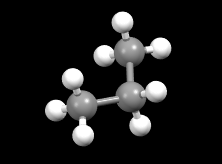](CIF/Alkanes/JAYDUI.cif) |
| n-Butane | [DUCKOB04](https://www.ccdc.cam.ac.uk/structures/Search?Ccdcid=DUCKOB04&DatabaseToSearch=Published) | [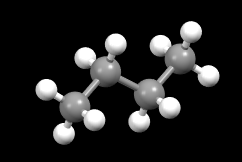](CIF/Alkanes/DUCKOB04.cif) |
| n-Pentane | [PENTAN01](https://www.ccdc.cam.ac.uk/structures/Search?Ccdcid=PENTAN01&DatabaseToSearch=Published) | [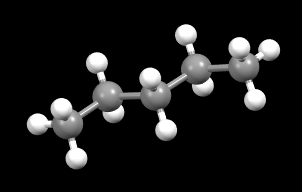](CIF/Alkanes/PENTAN01.cif) |
| n-Hexane | [HEXANE01](https://www.ccdc.cam.ac.uk/structures/Search?Ccdcid=HEXANE01&DatabaseToSearch=Published) | [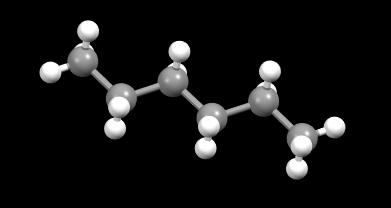](CIF/Alkanes/HEXANE01.cif) |
| n-Heptane | [HEPTAN03](https://www.ccdc.cam.ac.uk/structures/Search?Ccdcid=HEPTAN03&DatabaseToSearch=Published) | [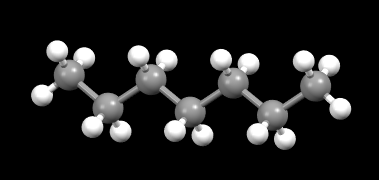](CIF/Alkanes/HEPTAN03.cif) |
| n-Octane | [OCTANE12](https://www.ccdc.cam.ac.uk/structures/Search?Ccdcid=OCTANE12&DatabaseToSearch=Published) | [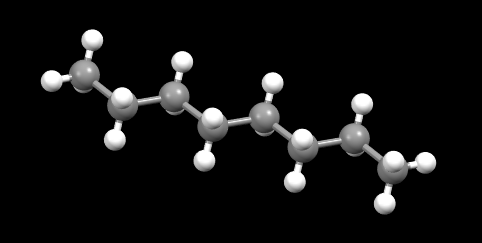](CIF/Alkanes/OCTANE12.cif) |
| Cyclopropane | [QQQCIS01](https://www.ccdc.cam.ac.uk/structures/Search?Ccdcid=QQQCIS01&DatabaseToSearch=Published) | [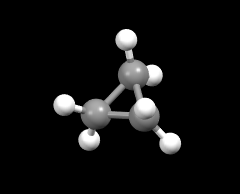](CIF/Alkanes/QQQCIS01.cif) |
| Cyclobutane | [ZZZWEO02](https://www.ccdc.cam.ac.uk/structures/Search?Ccdcid=ZZZWEO02&DatabaseToSearch=Published) | [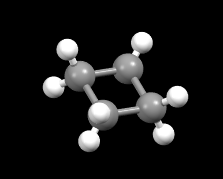](CIF/Alkanes/ZZZWEO02.cif) |
| Cyclohexane | [CYCHEX](https://www.ccdc.cam.ac.uk/structures/Search?Ccdcid=CYCHEX&DatabaseToSearch=Published) | [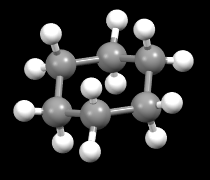](CIF/Alkanes/CYCHEX.cif) |

# Alkenes

| **Name** | **WebCSD** | **Mercury** |
| --- | --- | --- |
| Ethene | [ETHLEN10](https://www.ccdc.cam.ac.uk/structures/Search?Ccdcid=ETHLEN10&DatabaseToSearch=Published) | [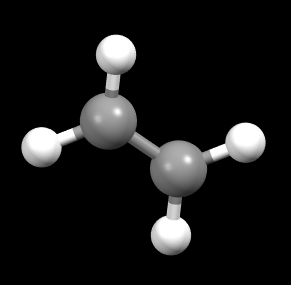](CIF/Alkenes/ETHLEN10.cif) |
| Tetramethylethene | [PAPVAD](https://www.ccdc.cam.ac.uk/structures/Search?Ccdcid=PAPVAD&DatabaseToSearch=Published) | [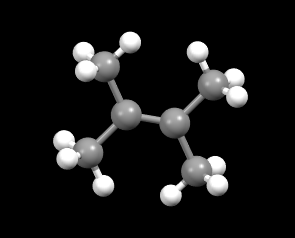](CIF/Alkenes/PAPVAD.cif) |
| 1,7-Octadiene | [XOMHUC](https://www.ccdc.cam.ac.uk/structures/Search?Ccdcid=XOMHUC&DatabaseToSearch=Published) | [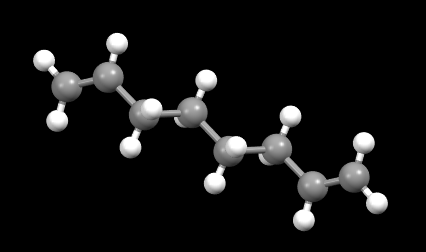](CIF/Alkenes/XOMHUC.cif) |
| 1,9-Decadiene | [XOMJAK](https://www.ccdc.cam.ac.uk/structures/Search?Ccdcid=XOMJAK&DatabaseToSearch=Published) | [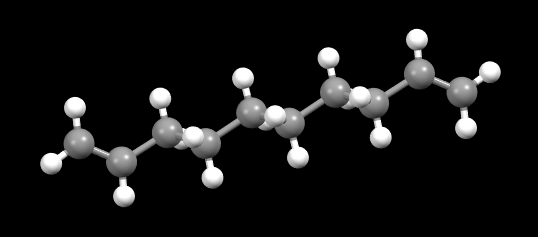](CIF/Alkenes/XOMJAK.cif) |
| Cyclohexene | [COVJON](https://www.ccdc.cam.ac.uk/structures/Search?Ccdcid=COVJON&DatabaseToSearch=Published) | [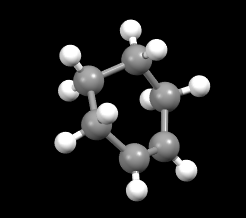](CIF/Alkenes/COVJON.cif) |
| Cyclo-octatetraene | [ZZZSAE01](https://www.ccdc.cam.ac.uk/structures/Search?Ccdcid=ZZZSAE01&DatabaseToSearch=Published) | [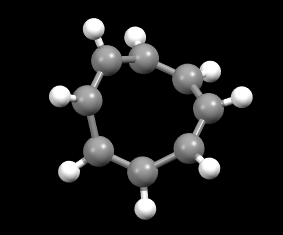](CIF/Alkenes/ZZZSAE01.cif) |

# Alkynes

| **Name** | **WebCSD** | **Mercury** |
| --- | --- | --- |
| Ethyne, Acetylene | [ACETYL03](https://www.ccdc.cam.ac.uk/structures/Search?Ccdcid=ACETYL03&DatabaseToSearch=Published) | [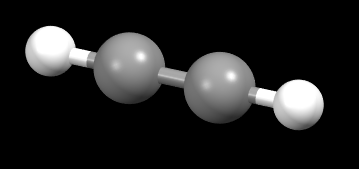](CIF/Alkynes/ACETYL03.cif) |
| But-2-in hydrogen chloride | [JUFDUJ](https://www.ccdc.cam.ac.uk/structures/Search?Ccdcid=JUFDUJ&DatabaseToSearch=Published) | [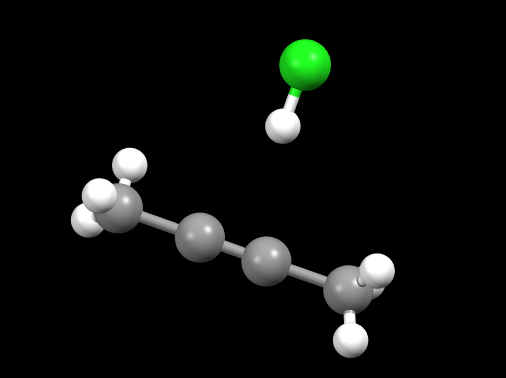](CIF/Alkynes/JUFDUJ.cif) |
| Octa-2,4,6-triyne | [OCTRNE](https://www.ccdc.cam.ac.uk/structures/Search?Ccdcid=OCTRNE&DatabaseToSearch=Published) | [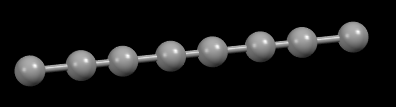](CIF/Alkynes/OCTRNE.cif) |
| Cyanoacetlyene | [CAACTY](https://www.ccdc.cam.ac.uk/structures/Search?Ccdcid=CAACTY&DatabaseToSearch=Published) | [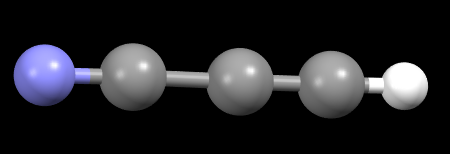](CIF/Alkynes/CAACTY.cif) |
| 1,7-Octadiin | [XOMJEO](https://www.ccdc.cam.ac.uk/structures/Search?Ccdcid=XOMJEO&DatabaseToSearch=Published) | [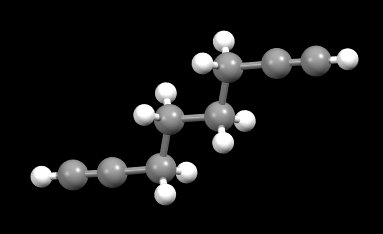](CIF/Alkynes/XOMJEO.cif) |

# Aromatics

| **Name** | **WebCSD** | **Mercury** |
| --- | --- | --- |
| Benzene (neutron diffraction) | [BENZEN](https://www.ccdc.cam.ac.uk/structures/Search?Ccdcid=BENZEN&DatabaseToSearch=Published) | [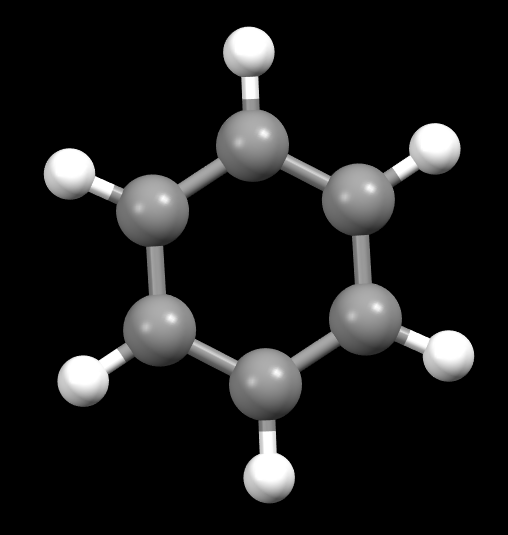](CIF/Aromatics/BENZEN.cif) |
| Benzene | [BENZEN02](https://www.ccdc.cam.ac.uk/structures/Search?Ccdcid=BENZEN02) | [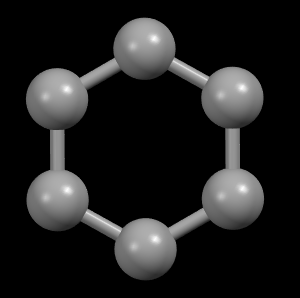](CIF/Aromatics/BENZEN02.cif) |
| Toluene | [TOLUEN](https://www.ccdc.cam.ac.uk/structures/Search?Ccdcid=TOLUEN&DatabaseToSearch=Published) | [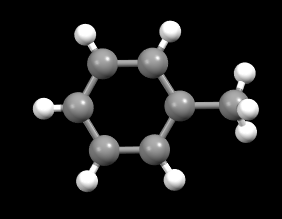](CIF/Aromatics/TOLUEN.cif) |
| Phenol | [PHENOL03](https://www.ccdc.cam.ac.uk/structures/Search?Ccdcid=PHENOL03&DatabaseToSearch=Published) | [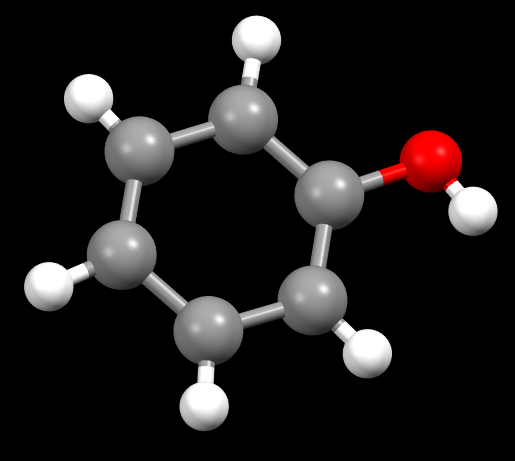](CIF/Aromatics/PHENOL03.cif) |
| Catecholine, 2-Hydroxyphenol | [CATCOL13](https://www.ccdc.cam.ac.uk/structures/Search?Ccdcid=CATCOL13&DatabaseToSearch=Published) | [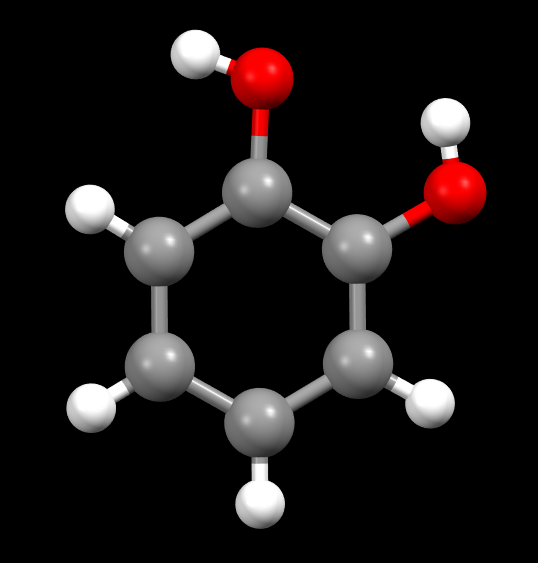](CIF/Aromatics/CATCOL13.cif) |
| 2-Amino-5-nitrophenol | [AMNPHA](https://www.ccdc.cam.ac.uk/structures/Search?Ccdcid=AMNPHA&DatabaseToSearch=Published) | [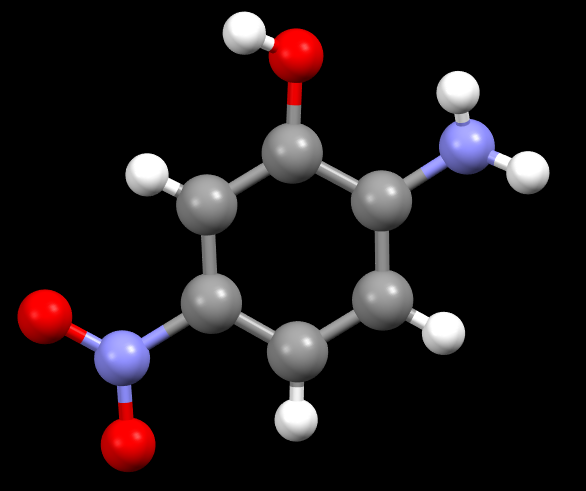](CIF/Aromatics/AMNPHA.cif) |
| 1,3,5-Trinitrobenzene | [TNBENZ12](https://www.ccdc.cam.ac.uk/structures/Search?Ccdcid=TNBENZ12&DatabaseToSearch=Published) | [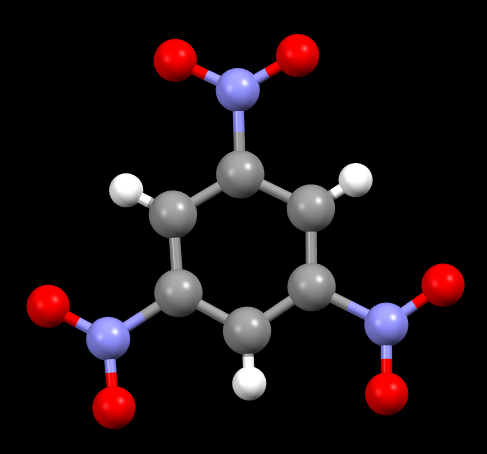](CIF/Aromatics/TNBENZ12.cif) |
| 2,4,6-Trinitrotoluene | [ZZZMUC01](https://www.ccdc.cam.ac.uk/structures/Search?Ccdcid=ZZZMUC01&DatabaseToSearch=Published) | [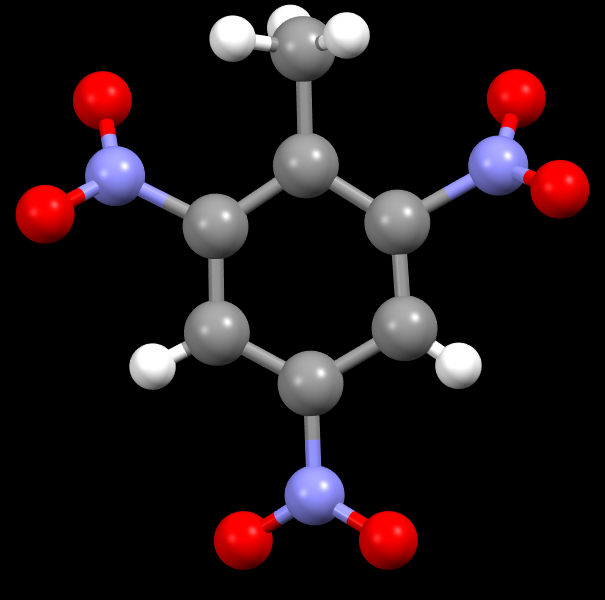](CIF/Aromatics/ZZZMUC01.cif) |
| Hexaaminobenzene | [ZZZWOU01](https://www.ccdc.cam.ac.uk/structures/Search?Ccdcid=ZZZWOU01&DatabaseToSearch=Published) | [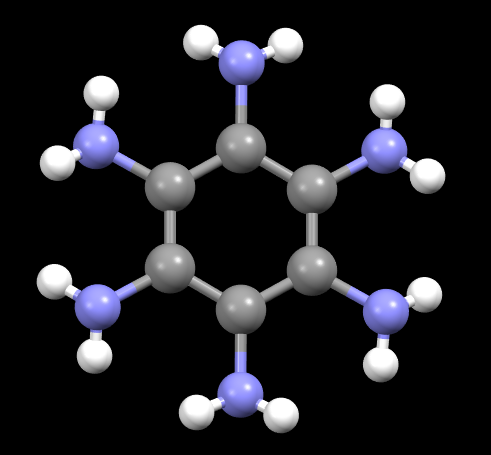](CIF/Aromatics/ZZZWOU01.cif) |
| Benzonitrile | [BZONTR](https://www.ccdc.cam.ac.uk/structures/Search?Ccdcid=BZONTR&DatabaseToSearch=Published) | [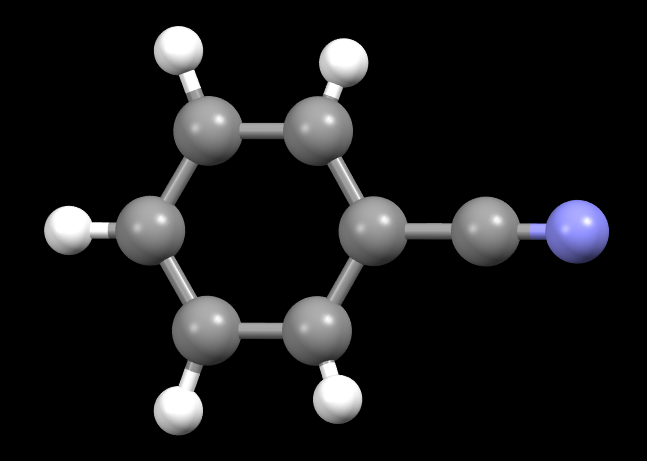](CIF/Aromatics/BZONTR.cif) |
| 5-Brom-1,3-dichlor-2-iod-benzol | [ACEPOO](https://www.ccdc.cam.ac.uk/structures/Search?Ccdcid=ACEPOO&DatabaseToSearch=Published) | [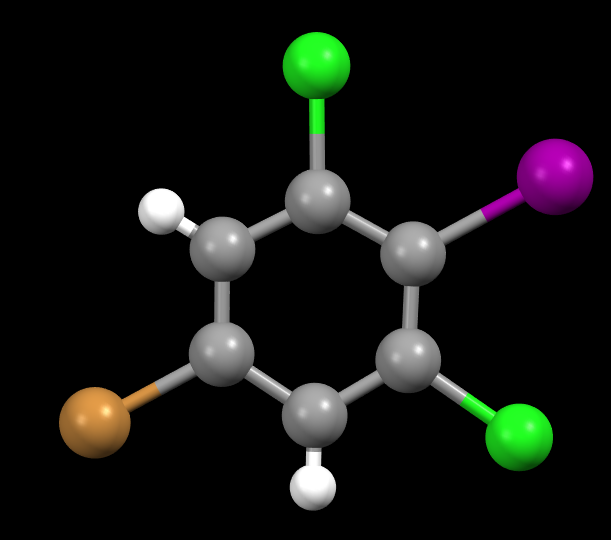](CIF/Aromatics/ACEPOO.cif) |
| Naphthalene | [NAPHTA12](https://www.ccdc.cam.ac.uk/structures/Search?Ccdcid=NAPHTA12&DatabaseToSearch=Published) | [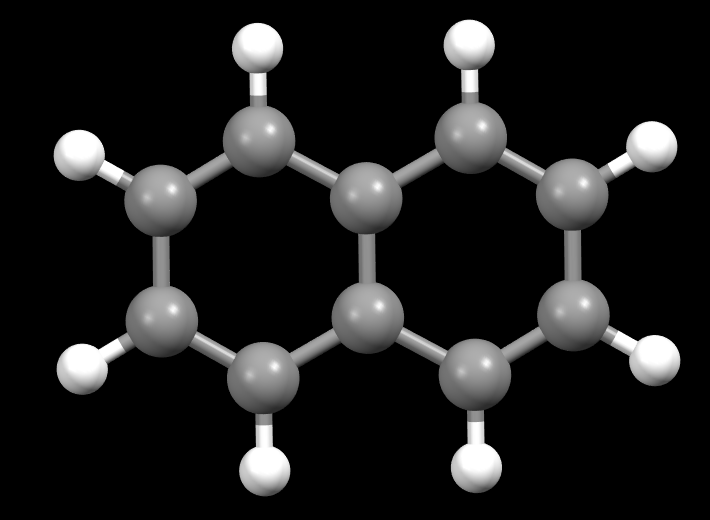](CIF/Aromatics/NAPHTA12.cif) |
| Anthraquinone | [ANTQUO08](https://www.ccdc.cam.ac.uk/structures/Search?Ccdcid=ANTQUO08&DatabaseToSearch=Published) | [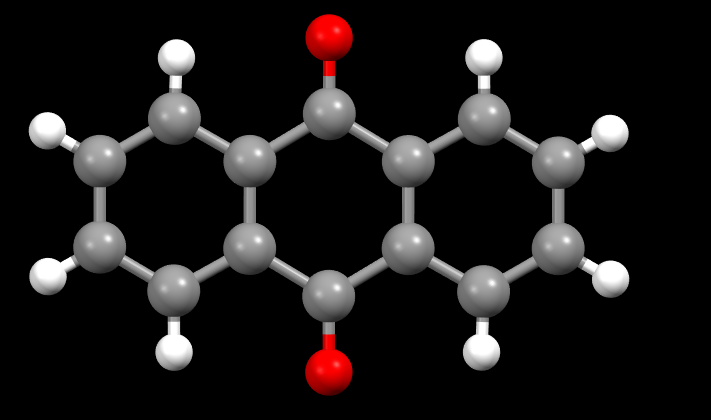](CIF/Aromatics/ANTQUO08.cif) |
| (16)Annulene | [ANNULE01](https://www.ccdc.cam.ac.uk/structures/Search?Ccdcid=ANNULE01&DatabaseToSearch=Published) | [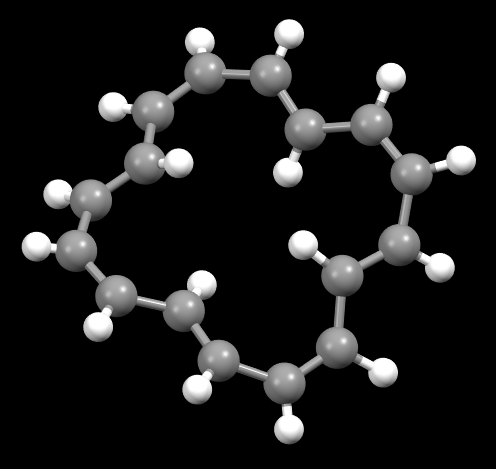](CIF/Aromatics/ANNULE01.cif) |
| (18)Annulene | [ANULEN](https://www.ccdc.cam.ac.uk/structures/Search?Ccdcid=ANULEN&DatabaseToSearch=Published) | [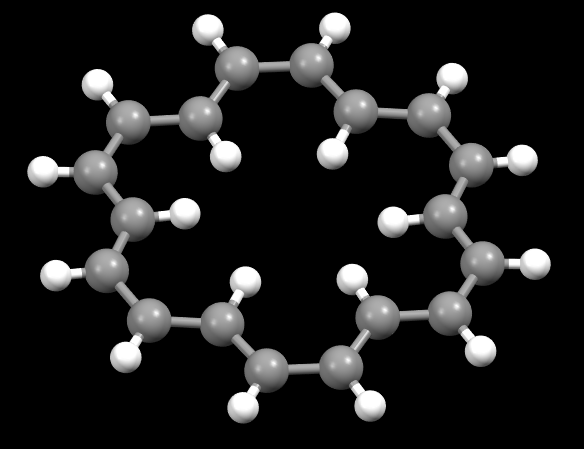](CIF/Aromatics/ANULEN.cif) |

# Alkyl halides

| **Name** | **WebCSD** | **Mercury** |
| --- | --- | --- |
| Chloromethane | [CLMETH](https://www.ccdc.cam.ac.uk/structures/Search?Ccdcid=CLMETH&DatabaseToSearch=Published) | [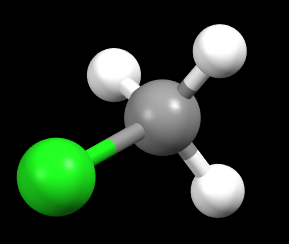](CIF/Alkyl_halides/CLMETH.cif) |
| Diiodomethane | [DIMETH03](https://www.ccdc.cam.ac.uk/structures/Search?Ccdcid=DIMETH03&DatabaseToSearch=Published) | [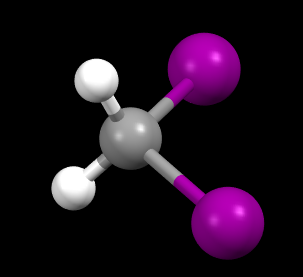](CIF/Alkyl_halides/DIMETH03.cif) |
| Bromomethane | [MBRMET10](https://www.ccdc.cam.ac.uk/structures/Search?Ccdcid=MBRMET10&DatabaseToSearch=Published) | [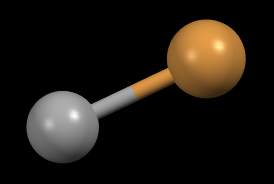](CIF/Alkyl_halides/MBRMET10.cif) |
| Iodomethane | [MIMETH10](https://www.ccdc.cam.ac.uk/structures/Search?Ccdcid=MIMETH10&DatabaseToSearch=Published) | [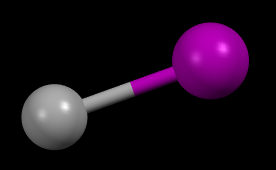](/winfs-uni.top.gwdg.de/eirmer$/XLAB/CSD/Teaching%20Materials%20EI/CIF/Alkyl_halides/MIMETH10.cif) |
| Carbon tetraiodide | [ZZZKDW01](https://www.ccdc.cam.ac.uk/structures/Search?Ccdcid=ZZZKDW01&DatabaseToSearch=Published) | [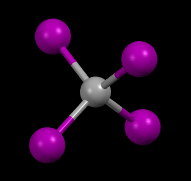](CIF/Alkyl_halides/ZZZKDW01.cif) |
| Dichloromethane (solvent) | [BEJKUW](https://www.ccdc.cam.ac.uk/structures/Search?Ccdcid=BEJKUW&DatabaseToSearch=Published) | [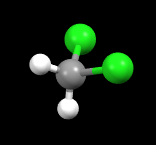](CIF/Alkyl_halides/BEJKUW.cif) |
| Dibromohexafluoropropane | [BOCGAB](https://www.ccdc.cam.ac.uk/structures/Search?Ccdcid=BOCGAB&DatabaseToSearch=Published) | [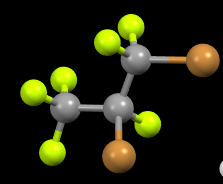](CIF/Alkyl_halides/BOCGAB.cif) |

# Alkanols

| **Name** | **WebCSD** | **Mercury** |
| --- | --- | --- |
| Methanol | [METHOL](https://www.ccdc.cam.ac.uk/structures/Search?Ccdcid=METHOL&DatabaseToSearch=Published) | [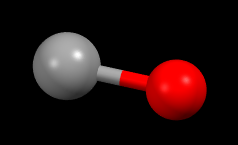](CIF/Alkanols/METHOL.cif) |
| Ethanol | [ETANOL](https://www.ccdc.cam.ac.uk/structures/Search?Ccdcid=ETANOL&DatabaseToSearch=Published) | [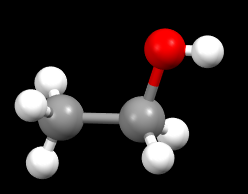](CIF/Alkanols/ETANOL.cif) |
| Propan-1-ol (solvent) | [VENVAM](https://www.ccdc.cam.ac.uk/structures/Search?Ccdcid=VENVAM&DatabaseToSearch=Published) | [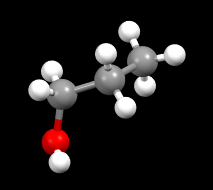](CIF/Alkanols/VENVAM.cif) |
| Isopropanol (solvent) | [ABALEV](https://www.ccdc.cam.ac.uk/structures/Search?Ccdcid=ABALEV&DatabaseToSearch=Published) | [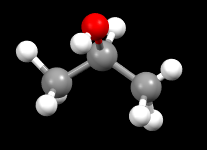](CIF/Alkanols/ABALEV.cif) |
| Butan-2-ol (solvent) | [AVEPIB](https://www.ccdc.cam.ac.uk/structures/Search?Ccdcid=AVEPIB&DatabaseToSearch=Published) | [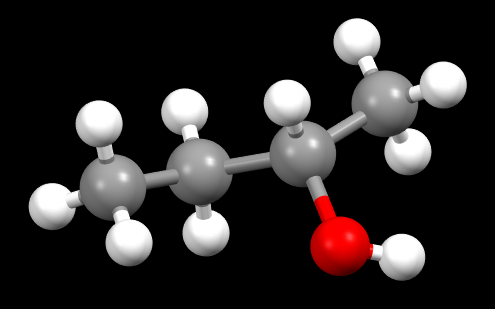](CIF/Alkanols/ABALEV.cif) |

# Amines

| **Name** | **WebCSD** | **Mercury** |
| --- | --- | --- |
| 1,2-Diaminoethane | [ETDIAM12](https://www.ccdc.cam.ac.uk/structures/Search?Ccdcid=ETDIAM12&DatabaseToSearch=Published) | [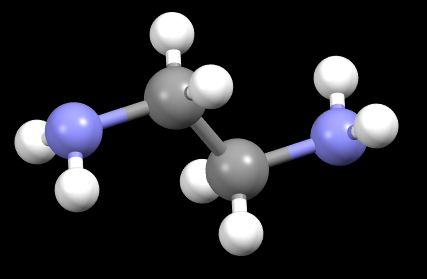](CIF/Amines/ETDIAM12.cif) |
| Methylamine | [METAMI](https://www.ccdc.cam.ac.uk/structures/Search?Ccdcid=METAMI&DatabaseToSearch=Published) | [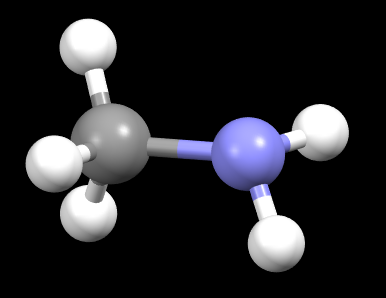](CIF/Amines/METAMI.cif) |
| Aniline | [BAZGOY](https://www.ccdc.cam.ac.uk/structures/Search?Ccdcid=BAZGOY&DatabaseToSearch=Published) | [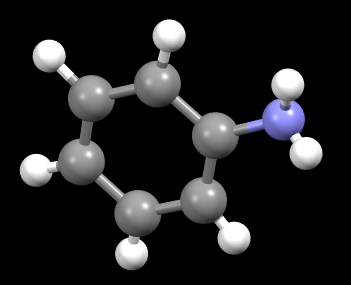](CIF/Amines/BAZGOY.cif) |
| Urea | [UREAXX](https://www.ccdc.cam.ac.uk/structures/Search?Ccdcid=UREAXX&DatabaseToSearch=Published) | [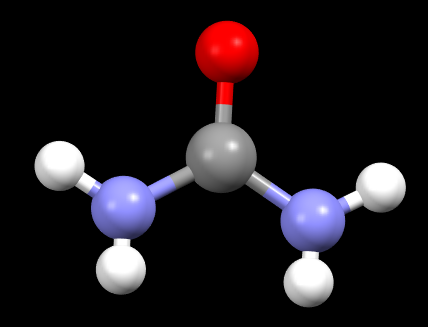](CIF/Amines/UREAXX.cif) |
| Trimethylamine | [CEKGUU01](https://www.ccdc.cam.ac.uk/structures/Search?Ccdcid=CEKGUU01&DatabaseToSearch=Published) | [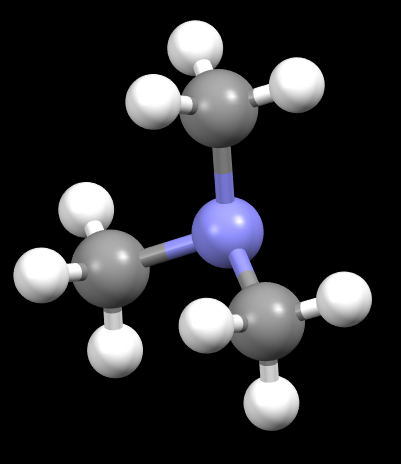](CIF/Amines/CEKGUU01.cif) |

# Aldehydes

| **Name** | **WebCSD** | **Mercury** |
| --- | --- | --- |
| Formaldehyde | [GURNEN](https://www.ccdc.cam.ac.uk/structures/Search?Ccdcid=GURNEN&DatabaseToSearch=Published) | [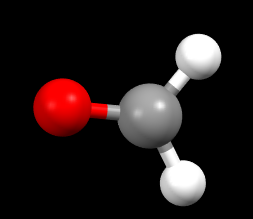](CIF/Aldehydes/GURNEN.cif) |

# Ketones

| **Name** | **WebCSD** | **Mercury** |
| --- | --- | --- |
| Acetone | [HIXHIF05](https://www.ccdc.cam.ac.uk/structures/Search?Ccdcid=HIXHIF05&DatabaseToSearch=Published) | [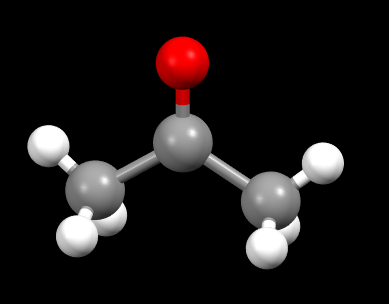](CIF/Ketones/HIXHIF05.cif) |
| Anthraquinone | [ANTQUO08](https://www.ccdc.cam.ac.uk/structures/Search?Ccdcid=ANTQUO08&DatabaseToSearch=Published) | [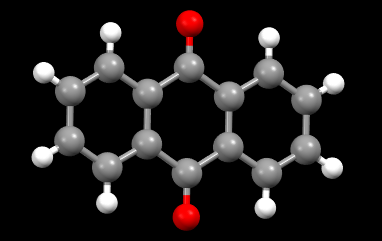](CIF/Ketones/ANTQUO08.cif) |
| Benzophenone | [BPHENO03](https://www.ccdc.cam.ac.uk/structures/Search?Ccdcid=BPHENO03&DatabaseToSearch=Published) | [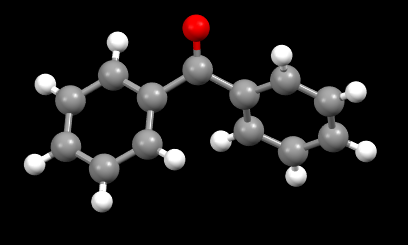](CIF/Ketones/BPHENO03.cif) |

# Carboxylic acids

| **Name** | **WebCSD** | **Mercury** |
| --- | --- | --- |
| Formic acid (methanoic acid) | [FORMAC01](https://www.ccdc.cam.ac.uk/structures/Search?Ccdcid=FORMAC01&DatabaseToSearch=Published) | [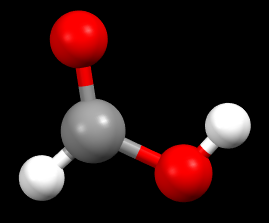](CIF/Carboxylic_acids/FORMAC01.cif) |
| Acetic acid (ethanoic acid) | [ACETAC07](https://www.ccdc.cam.ac.uk/structures/Search?Ccdcid=ACETAC07&DatabaseToSearch=Published) | [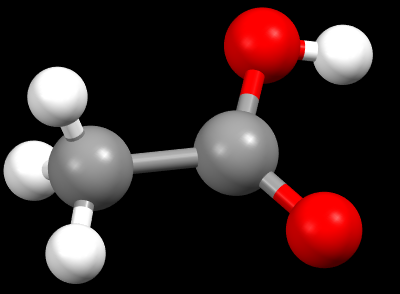](CIF/Carboxylic_acids/ACETAC07.cif) |
| Benzoic acid | [BENZAC02](https://www.ccdc.cam.ac.uk/structures/Search?Ccdcid=BENZAC02&DatabaseToSearch=Published) | [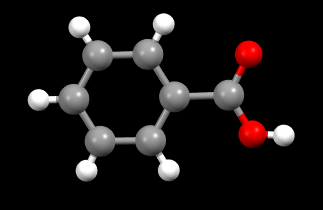](CIF/Carboxylic_acids/BENZAC02.cif) |
| Prop-2-ene acid | [ACRLAC02](https://www.ccdc.cam.ac.uk/structures/Search?Ccdcid=ACRLAC02&DatabaseToSearch=Published) | [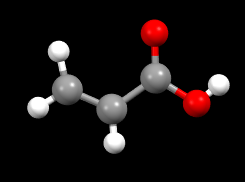](CIF/Carboxylic_acids/ACRLAC02.cif) |
| Adipic acid | [ADIPAC04](https://www.ccdc.cam.ac.uk/structures/Search?Ccdcid=ADIPAC04&DatabaseToSearch=Published) | [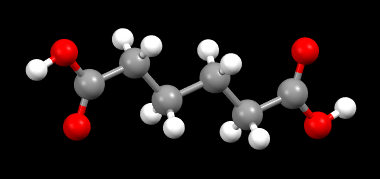](CIF/Carboxylic_acids/ADIPAC04.cif) |
| L-(+)-lactic acid | [YILLAG](https://www.ccdc.cam.ac.uk/structures/Search?Ccdcid=YILLAG&DatabaseToSearch=Published) | [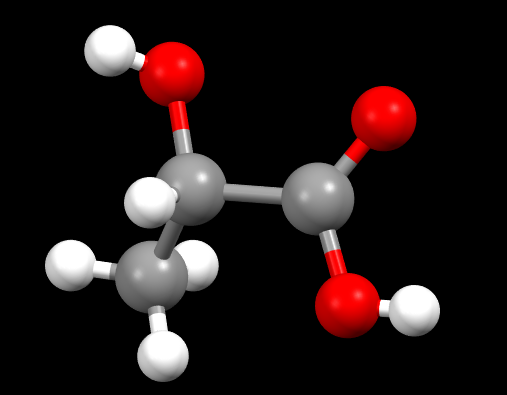](CIF/Carboxylic_acids/YILLAG.cif) |
| Citric acid | [CITRAC10](https://www.ccdc.cam.ac.uk/structures/Search?Ccdcid=CITRAC10&DatabaseToSearch=Published) | [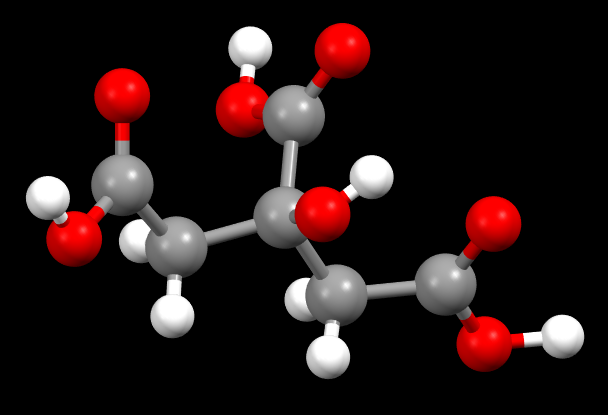](CIF/Carboxylic_acids/CITRAC10.cif) |
| Citric acid monohydrate | [CITARC](https://www.ccdc.cam.ac.uk/structures/Search?Ccdcid=CITARC&DatabaseToSearch=Published) | [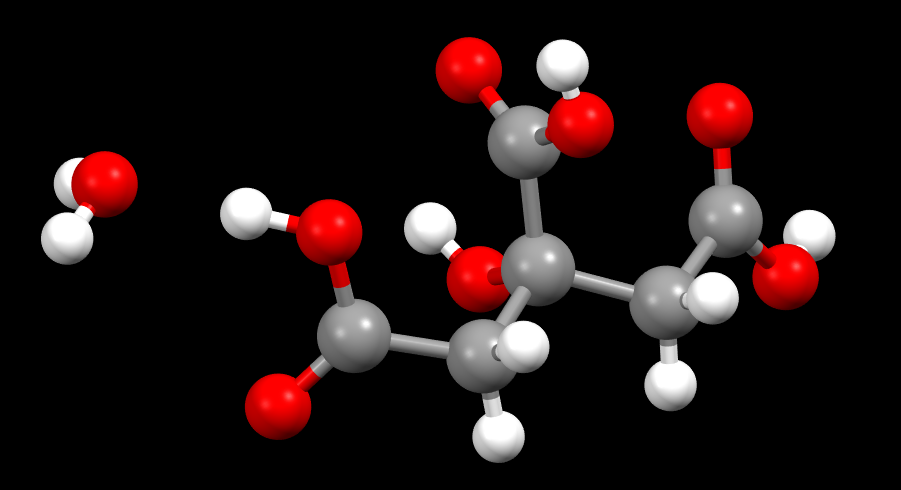](CIF/Carboxylic_acids/CITARC.cif) |
| Fumaric acid | [FUMAAC01](https://www.ccdc.cam.ac.uk/structures/Search?Ccdcid=FUMAAC01&DatabaseToSearch=Published) | [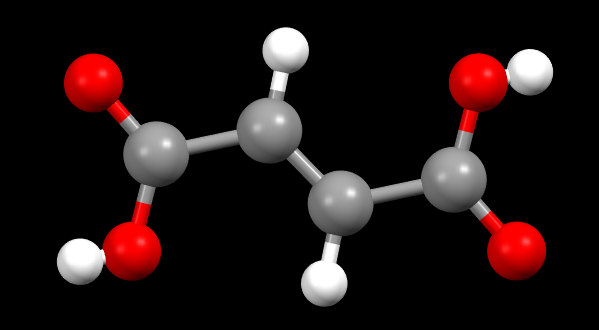](CIF/Carboxylic_acids/FUMAAC01.cif) |
| Monofluoroacetic acid | [FACETC10](https://www.ccdc.cam.ac.uk/structures/Search?Ccdcid=FACETC10&DatabaseToSearch=Published) | [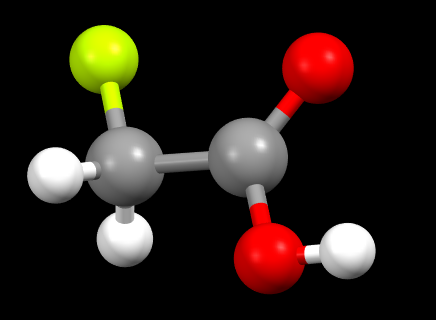](CIF/Carboxylic_acids/FACETC10.cif) |
| Sodium acetate | [BOPKOG10](https://www.ccdc.cam.ac.uk/structures/Search?Ccdcid=BOPKOG10&DatabaseToSearch=Published) | [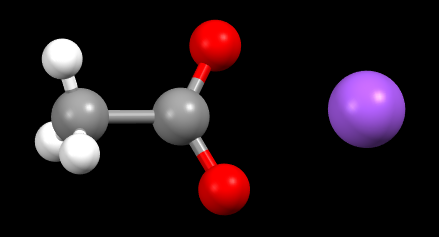](CIF/Carboxylic_acids/BOPKOG10.cif) |
| Calcium formate | [CAFORM05](https://www.ccdc.cam.ac.uk/structures/Search?Ccdcid=CAFORM05&DatabaseToSearch=Published) | [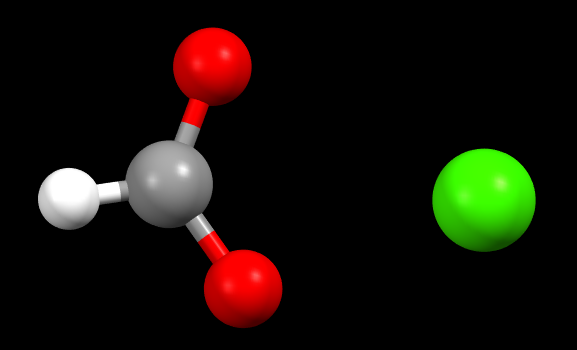](CIF/Carboxylic_acids/CAFORM05.cif) |

# Esters

| **Name** | **WebCSD** | **Mercury** |
| --- | --- | --- |
| Methylacetate | [BAHSUY](https://www.ccdc.cam.ac.uk/structures/Search?Ccdcid=BAHSUY&DatabaseToSearch=Published) | [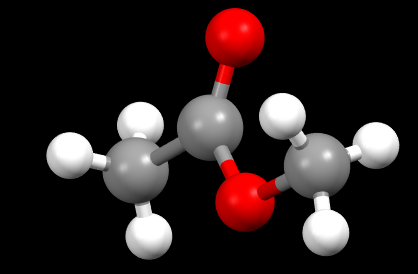](CIF/Esters/BAHSUY.cif) |
| Ethylpropionate | [YARZUN03](https://www.ccdc.cam.ac.uk/structures/Search?Ccdcid=YARZUN03&DatabaseToSearch=Published) | [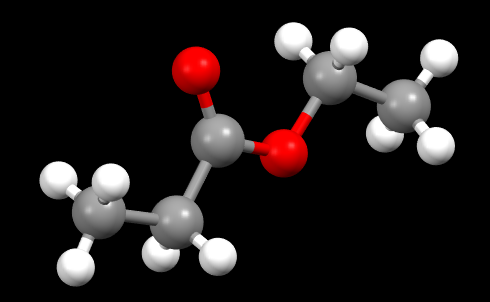](CIF/Esters/YARZUN03.cif) |

# Amino acids

| **Name** | **WebCSD** | **Mercury** |
| --- | --- | --- |
| Glycine | [GLYCIN](https://www.ccdc.cam.ac.uk/structures/Search?Ccdcid=GLYCIN&DatabaseToSearch=Published) | [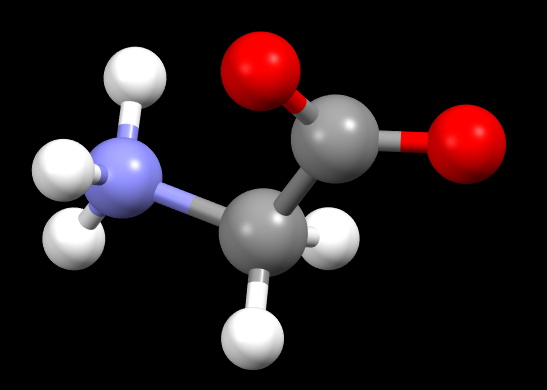](CIF/Amino_acids/GLYCIN.cif) |
| D-Alanine | [ALUCAL05](https://www.ccdc.cam.ac.uk/structures/Search?Ccdcid=ALUCAL05&DatabaseToSearch=Published) | [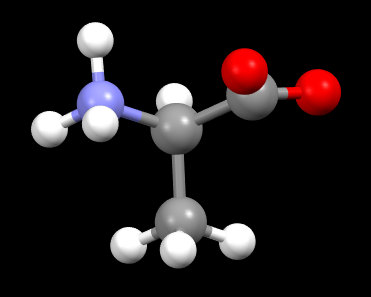](CIF/Amino_acids/ALUCAL05.cif) |
| L-Alanine | [LALNIN23](https://www.ccdc.cam.ac.uk/structures/Search?Ccdcid=LALNIN23&DatabaseToSearch=Published) | [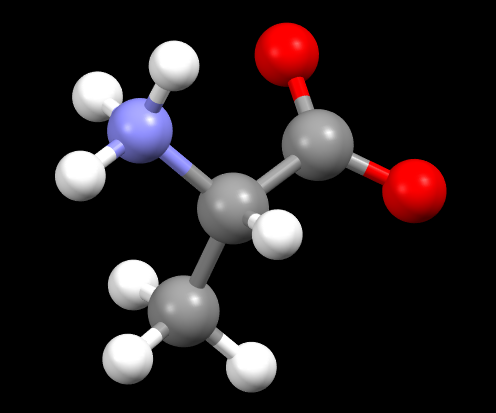](CIF/Amino_acids/LALNIN23.cif) |
| DL-Valine | [VALIDL](https://www.ccdc.cam.ac.uk/structures/Search?Ccdcid=VALIDL&DatabaseToSearch=Published) | [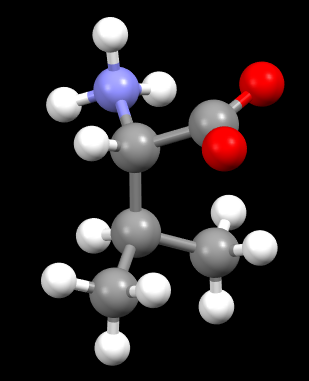](CIF/Amino_acids/VALIDL.cif) |
| L-Proline | [PROLIN](https://www.ccdc.cam.ac.uk/structures/Search?Ccdcid=PROLIN&DatabaseToSearch=Published) | [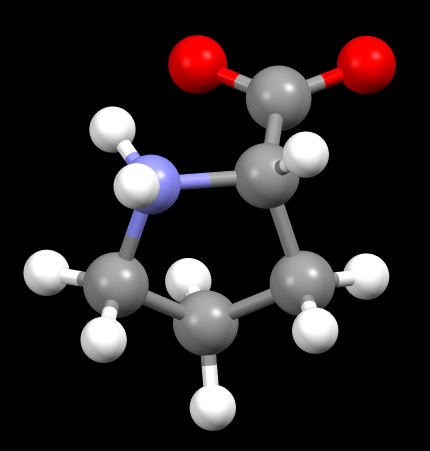](CIF/Amino_acids/PROLIN.cif) |
| L-Glutamic acid | [LGLUAC01](https://www.ccdc.cam.ac.uk/structures/Search?Ccdcid=LGLUAC01&DatabaseToSearch=Published) | [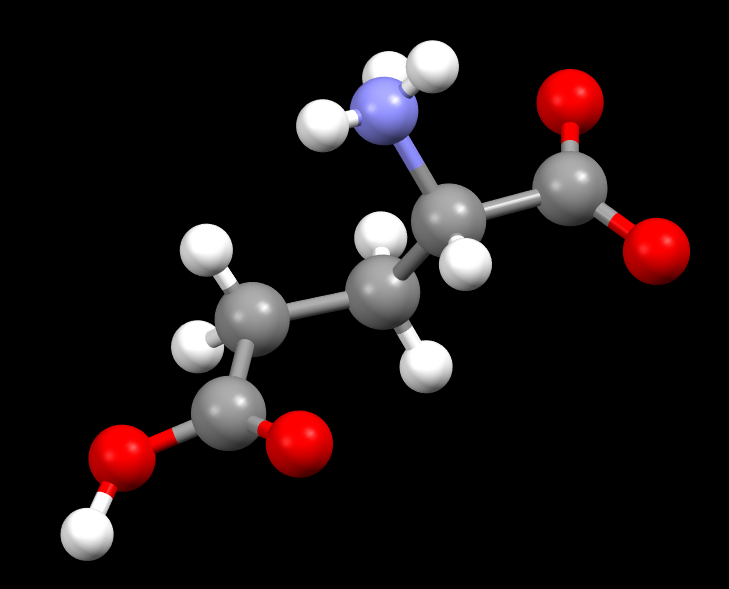](CIF/Amino_acids/LGLUAC01.cif) |
| L-Serine | [LSERIN01](https://www.ccdc.cam.ac.uk/structures/Search?Ccdcid=LSERIN01&DatabaseToSearch=Published) | [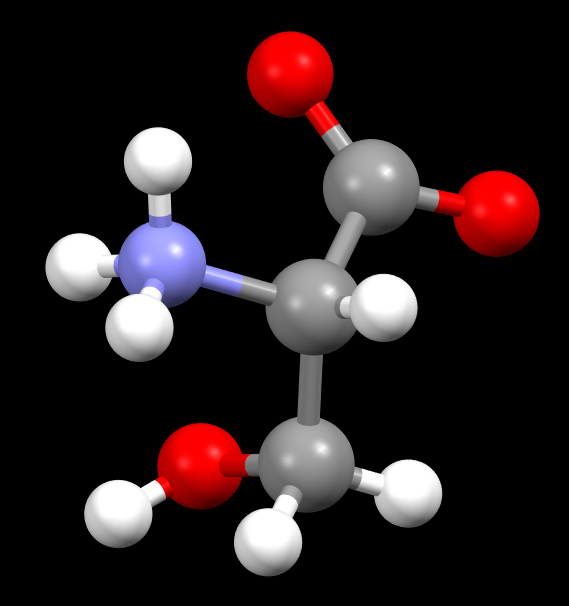](CIF/Amino_acids/LSERIN01.cif) |
| L-Threonine | [LTHREO01](https://www.ccdc.cam.ac.uk/structures/Search?Ccdcid=LTHREO01&DatabaseToSearch=Published) | [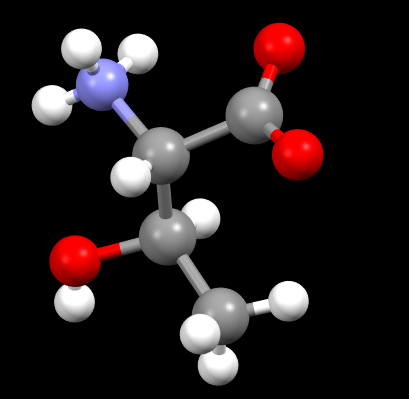](CIF/Amino_acids/LTHREO01.cif) |
| L-Tyrosine | [LTYROS10](https://www.ccdc.cam.ac.uk/structures/Search?Ccdcid=LTYROS10&DatabaseToSearch=Published) | [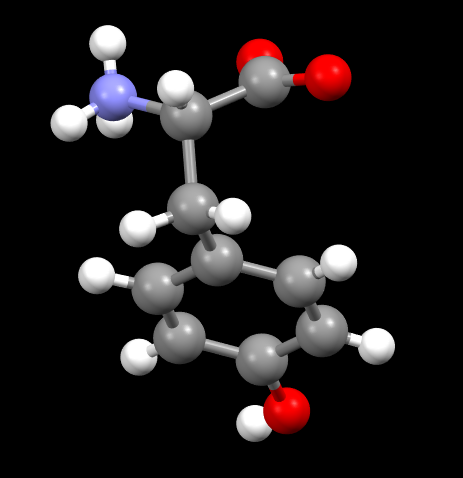](CIF/Amino_acids/LTYROS10.cif) |
| L-Cystine | [LCYSTI10](https://www.ccdc.cam.ac.uk/structures/Search?Ccdcid=LCYSTI10&DatabaseToSearch=Published) | [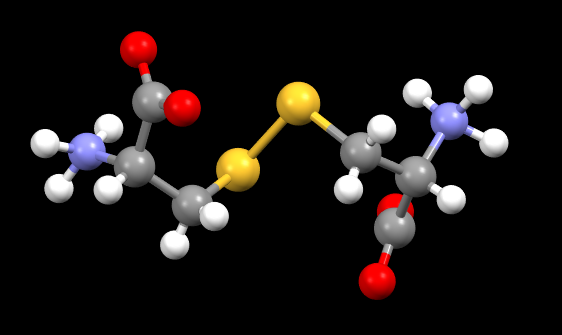](CIF/Amino_acids/LCYSTI10.cif) |
| L-Cysteine | [LCYSTN22](https://www.ccdc.cam.ac.uk/structures/Search?Ccdcid=LCYSTN22&DatabaseToSearch=Published) | [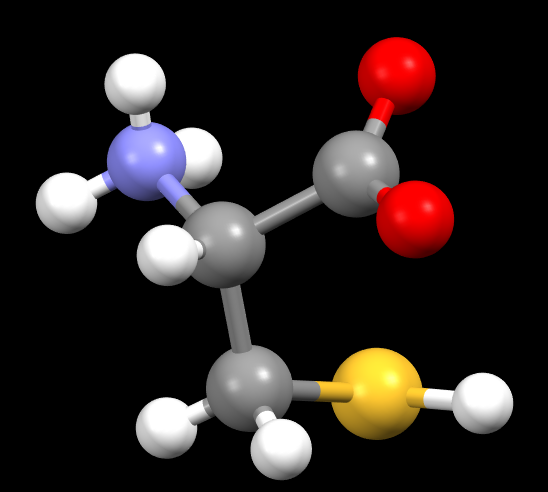](CIF/Amino_acids/LCYSTN22.cif) |
| DL-Methionine | [DLMETA05](https://www.ccdc.cam.ac.uk/structures/Search?Ccdcid=DLMETA05&DatabaseToSearch=Published) | [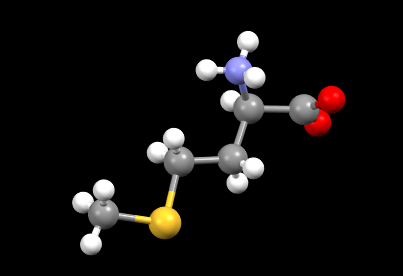](CIF/Amino_acids/DLMETA05.cif) |
| L-Glutamine | [GLUTAM01](https://www.ccdc.cam.ac.uk/structures/Search?Ccdcid=GLUTAM01&DatabaseToSearch=Published) | 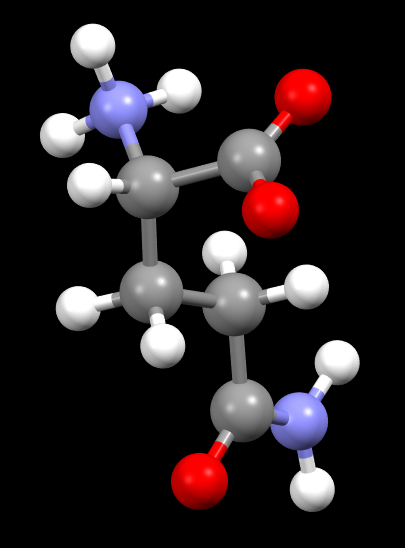 |
| L-Asparagine Monohydrate | [ASPARM08](https://www.ccdc.cam.ac.uk/structures/Search?Ccdcid=ASPARM08&DatabaseToSearch=Published) | [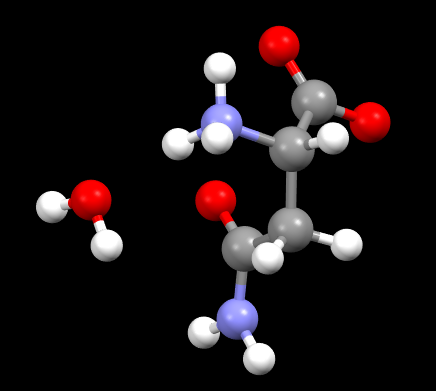](CIF/Amino_acids/ASPARM08.cif) |
| DL-Arginine Dihydrate | [WIJNEI](https://www.ccdc.cam.ac.uk/structures/Search?Ccdcid=WIJNEI&DatabaseToSearch=Published) | [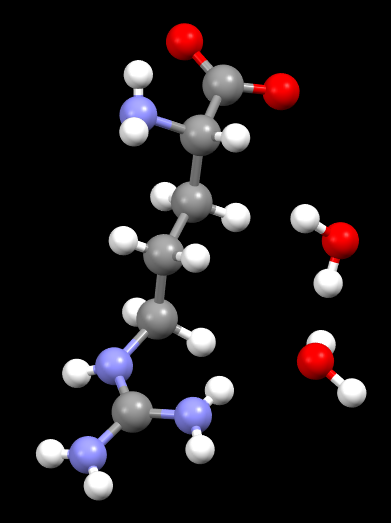](CIF/Amino_acids/WIJNEI.cif) |

# Peptides

| **Name** | **WebCSD** | **Mercury** |
| --- | --- | --- |
| L-Alanyl-L-Alanine | [ALALHC](https://www.ccdc.cam.ac.uk/structures/Search?Ccdcid=ALALHC&DatabaseToSearch=Published) | [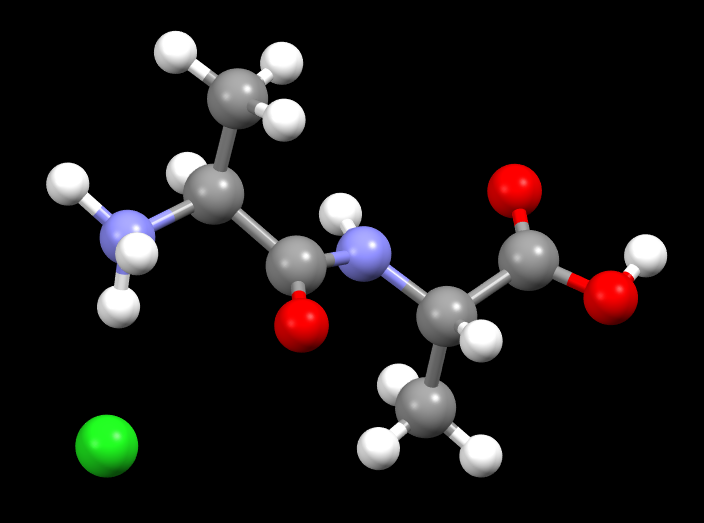](CIF/Peptides/ALALHC.cif) |

# Carbohydrates

| **Name** | **WebCSD** | **Mercury** |
| --- | --- | --- |
| alpha-D-Glucose | [GLUCSA](https://www.ccdc.cam.ac.uk/structures/Search?Ccdcid=GLUCSA&DatabaseToSearch=Published) | [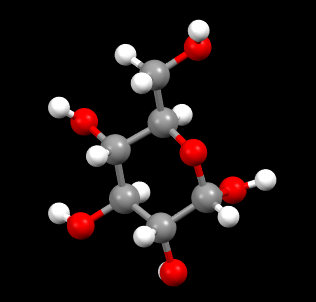](CIF/Carbohydrates/GLUCSA.cif) |
| beta-D-Glucose | [GLUCSE02](https://www.ccdc.cam.ac.uk/structures/Search?Ccdcid=GLUCSE02&DatabaseToSearch=Published) | [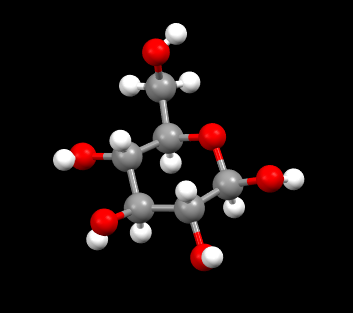](CIF/Carbohydrates/GLUCSE02.cif) |
| beta-D-Fructose | [FRUCTO11](https://www.ccdc.cam.ac.uk/structures/Search?Ccdcid=FRUCTO11&DatabaseToSearch=Published) | [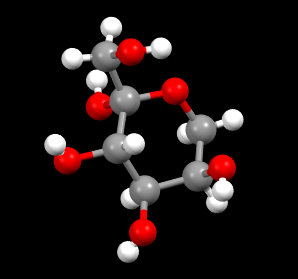](CIF/Carbohydrates/FRUCTO11.cif) |
| Sucrose | [SUCROS01](https://www.ccdc.cam.ac.uk/structures/Search?Ccdcid=SUCROS01&DatabaseToSearch=Published) | [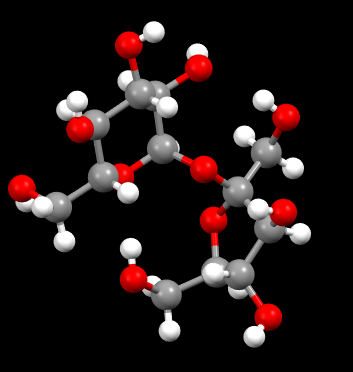](CIF/Carbohydrates/SUCROS01.cif) |
| beta-L-Arabinose | [ABINOS](https://www.ccdc.cam.ac.uk/structures/Search?Ccdcid=ABINOS&DatabaseToSearch=Published) | [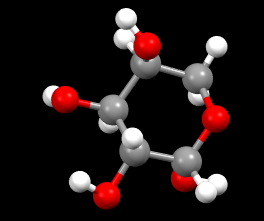](CIF/Carbohydrates/ABINOS.cif) |

# Vitamins

| **Name** | **WebCSD** | **Mercury** |
| --- | --- | --- |
| Vitamin A acid | [VITAAC01](https://www.ccdc.cam.ac.uk/structures/Search?Ccdcid=VITAAC01&DatabaseToSearch=Published) | [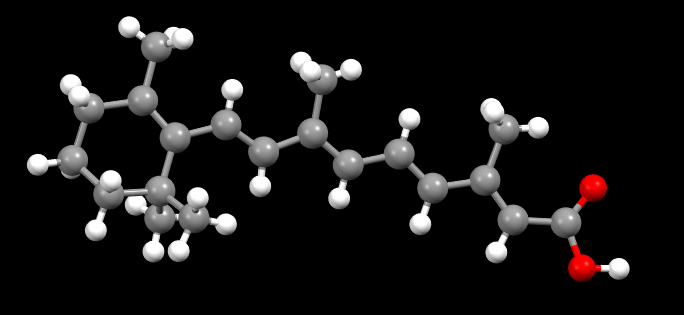](CIF/Vitamins/VITAAC01.cif) |
| Vitamin C | [LASCAC02](https://www.ccdc.cam.ac.uk/structures/Search?Ccdcid=LASCAC02&DatabaseToSearch=Published) | [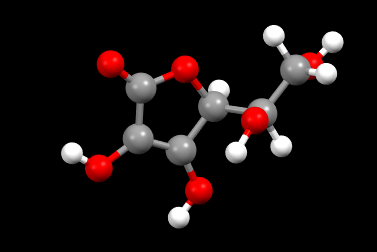](CIF/Vitamins/LASCAC02.cif) |

# Natural products

| **Name** | **WebCSD** | **Mercury** |
| --- | --- | --- |
| (-)Adrenalin | [ADRENL](https://www.ccdc.cam.ac.uk/structures/Search?Ccdcid=ADRENL&DatabaseToSearch=Published) | [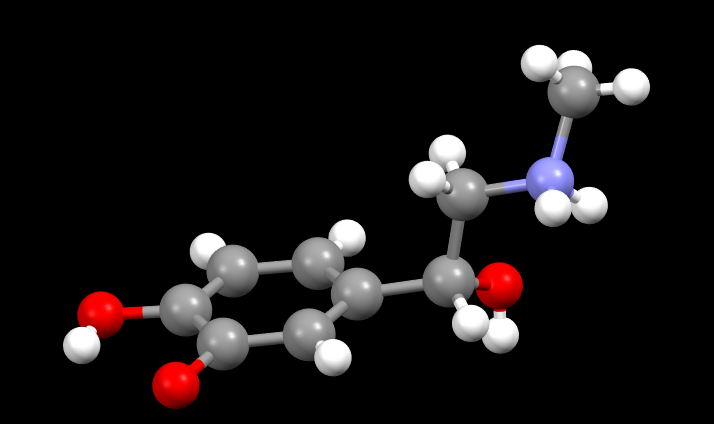](CIF/Natural_products/ADRENL.cif) |
| Adenosine | [ADENOS10](https://www.ccdc.cam.ac.uk/structures/Search?Ccdcid=ADENOS10&DatabaseToSearch=Published) |  |
| Aspirin, Acetylsalicylic acid | [ACSALA01](https://www.ccdc.cam.ac.uk/structures/Search?Ccdcid=ACSALA01&DatabaseToSearch=Published) |  |
| Caffeine monohydrate | [CAFINE](https://www.ccdc.cam.ac.uk/structures/Search?Ccdcid=CAFINE&DatabaseToSearch=Published) |  |
| beta-Carotene | [CARTEN02](https://www.ccdc.cam.ac.uk/structures/Search?Ccdcid=CARTEN02&DatabaseToSearch=Published) |  |

# Polymers

| **Name** | **WebCSD** | **Mercury** |
| --- | --- | --- |
| Poly(ethene) | [QILHUO01](https://www.ccdc.cam.ac.uk/structures/Search?Ccdcid=QILHUO01&DatabaseToSearch=Published) |  |
| Poly(propene) | [SUSJIZ](https://www.ccdc.cam.ac.uk/structures/Search?Ccdcid=SUSJIZ&DatabaseToSearch=Published) |  |
| Poly(1-butene) | [LEJKIU](https://www.ccdc.cam.ac.uk/structures/Search?Ccdcid=LEJKIU&DatabaseToSearch=Published) |  |
| Poly(styrene) | [SUSKOG](https://www.ccdc.cam.ac.uk/structures/Search?Ccdcid=SUSKOG&DatabaseToSearch=Published) |  |

## Alphabetical Index

(16)Annulene 7

(18)Annulene 7

1,3,5-Trinitrobenzene 6

2,4,6-Trinitrotoluene 6

2-Amino-5-nitrophenol 6

2-Hydroxyphenol 6

5-Brom-1,3-dichlor-2-iod-benzol 7

Acetic acid 11

Acetone 10

Acetylene 5

Acetylsalicylic acid 16

Adenosine 16

Adipic acid 11

Adrenalin 16

Alanine 12, 13

alpha-D-Glucose 15

Aniline 9

Annulene 7

Anthraquinone 7, 10

Arabinose 15

Arginine 14

Asparagine Monohydrate 14

Aspirin 16

Benzene 5

Benzoic acid 11

Benzonitrile 6

Benzophenone 10

beta-Carotene 16

beta-D-Fructose 15

beta-D-Glucose 15

beta-L-Arabinose 15

Bromomethane 8

But-2-in 5

Butan-2-ol 9

Butane 3

Caffeine monohydrate 16

Calcium formate 12

Carbon tetraiodide 8

Carotene 16

Catecholine 6

Chloromethane 7

Citric acid 11

Citric acid monohydrate 11

Cyanoacetlyene 5

Cyclobutane 3

Cyclohexane 4

Cyclohexene 4

Cyclo-octatetraene 4

Cyclopropane 3

Cysteine 14

Cystine 14

D-Alanine 12

Decadiene 4

Diaminoethane 9

Dibromohexafluoropropane 8

Dichloromethane 8

Diiodomethane 7

DL-Arginine Dihydrate 14

DL-Methionine 14

DL-Valine 13

Ethane 3

Ethanol 8

Ethene 4

Ethyne 5

Formaldehyde 10

Formic acid 11

Fructose 15

Fumaric acid 11

Glucose 15

Glutamic acid 13

Glutamine 14

Glycine 12

Heptane 3

Hexaaminobenzene 6

Hexane 3

Hydroxyphenol 6

Iodomethane 8

Isopropanol 9

L-(+)-lactic acid 11

L-Alanine 13

L-Alanyl-L-Alanine 15

L-Asparagine Monohydrate 14

L-Cysteine 14

L-Cystine 14

L-Glutamic acid 13

L-Glutamine 14

L-Proline 13

L-Serine 13

L-Threonine 13

L-Tyrosine 13

Methanol 8

Methionine 14

Methylamine 9

Monofluoroacetic acid 12

Naphthalene 7

Octa-2,4,6-triyne 5

Octadiene 4

Octadiin 5

Octane 3

Pentane 3

Phenol 6

Poly(1-butene) 17

Poly(ethene) 17

Poly(propene) 17

Poly(styrene) 17

Proline 13

Prop-2-ene acid 11

Propan-1-ol 9

Propane 3

Serine 13

Sodium acetate 12

Sucrose 15

Tetramethylethene 4

Threonine 13

Toluene 5

Trimethylamine 10

Trinitrobenzene 6

Trinitrotoluene 6

Tyrosine 13

Urea 9

Valine 13

Vitamin A acid 16

Vitamin C 16
