# Supplementary material for: Crystallography in school
Source: J Appl Crystallogr. 2025 Sep 12;58(Pt 5):1802–9. doi: 10.1107/S1600576725007459 (PMC12502877; doi:10.1107/S1600576725007459)
Supplement: Supplementary file 3 [file j-58-01802-sup3.zip › Teaching Subset substance classes.pdf]

The CSD Teaching Subset
sorted by substance classes

A selection of school-relevant organic structures

by Erhard Irmer (XLAB Göttingen, Germany)

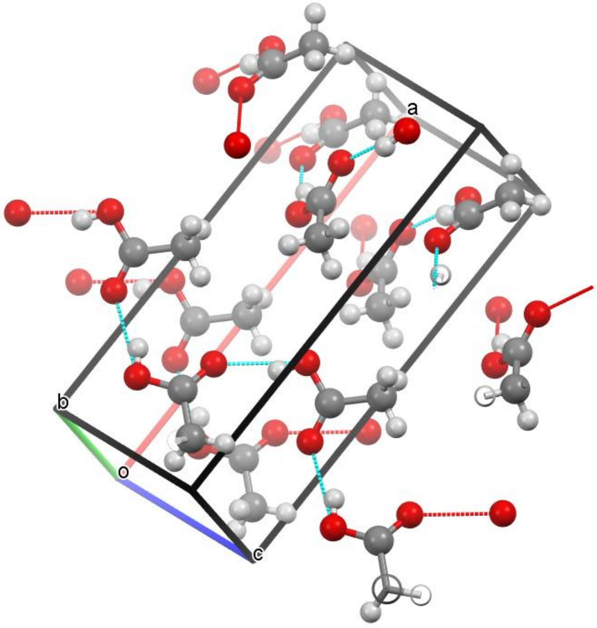

Contents

Contents 2

1. Alkanes 3
2. Alkenes 4
3. Alkynes 5
4. Aromatics 5
5. Alkyl halides 7
6. Alkanols 8
7. Amines 9
8. Aldehydes 10
9. Ketones 10
10. Carboxylic acids 11
11. Esters 12
12. Amino acids 12
13. Peptides 15
14. Carbohydrates 15
15. Vitamins 16
16. Natural products 16
17. Polymers 17

Alphabetical Index 18

1. Alkanes

Name	WebCSD	Mercury
Ethane	ETHANE01	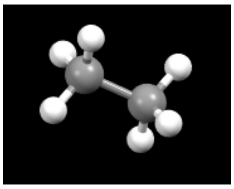
Propane	JAYDUI	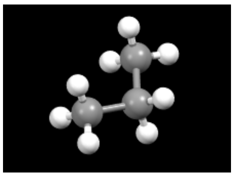
n-Butane	DUCKOB04	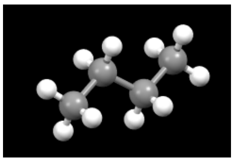
n-Pentane	PENTAN01	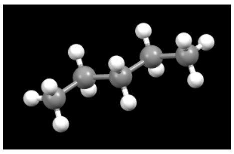
n-Hexane	HEXANE01	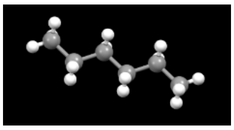
n-Heptane	HEPTAN03	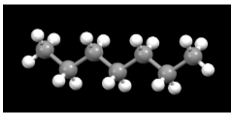
n-Octane	OCTANE12	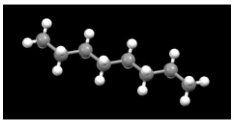
Cyclopropane	QQQCIS01	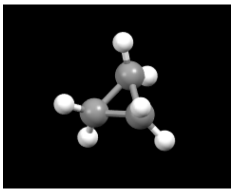
Cyclobutane	ZZZWEO02	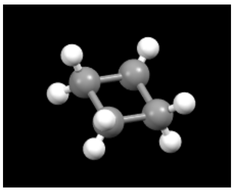

Cyclohexane	CYCHEX	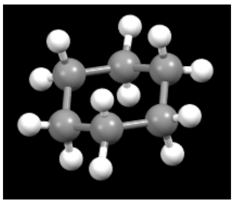

2. Alkenes

Name	WebCSD	Mercury
Ethene	ETHLEN10	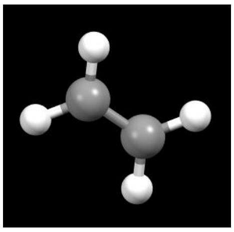
Tetramethylethene	PAPVAD	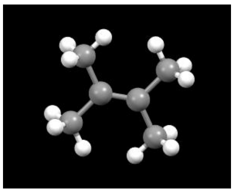
1,7-Octadiene	XOMHUC	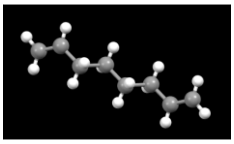
1,9-Decadiene	XOMJAK	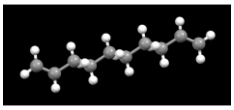
Cyclohexene	COVJON	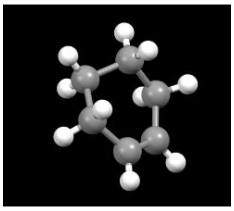
Cyclo-octatetraene	ZZZSAE01	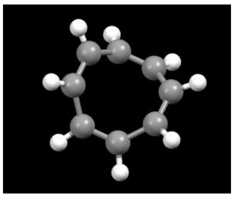

3. Alkynes

Name	WebCSD	Mercury
Ethyne, Acetylene	ACETYL03	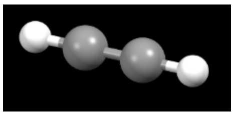
But-2-in hydrogen chloride	JUFDUJ	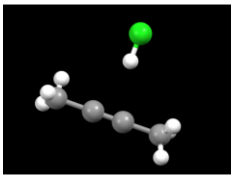
Octa-2,4,6-triyne	OCTRNE	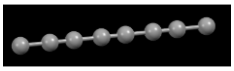
Cyanoacetlyene	CAACTY	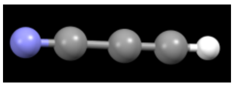
1,7-Octadiin	XOMJEO	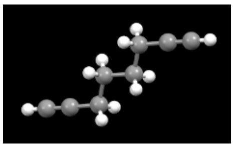

4. Aromatics

Name	WebCSD	Mercury
Benzene (neutron diffraction)	BENZEN	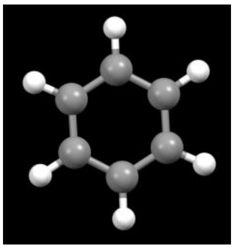
Benzene	BENZEN02	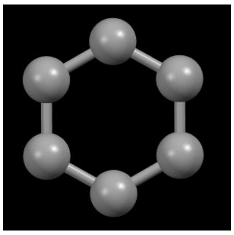
Toluene	TOLUEN	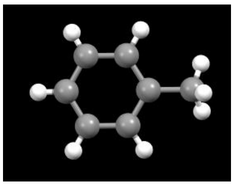

Phenol	PHENOL03	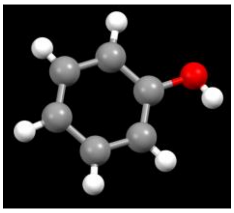
Catecholine, 2-Hydroxyphenol	CATCOL13	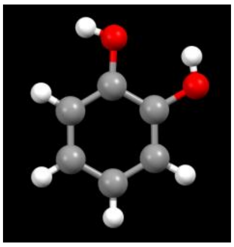
2-Amino-5-nitrophenol	AMNPHA	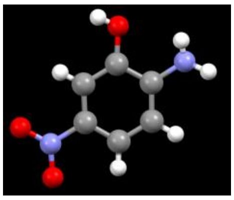
1,3,5-Trinitrobenzene	TNBENZ12	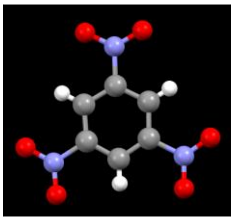
2,4,6-Trinitrotoluene	ZZZMUC01	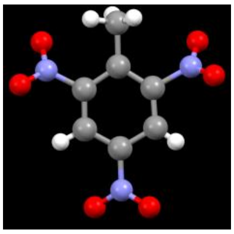
Hexaaminobenzene	ZZZWOU01	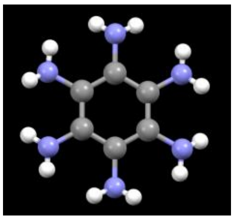
Benzonitrile	BZONTR	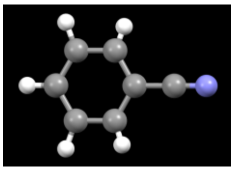

5-Brom-1,3-dichlor-2-iod-benzol	ACEPOO	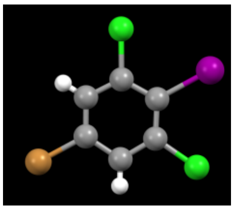
Naphthalene	NAPHTA12	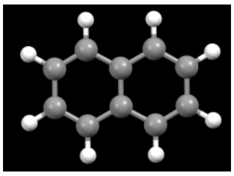
Anthraquinone	ANTQUO08	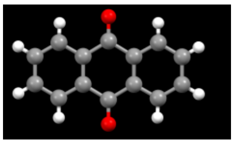
(16)Annulene	ANNULE01	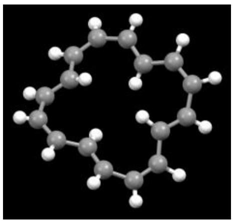
(18)Annulene	ANULEN	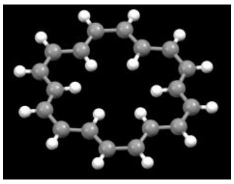

5. Alkyl halides

**Name**	**WebCSD**	**Mercury**
Chloromethane	CLMETH	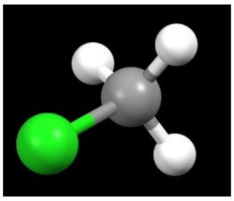
Diiodomethane	DIMETH03	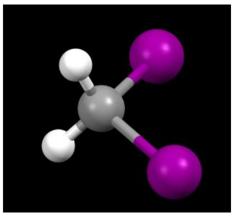

Bromomethane	MBRMET10	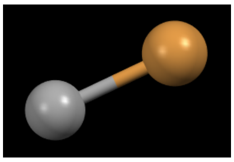
Iodomethane	MIMETH10	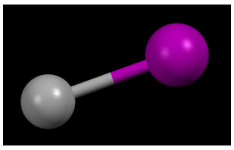
Carbon tetraiodide	ZZZKDW01	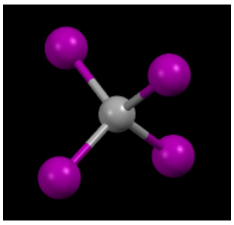
Dichloromethane (solvent)	BEJKUW	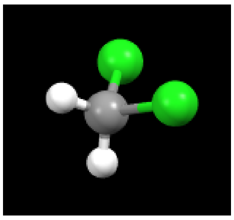
Dibromohexafluoropropane	BOCGAB	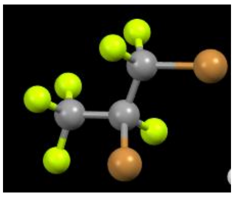

6. Alkanols

**Name**	**WebCSD**	**Mercury**
Methanol	METHOL	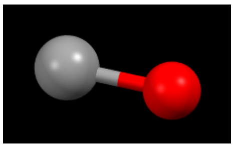
Ethanol	ETANOL	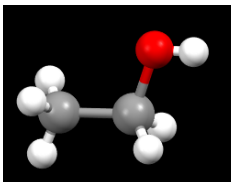

Propan-1-ol (solvent)	VENVAM	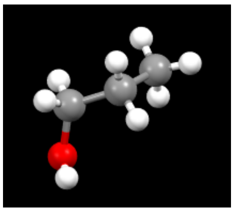
Isopropanol (solvent)	ABALEV	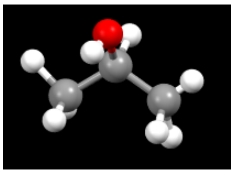
Butan-2-ol (solvent)	AVEPIB	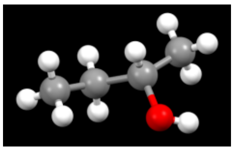

7. Amines

Name	WebCSD	Mercury
1,2-Diaminoethane	ETDIAM12	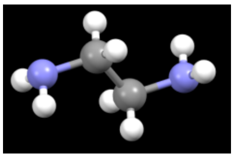
Methylamine	METAMI	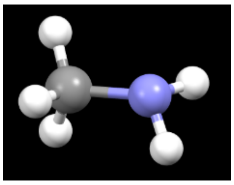
Aniline	BAZGOY	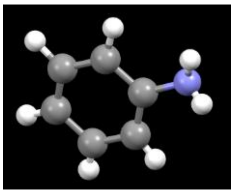
Urea	UREAXX	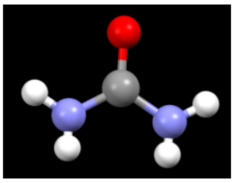

Trimethylamine	CEKGUU01	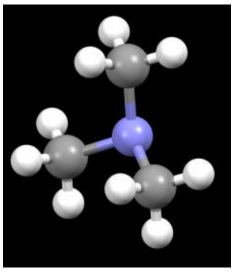

8. Aldehydes

Name	WebCSD	Mercury
Formaldehyde	GURNEN	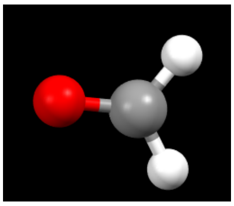

9. Ketones

Name	WebCSD	Mercury
Acetone	HIXHIF05	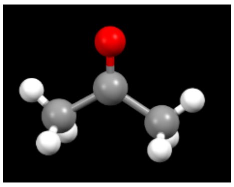
Anthraquinone	ANTQUO08	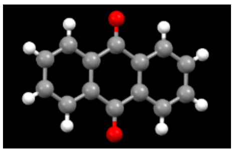
Benzophenone	BPHENO03	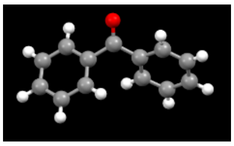

10. Carboxylic acids

Name	WebCSD	Mercury
Formic acid (methanoic acid)	FORMAC01	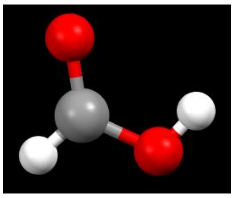
Acetic acid (ethanoic acid)	ACETAC07	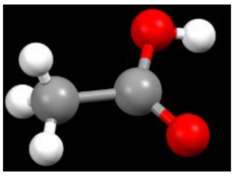
Benzoic acid	BENZAC02	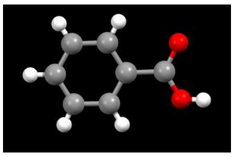
Prop-2-ene acid	ACRLAC02	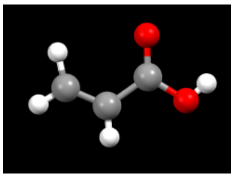
Adipic acid	ADIPAC04	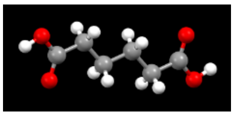
L-(+)-lactic acid	YILLAG	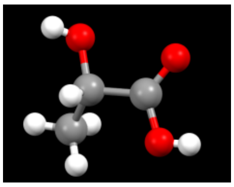
Citric acid	CITRAC10	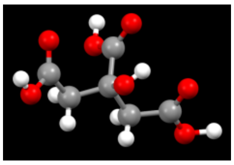
Citric acid monohydrate	CITARC	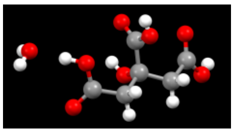
Fumaric acid	FUMAAC01	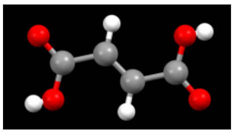

Monofluoroacetic acid	FACETC10	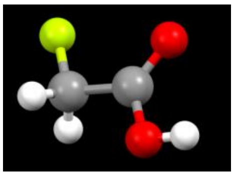
Sodium acetate	BOPKOG10	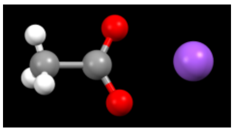
Calcium formate	CAFORM05	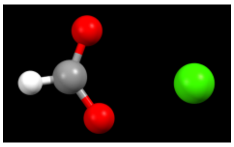

11. Esters

Name	WebCSD	Mercury
Methylacetate	BAHSUY	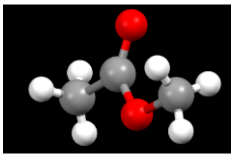
Ethylpropionate	YARZUN03	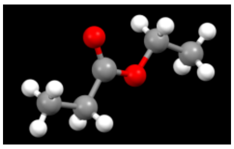

12. Amino acids

Name	WebCSD	Mercury
Glycine	GLYCIN	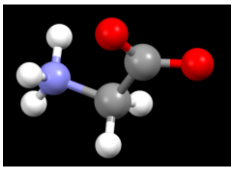
D-Alanine	ALUCAL05	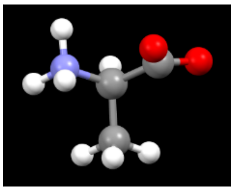

L-Alanine	LALNIN23	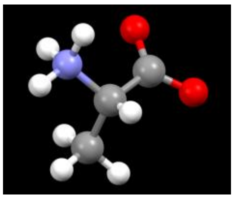
DL-Valine	VALIDL	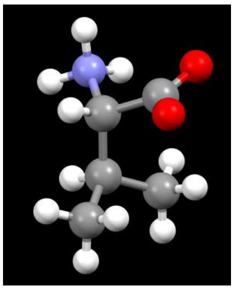
L-Proline	PROLIN	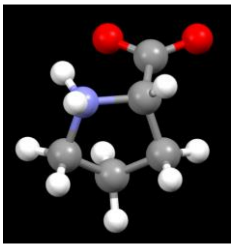
L-Glutamic acid	LGLUAC01	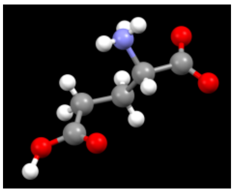
L-Serine	LSERIN01	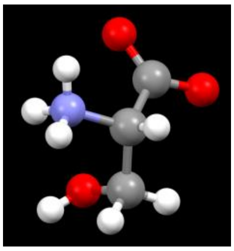
L-Threonine	LTHREO01	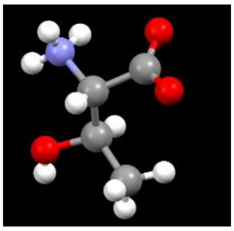
L-Tyrosine	LTYROS10	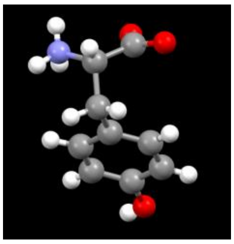

L-Cystine	LCYSTI10	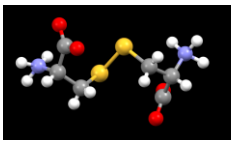
L-Cysteine	LCYSTN22	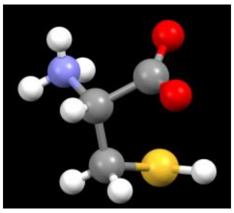
DL-Methionine	DLMETA05	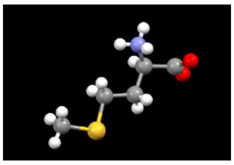
L-Glutamine	GLUTAM01	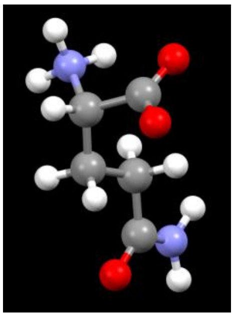
L-Asparagine Monohydrate	ASPARM08	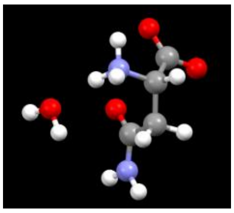
DL-Arginine Dihydrate	WIJNEI	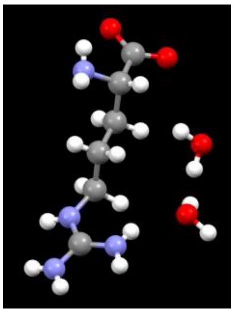

13. Peptides

Name	WebCSD	Mercury
L-Alanyl-L-Alanine	ALALHC	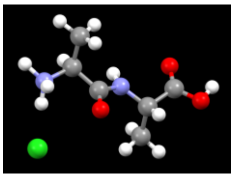

14. Carbohydrates

Name	WebCSD	Mercury
alpha-D-Glucose	GLUCSA	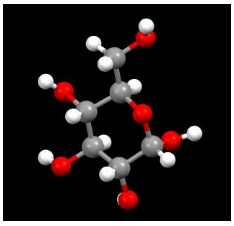
beta-D-Glucose	GLUCSE02	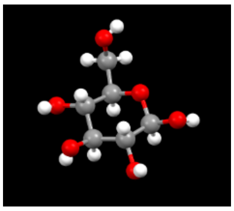
beta-D-Fructose	FRUCTO11	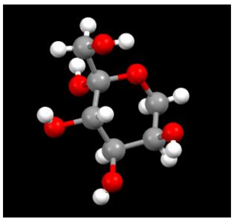
Sucrose	SUCROS01	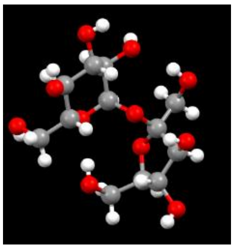
beta-L-Arabinose	ABINOS	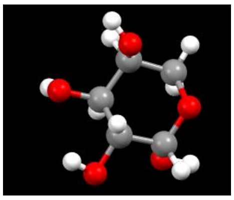

15. Vitamins

Name	WebCSD	Mercury
Vitamin A acid	VITAAC01	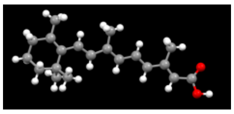
Vitamin C	LASCAC02	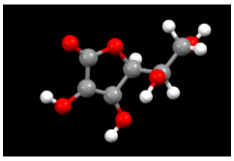

16. Natural products

Name	WebCSD	Mercury
(-)Adrenalin	ADRENL	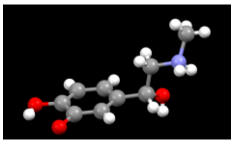
Adenosine	ADENOS10	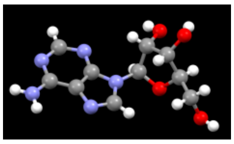
Aspirin, Acetylsalicylic acid	ACSALA01	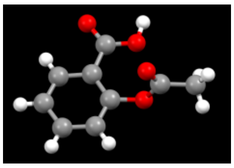
Caffeine monohydrate	CAFINE	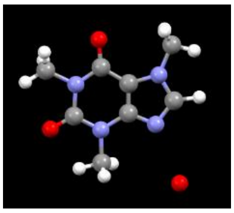
beta-Carotene	CARTEN02	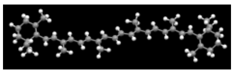

17. Polymers

Name	WebCSD	Mercury
Poly(ethene)	QILHUO01	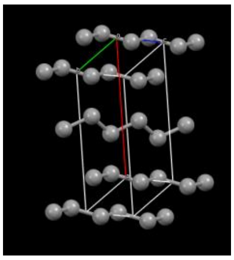
Poly(propene)	SUSJIZ	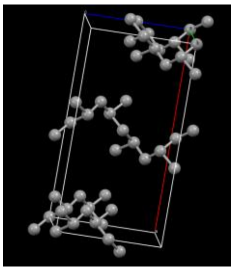
Poly(1-butene)	LEJKIU	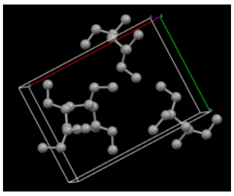
Poly(styrene)	SUSKOG	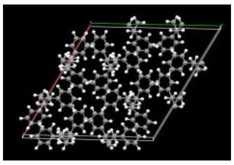

Alphabetical Index

(16)Annulene ... 7
(18)Annulene ... 7
1,3,5-Trinitrobenzene ... 6
2,4,6-Trinitrotoluene ... 6
2-Amino-5-nitrophenol ... 6
2-Hydroxyphenol ... 6
5-Brom-1,3-dichlor-2-iod-benzol ... 7
Acetic acid ... 11
Acetone ... 10
Acetylene ... 5
Acetylsalicylic acid ... 16
Adenosine ... 16
Adipic acid ... 11
Adrenalin ... 16
Alanine ... 12, 13
alpha-D-Glucose ... 15
Aniline ... 9
Annulene ... 7
Anthraquinone ... 7, 10
Arabinose ... 15
Arginine ... 14
Asparagine Monohydrate ... 14
Aspirin ... 16
Benzene ... 5
Benzoic acid ... 11
Benzonitrile ... 6
Benzophenone ... 10
beta-Carotene ... 16
beta-D-Fructose ... 15
beta-D-Glucose ... 15
beta-L-Arabinose ... 15
Bromomethane ... 8
But-2-in ... 5
Butan-2-ol ... 9
Butane ... 3
Caffeine monohydrate ... 16
Calcium formate ... 12
Carbon tetraiodide ... 8
Carotene ... 16
Catecholine ... 6
Chloromethane ... 7
Citric acid ... 11
Citric acid monohydrate ... 11
Cyanoacetlyene ... 5
Cyclobutane ... 3
Cyclohexane ... 4
Cyclohexene ... 4
Cyclo-octatetraene ... 4
Cyclopropane ... 3
Cysteine ... 14
Cystine ... 14
D-Alanine ... 12
Decadiene ... 4
Diaminoethane ... 9
Dibromohexafluoropropane ... 8
Dichloromethane ... 8
Diiodomethane ... 7
DL-Arginine Dihydrate ... 14
DL-Methionine ... 14
DL-Valine ... 13
Ethane ... 3
Ethanol ... 8
Ethene ... 4
Ethyne ... 5
Formaldehyde ... 10
Formic acid ... 11
Fructose ... 15
Fumaric acid ... 11
Glucose ... 15
Glutamic acid ... 13
Glutamine ... 14
Glycine ... 12
Heptane ... 3
Hexaaminobenzene ... 6
Hexane ... 3
Hydroxyphenol ... 6
Iodomethane ... 8
Isopropanol ... 9
L-(+)-lactic acid ... 11
L-Alanine ... 13
L-Alanyl-L-Alanine ... 15
L-Asparagine Monohydrate ... 14
L-Cysteine ... 14
L-Cystine ... 14
L-Glutamic acid ... 13
L-Glutamine ... 14
L-Proline ... 13
L-Serine ... 13
L-Threonine ... 13
L-Tyrosine ... 13
Methanol ... 8
Methionine ... 14
Methylamine ... 9
Monofluoroacetic acid ... 12
Naphthalene ... 7
Octa-2,4,6-triyne ... 5

Octadiene 4
Octadiin 5
Octane 3
Pentane 3
Phenol...6
Poly(1-butene)...17
Poly(ethene) 17
Poly(propene)...17
Poly(styrene) 17
Proline 13
Prop-2-ene acid 11
Propan-1-ol...9
Propane 3
Serine..13
Sodium acetate 12
Sucrose.. 15
Tetramethylethene 4
Threonine.. 13
Toluene 5
Trimethylamine.. 10
Trinitrobenzene 6
Trinitrotoluene... 6
Tyrosine 13
Urea 9
Valine 13
Vitamin A acid.. 16
Vitamin C... 16
