# Supplementary material for: Crystallography in school
Source: J Appl Crystallogr. 2025 Sep 12;58(Pt 5):1802–9. doi: 10.1107/S1600576725007459 (PMC12502877; doi:10.1107/S1600576725007459)
Supplement: Supplementary file 4 [file j-58-01802-sup4.pptx]

## Slide 1
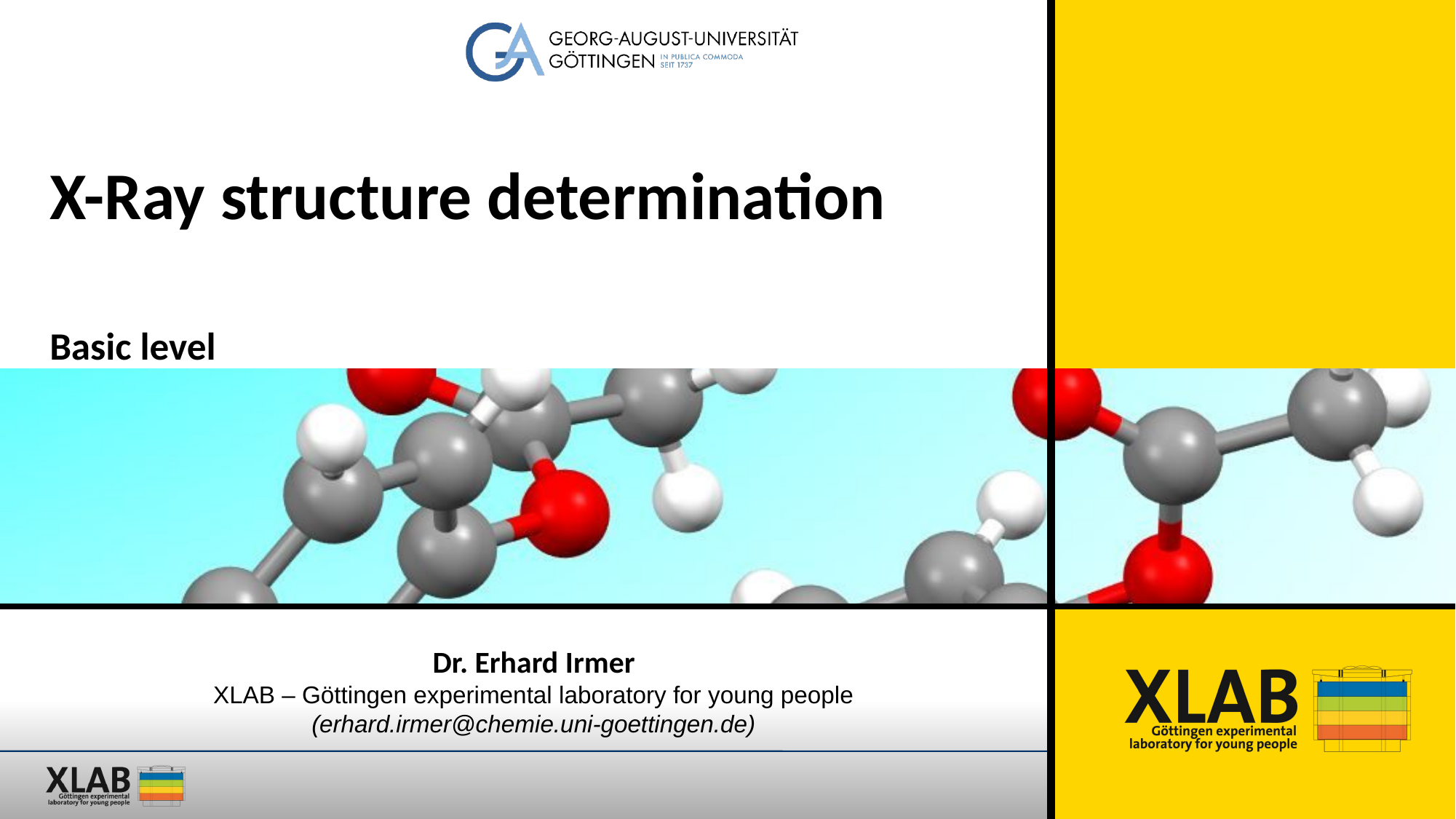

X-Ray structure determination
Basic level
Dr. Erhard Irmer
XLAB – Göttingen experimental laboratory for young people
(erhard.irmer@chemie.uni-goettingen.de)

## Slide 2
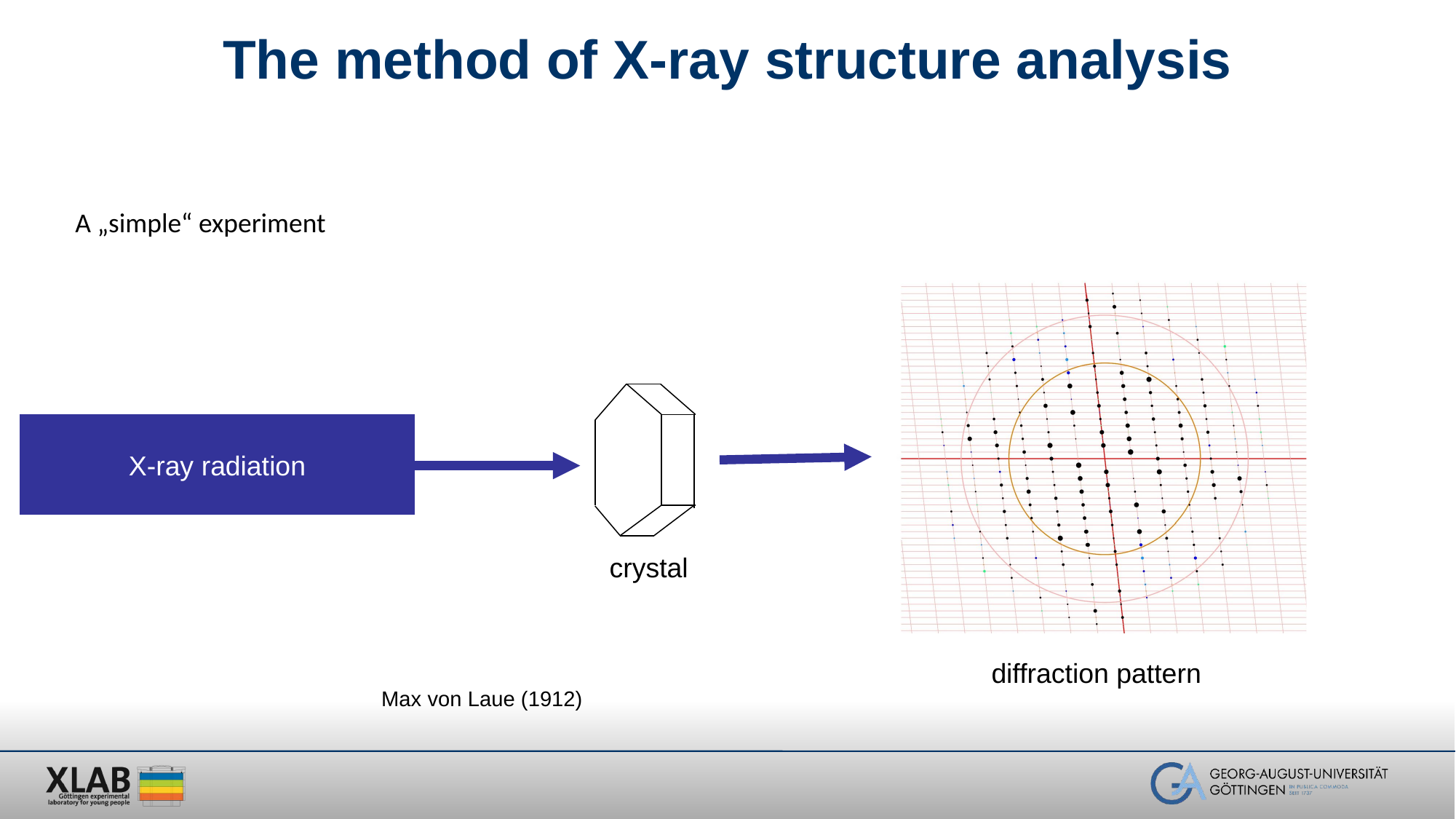

# The method of X-ray structure analysis
A „simple“ experiment
X-ray radiation
crystal
diffraction pattern
Max von Laue (1912)

## Slide 3
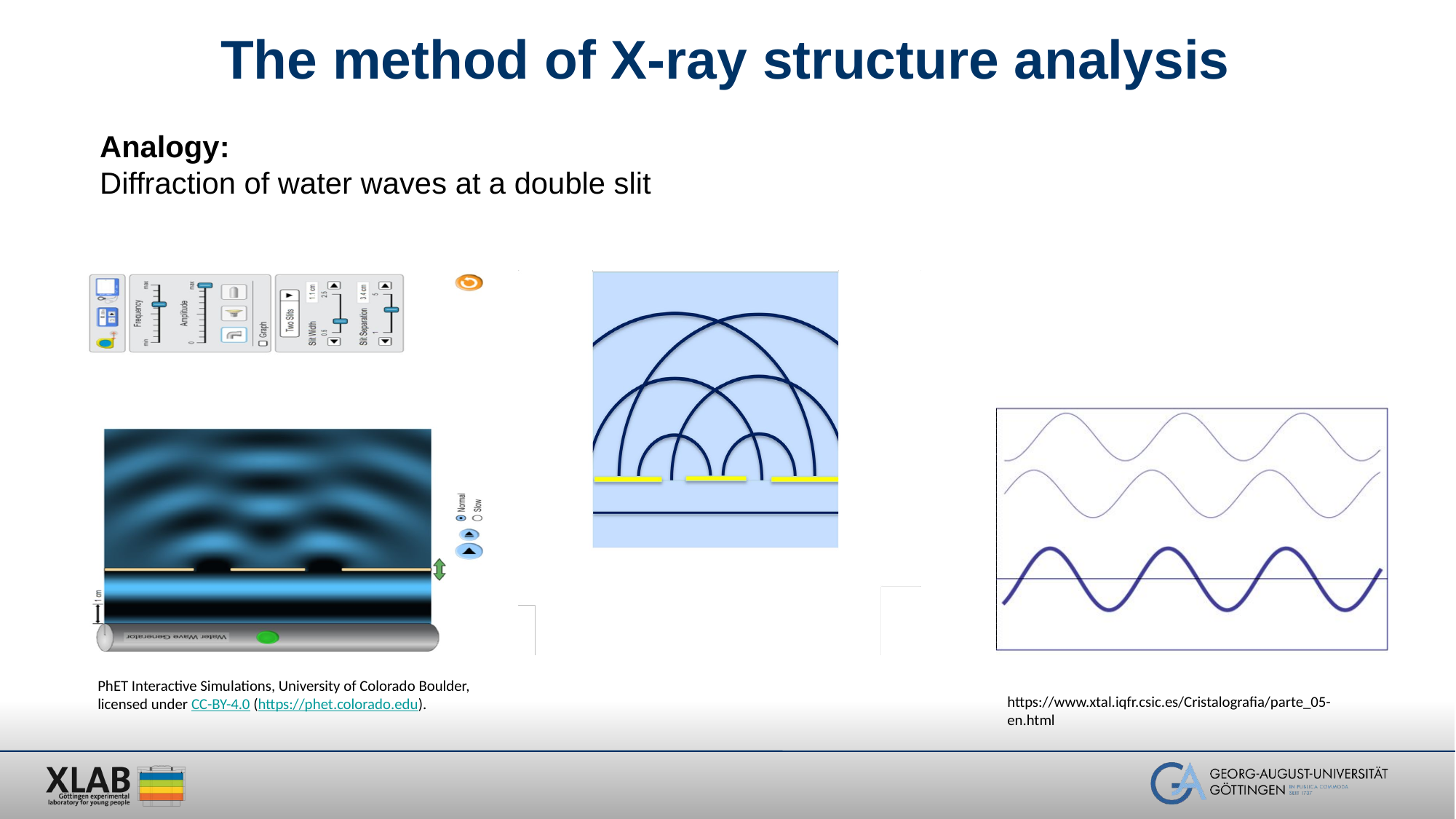

# The method of X-ray structure analysis
Analogy:
Diffraction of water waves at a double slit
PhET Interactive Simulations, University of Colorado Boulder, licensed under CC-BY-4.0 (https://phet.colorado.edu).
https://www.xtal.iqfr.csic.es/Cristalografia/parte_05-en.html

## Slide 4
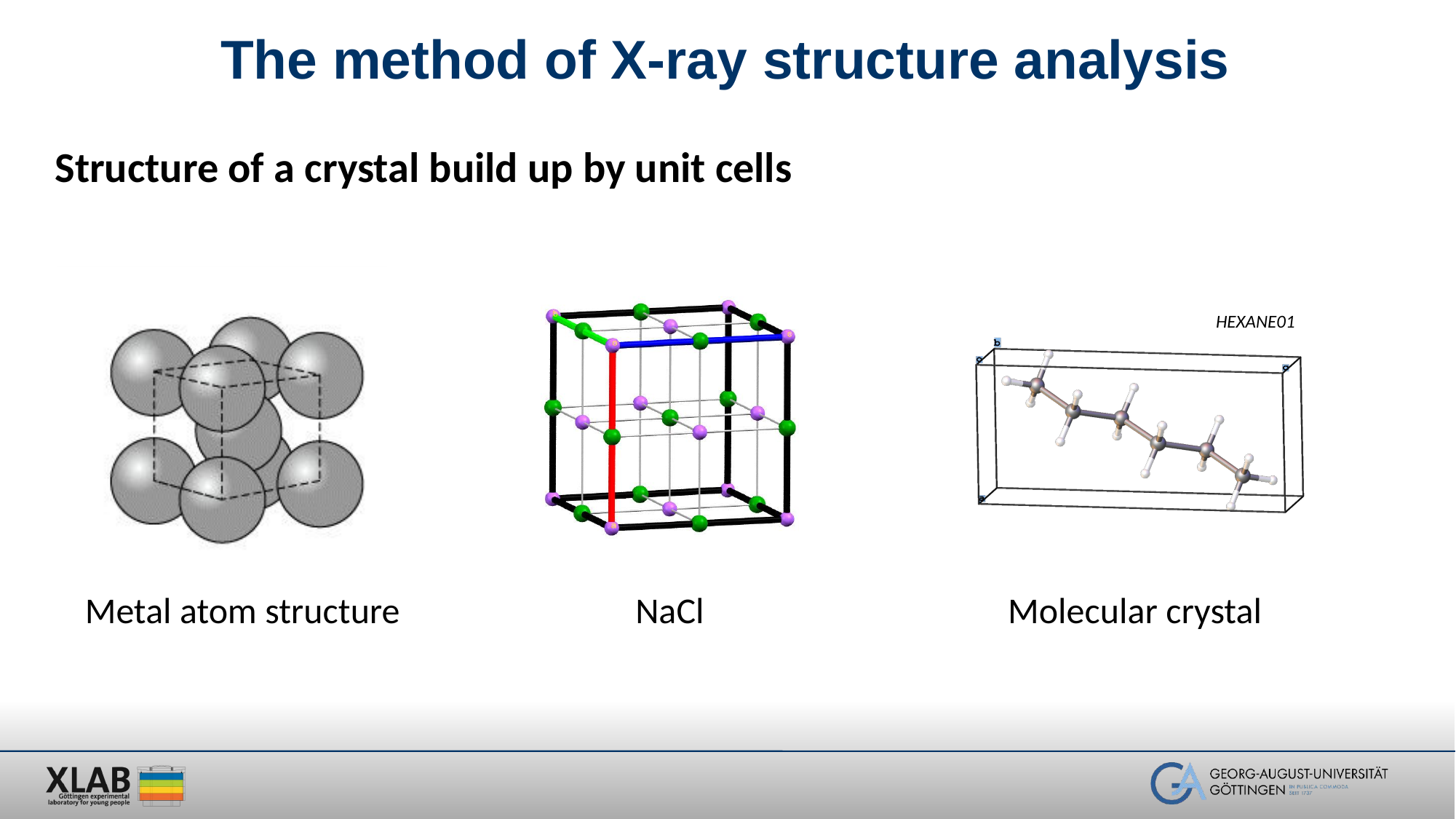

# The method of X-ray structure analysis
Structure of a crystal build up by unit cells
HEXANE01
Metal atom structure
NaCl
Molecular crystal

## Slide 5
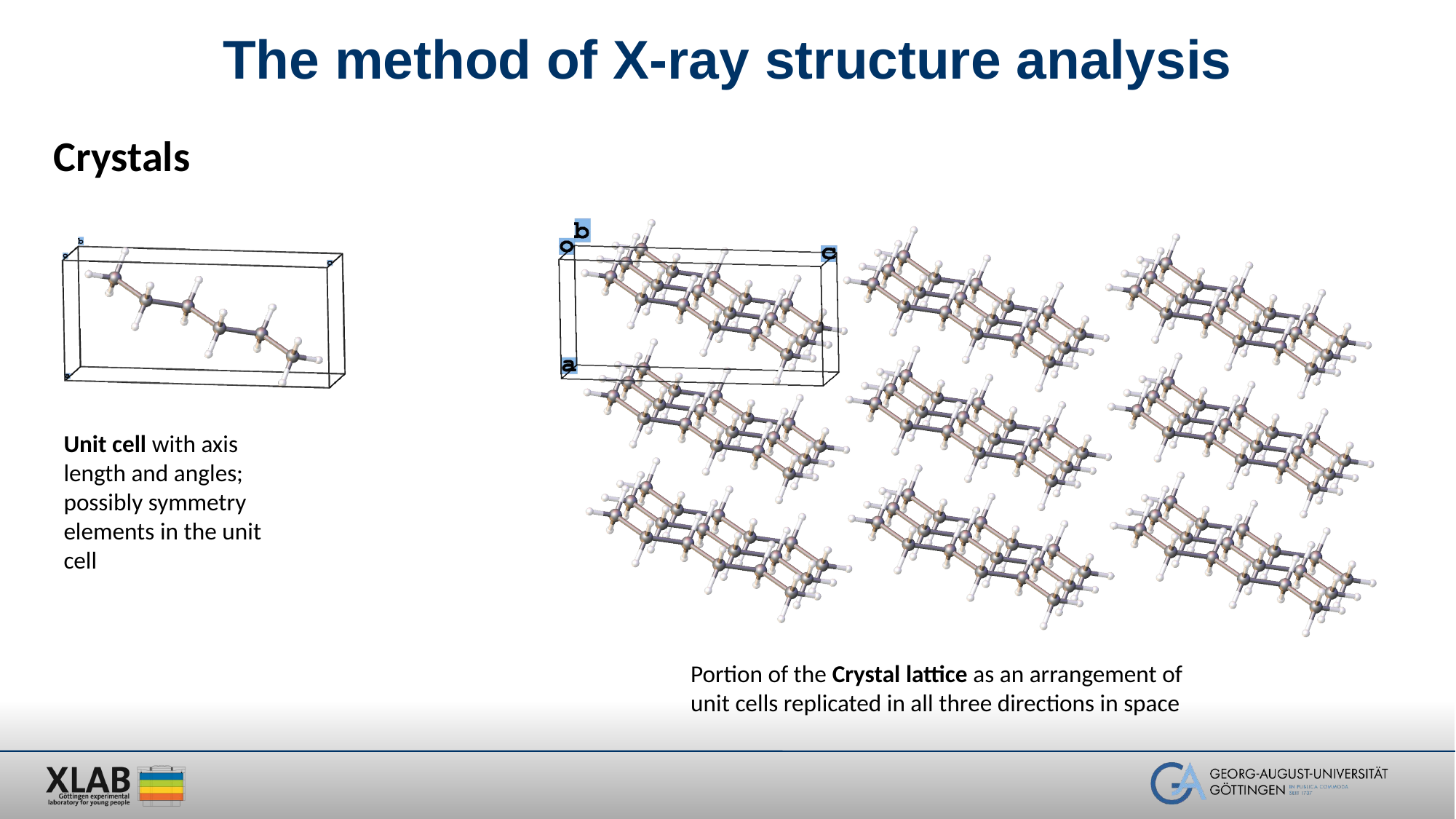

# The method of X-ray structure analysis
Crystals
Unit cell with axis length and angles; possibly symmetry elements in the unit cell
Portion of the Crystal lattice as an arrangement of unit cells replicated in all three directions in space

## Slide 6
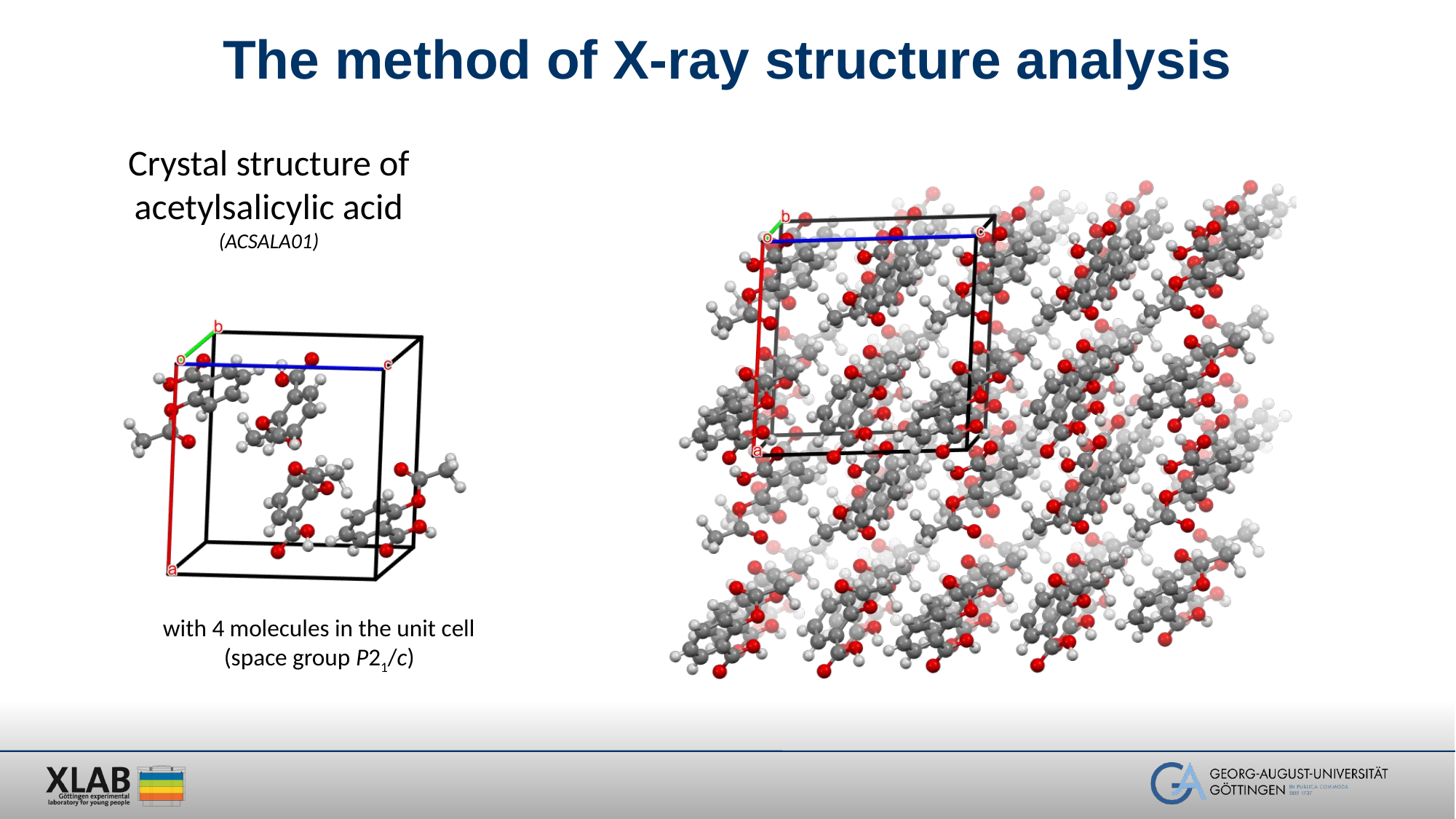

# The method of X-ray structure analysis
Crystal structure of acetylsalicylic acid
(ACSALA01)
with 4 molecules in the unit cell (space group P21/c)

## Slide 7
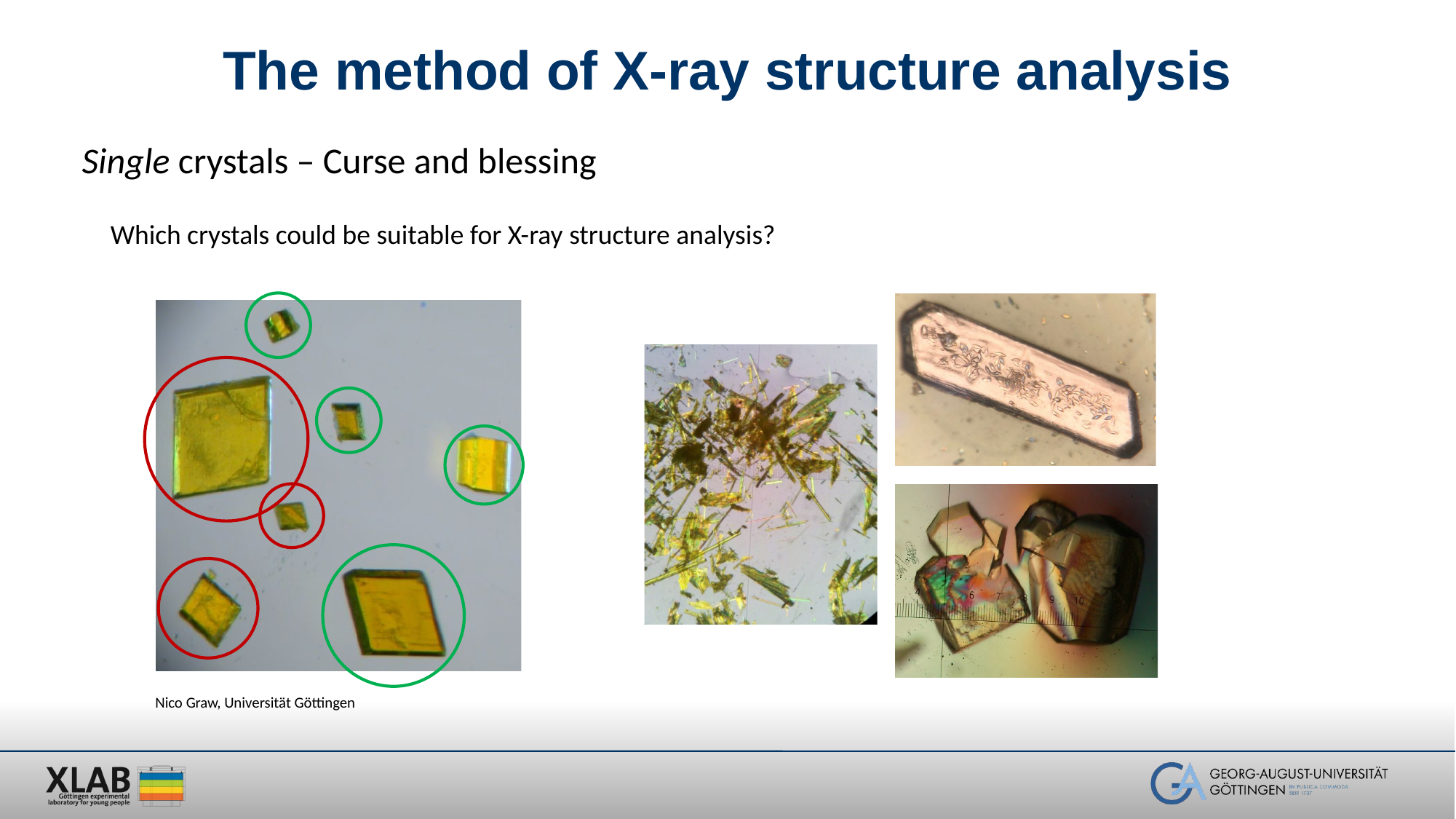

The method of X-ray structure analysis
Single crystals – Curse and blessing
Which crystals could be suitable for X-ray structure analysis?
Nico Graw, Universität Göttingen

## Slide 8
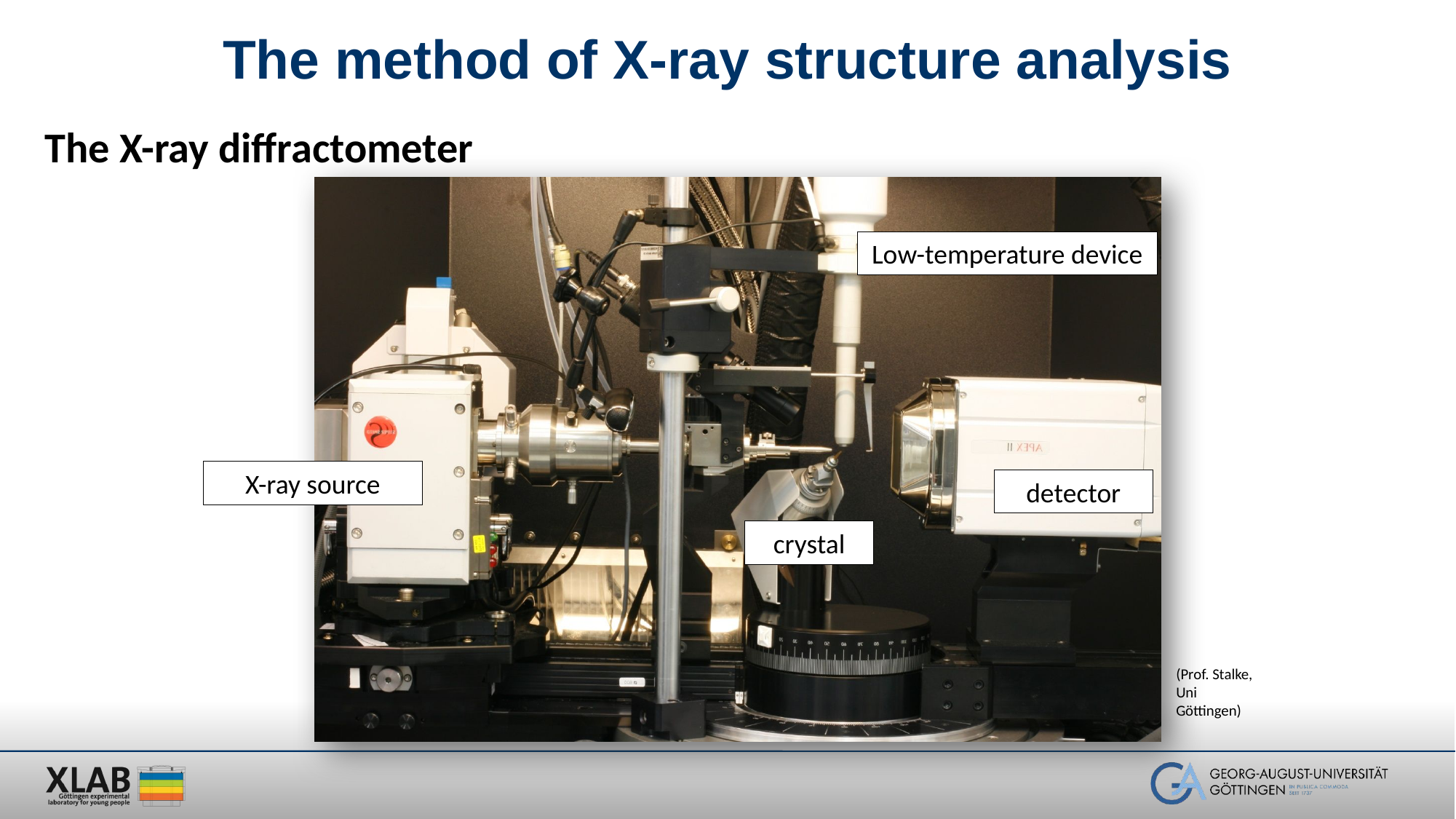

# The method of X-ray structure analysis
The X-ray diffractometer
Low-temperature device
X-ray source
detector
crystal
(Prof. Stalke, Uni Göttingen)

## Slide 9
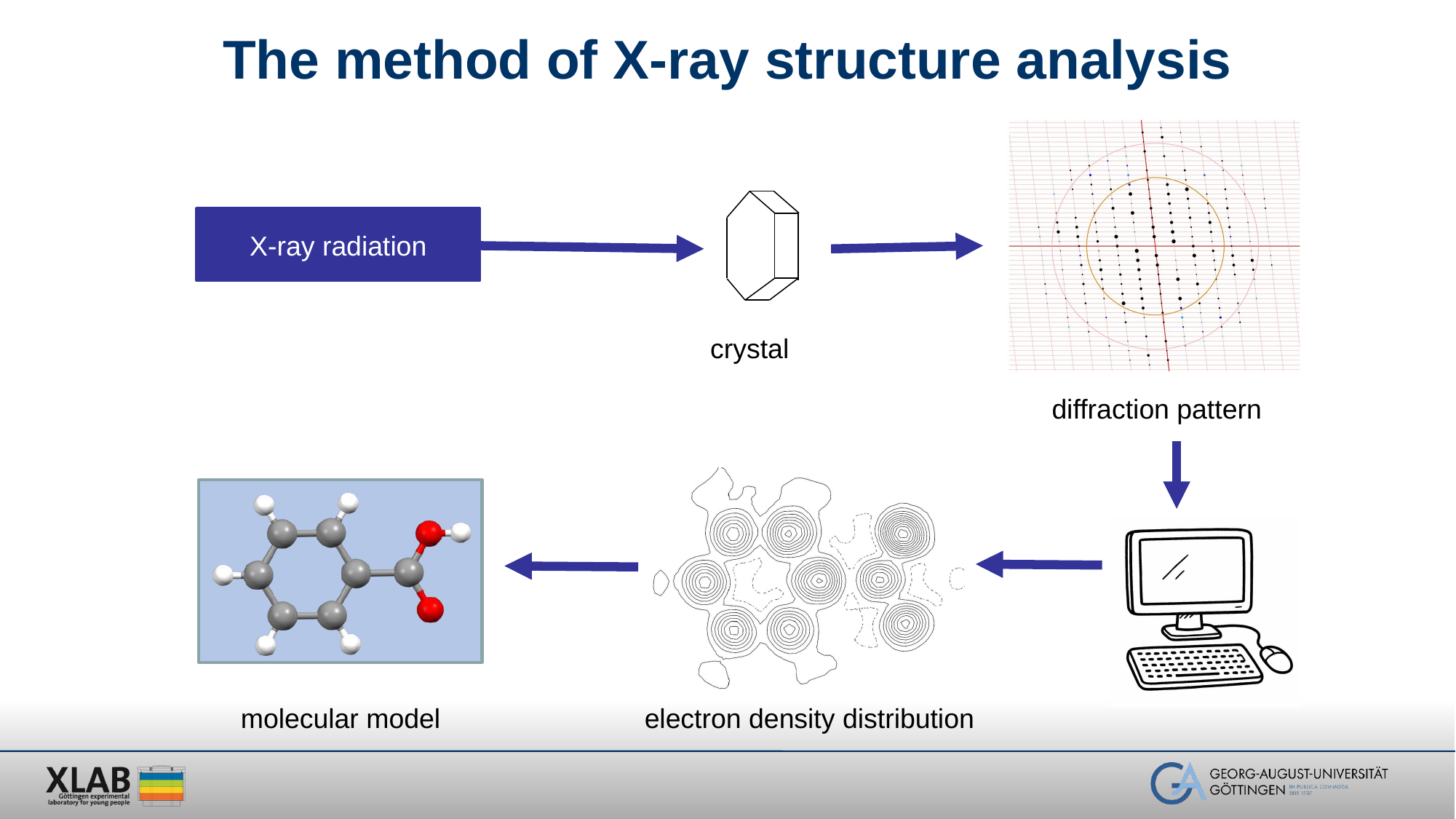

# The method of X-ray structure analysis
X-ray radiation
crystal
diffraction pattern
molecular model
electron density distribution

## Slide 10
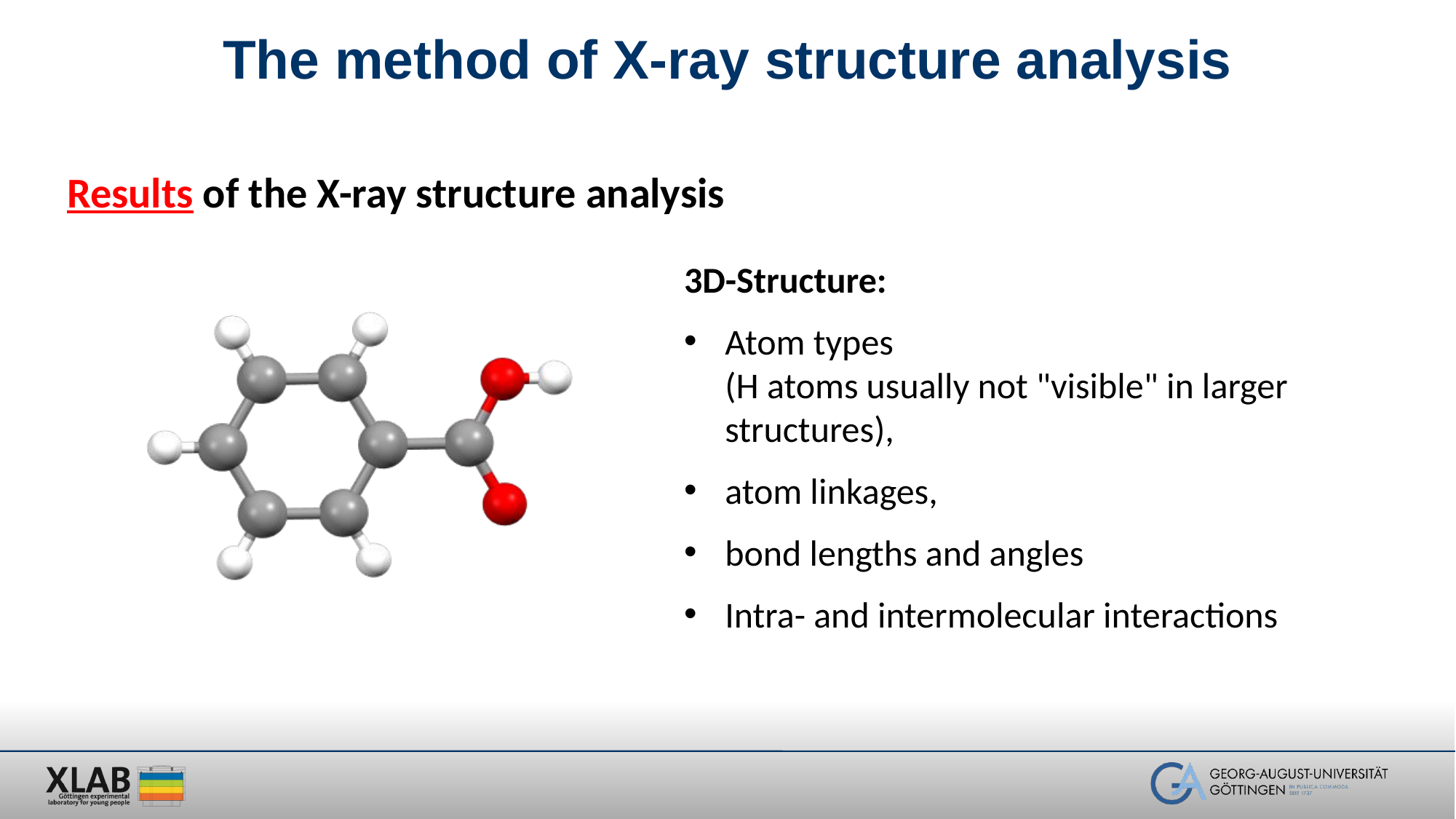

# The method of X-ray structure analysis
Results of the X-ray structure analysis
3D-Structure:
Atom types
(H atoms usually not "visible" in larger structures),
atom linkages,
bond lengths and angles
Intra- and intermolecular interactions
