# Supplementary material for: Crystallography in school
Source: J Appl Crystallogr. 2025 Sep 12;58(Pt 5):1802–9. doi: 10.1107/S1600576725007459 (PMC12502877; doi:10.1107/S1600576725007459)
Supplement: Supplementary file 5 [file j-58-01802-sup5.pptx]

## Slide 1
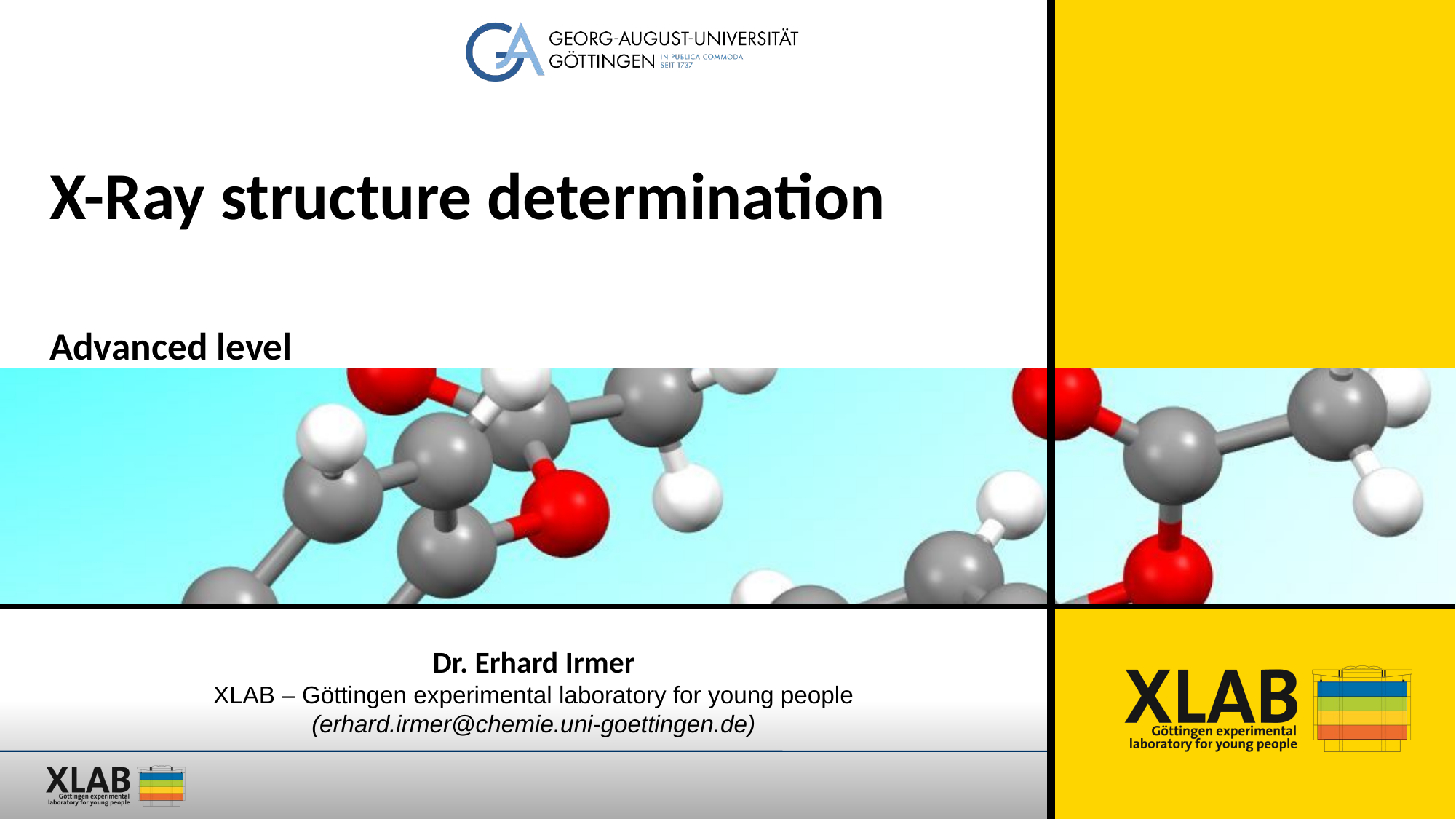

X-Ray structure determination
Advanced level
Dr. Erhard Irmer
XLAB – Göttingen experimental laboratory for young people
(erhard.irmer@chemie.uni-goettingen.de)

## Slide 2
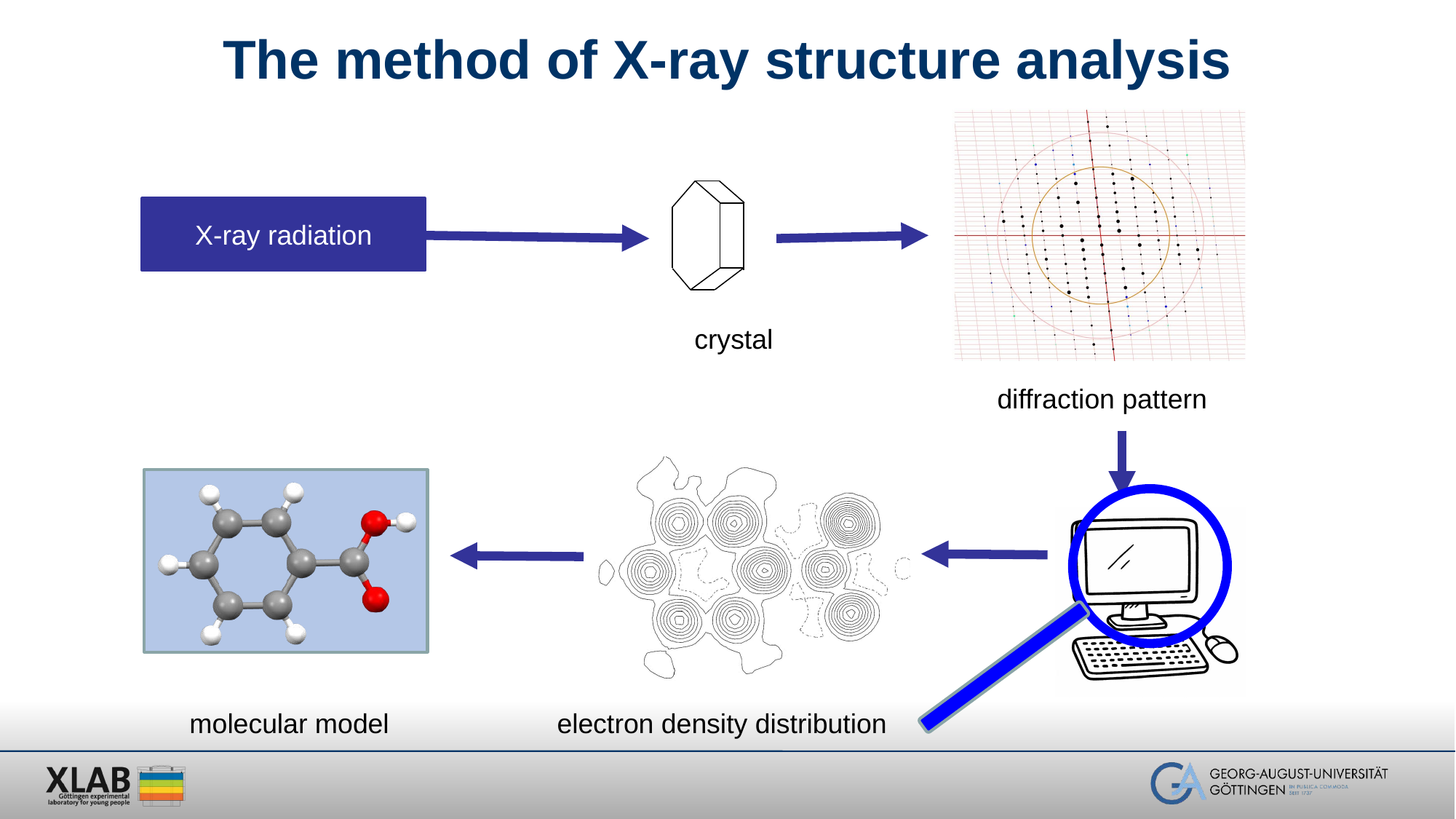

# The method of X-ray structure analysis
X-ray radiation
crystal
diffraction pattern
molecular model
electron density distribution

## Slide 3
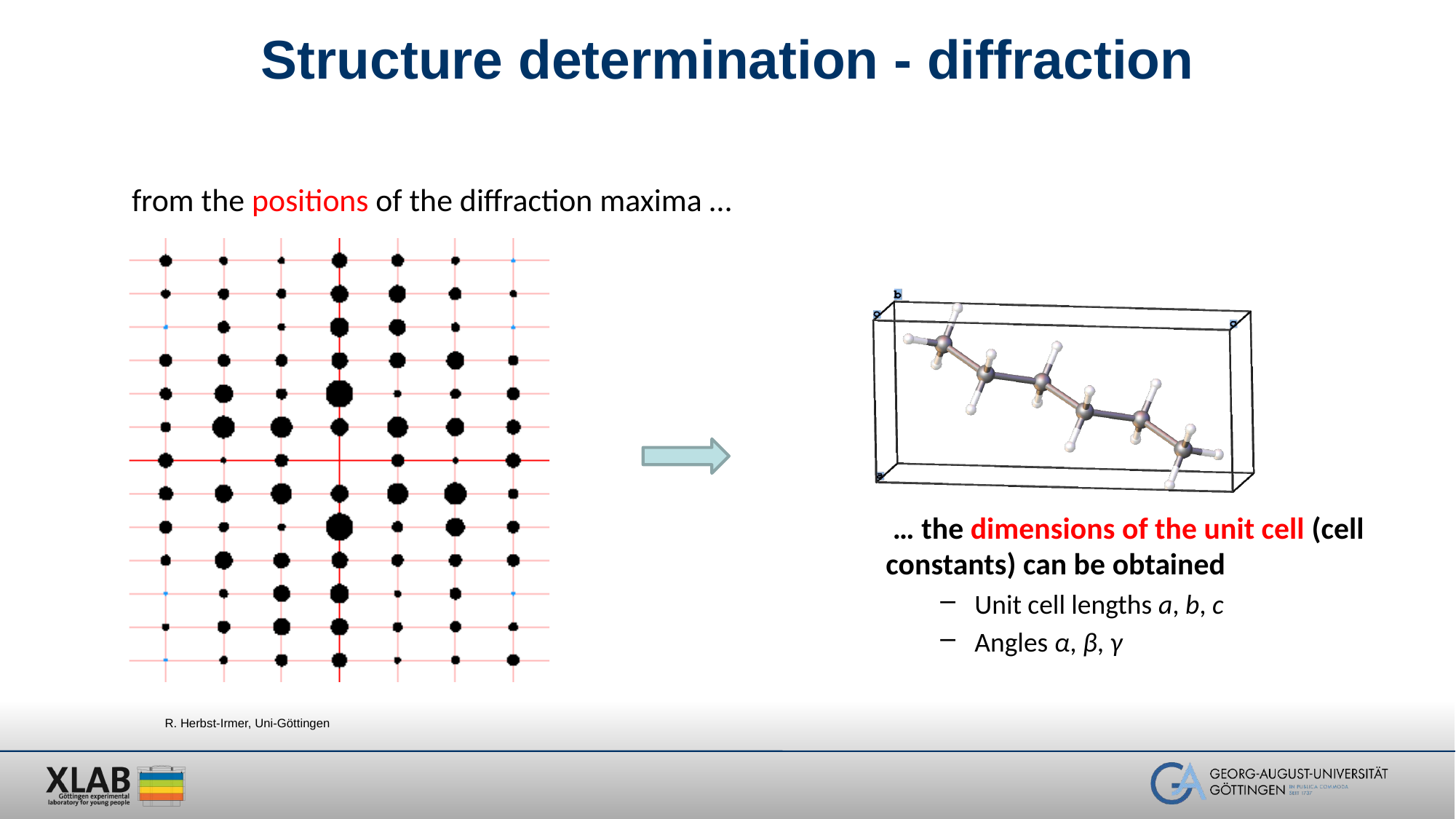

# Structure determination - diffraction
from the positions of the diffraction maxima …
 … the dimensions of the unit cell (cell constants) can be obtained
Unit cell lengths a, b, c
Angles α, β, γ
R. Herbst-Irmer, Uni-Göttingen

## Slide 4
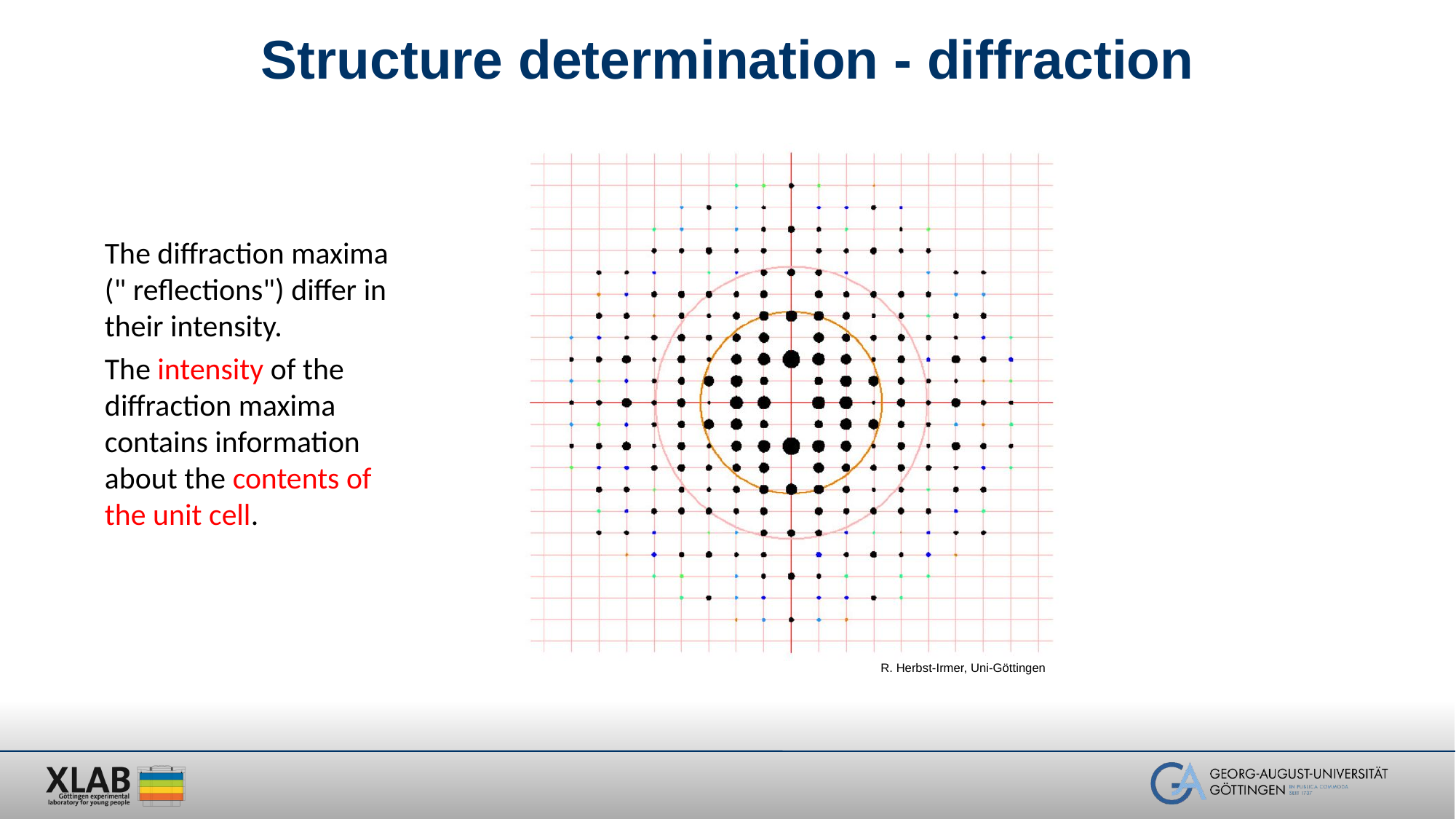

# Structure determination - diffraction
The diffraction maxima (" reflections") differ in their intensity.
The intensity of the diffraction maxima contains information about the contents of the unit cell.
R. Herbst-Irmer, Uni-Göttingen

## Slide 5
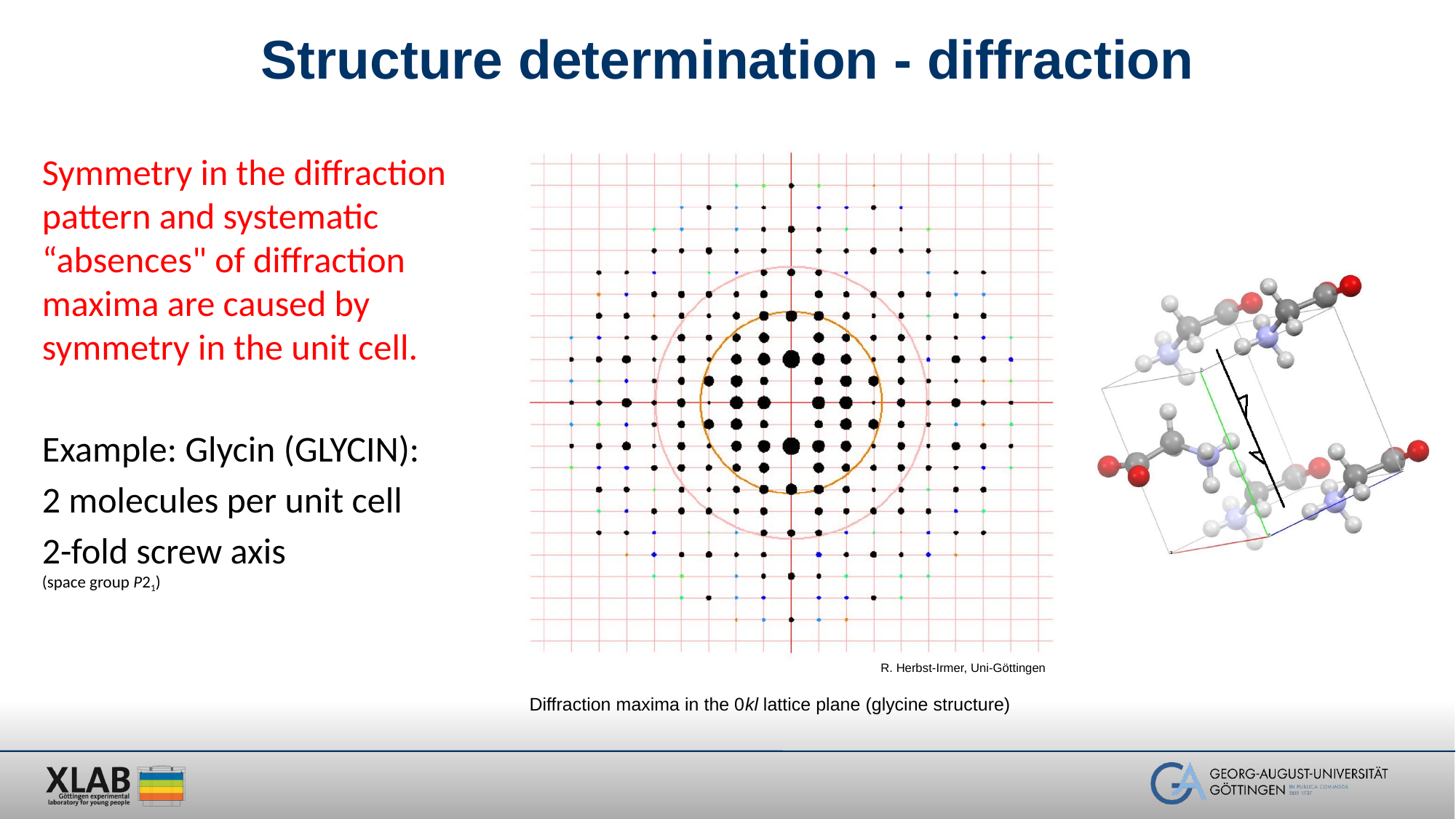

# Structure determination - diffraction
Symmetry in the diffraction pattern and systematic “absences" of diffraction maxima are caused by symmetry in the unit cell.
Example: Glycin (GLYCIN):
2 molecules per unit cell
2-fold screw axis
(space group P21)
R. Herbst-Irmer, Uni-Göttingen
Diffraction maxima in the 0kl lattice plane (glycine structure)

## Slide 6
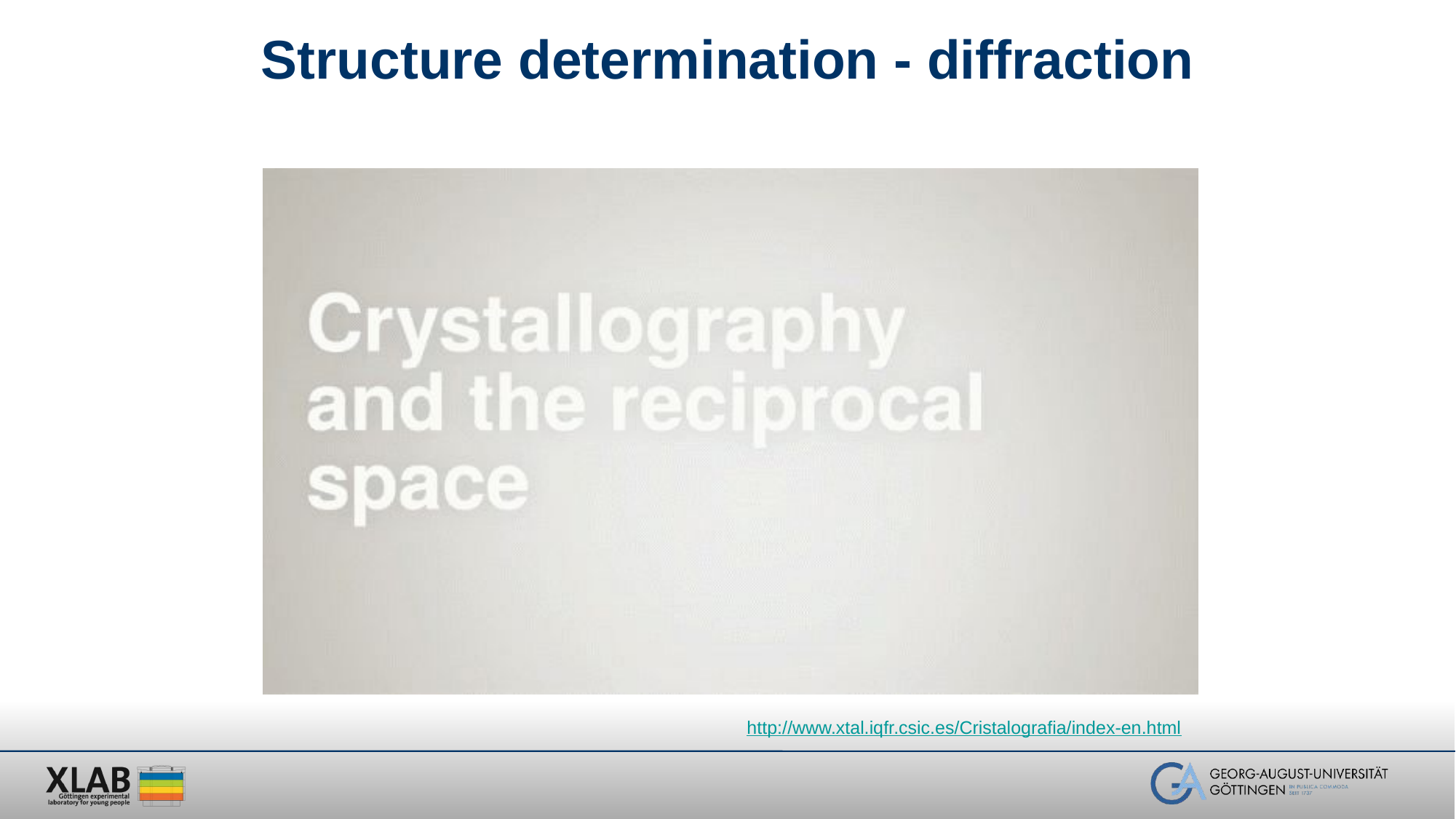

# Structure determination - diffraction
http://www.xtal.iqfr.csic.es/Cristalografia/index-en.html

## Slide 7
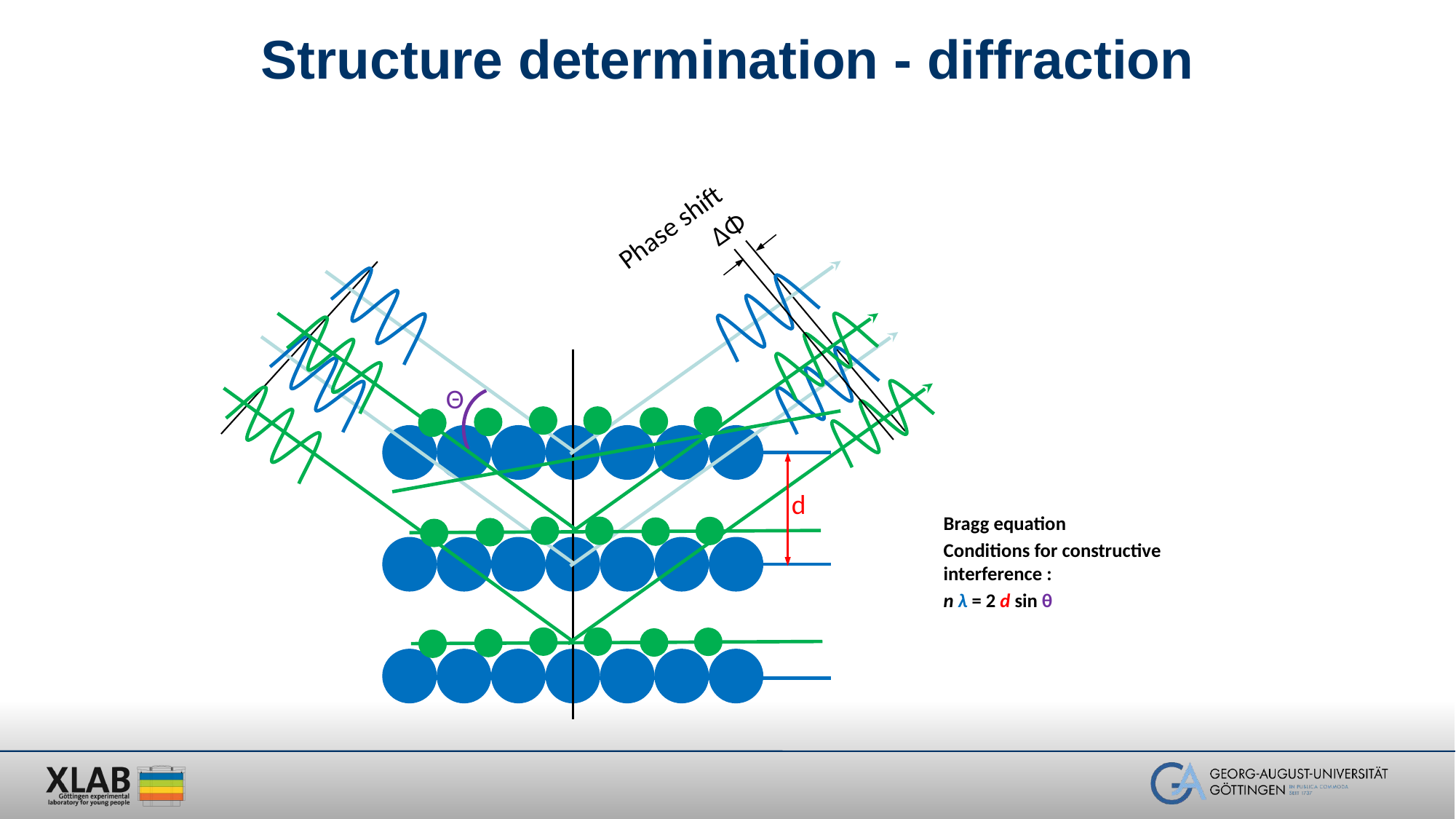

# Structure determination - diffraction
Phase shift
ΔΦ
Θ
d

## Slide 8
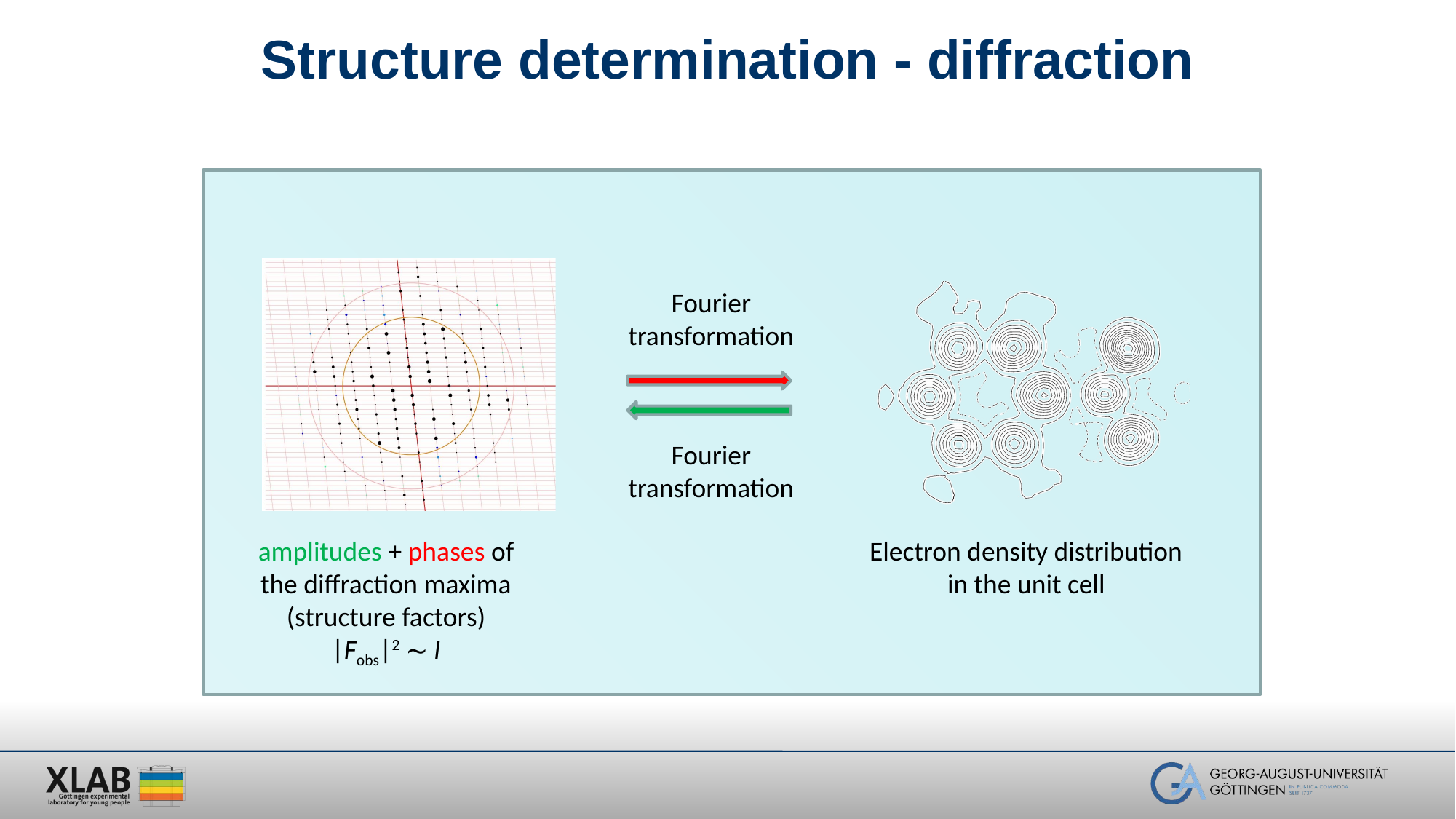

# Structure determination - diffraction
Fourier transformation
Fourier transformation
Electron density distribution in the unit cell
amplitudes + phases of the diffraction maxima
(structure factors)
|Fobs|2 ∼ I

## Slide 9
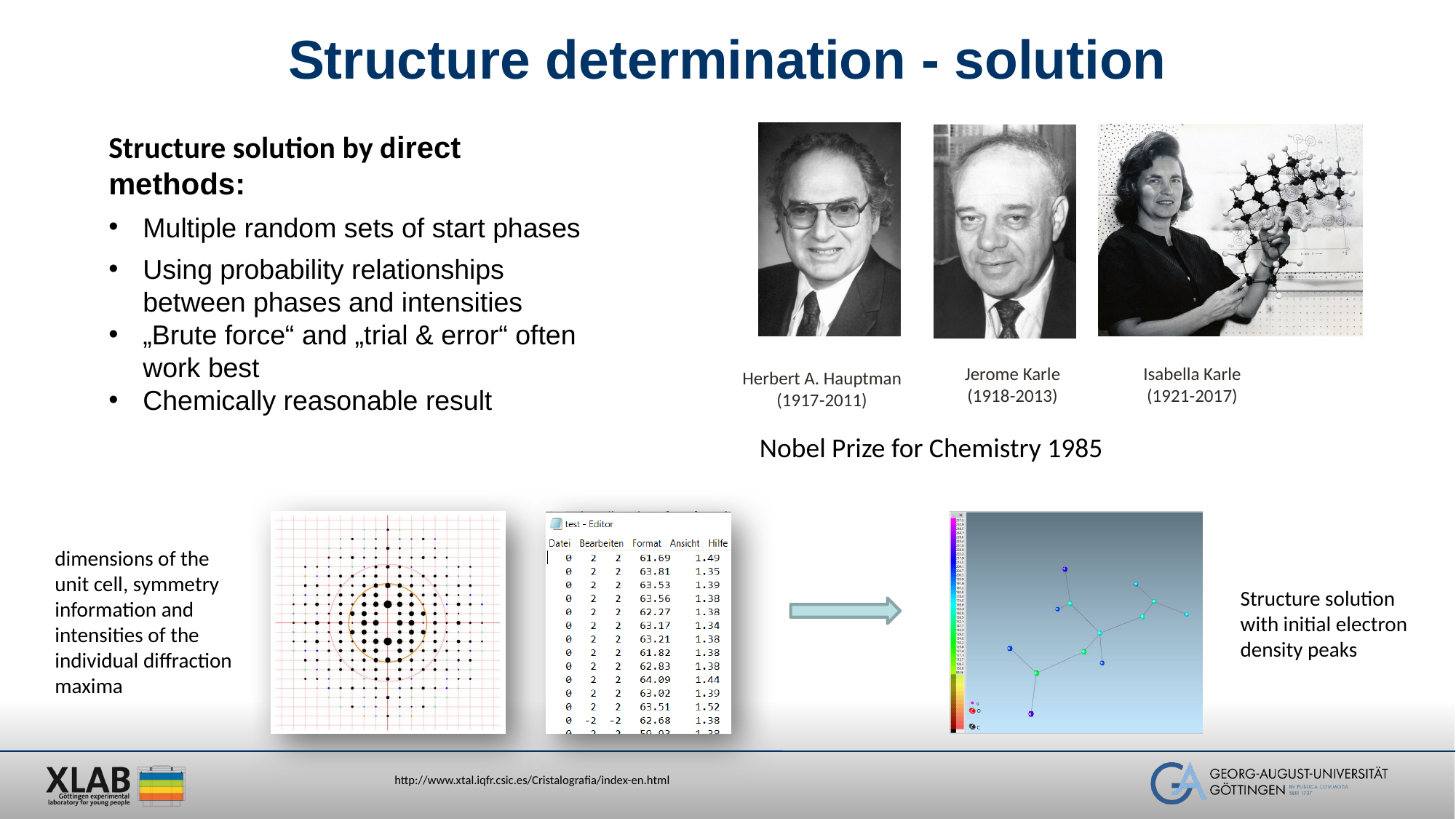

# Structure determination - solution
Structure solution by direct methods:
Multiple random sets of start phases
Using probability relationships between phases and intensities
„Brute force“ and „trial & error“ often work best
Chemically reasonable result
Isabella Karle (1921-2017)
Jerome Karle
(1918-2013)
Herbert A. Hauptman
(1917-2011)
Nobel Prize for Chemistry 1985
dimensions of the unit cell, symmetry information and intensities of the individual diffraction maxima
Structure solution with initial electron density peaks
http://www.xtal.iqfr.csic.es/Cristalografia/index-en.html

## Slide 10
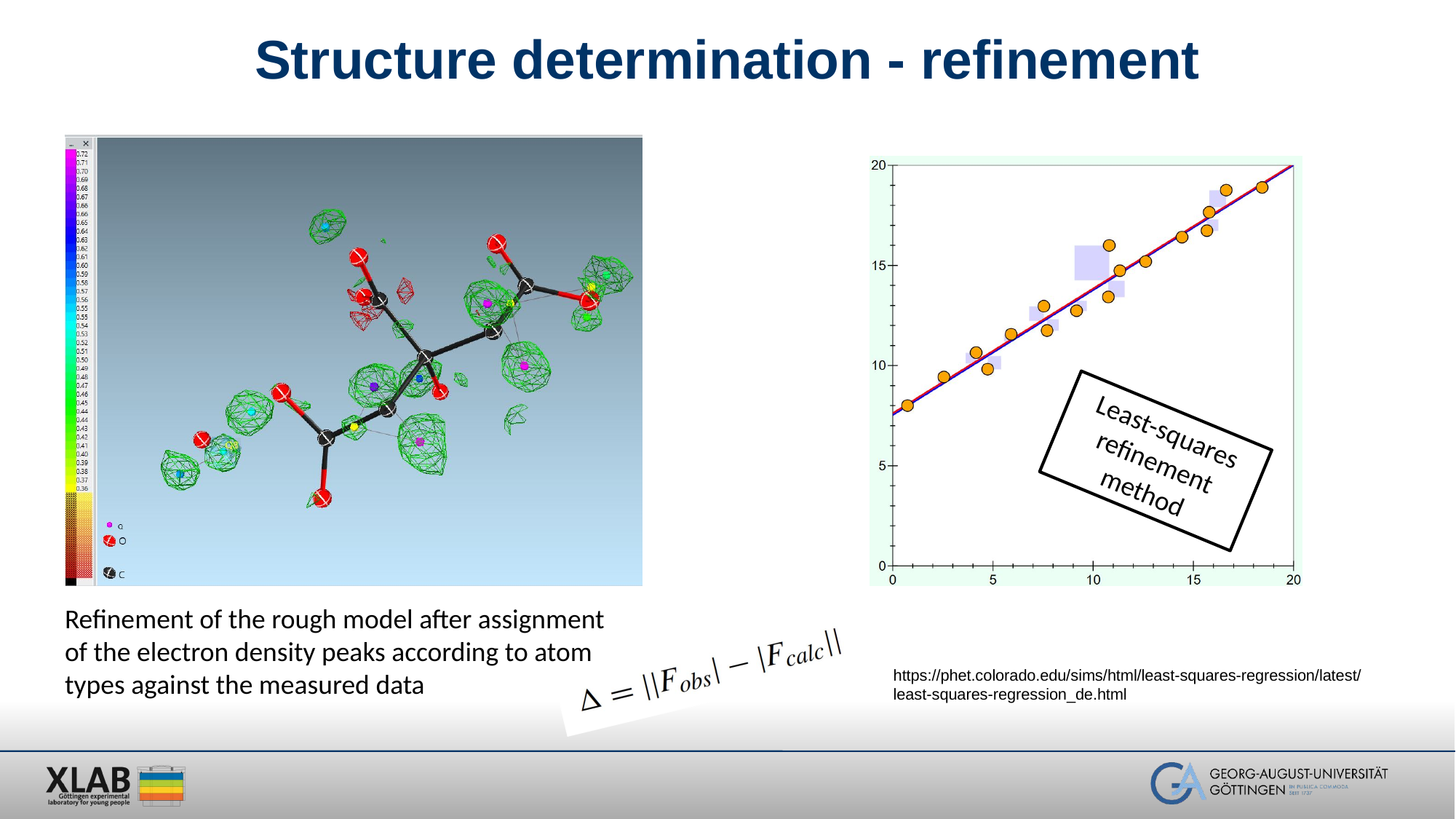

# Structure determination - refinement
Least-squares refinement method
Refinement of the rough model after assignment of the electron density peaks according to atom types against the measured data
https://phet.colorado.edu/sims/html/least-squares-regression/latest/least-squares-regression_de.html

## Slide 11
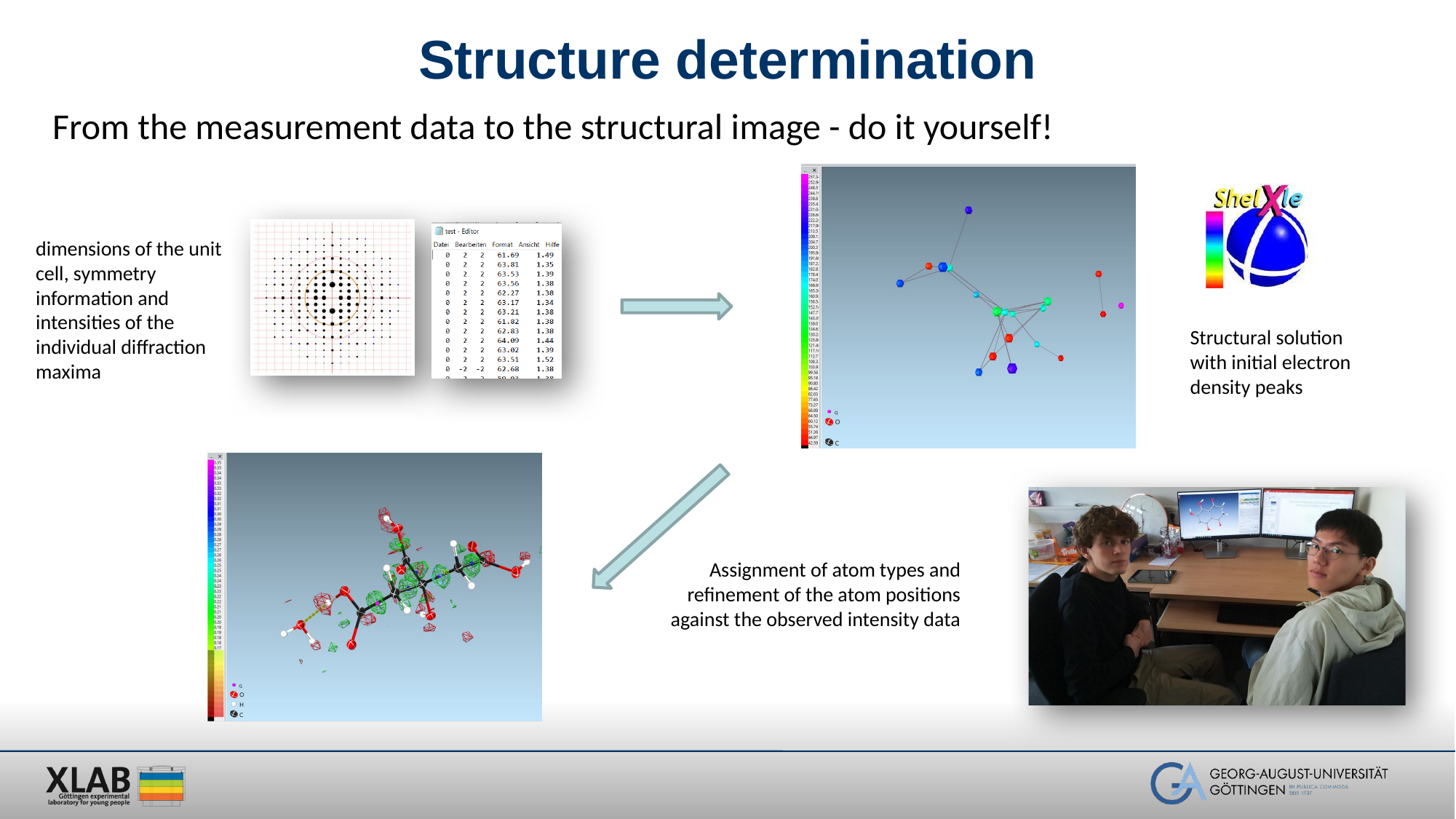

# Structure determination
From the measurement data to the structural image - do it yourself!
dimensions of the unit cell, symmetry information and intensities of the individual diffraction maxima
Structural solution with initial electron density peaks
Assignment of atom types and refinement of the atom positions against the observed intensity data
