# Supplementary material for: Crystallography in school
Source: J Appl Crystallogr. 2025 Sep 12;58(Pt 5):1802–9. doi: 10.1107/S1600576725007459 (PMC12502877; doi:10.1107/S1600576725007459)
Supplement: Supplementary file 6 [file j-58-01802-sup6.pdf]

GEORG-AUGUST-UNIVERSITÄT
GÖTTINGEN IN PUBLICA COMMODA SEIT 1737

Step-by-step guide for the structure determination with Olex2

Aspirin

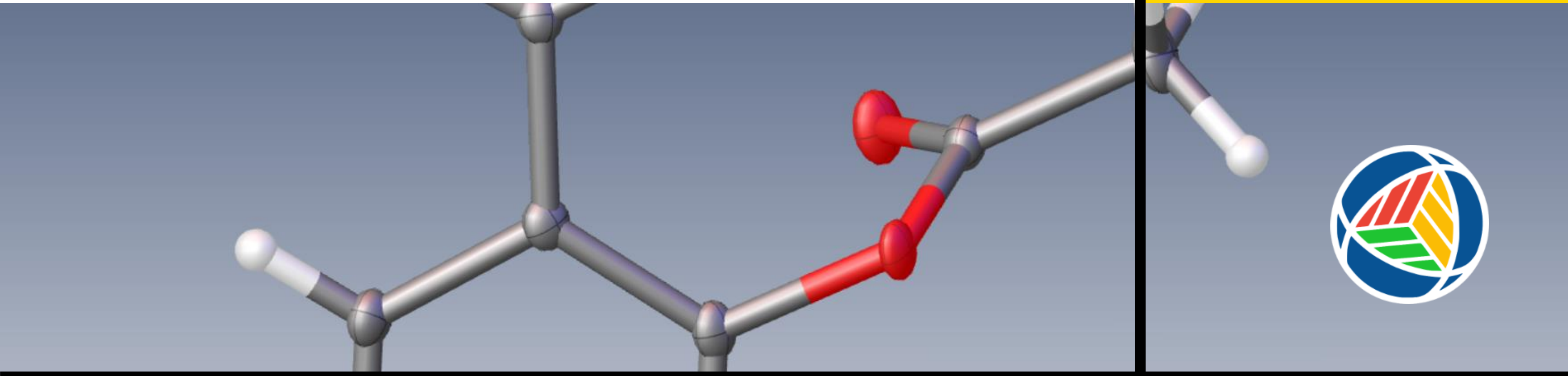

Erhard Irmer

XLAB Göttinger Experimentallabor für junge Leute

XLAB Göttinger Experimentallabor für junge Leute

Übersicht

- Starting the program
- Introduction
- Structure solution
- The controls
- Refinement
- Labelling of the atoms
- Adding hydrogen atoms
- The finishing touch
- Analysis of the structure
- Creating images

Starting the program

Double-click to open the “Olex2 " program on the computer desktop.

The programme interface opens with a preset structure (that of sucrose).

To load our tutorial structure, click on ‘Open’ on the right-hand side of the screen and then navigate to the ‘aspirin.ins’ file, which is located in the ‘Xray - Tutorial’ subfolder on the computer's desktop.

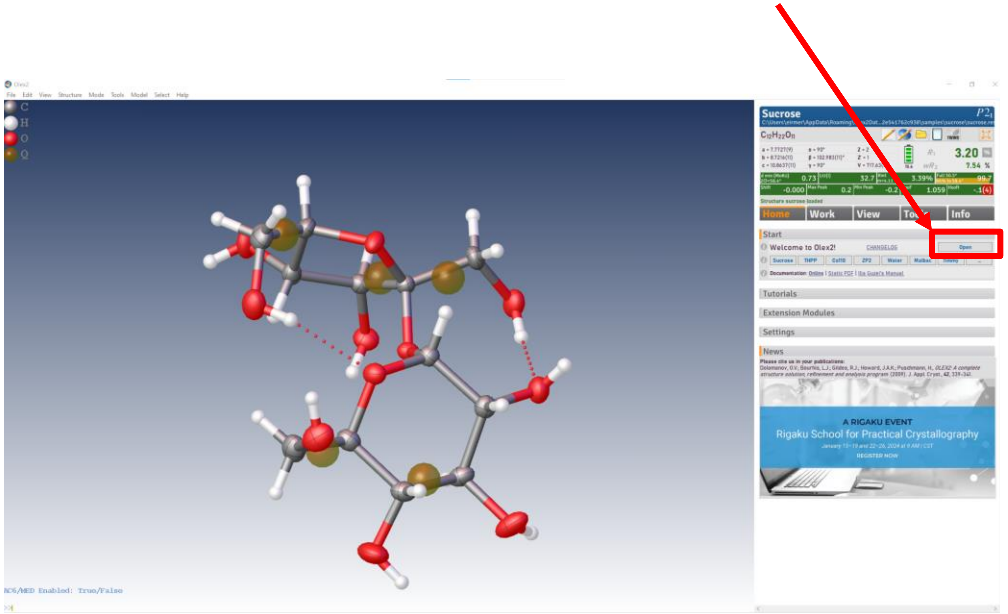

Introduction

Structure:

Olex2 essentially consists of two areas. A window on the left in which the structural model is displayed and a work window on the right in which most of the processing steps are carried out.

There are several main tabs in the right-hand window. The one that is currently open appears in orange. The 'Home' tab contains various settings and tutorials for Olex2. The most important tab for us is the "Work" tab to the right of the 'Home' tab.

The structural model is displayed here.

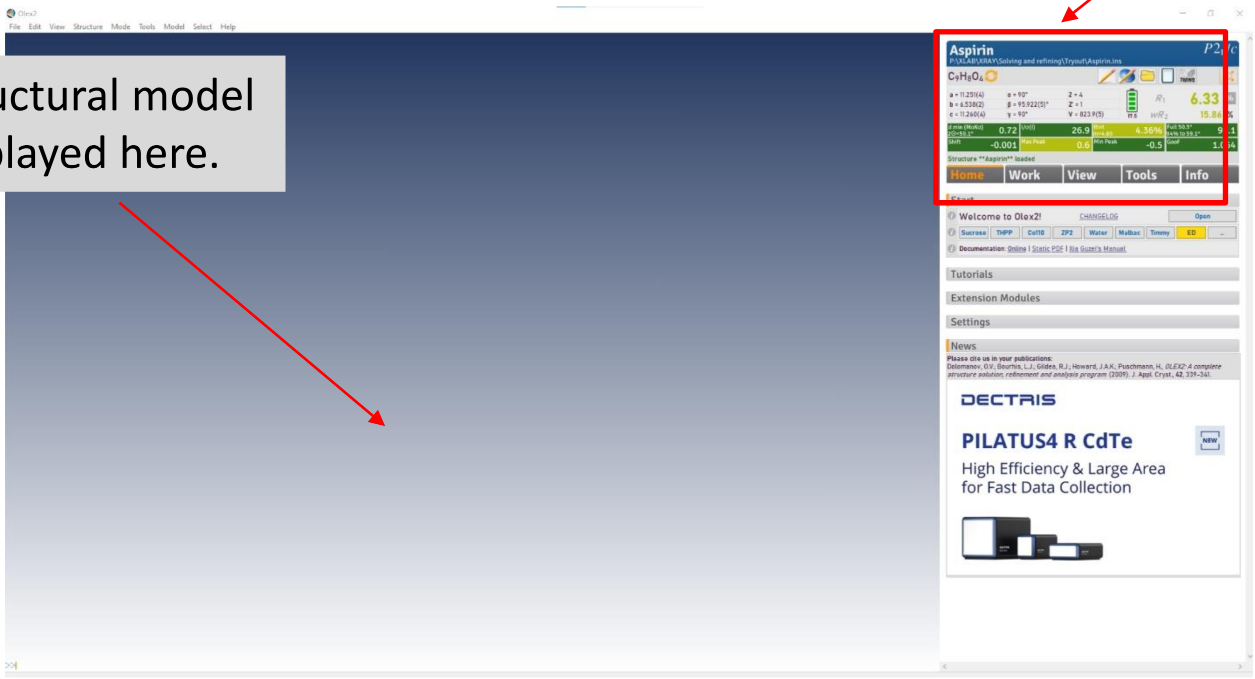

Structure solution

The „Work“-Tab:

The ‘Work’ tab has four sub-tabs ‘Solve’, ‘Refine’, “Draw” and ‘Report’. The tab options can be opened by clicking on the ‘down arrow’ to the right of their name. The rest of the button executes the respective action. ‘Solve’ searches for a structural solution, ‘Refine’ carries out the refinement, “Draw” provides options for visualisation and analysis and ‘Report’ is used to prepare the data for publication.Click on the arrow next to ‘Solve’.

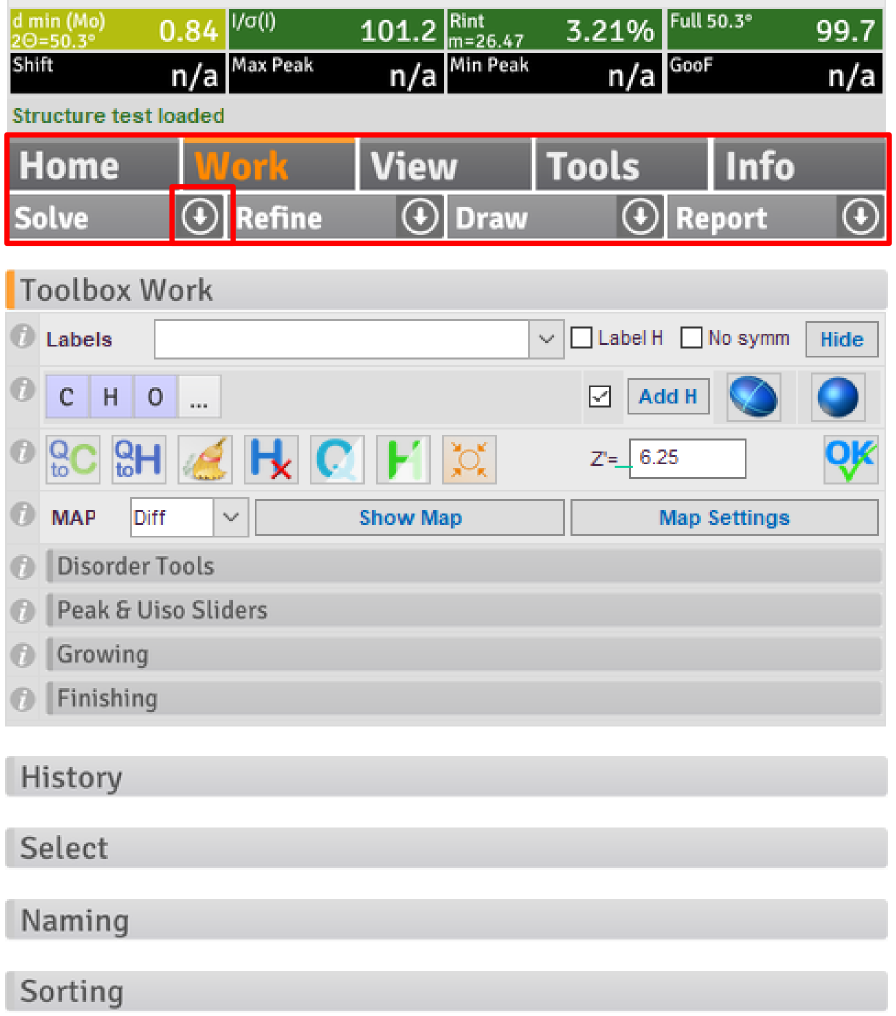

Structure solution

Settings for the structure solution:
Check whether the programme settings match the settings here. In particular, the Aspirin.hkl file must be selected for ‘Reflections’. In ‘Composition’ we could also change the suggested molecular formula to the actual molecular formula C9H8O4, but this is not absolutely necessary. A left click on ‘Solve’ performs the structure solution with the selected settings.

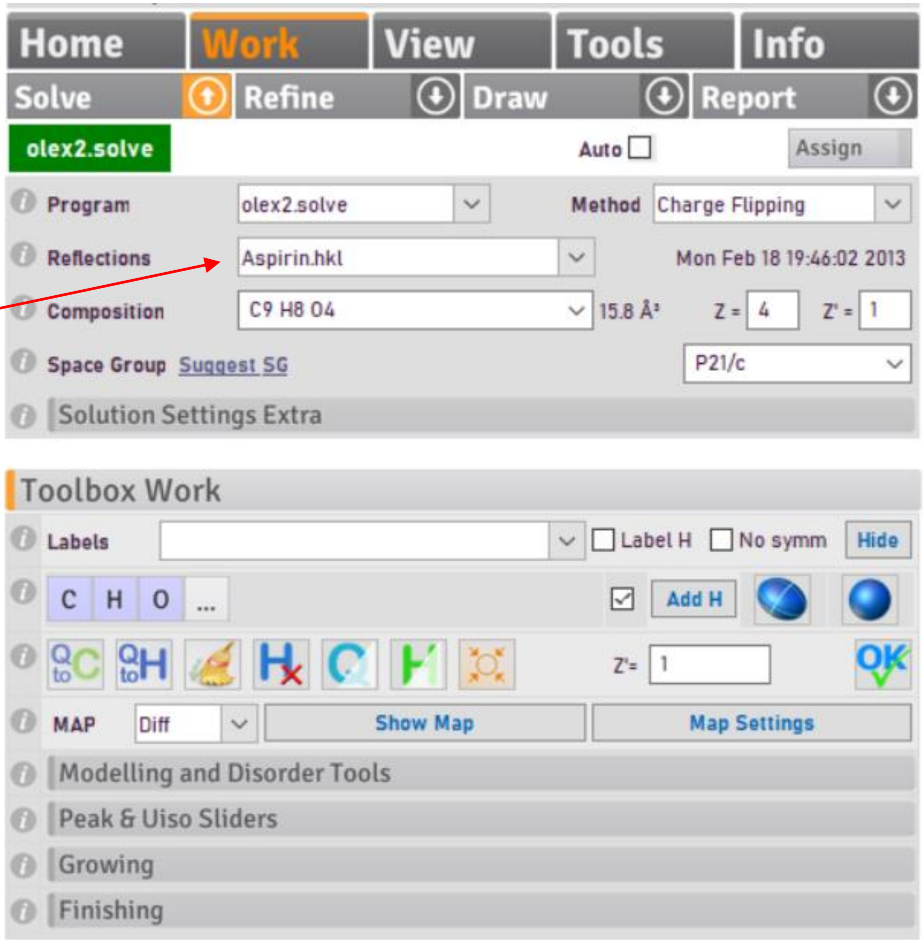

Structure solution

Structure solution:
Some golden spheres now appear in the left-hand window. These are so-called Q-peaks, i.e. places where there is a high electron density based on the data and therefore potential atom positions. To assemble a molecule from these fragments, we now click on the 'Center' button. If the result is not yet clearly visible, press 'Center' several times.

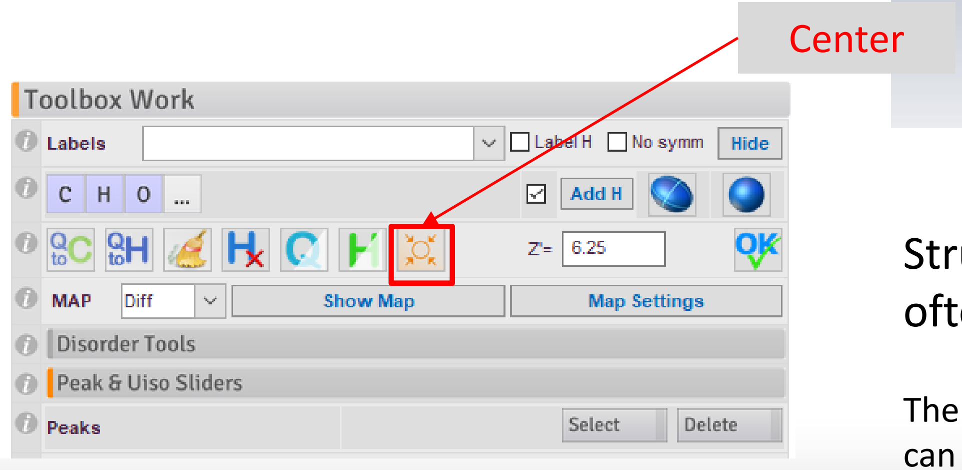

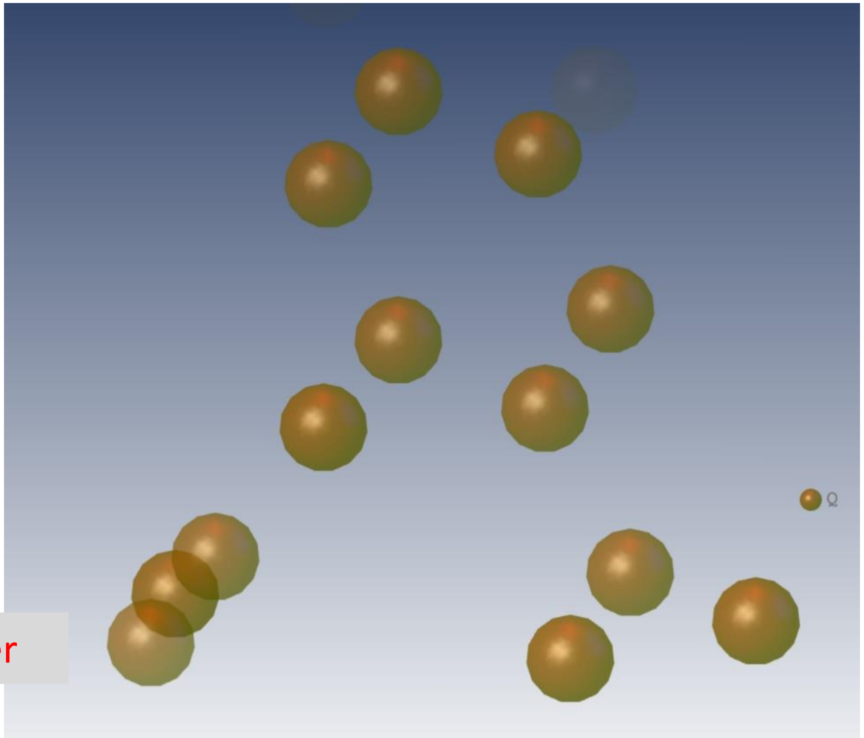

Structures, such as six rings, are now often already recognisable.

The text in the background of the molecule image can be hidden with 'Ctrl-T'.

The controls

The Q peaks can be displayed or hidden step by step by turning the mouse wheel.

The molecule is rotated by moving the mouse while holding down the left mouse button.

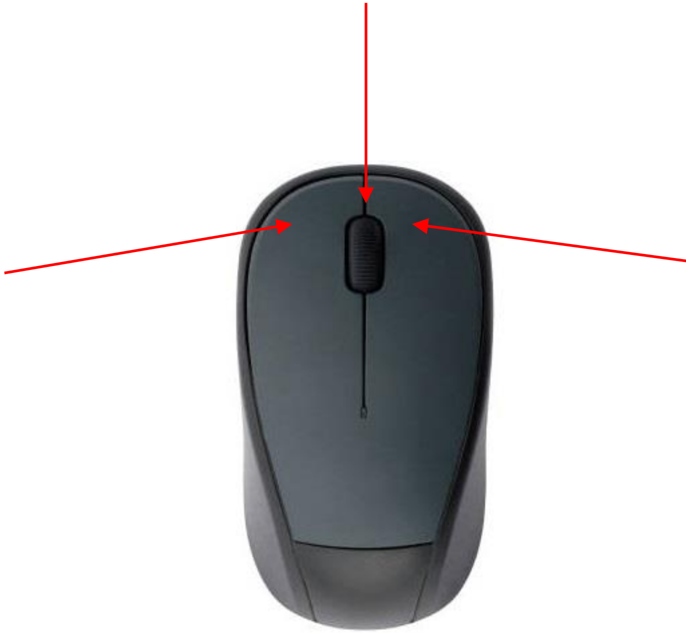

The display can be zoomed by moving the mouse while holding down the right mouse button.

Try this out until you have developed a feel for it.

Structure solution

Assigning of the Q peaks to atoms:
Now chemistry comes into play!
Think about which atom types could be hidden behind the Q peaks.
You can make the interpretation a little easier by displaying ‘bonds’ between the Q peaks. To do this, press the Q button in the ‘Toolbox Work’ repeatedly until the connecting lines appear.

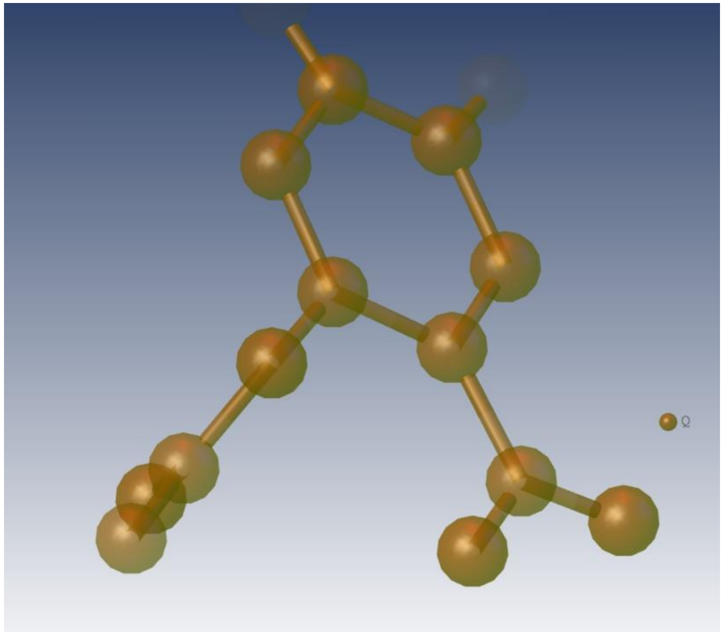

Probable gross formula of the compound:
$C_9H_8O_4$

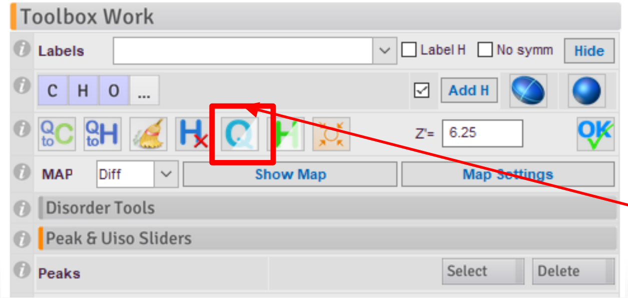

Q-Peaks
(display / display with bonds / hide)

Structure solution

Assigning of the Q peaks to atoms:
You have probably been able to quickly assign the six-membered ring to carbon atoms. By clicking on ‘C’ in the Work toolbox and then clicking on the Q peaks to be assigned, these are marked as carbon atoms.

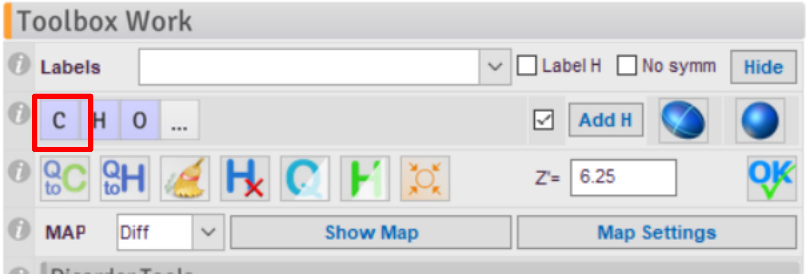

Some Q peaks with low electron density do not make sense chemically. We can hide them by turning the mouse wheel.

However, it is often not known what the exact composition of the compound is. In this case, you could start by labelling all meaningful Q peaks of an organic compound as C atoms. Let's proceed in this way. Mark the remaining Q peaks as C atoms as well.

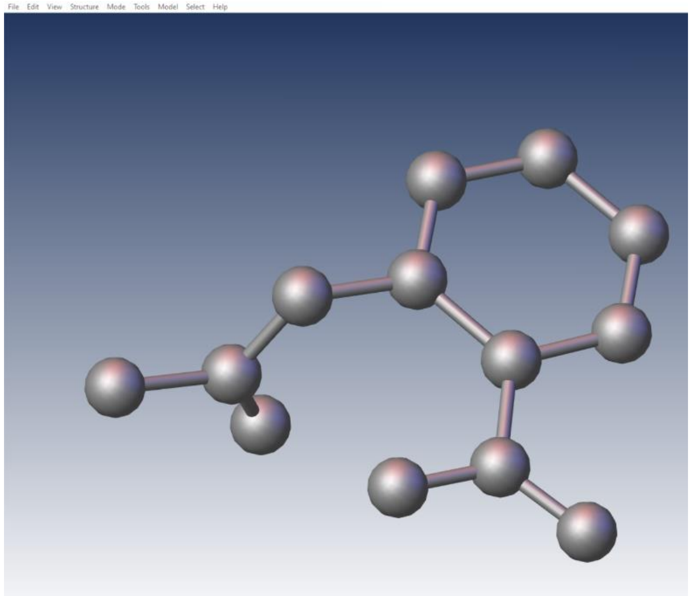

Refinement

Settings for the refinement:
In the refinement, we adapt our rough structural model to the measured data.

Please open the settings for refinement by clicking on the arrow next to ‘Refine’ and check that the settings match those on the right.

Under ‘Cycles’ you specify how many least squares refinement cycles the programme should perform (at least 4); the maximum number of Q peaks to be displayed should be set to at least 11.

Now start the refinement by clicking on the ‘Refine’ button.

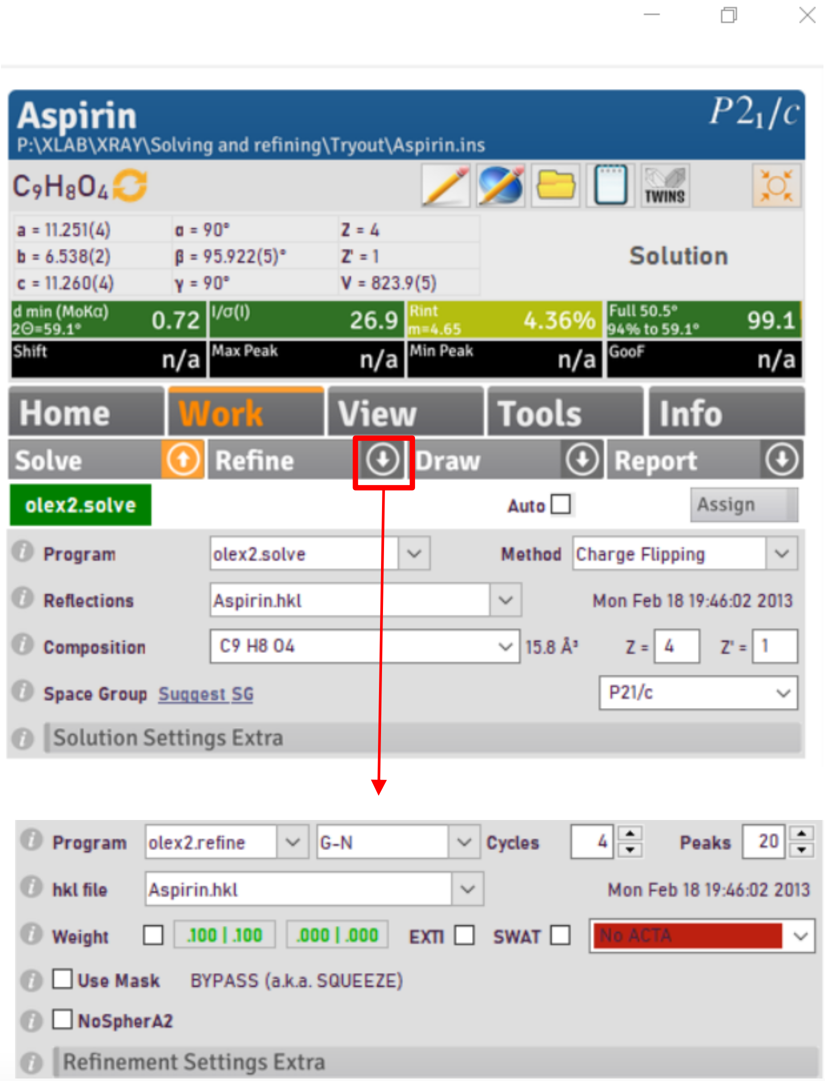

Refinement

First refinement:
Now we check whether the specified structural model (in our case all atoms carbon) matches the measured intensity data from the diffraction measurement.

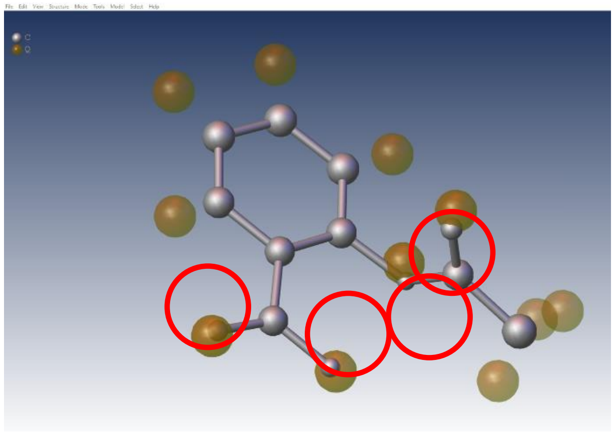

A new structure image appears in the left-hand window.We see a new Q-peak in the immediate neighbourhood of some atoms.

We can also have a look at the residual electron density map. Hide the Q peaks by turning the mouse wheel. Then click on 'Show Map'. Now places where there is more electron density than in the model based on the measurement data are shown with a green grid structure.

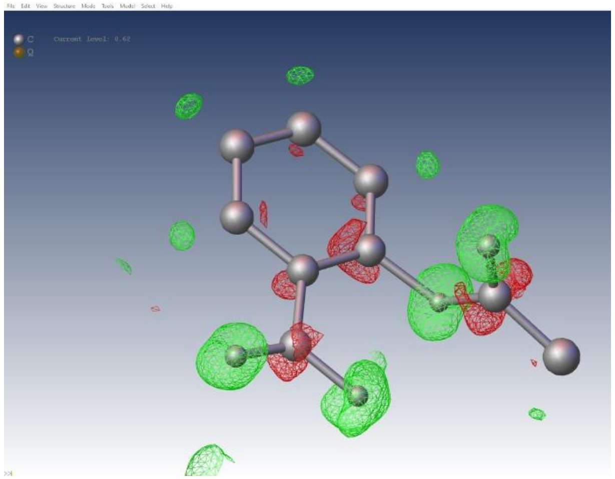

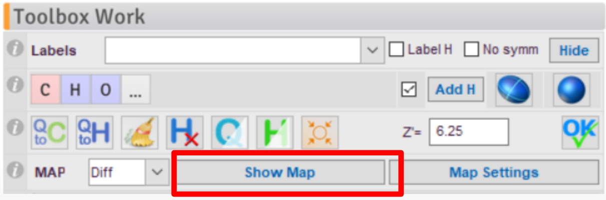

Refinement

In a nutshell: What are we actually seeing?

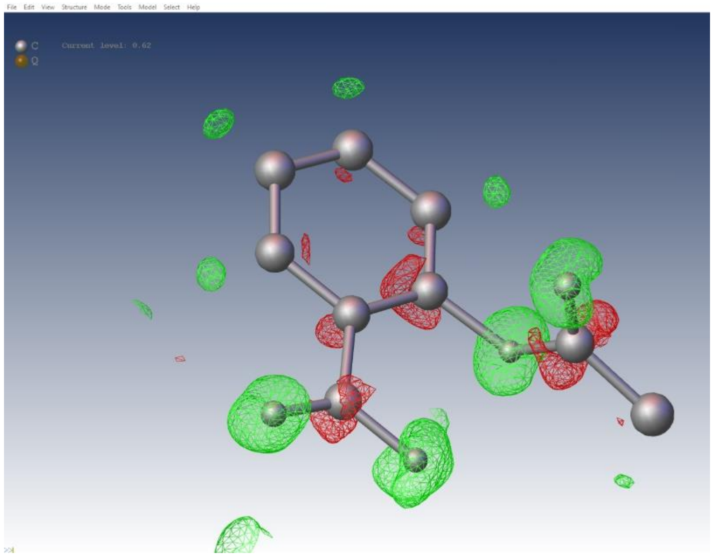

What do the grey spheres in our structural image actually represent?

At first glance, one might assume that they are atoms.

In fact, the centres of the spheres can be interpreted as atomic positions. However, the size of the spheres does not reflect the size or shape of the atoms, as in the calotte model below, but shows the so-called displacement parameters DP.

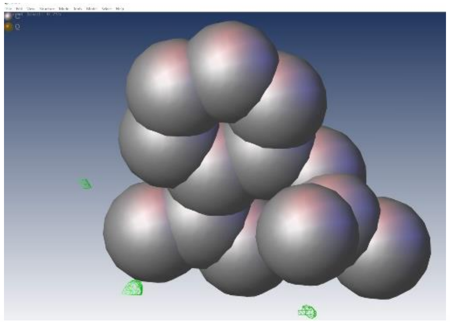

The example shows that the DPs of the four marked atoms are much too small, an indication that an atom type with too few electrons was assumed.

Refinement

Correction of the structural model and further refinement

We had seen that in our first structural proposal (only C atoms) too little electron density had been assumed at 4 non-ring atoms.

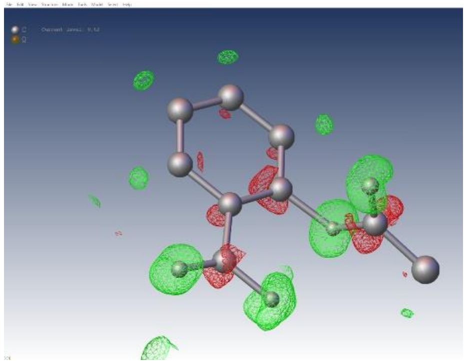

We therefore change our proposed structure by assigning these atoms as oxygen atoms: Select ‘O’ in the toolbox and then click on the corresponding atoms. To do this, the residual electron density map must be hidden (click on ‘Show Map’ again).

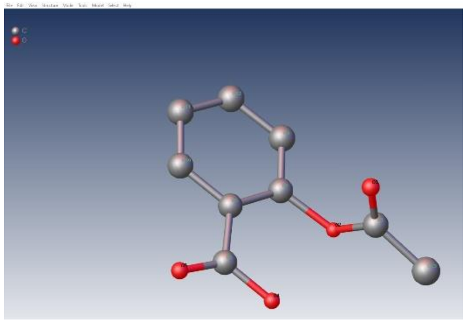

If you have accidentally assigned an atom type incorrectly, you can undo the action with ‘Ctrl’ and ‘z’.

By pressing the “Refine” button again, we can adjust this new structure proposal with our measurement data.

Refinement

Does our structural model match the data?

The R value is shown in the top right-hand window as a measure of how well our model matches the measurement data.

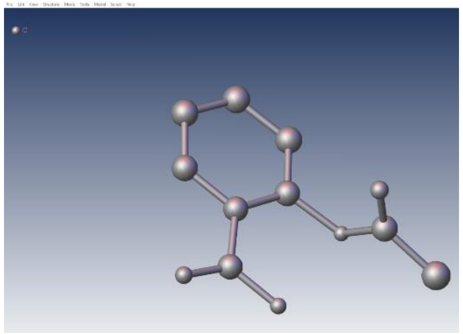

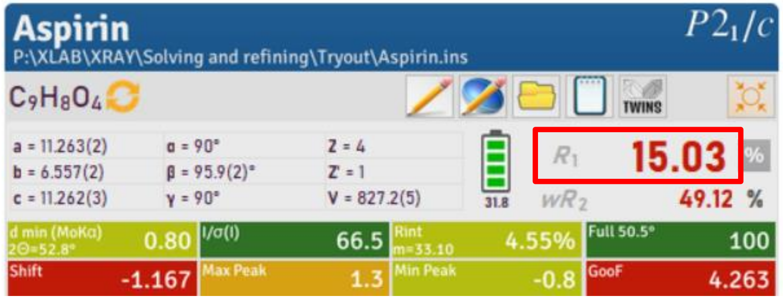

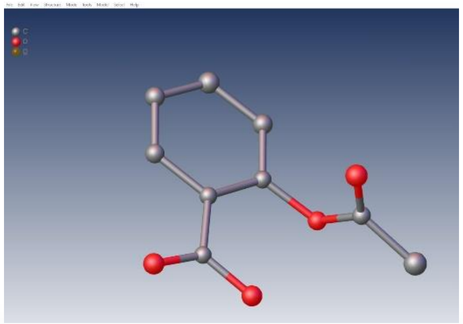

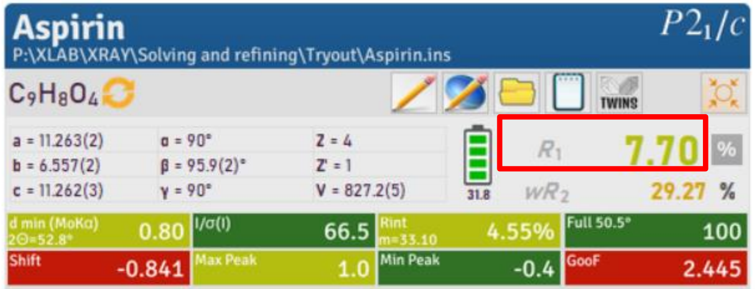

We can see that the R-value has fallen from 15% to around 8%.

Refinement

Anisotropic refinement:

So far, we have refined the vibrational behaviour to be spherical (isotropic). However, this vibrational behaviour is not realistic, especially for terminal atoms.

In the next stage of refinement, we assume that atoms move in an ellipsoidal shape by pressing the ‘Make Atoms Anisotropic’ button in the toolbox and thus refining anisotropically.

The R value drops again to 6.74 % and new Q peaks appear. We hide them again and display the residual electron density (‘Show Map’).

How can this ‘green’ residual electron density be interpreted?

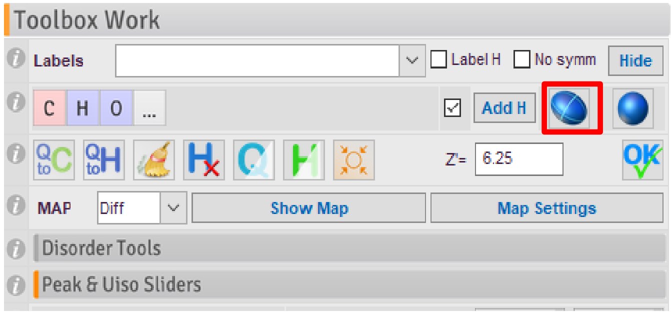

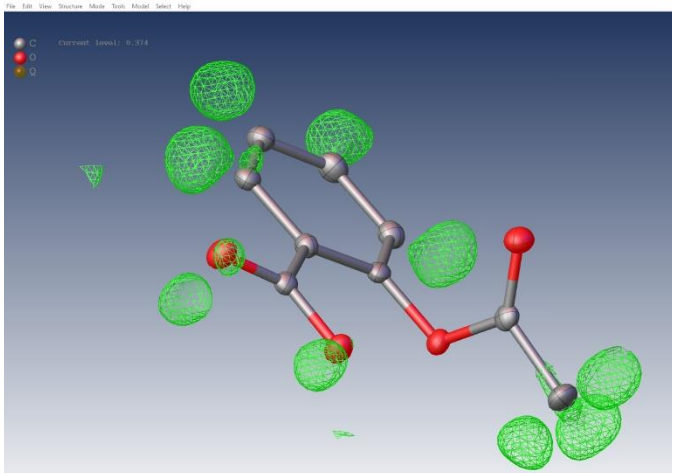

Labelling of the atoms

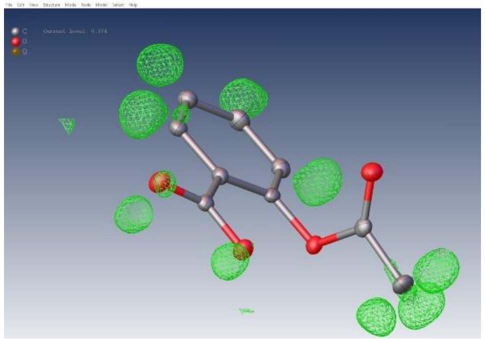

Of course you have guessed it: the green areas of additional residual electron density near atoms are the hydrogen atom positions.

However, before we assign H atom positions to these Q peaks, it is useful to name the existing atoms in a meaningful way. Hide the residual electron density map and left-click on the 'Naming' button in the 'Work' area. You may have to scroll down in the work window to do this.

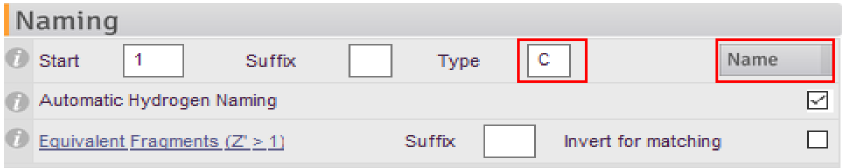

To label the C atoms, starting with C1, enter 'C' as the type and start the labelling by clicking on 'Name'.

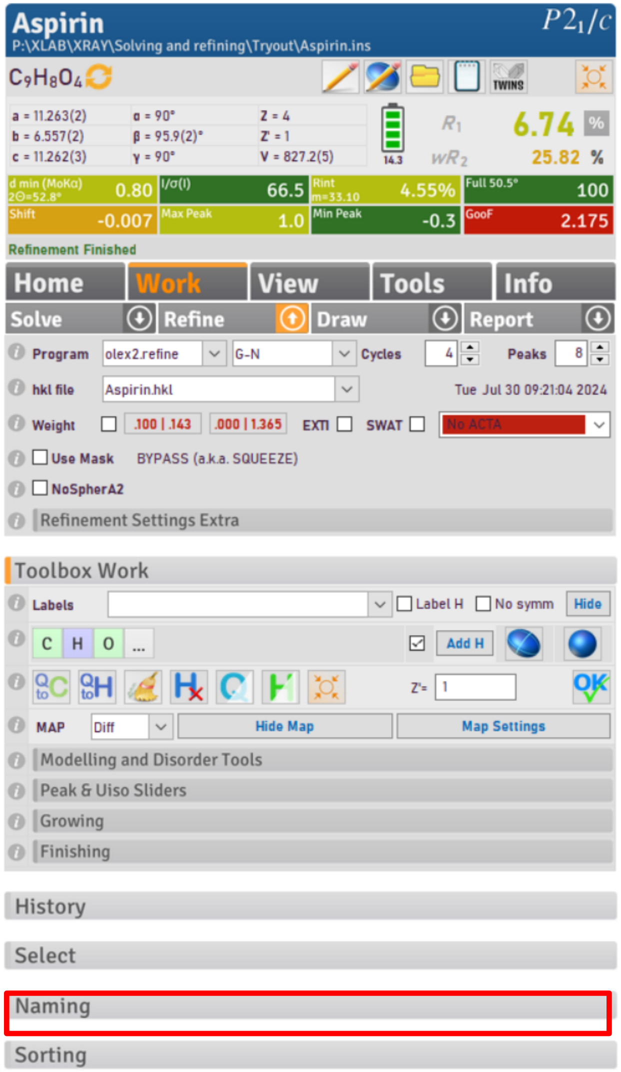

Labelling of the atoms

By clicking on the C atoms, we can number them consecutively. It makes sense to proceed systematically, e.g. clockwise.

We then change the atom type to “O” and confirm this input by pressing “Name”. Now we can number the oxygen atoms by clicking on them.

Finally, we exit ‘Renaming Mode’ by pressing the ‘Esc’ key.

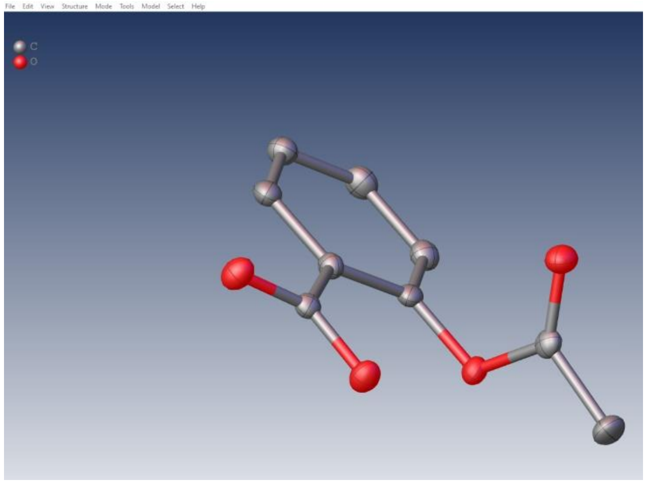

Caution: In ‘Renaming Mode’ it is possible to overwrite the atom types. If you have accidentally changed the atom type, you can undo the last step by pressing ‘Ctrl’ and ‘z’ at the same time.

Adding hydrogen atoms

H atoms on oxygen atoms:

A Q peak after anisotropic refinement is located near an O atom. We can assume that this is a hydroxyl hydrogen atom.

We assign the atom type ‘H atom’ to this Q peak in the ‘Toolbox’ and refine again.

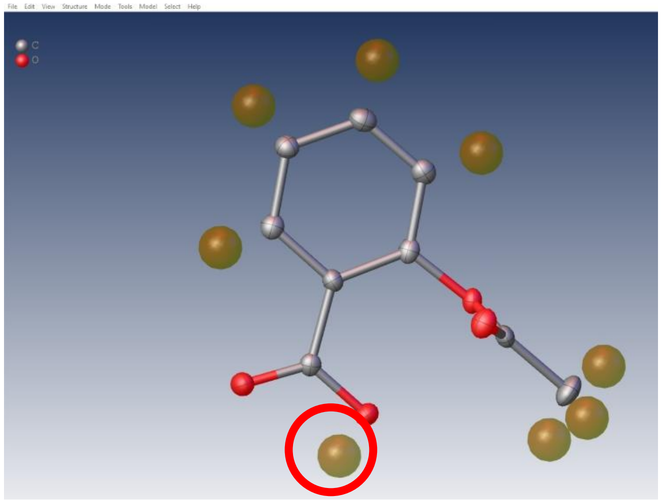

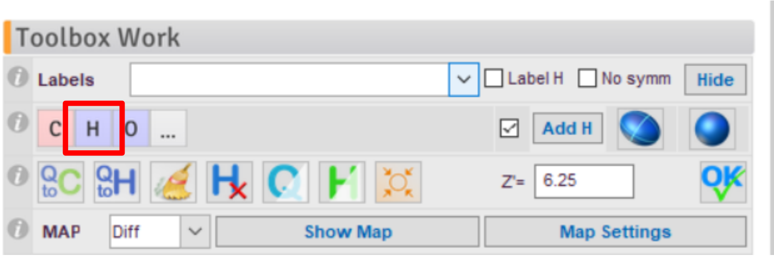

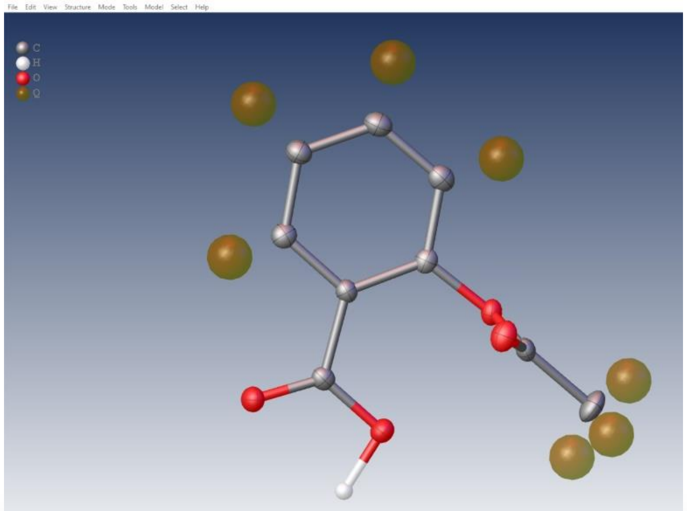

The refinement improves the R-value to 6.68 %.

Adding hydrogen atoms

H atoms on carbon atoms:

After refining the hydroxyl H atoms, we recognise that the remaining Q peaks are located in the neighbourhood of carbon atoms.

H atoms on carbon atoms are usually not refined ‘freely’, but are given a chemically meaningful geometry and then adapted to the measurement data.

The programme does this for us when we press ‘Add H’ in the toolbox. Please check that the correct number of H atoms have been added to the respective carbon atom type (sp^3, sp^2 or aromatic). As a rule, H atom positions are only ever refined isotropically.

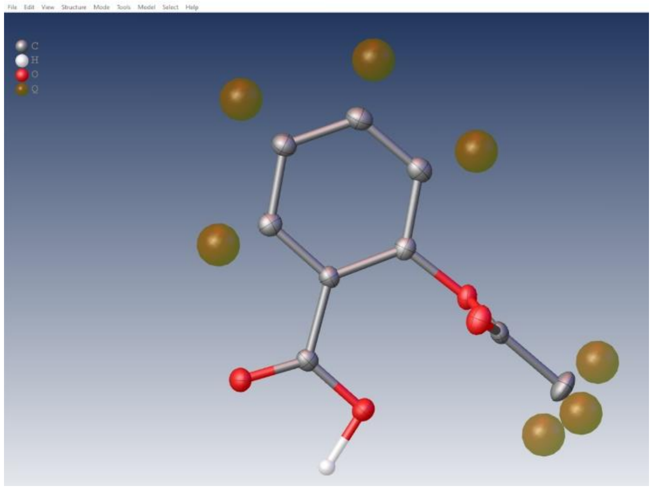

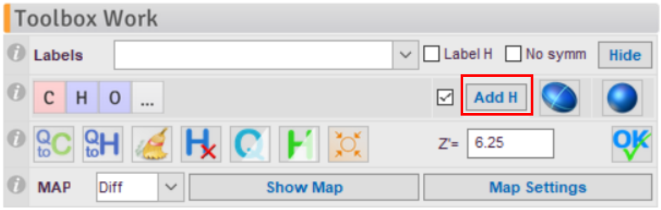

The finishing touch

Refining the weighting scheme:

At the very end, we refine the weighting scheme by ticking ‘Weight’ in the ‘Refine’ section and refining it again, now with 10 cycles. This takes into account that weaker diffraction maxima have a larger error than strong ones and ensures that the refinement is complete. If necessary, repeat the refinement until the values next to ‘weight’ are displayed in green. In this case you will only reach red and orange numbers.

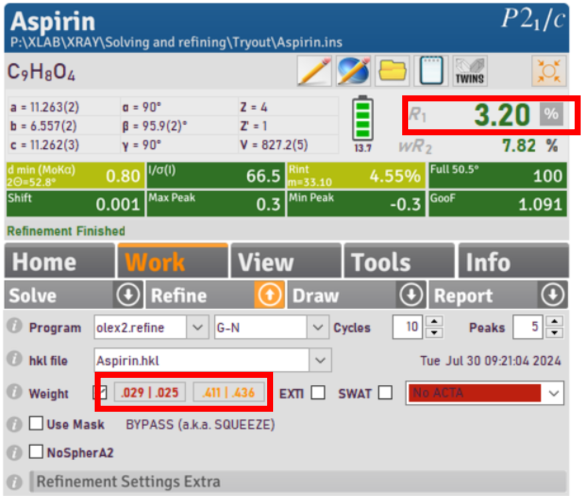

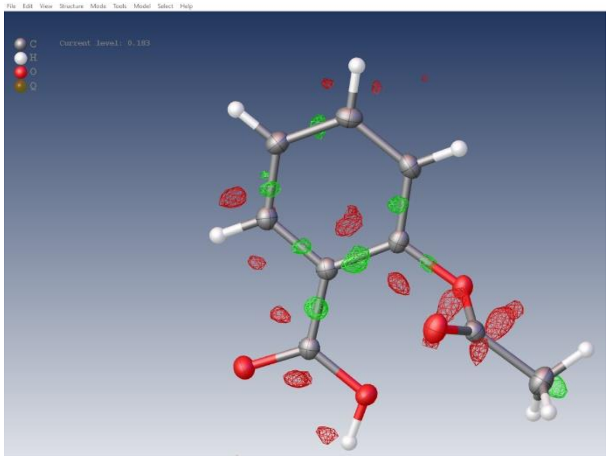

We note that the R-value has dropped to 3.20 %. This low value shows us that the structural model is in good agreement with the diffraction data.

Analysis of the structure

Analysing bond lengths and angles:

Our structure is now completely refined and thus provides much more information than just the connectivity of the atoms.

By hovering the mouse pointer over a bond, the bond distance is displayed in Å (1 Å = 100 pm). Alternatively, you can also right-click on the bond.

Analyse the bonds in the molecule and assign the bond type using the standard bond lengths and draw a structural formula.

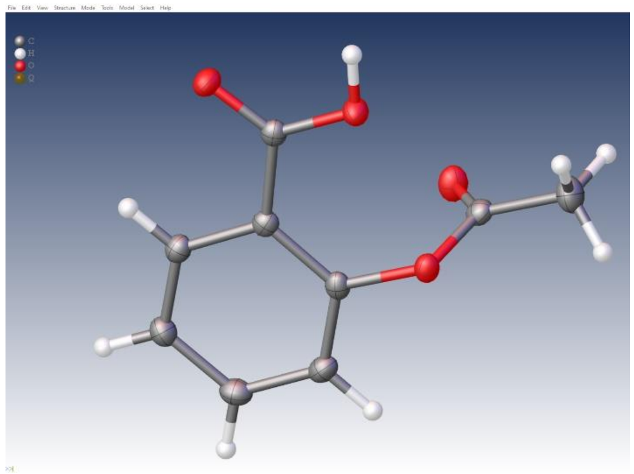

Bindungstyp	Typische Bindungslänge in Å
C(sp³)-C (sp³)	1.54
C(sp²)-C (sp²)	1.47
C(sp³)-C(sp²)	1.50
C=C	1.34
C-C (aromatisch)	1.40
C-O	1.43
C=O	1.20

Analyse der Struktur

Analysing bond lengths and angles:

More information on the structure can be obtained by clicking on the arrow next to the ‘Report’ button and then selecting ‘HTML Report’. A browser window opens with a lot more information, such as a complete list of bond lengths and angles. With the help of torsion angles (dihedral angles) between 4 atoms, you can make statements about the planarity of these 4 atoms.

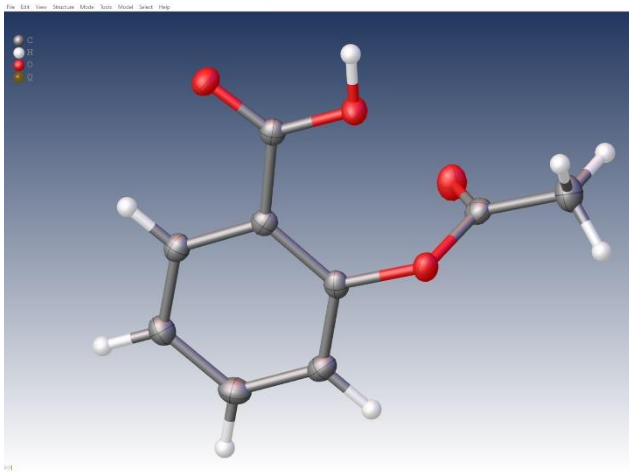

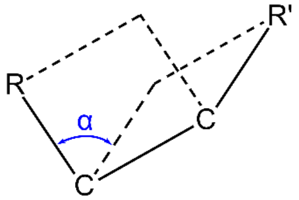

Torsion angle α
between R-C-C-R'

Did you find that out too?

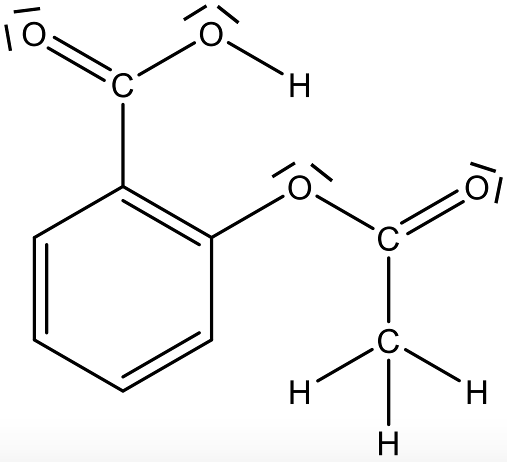

Creating images

Photo shooting!

A nice photo or a moving image series of ‘our’ molecule in a meaningful orientation can help us to share the results of the structure determination with others.

Under the tab ‘Tools’ and ‘Images’ you will find a lot of options for creating nice molecule images.

Just give it a try!

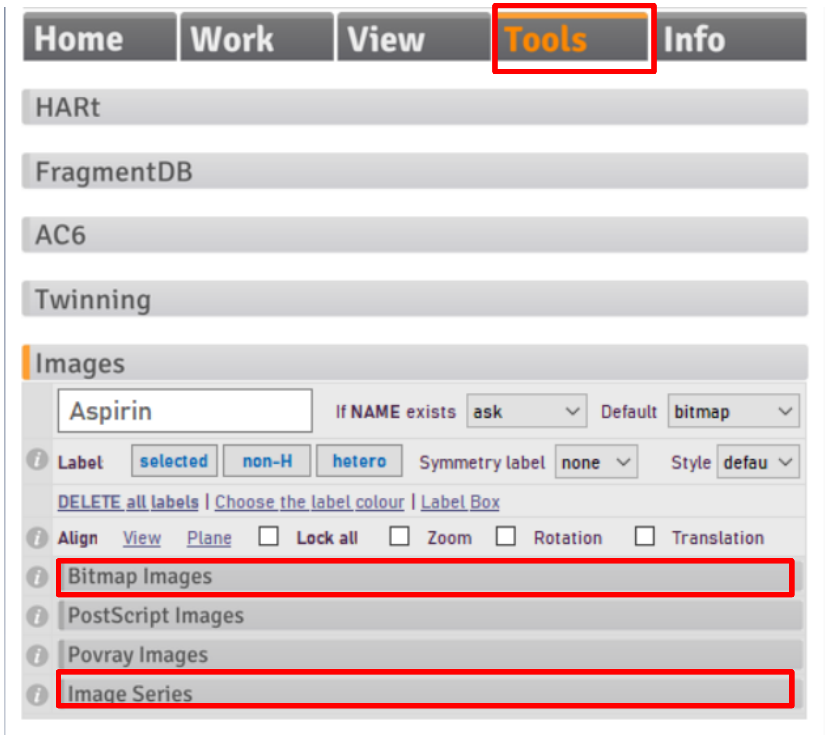

Practise…

Now that you have solved and refined your first structure yourself under guidance, you can work independently on the other test structures.

You are sure to encounter a few difficulties along the way. Always remember: chemical expertise often helps here, for example about the bonding states of certain types of atoms.

To check your own refinement results, you can compare them with the 'sample solutions' stored in the 'final' folder. Open the relevant res file in Olex2, refine it again and compare the result with your last refinement.

Have fun!

Dolomanov, O.V.; Bourhis, L.J.; Gildea, R.J.; Howard, J.A.K.; Puschmann, H., OLEX2: A complete structure solution, refinement and analysis program (2009). J. Appl. Cryst., 42, 339-341.
