# Supplementary material for: Crystallography in school
Source: J Appl Crystallogr. 2025 Sep 12;58(Pt 5):1802–9. doi: 10.1107/S1600576725007459 (PMC12502877; doi:10.1107/S1600576725007459)
Supplement: Supplementary file 7 [file j-58-01802-sup7.pdf]

GEORG-AUGUST-UNIVERSITÄT GÖTTINGEN
IN PUBLICA COMMODA SEIT 1737

Step-by-step guide to structure solution and refinement with ShelXle

Aspirin

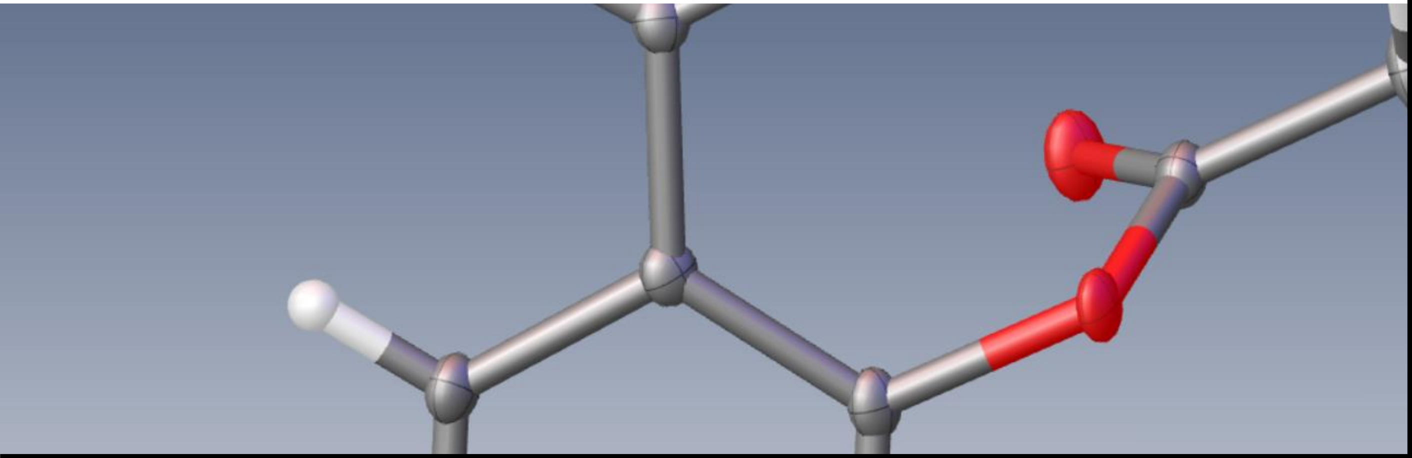

Erhard Irmer

XLAB Göttinger Experimentallabor für junge Leute

Overview

- Introduction 4
- Structural solution 5
- Control system 7
- Naming the atoms 9
- Refinement 10
- Add hydrogen atoms 18
- The final touches 20
- Analysing the structure 22
- Creating images 28

Start of the programme

Double-click to open the "ShelXle XLAB Edition" programme on the computer desktop.

The programme interface opens.To load the aspirin structure, click on the ".res" button on the right-hand side of the screen and then navigate to the "aspirin.ins" file, which is located in the "Aspirin - Tutorial" subfolder on the computer's desktop.

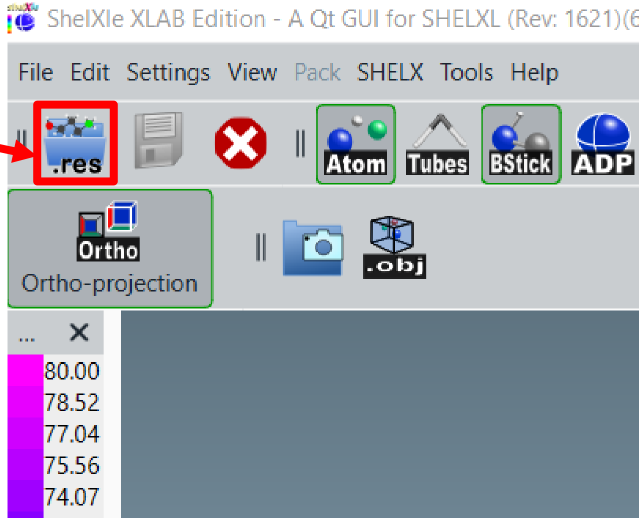

Introduction

Structure:

ShelXle essentially consists of four areas. A window in which the structural model is displayed, the command menu, an info and results window and the instruction statement.

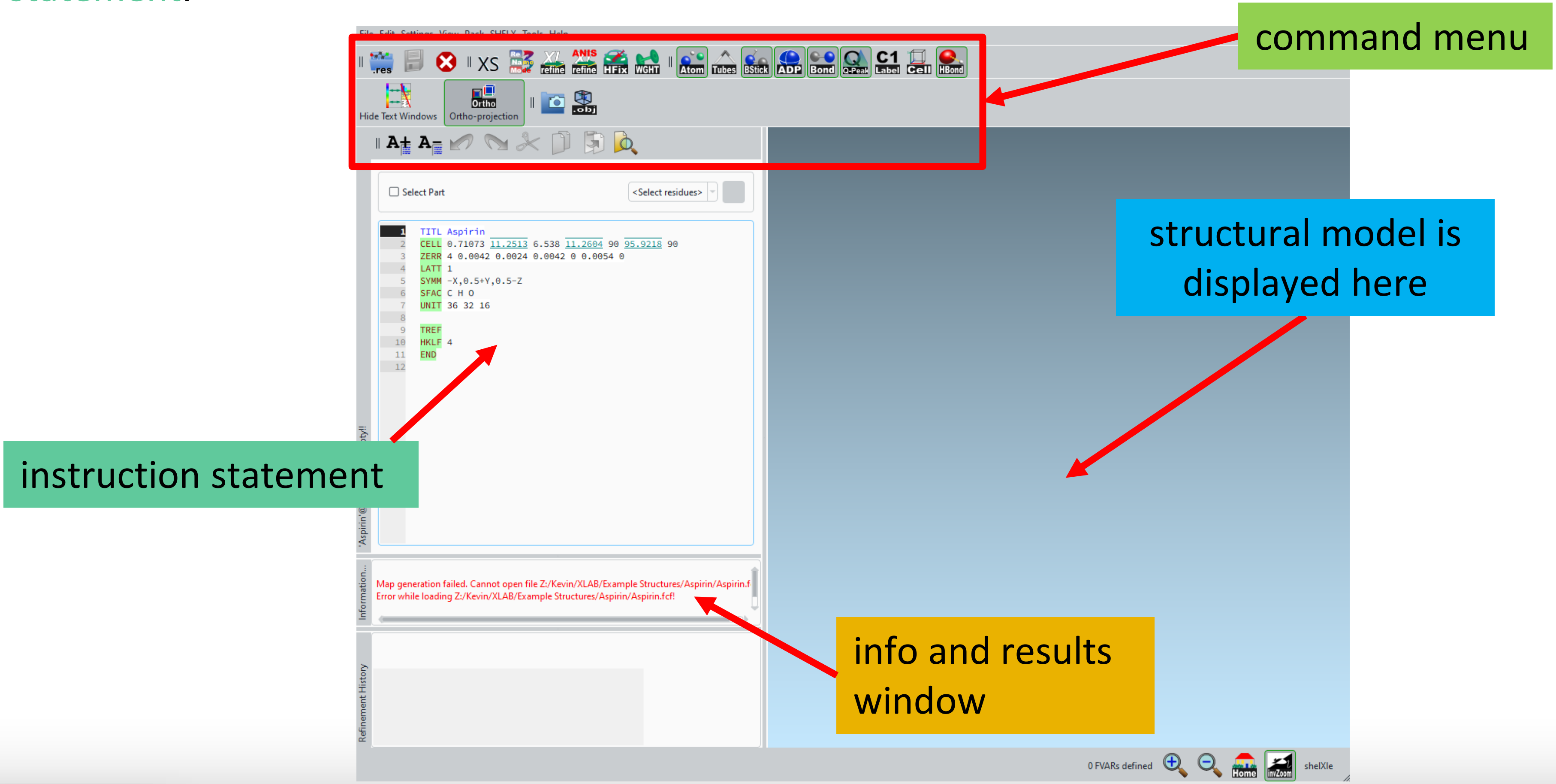

Structural solution

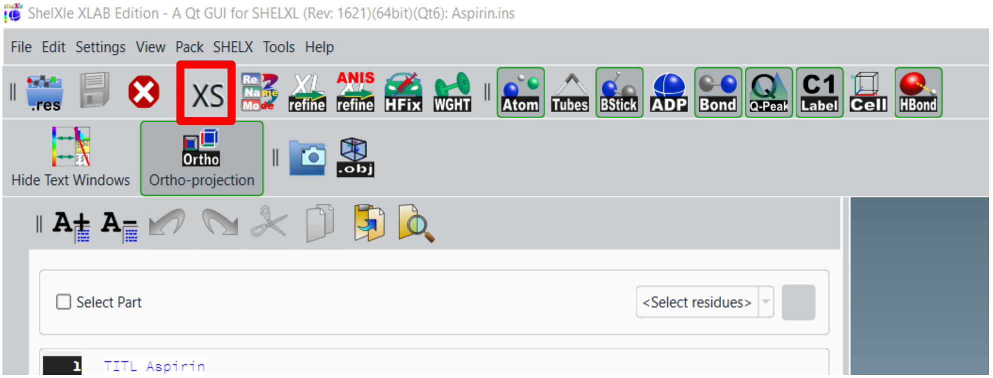

The first step is to carry out the structure solution. Statistical methods are used to assign phases to each reflex and the chemically most sensible solution is output according to various criteria. ShelXle uses the "ShelXS" programme for this purpose, which is executed by clicking on the "XS" symbol.

The structural model is loaded by clicking on "Load Aspirin.res" in the window that now appears.

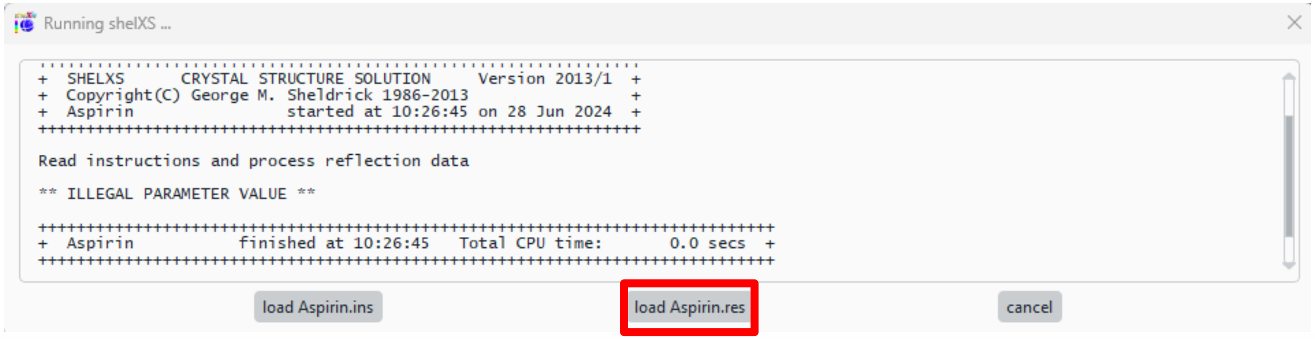

XLAB Göttinger Experimentallabor für junge Leute

GEORG-AUGUST-UNIVERSITÄT GÖTTINGEN

Structural solution

Structural solution:
A series of coloured polyhedra now appear in the right-hand window. These are so-called Q peaks, i.e. places where a high electron density was measured. They may therefore be potential atomic positions.

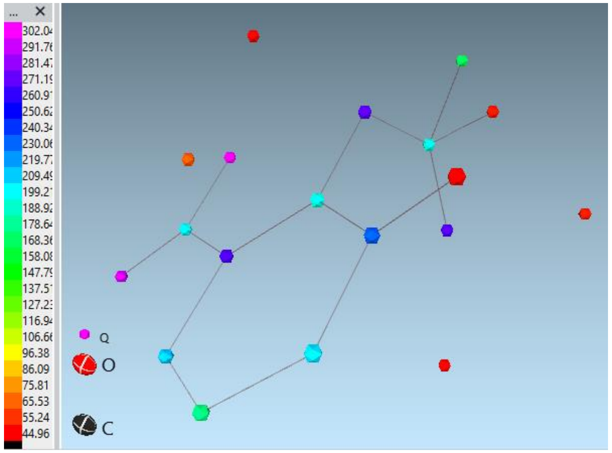

The colour scale shows the electron density from red (low electron density) to violet (high electron density).By clicking on the yellow-orange area of the colour scale, the Q peaks with low electron density can be hidden.

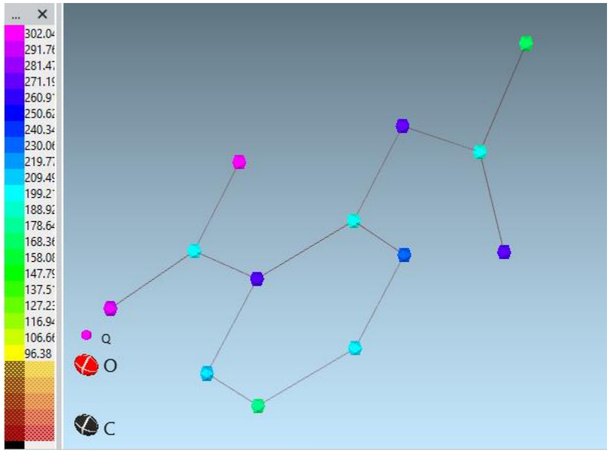

Structures, such as six rings, are now often already recognisable.

Control system

The size of the labelling of the Q peaks (label) is controlled by turning the mouse wheel.The molecule can be moved by holding down the mouse wheel.

The molecule is rotated by moving the mouse while holding down the left mouse button.

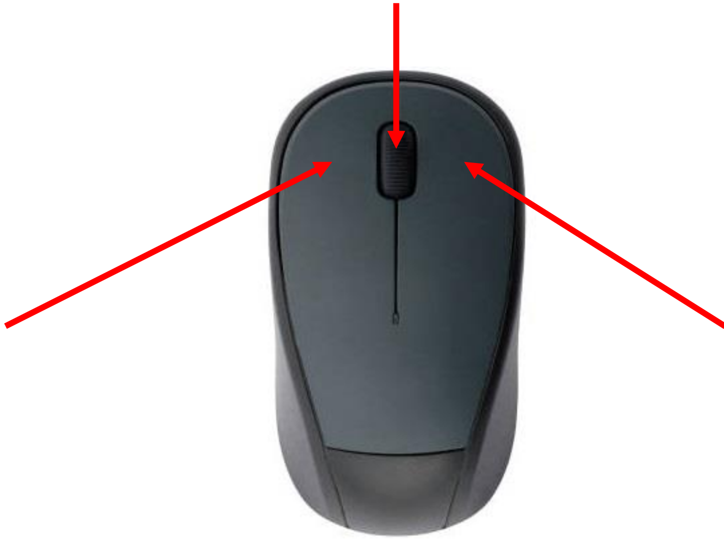

The display can be zoomed by moving the mouse while holding down the right mouse button.

Try this out until you have developed a feel for it.

Structural solution

Assignment of Q peaks to atoms:

The displayed Q peaks can be assigned to atom types.

However, it is often not known what the exact composition of the compound is. In this case, all Q peaks of an organic compound could first be labelled as C atoms. Let's proceed in this way.

The next slide shows how atoms can be assigned to the Q peaks.

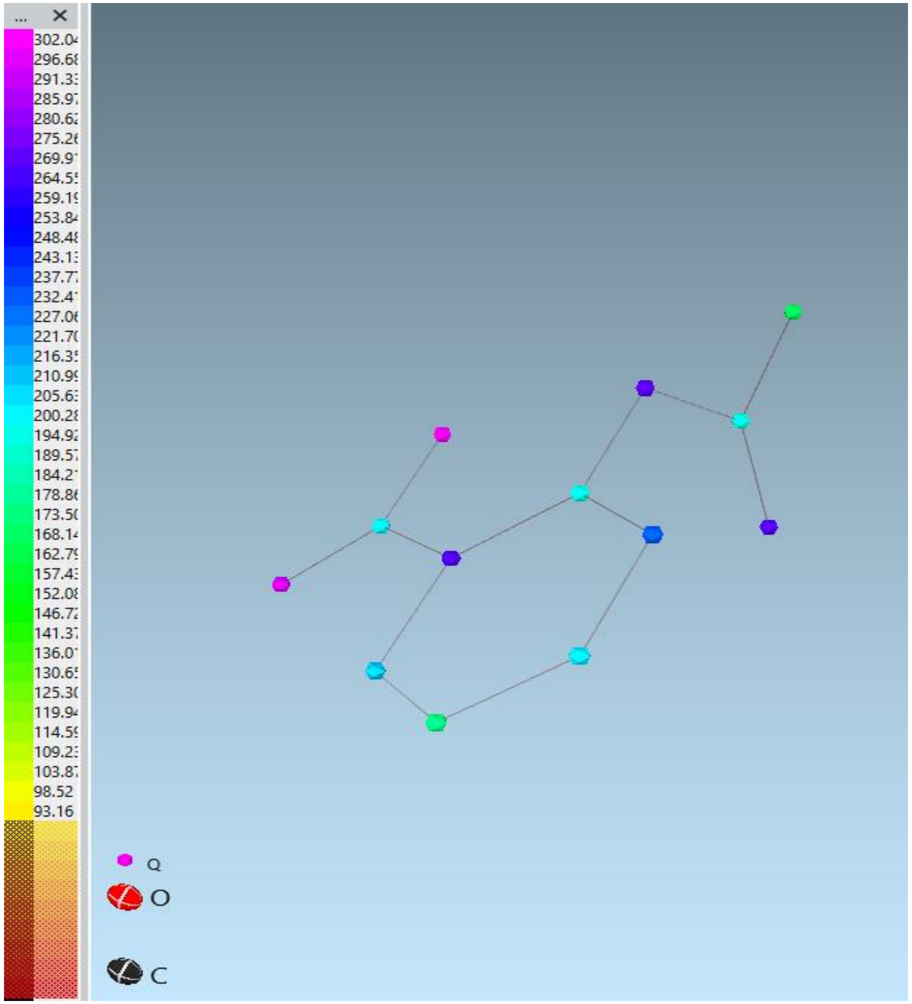

Presumed gross formula of the compound : $C_9H_8O_4$

Structural solution

Assignment of the Q peaks to atoms: To do this, switch to the "Rename mode".

A window will open on the left-hand side in which you can select the desired atom type (in our case, C atoms for now).

Then click on the Q peaks in the structure window that you want to convert into C atoms.

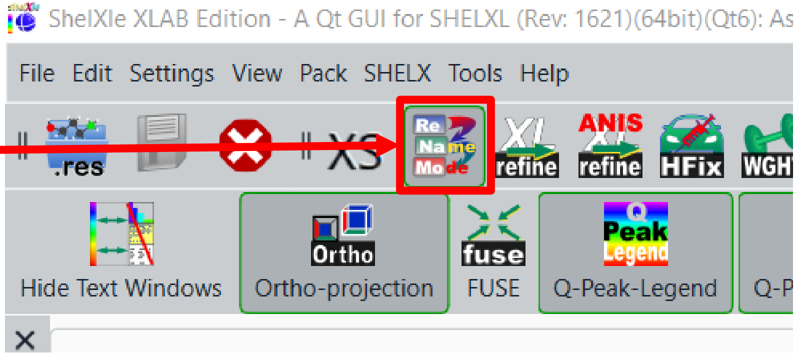

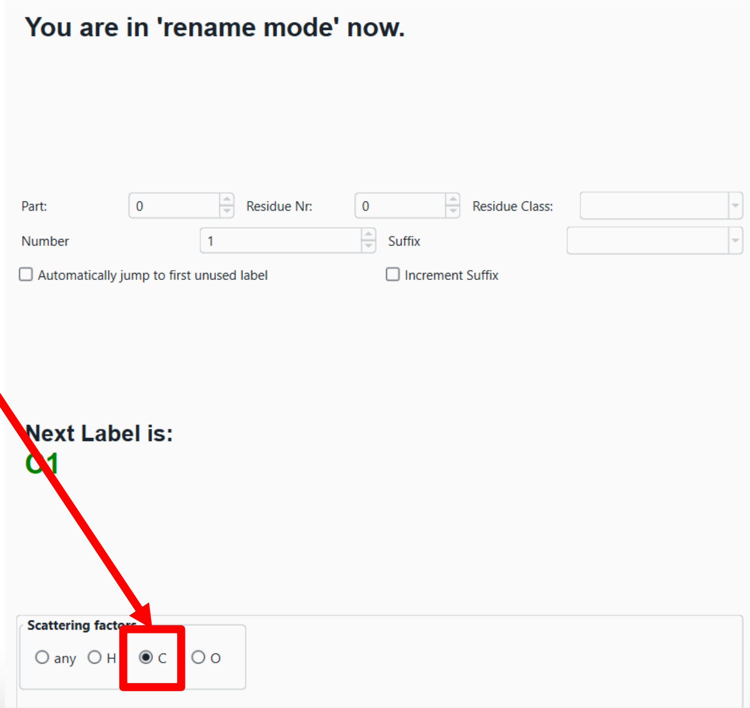

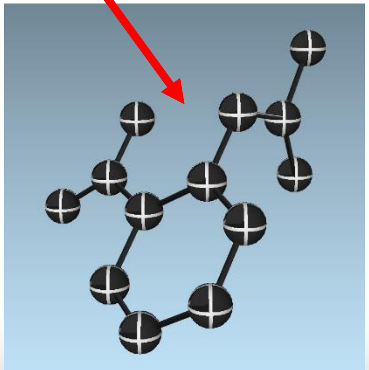

Refinement

First refinement:
We start the refinement by clicking on the "XL refine" button. Now we check whether the specified structure model (in our case all C atoms) matches the measured intensity data from the diffraction measurement.

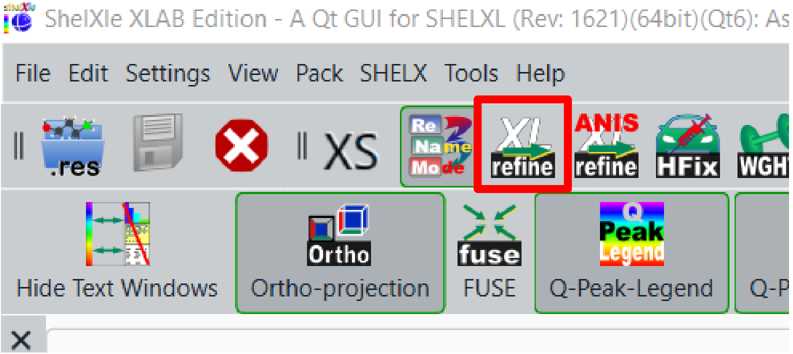

A refinement protocol is displayed in the results window on the left. We must call up the result of the refinement by clicking on the "Load refinement results" button.

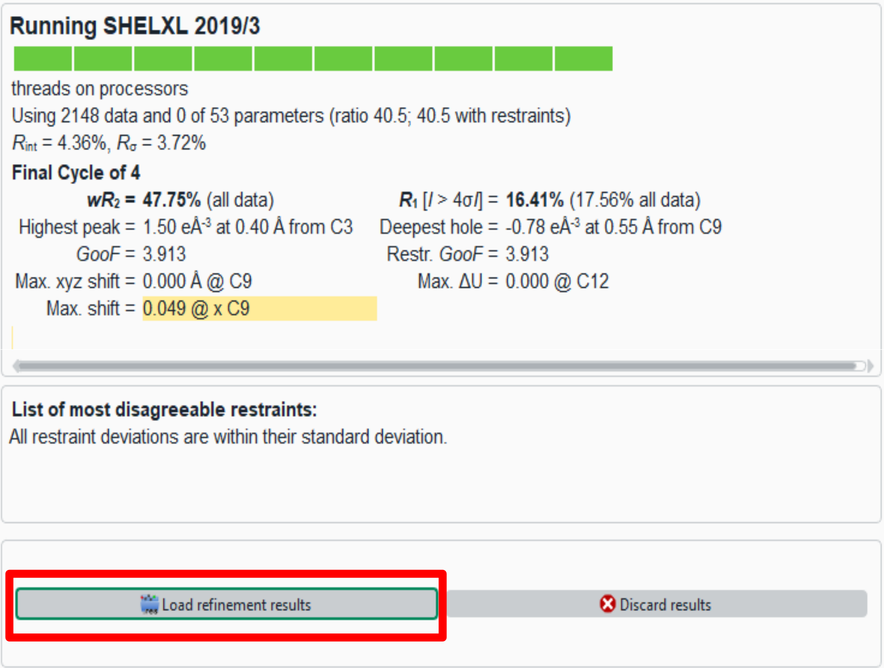

The refinement is only complete once the results have been loaded.

Refinement

First refinement: The result

After refinement, new Q peaks have appeared and some atoms have a green "net" around them. This indicates that too little electron density was assumed at these points in our structural model in comparison with the measurement data.

This is therefore probably an atom with more electrons.

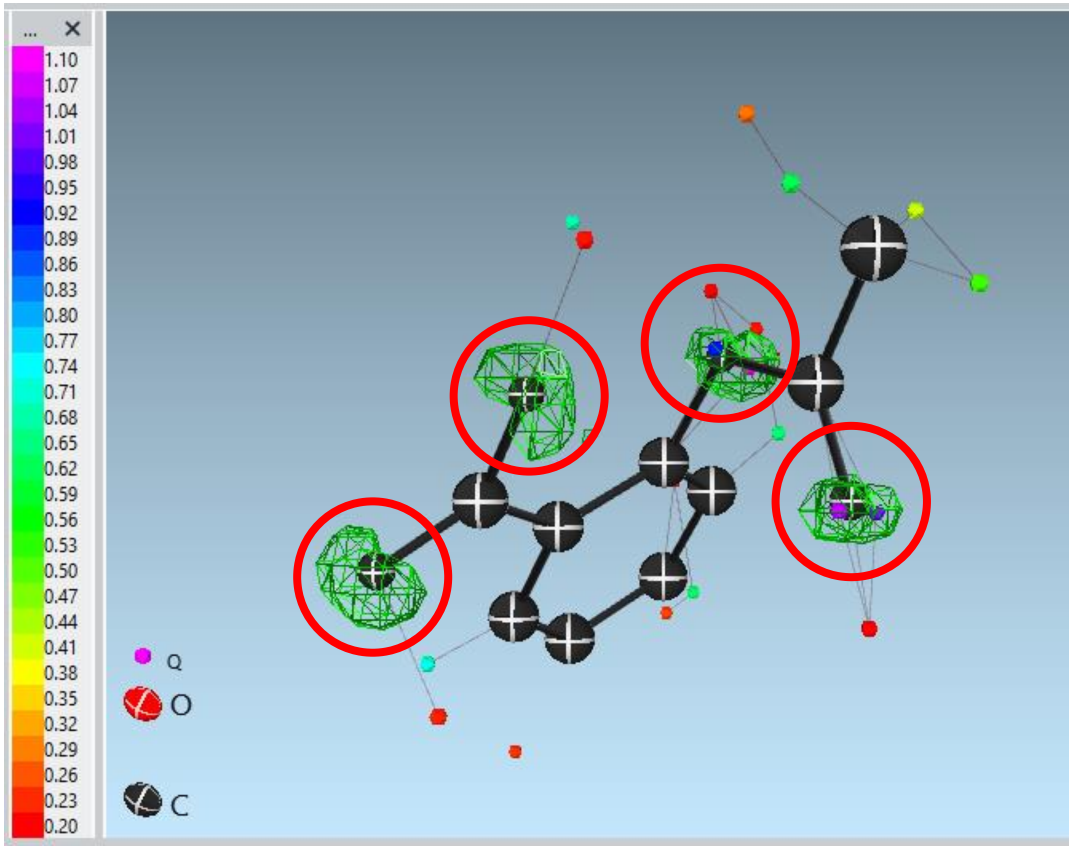

Refinement

What are we actually seeing?

What do the black spheres in our structural image actually represent?

At first glance, you might assume that they are atoms. In fact, the centres of the spheres can be interpreted as atomic positions. However, the size of the spheres does not reflect the size or shape of the atoms, as in the dome model below, but shows the so-called displacement parameters DP.

In the example, we can see that the DPs of the four marked atoms are smaller than the others, which is another indication that an atom type with too few electrons was assumed.

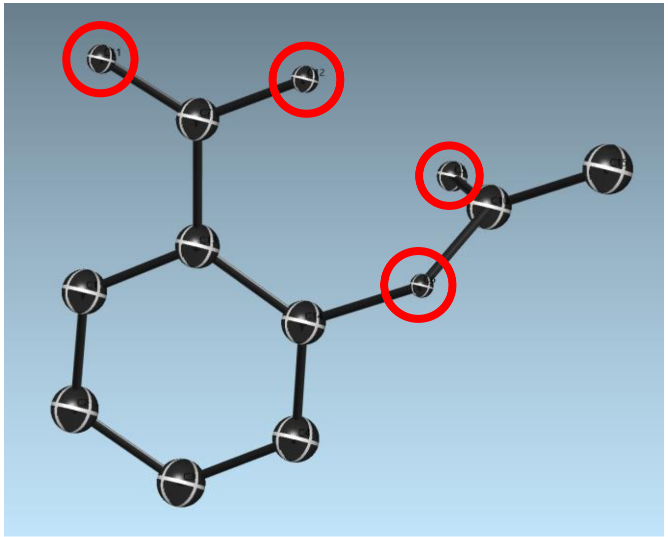

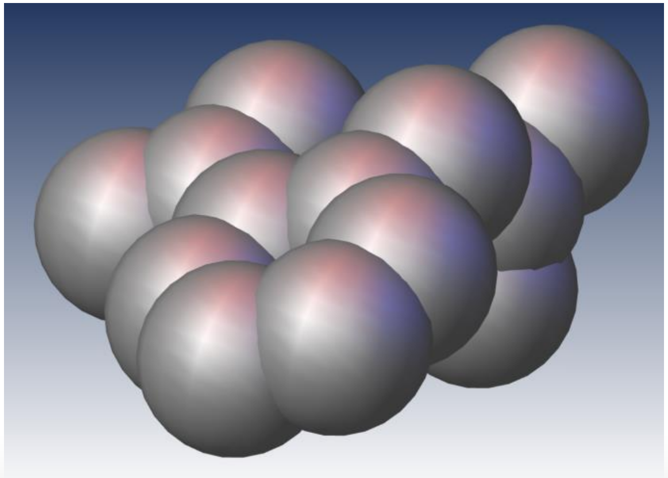

Refinement

Correction of the structural model and further refinement

We had realised that our first structural proposal (only C atoms) had assumed too little electron density at 4 C atoms.

We therefore change our structure proposal by assigning these atoms as oxygen atoms.

To do this, we switch back to "Rename mode" as on slide 9, select the "O" atoms option and click on the corresponding four atoms.

We then refine again and load the result, which appears in the structure window.

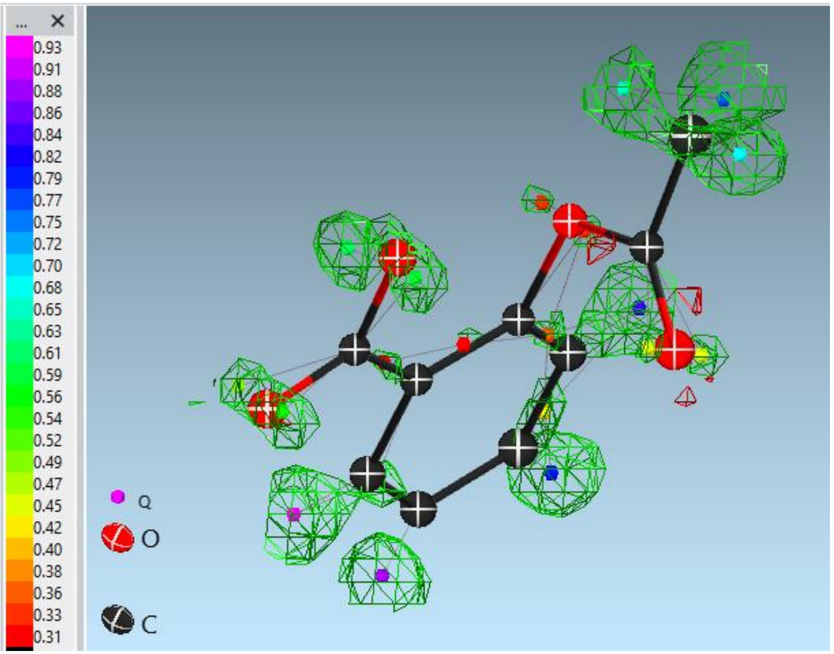

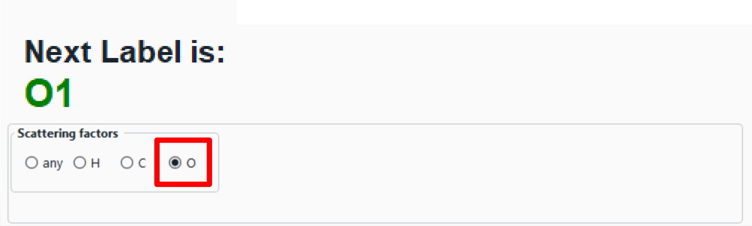

Refinement

Does our structural model match the data?

As a measure of how well our model agrees with the measured data, the development of the R values is shown graphically at the bottom left. A large R-value means that our model deviates significantly from the measured data.

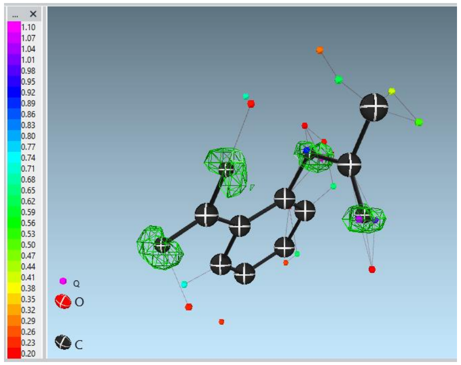

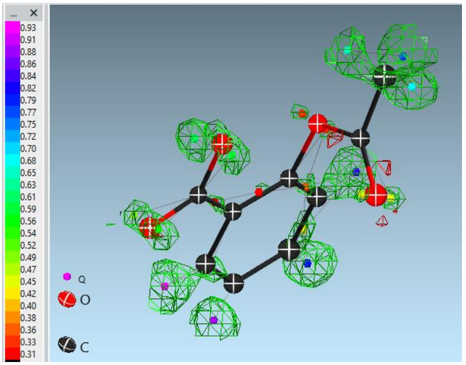

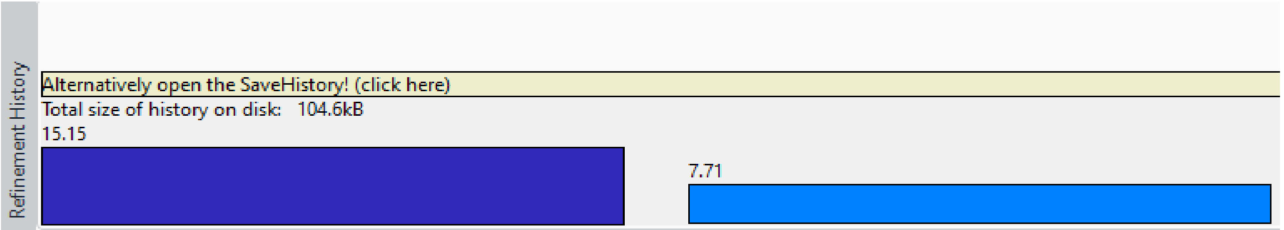

We can see that the R-value has fallen from over 15% to around 7%.

Refinement

Anisotropic refinement:

So far, we have refined the vibrational behaviour to be spherical (isotropic). However, this vibrational behaviour is not realistic, especially for terminal atoms.

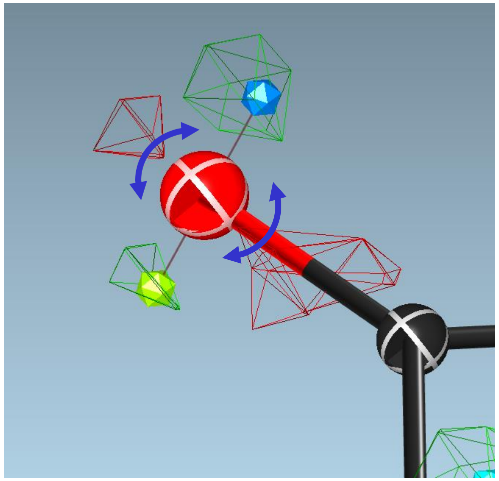

Instead, the following slide shows how the vibrational behaviour can be considered anisotropic.

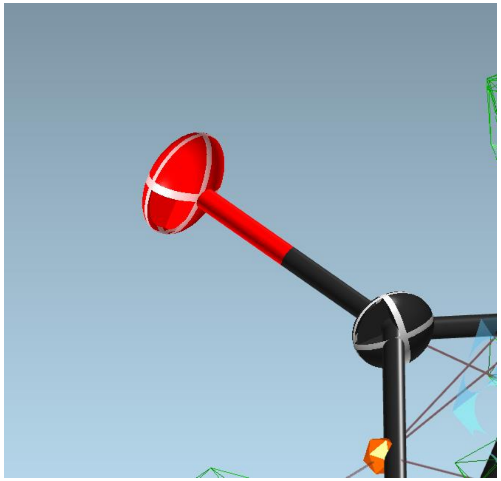

Refinement

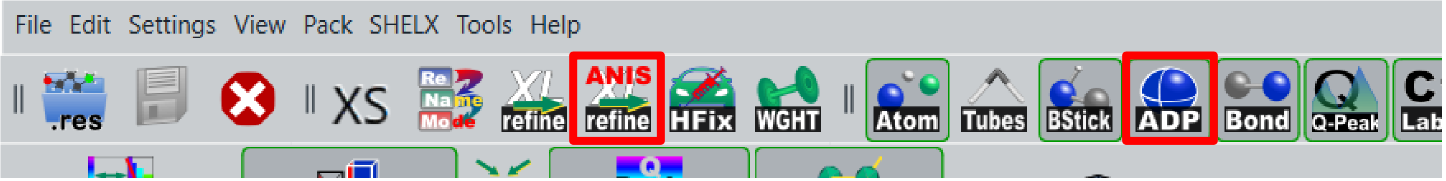

Anisotropic refinement:

In the next round of refinement, we assume that atoms move in an ellipsoidal shape by pressing the "ANIS refine" button in the command menu and thus refining anisotropically. Click on the "ADP" button to ensure that the anisotropic displacement parameters are displayed.

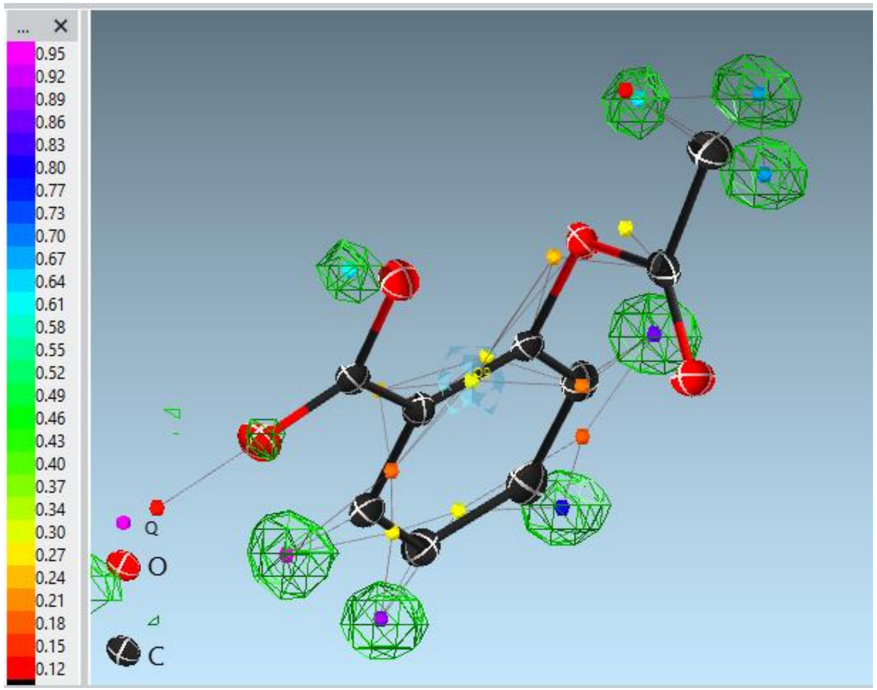

The R-value drops again to 6.73 % and new Q-peaks appear.

Naming the atoms

Of course you have guessed it: the green areas of additional residual electron density near atoms are the hydrogen atom positions.

However, before we assign H atoms to these Q peaks, it makes sense to name the existing atoms.
To do this, simply go into rename mode and name the C and O atoms according to a meaningful scheme.

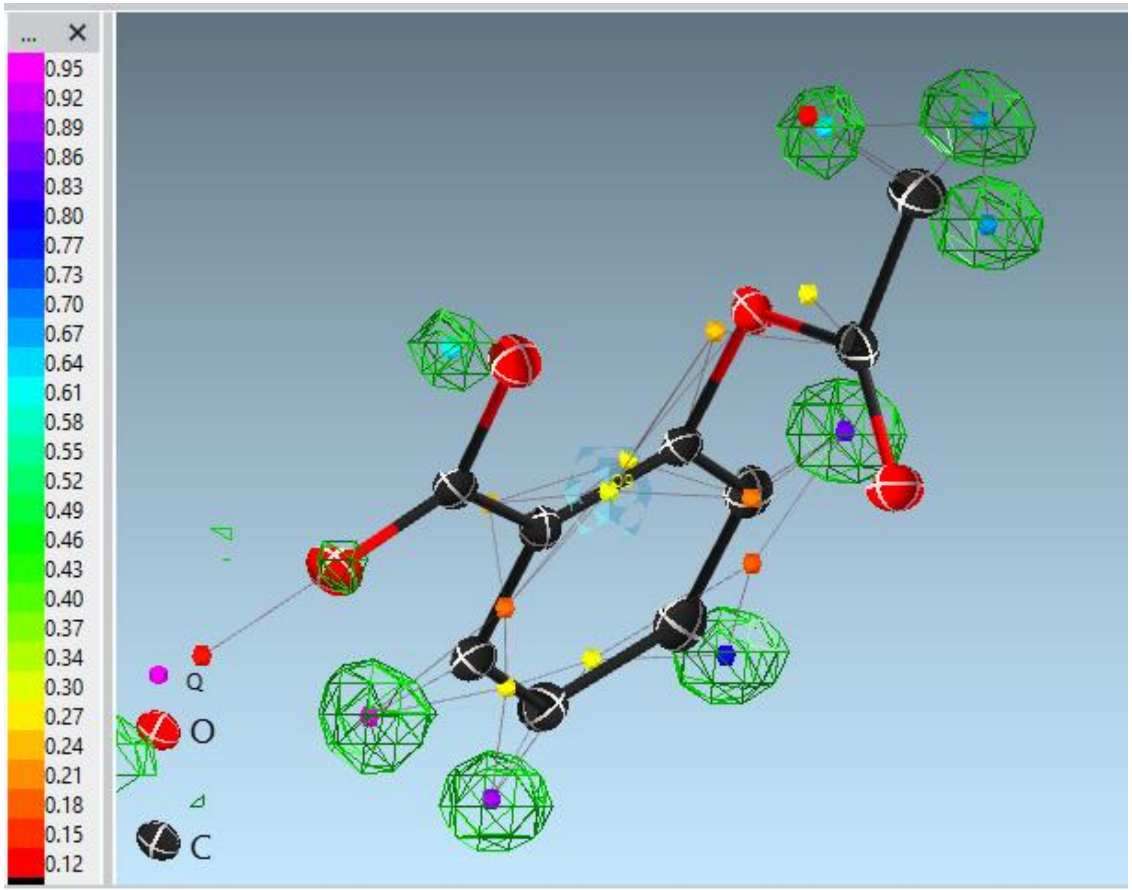

Add hydrogen atoms

H atoms on oxygen atoms:

A more intense Q peak after the anisotropic refinement is located near an O atom. We can assume that this is a hydroxyl hydrogen atom.

We assign the atom type "H atom" to this Q peak in "Rename" mode (slide 9) and refine again using the "XL refine" button.

In this step, only the H atom located near the oxygen atom should assigned.

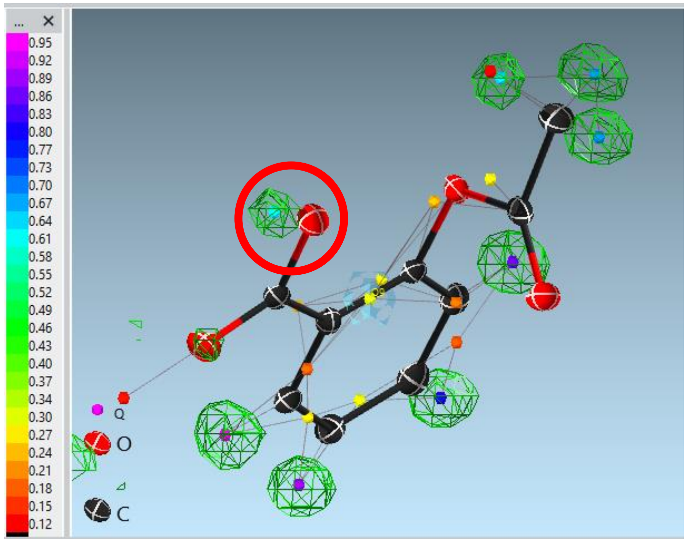

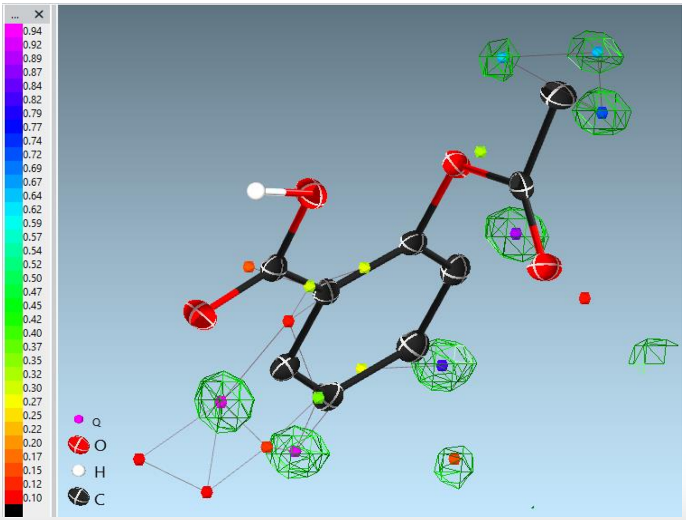

Add hydrogen atoms

H atoms on carbon atoms:

After refining the hydroxyl H atom, we recognise that the remaining more intense Q peaks are located in the vicinity of carbon atoms.

H atoms on carbon atoms are usually not refined "freely", but are given a chemically meaningful geometry and then adapted to the measurement data.

The programme does this for us when we press "HFix" in the toolbox. Please check that the correct number of H atoms has been set for the respective carbon atom type. Then refine again using "XL refine". As a rule, H atom positions are only ever refined isotropically.

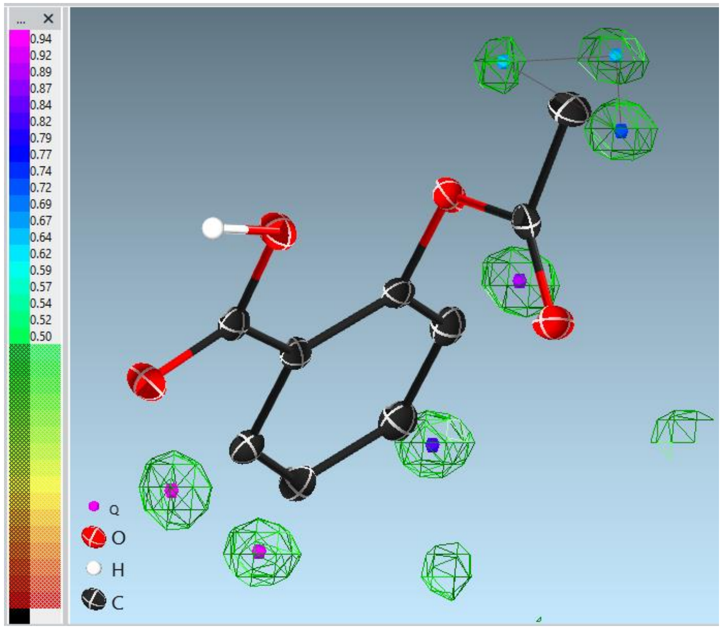

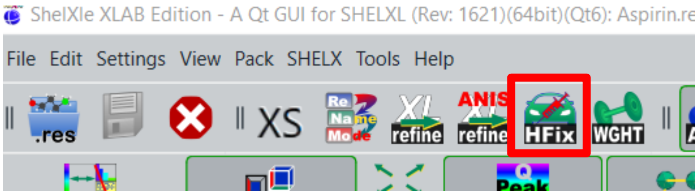

The refinement improves the R value to 3.41 %.

The finishing touches

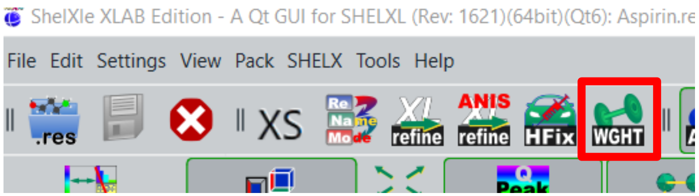

Refinement of the weighting scheme:

At the very end, we refine the weighting scheme by pressing the "WGHT" button. This takes into account the fact that weaker reflexes have a greater error than strong ones and ensures that all atoms are found.

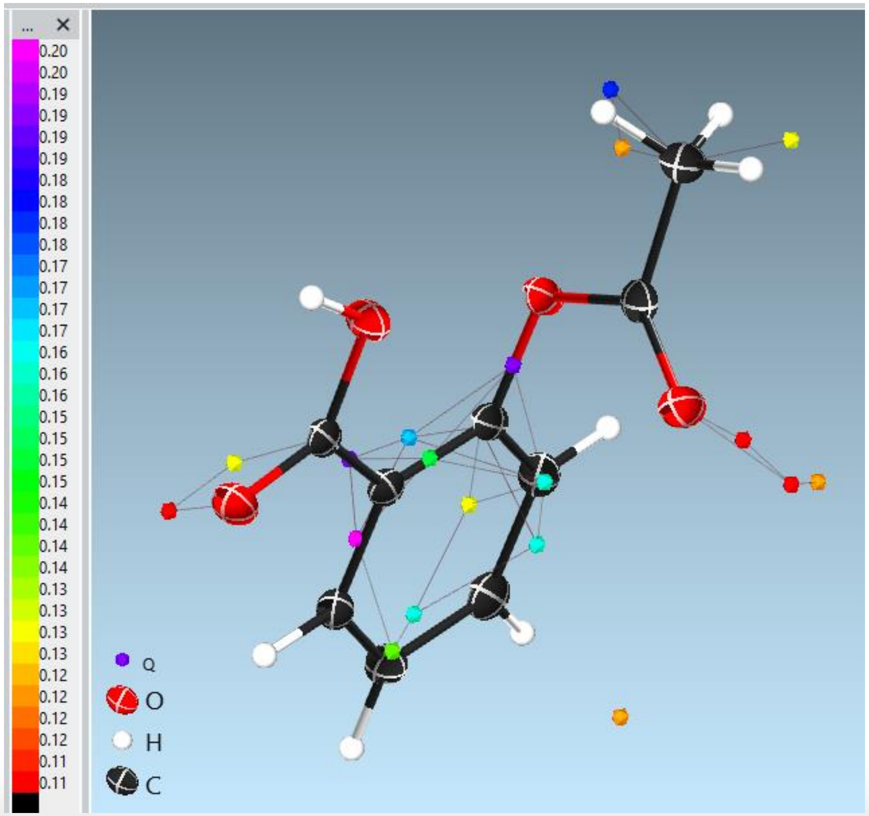

We note that the R-value has dropped to 3.20 %. This low value shows us that our structural model is in good agreement with the diffraction data.

Instruction manual

- All instructions and commands that were made in the previous refinement steps are saved here.

- After " CELL " in line 4 you can see the wavelength of the X-rays, the edge lengths and angles of the unit cell.
- With " SFAC " in line 8 information about the atom types used and with " UNIT " in line 9 about the number in the unit cell.
- The rest are some special refinement commands.
- From line 19 onwards, information about the individual atoms is given, with the coordinates and vibrations next to the atom name.

```
 1  TITL Aspirin
 2      Aspirin.res
 3      created by SHELXL-2019/3 at 15:07:15 on 27-Jun-2024
 4  CELL 0.71073 11.2513 6.538 11.2604 90 95.9218 90
 5  ZERR 4 0.0042 0.0024 0.0042 0 0.0054 0
 6  LATT 1
 7  SYMM -X,0.5+Y,0.5-Z
 8  SFAC C H O
 9  UNIT 36 32 16
10  LIST 6 ! automatically inserted. Change 6 to 4 for CHECKCIF!!
11  L.S. 4
12  BOND
13  FMAP 2
14  PLAN 20
15  WGHT    0.053100    1.164500
16  FVAR       0.84060
17  MOLE   1
18  MOLE   2
19  O1    3    0.490185    0.186461    0.404494    11.00000    0.02242    0.01849 =
20          0.01191    0.00268    0.00707    0.00471
21  C1    1    0.346485    0.440892    0.432949    11.00000    0.01471    0.01503 =
22          0.00755   -0.00125   -0.00031   -0.00095
23  O2    3    0.376047    0.142450    0.553685    11.00000    0.01968    0.01837 =
24          0.01334    0.00510    0.00664    0.00368
25  AFIX 148
26  H2A   2    0.420558    0.030586    0.565616    11.00000   -1.50000
27  AFIX   0
28  C2    1    0.384486    0.555639    0.339112    11.00000    0.01553    0.01778 =
29          0.00910   -0.00141    0.00249    0.00034
30  AFIX  43
31  H2    2    0.446763    0.506776    0.298784    11.00000   -1.20000
32  AFIX   0
33  O3    3    0.211052    0.419132    0.589752    11.00000    0.01797    0.01771 =
34          0.00532   -0.00066    0.00372   -0.00214
35  C3    1    0.331460    0.740230    0.304978    11.00000    0.01865    0.01807 =
```

'Aspirin'@ Aspirin.res | LIST FILE: Aspirin.lst | Visual Refinement output

Analysing the structure

Analysing bond lengths and angles:

Our structure is now fully refined and thus provides much more information than just the connectivity of the atoms.By clicking on neighbouring atoms, the bond distances are displayed in the info window on the left.

Examine the bonds in the molecule and assign the bond type using the standard bond lengths and draw a structural formula.

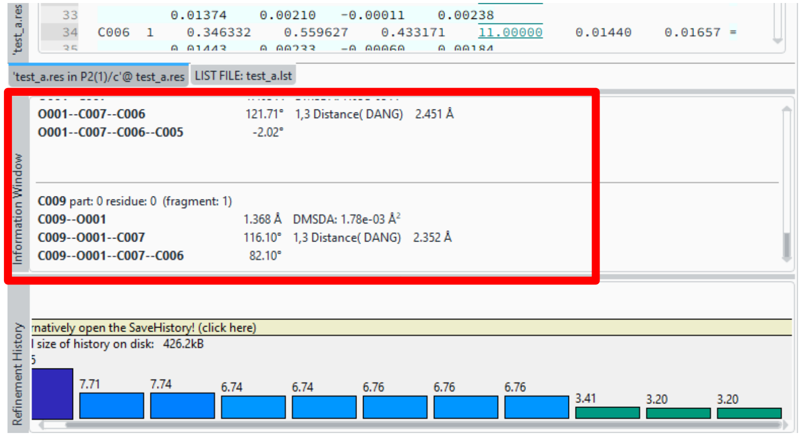

An overview of standard bond lengths and bond types with examples can be found on the next slide.

Analysing the structure

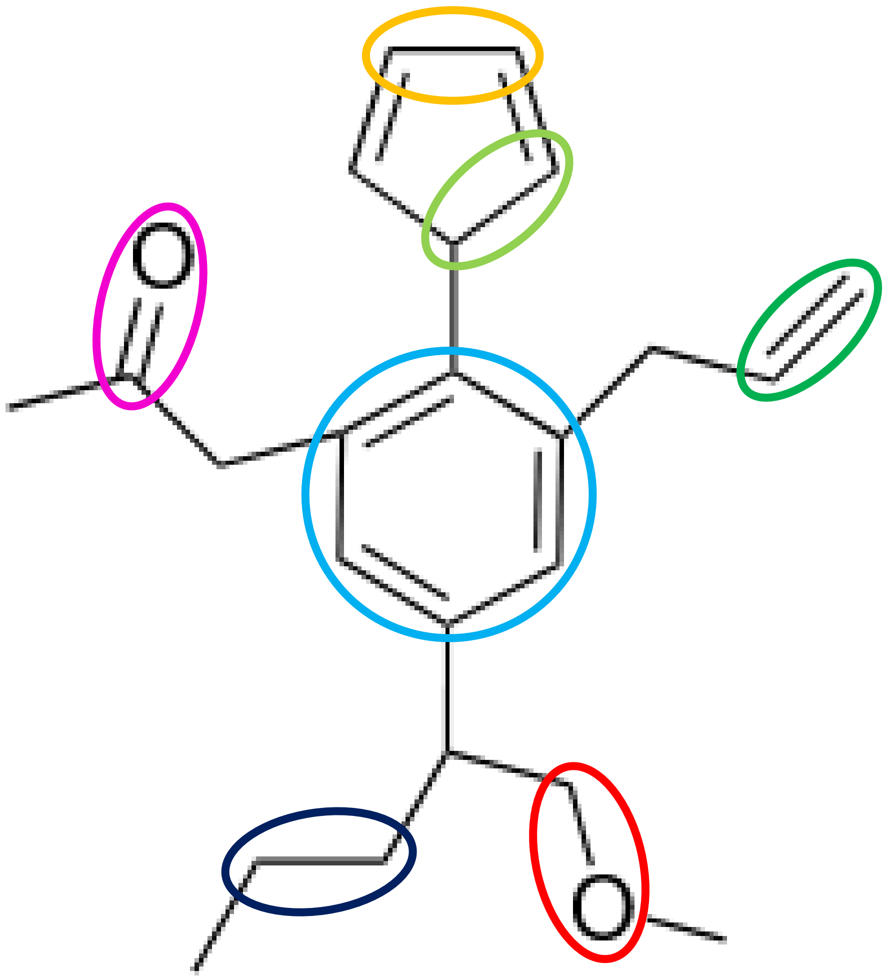

Binding type	Typical bond length in Å
$C(sp^3)–C\ (sp^3)$	1.54
$C(sp^2)–C\ (sp^2)$	1.47
$C(sp^3) – C(sp^2)$	1.50
C=C	1.34
C–C (aromatisch)	1.40
C–O	1.43
C=O	1.20

Analysing the structure

Analysing bond lengths and angles:

By clicking on four bonded atoms, you can also obtain the dihedral or torsion angle, which provides information on the planarity of these four atoms.

You can also display a complete list of all bond lengths and angles (with standard deviations) by selecting the "LIST FILE" option in the top left-hand info window and scrolling to the "Bond lengths and angles" item in the window.

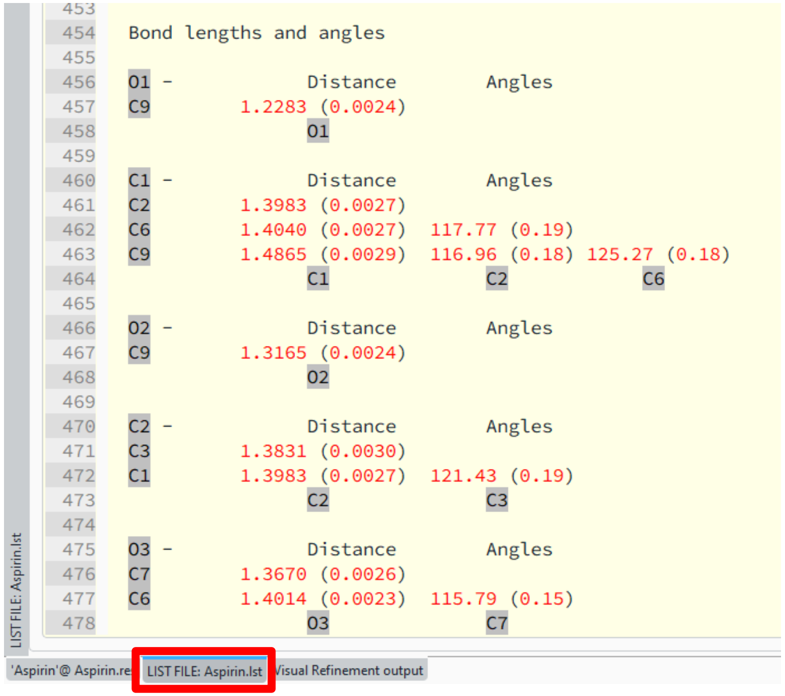

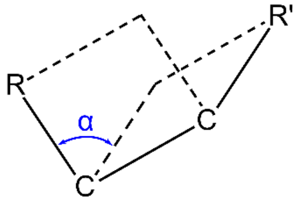

Torsion angle α between R-C-C-R‘

Analysing the structure

- In addition to the bond lengths and angles, the "LIST FILE" contains detailed information for each refinement cycle, error messages and the quality parameters.
- The refinement log displayed in the results window is a rough summary of the most important characteristics and quality criteria of the refinement

```
wR2 =  0.1587 before cycle   4 for    2148 data and    121 /    121 parameters

GooF = S =     1.204;     Restrained GooF =      1.204 for       0 restraints

Weight = 1 / [ sigma^2(Fo^2) + ( 0.0531 * P )^2 +   1.16 * P ]   where  P = ( Max ( Fo^2, 0 ) + 2 * Fc^2 ) / 3


    N      value        esd    shift/esd  parameter

    1     0.84060     0.00300     0.000     OSF

Mean shift/esd =   0.000  Maximum =     0.001 for tors H12A

Max. shift = 0.000 A for H12C        Max. dU = 0.000 for C7
```

Comparison of the results

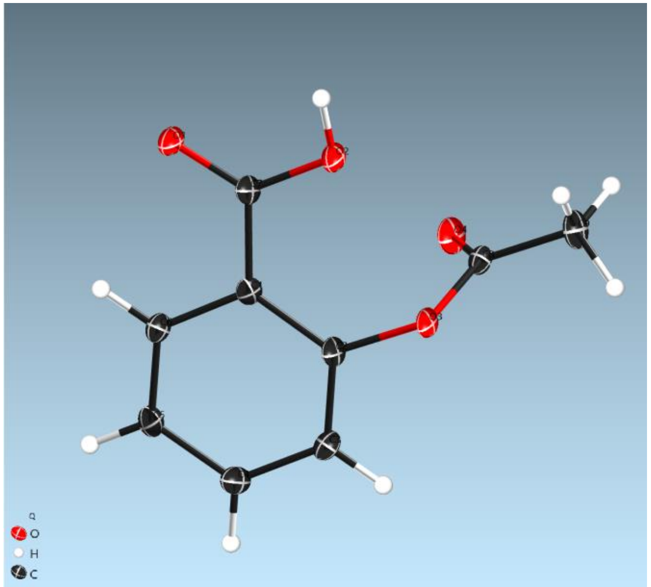

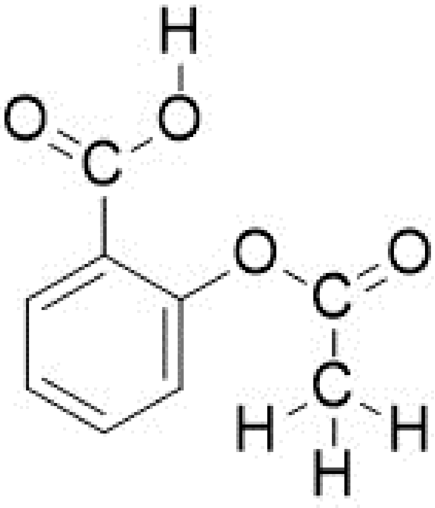

Did you find that out too?

Analysing the structure

The packing of the molecules in the crystal lattice:

You can use the "Pack - Pack Cell" menu command to display the complete contents of the unit cell.

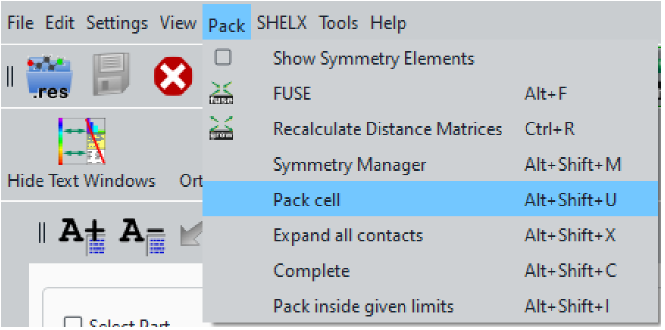

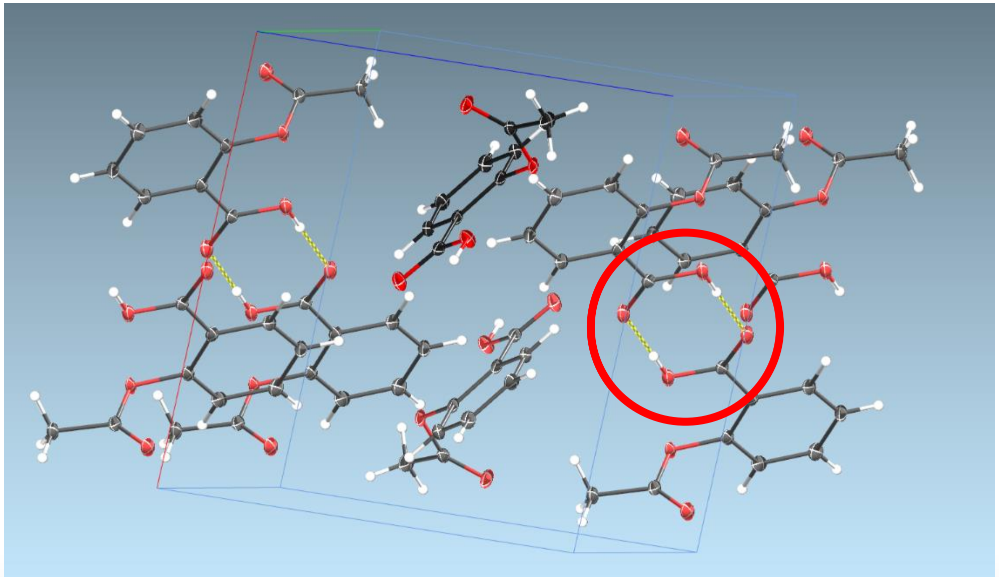

It can be recognised, for example, that two aspirin molecules are always connected to each other by hydrogen bonds.

Produce images

Photo opportunity!

You can obtain a beautiful photo or a rotatable 3D model from the menu list by pressing the two buttons opposite.

Just give it a try!

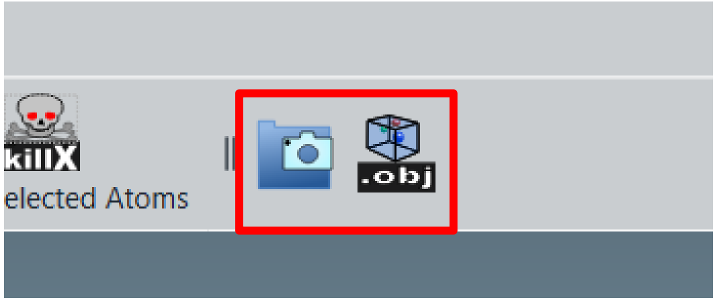

Practising...

Now that you have solved and refined your first structure yourself under guidance, you can work on the other test structures independently.

You will certainly encounter one or two difficulties along the way. Always remember: chemical expertise often helps here, for example with the number of possible bonds of certain types of atoms.

To check your own refinement results, you can compare them with the "sample solutions" stored in the "final" folder. Read the relevant res file into ShelXle, refine it again and compare the result with your last refinement.

Have fun!

Hübschle, C. B., Sheldrick, G. M., Dittrich, B. (2011). ShelXle: a Qt graphical user interface for SHELXL. J Appl Cryst **44**/Pt 6, 1281–1284.
