# Supplementary material for: Crystallography in school
Source: J Appl Crystallogr. 2025 Sep 12;58(Pt 5):1802–9. doi: 10.1107/S1600576725007459 (PMC12502877; doi:10.1107/S1600576725007459)
Supplement: Supplementary file 8 [file j-58-01802-sup8.zip › Further Structures/Final/Solutions of further structures.pdf]

Solutions on further structures

Structure 1:

It is paracetamol

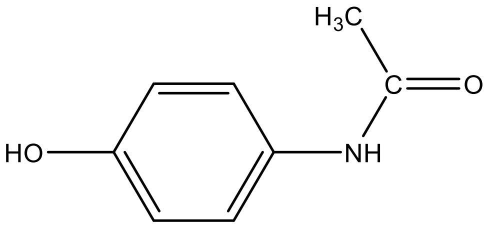

Structure 2:

It is L-Ascorbic acid

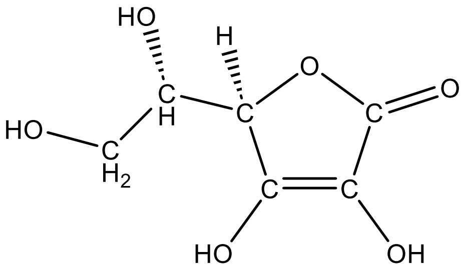

The data sets were measured in the working group of Prof Dr D. Stalke at the Institute of Inorganic Chemistry at Georg-August University.
