# Supplementary material for: Crystallography in school
Source: J Appl Crystallogr. 2025 Sep 12;58(Pt 5):1802–9. doi: 10.1107/S1600576725007459 (PMC12502877; doi:10.1107/S1600576725007459)
Supplement: Supplementary file 8 [file j-58-01802-sup8.zip › Further Structures/Notes on further structures.pdf]

Notes on the ‘Further structures’:

Structure 1:

The molecular formula of this compound is $C_8H_9NO_2$.

Solve and refine the structure and find out which chemical it is. A tip: The substance helps with pain and fever.

Structure 2:

This compound has the molecular formula $C_6H_8O_6$.

An important note: In this structure, you will find two molecules in the asymmetric unit of the unit cell. After solving the structure, you will probably only be able to recognise one of the two molecules ‘straight away’. Try to refine this first and then ‘search’ for the second molecule. If you find individual atoms unconnected to the molecules, please select the ‘SHELX - UNIQUE’ option in ShelXle. This should connect the molecule fragment with the others.

The molecules each contain two asymmetric C atoms. If the warning message ** Absolute structure probably wrong - invert and repeat refinement ** appears during refinement, go to the ‘SHELX’ tab in the menu bar and select the ‘Invert structure’ option and refine again.

This compound is found in citrus fruits and we react to too little intake of this compound with tooth loss.
