# Supplementary material for: Crystallography in school
Source: J Appl Crystallogr. 2025 Sep 12;58(Pt 5):1802–9. doi: 10.1107/S1600576725007459 (PMC12502877; doi:10.1107/S1600576725007459)
Supplement: Supplementary file 9 [file j-58-01802-sup9.pdf]

GEORG-AUGUST-UNIVERSITÄT GÖTTINGEN IN PUBLICA COMMODA SEIT 1737

X-Ray structure determination

Basic level

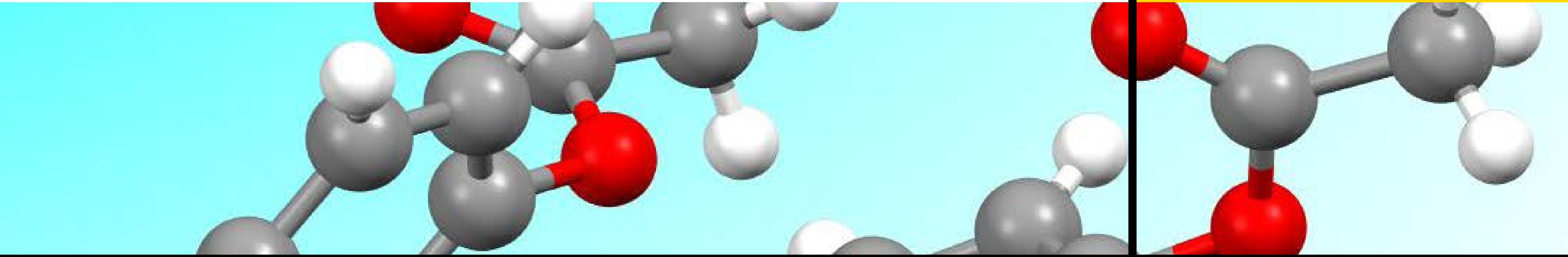

Dr. Erhard Irmer

XLAB – Göttingen experimental laboratory for young people

(erhard.irmer@chemie.uni-goettingen.de)

XLAB Göttingen experimental laboratory for young people

The method of X-ray structure analysis

A „simple“ experiment

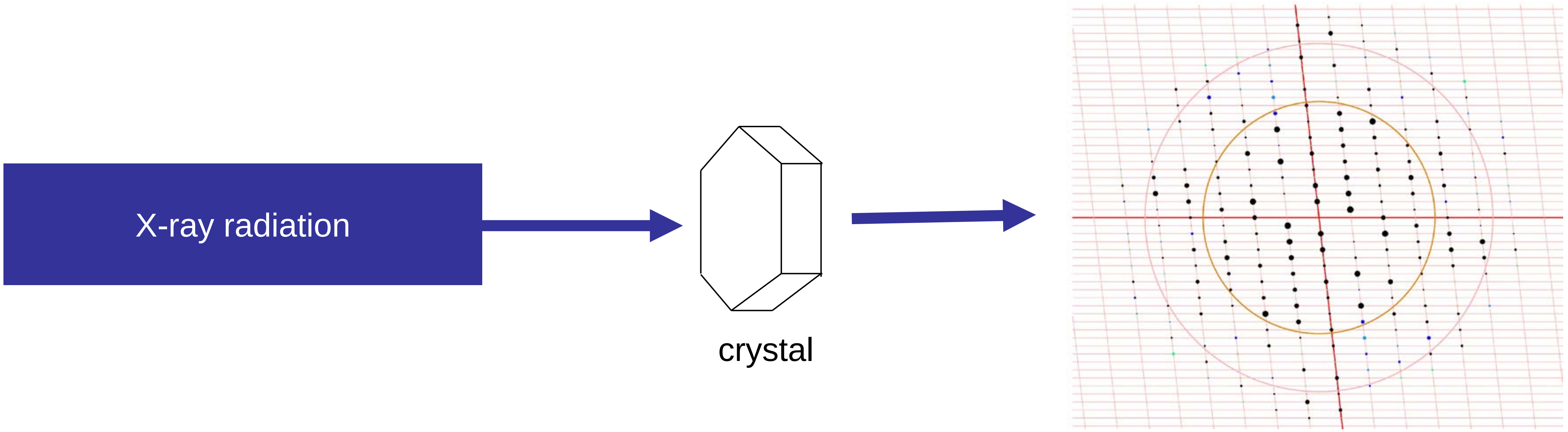

Max von Laue (1912)

The method of X-ray structure analysis

Analogy:
Diffraction of water waves at a double slit

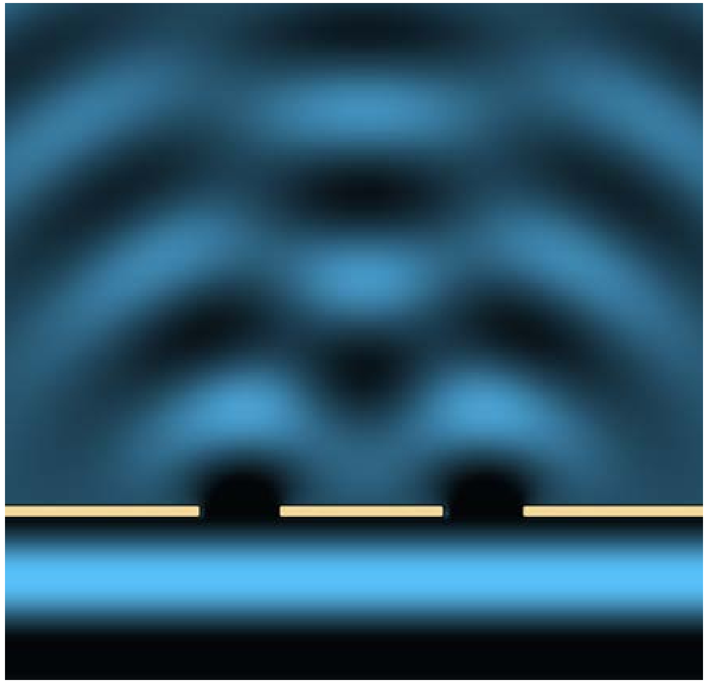

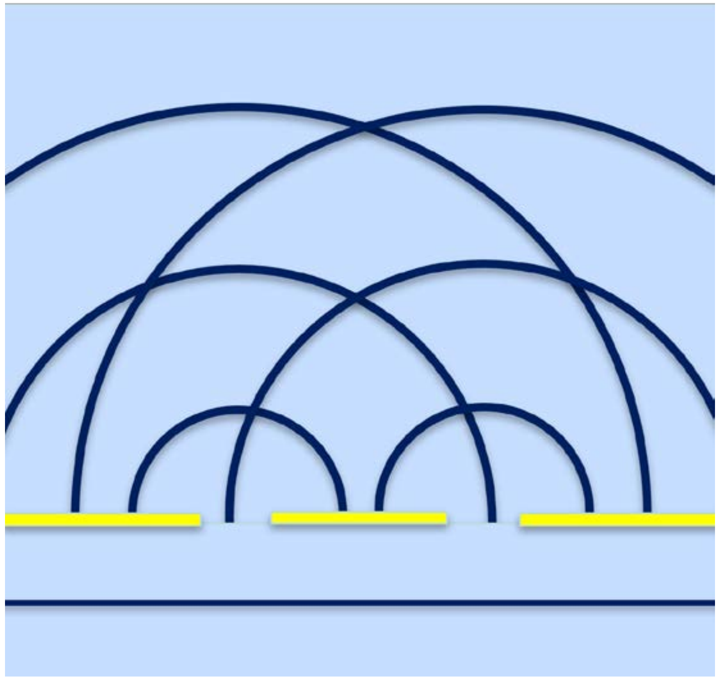

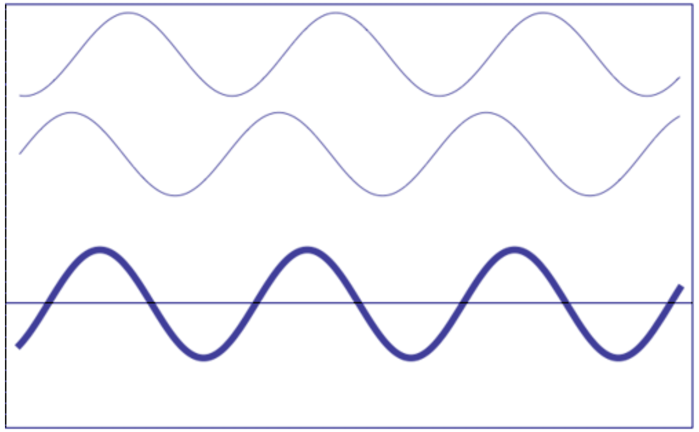

PhET Interactive Simulations, University of Colorado Boulder, licensed under CC-BY-4.0 (https://phet.colorado.edu).

https://www.xtal.iqfr.csic.es/Cristalografia/parte_05-en.html

The method of X-ray structure analysis

Structure of a crystal build up by unit cells

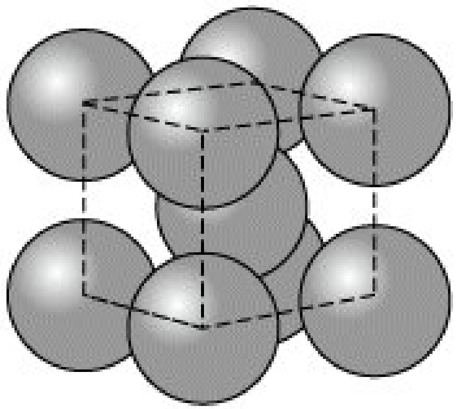

Metal atom structure

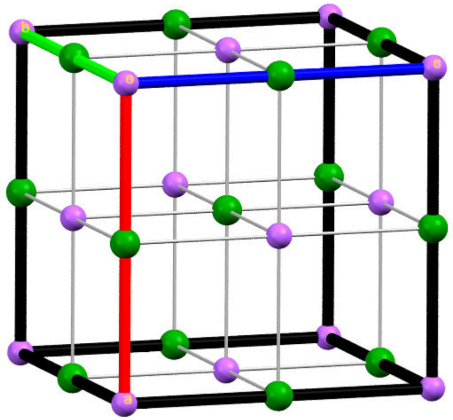

NaCl

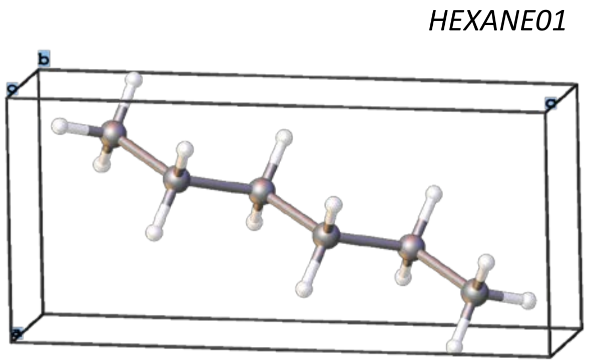

Molecular crystal

XLAB Göttingen experimental laboratory for young people
GEORG-AUGUST-UNIVERSITÄT GÖTTINGEN

The method of X-ray structure analysis

Crystals

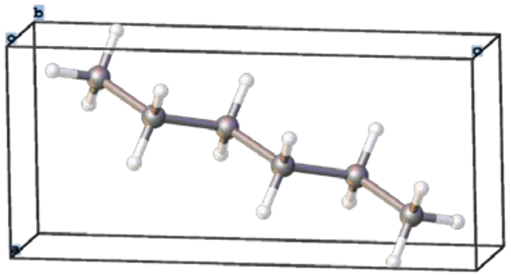

Unit cell with axis length and angles; possibly symmetry elements in the unit cell

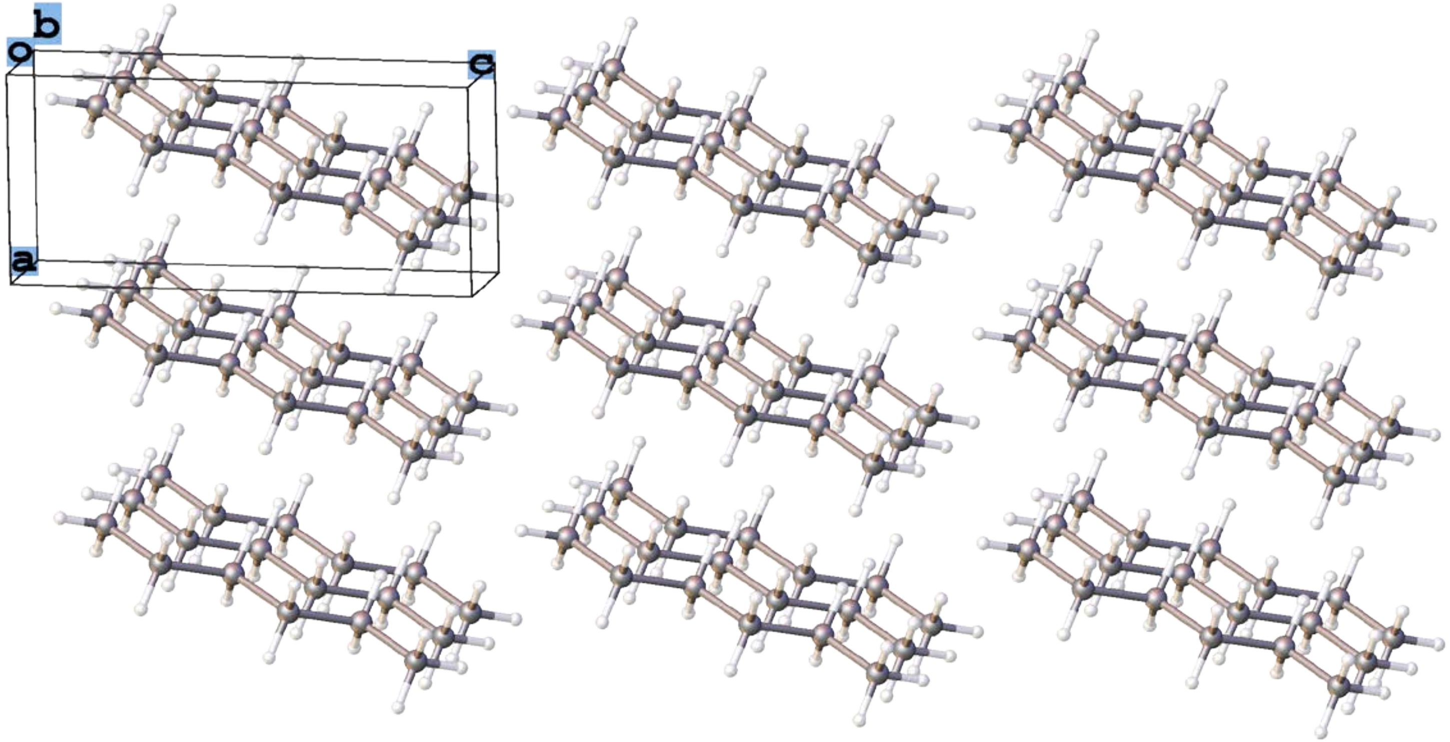

Portion of the **Crystal lattice** as an arrangement of unit cells replicated in all three directions in space

The method of X-ray structure analysis

Crystal structure of
acetylsalicylic acid
(ACSALA01)

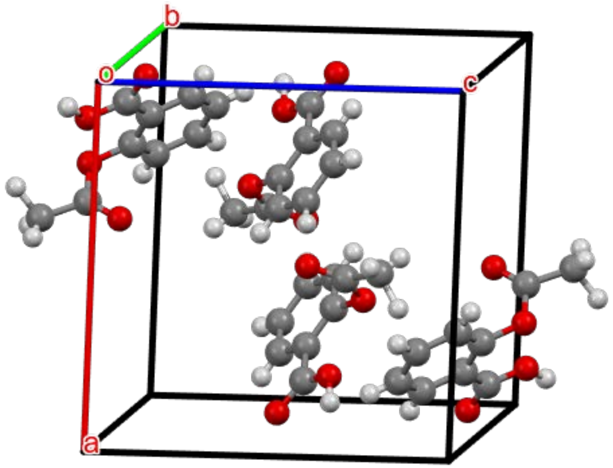

with 4 molecules in the unit cell
(space group $P2_1/c$)

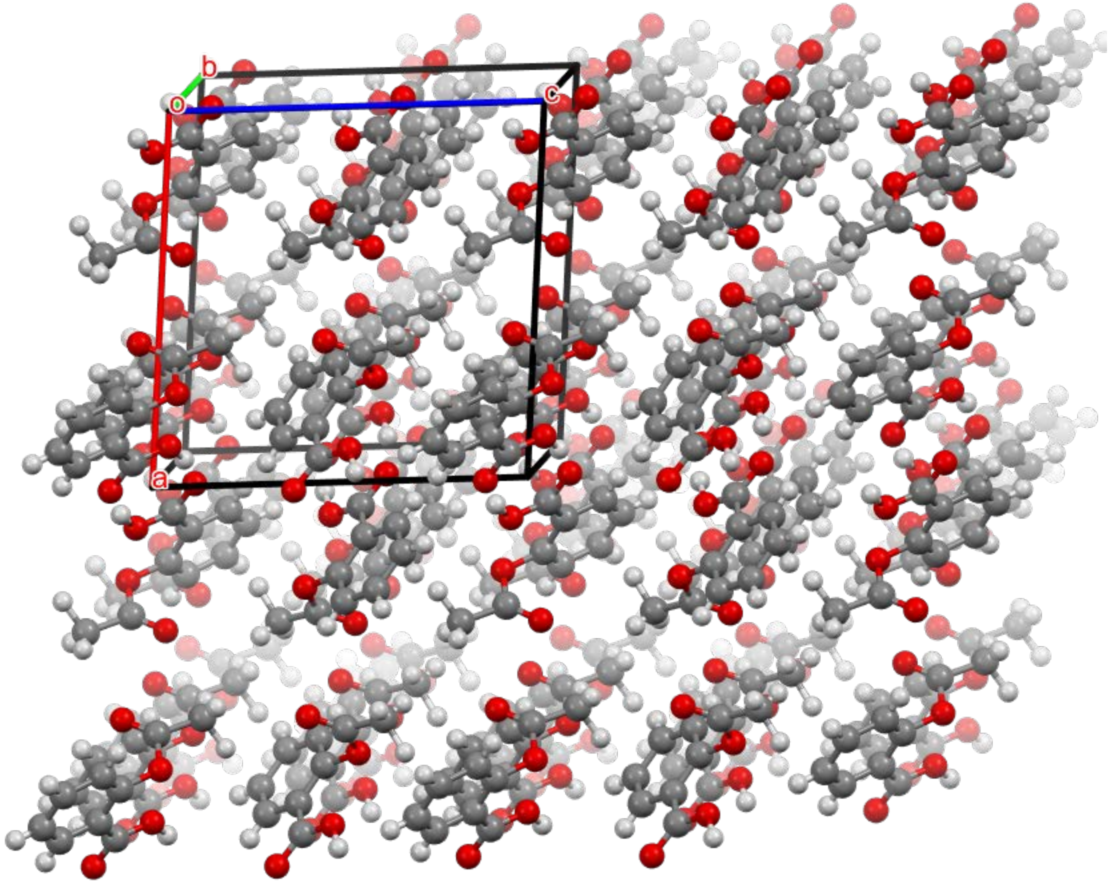

The method of X-ray structure analysis

Single crystals – Curse and blessing

Which crystals could be suitable for X-ray structure analysis?

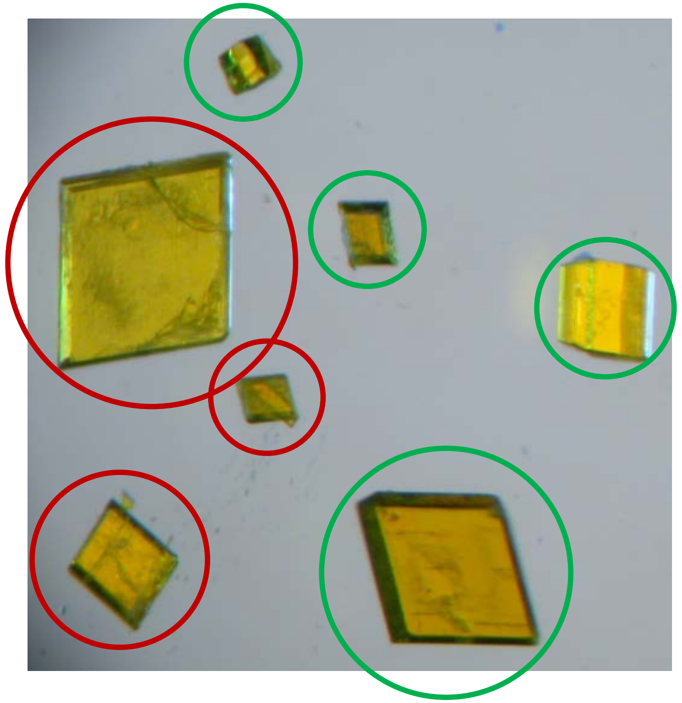

Nico Graw, Universität Göttingen

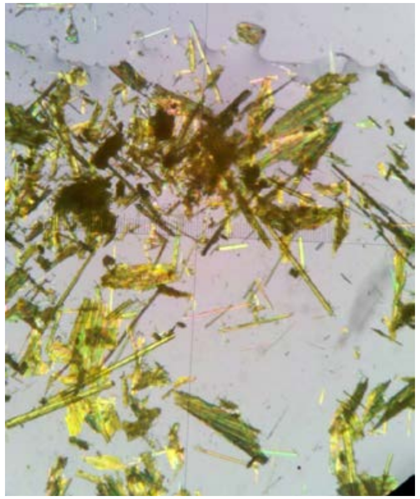

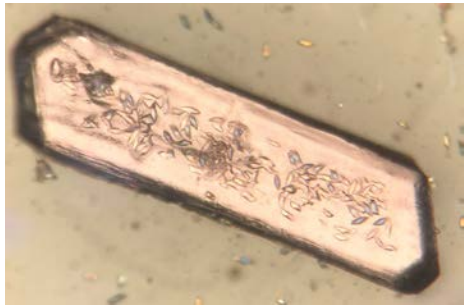

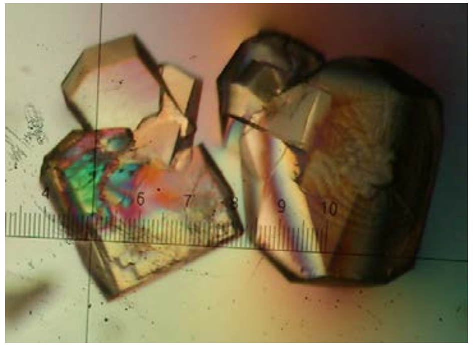

The method of X-ray structure analysis

The X-ray diffractometer

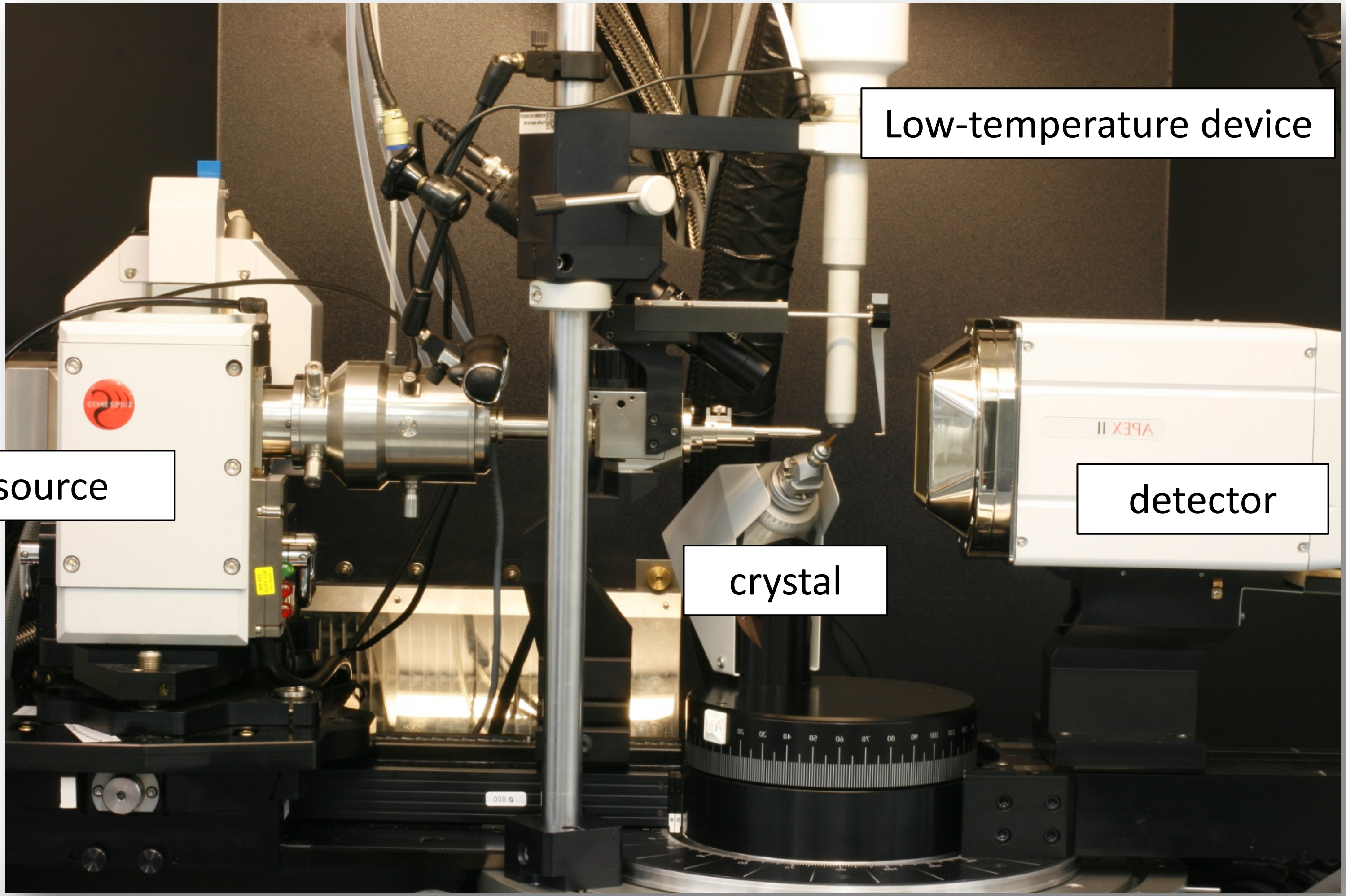

(Prof. Stalke, Uni Göttingen)

The method of X-ray structure analysis

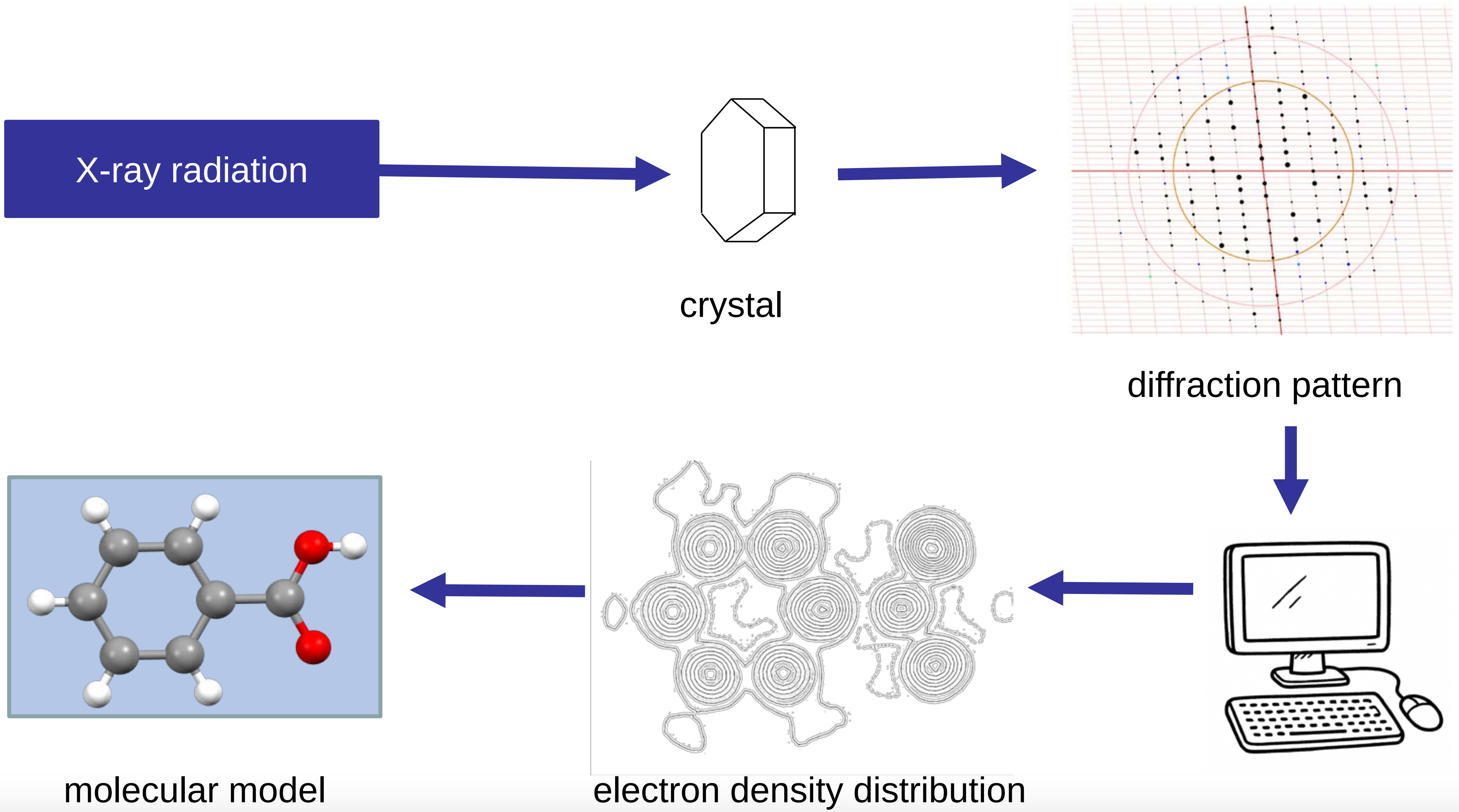

The method of X-ray structure analysis

Results of the X-ray structure analysis

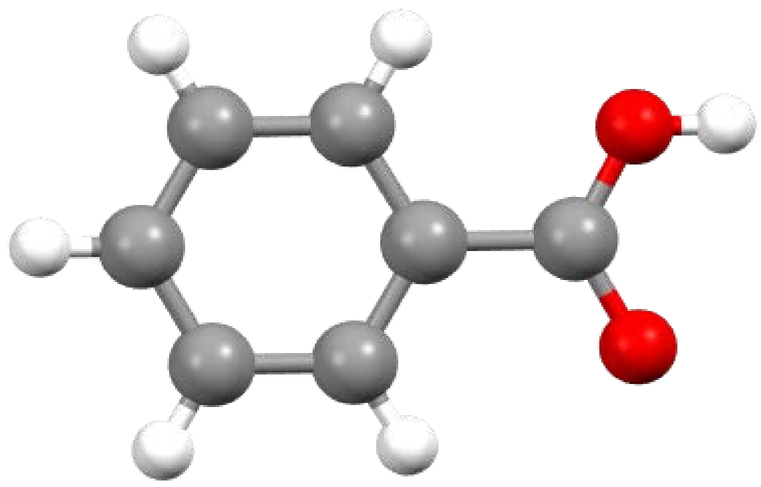

3D-Structure:

- Atom types
  (H atoms usually not "visible" in larger structures),
- atom linkages,
- bond lengths and angles
- Intra- and intermolecular interactions
