# Supplementary material for: Crystallography in school
Source: J Appl Crystallogr. 2025 Sep 12;58(Pt 5):1802–9. doi: 10.1107/S1600576725007459 (PMC12502877; doi:10.1107/S1600576725007459)

GEORG-AUGUST-UNIVERSITÄT
GÖTTINGEN IN PUBLICA COMMODA SEIT 1737

X-Ray structure determination

Advanced level

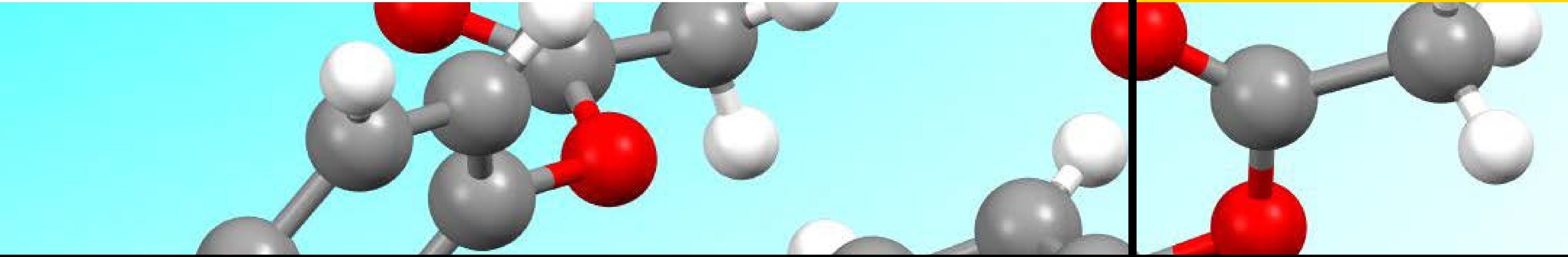

Dr. Erhard Irmer

XLAB – Göttingen experimental laboratory for young people

(erhard.irmer@chemie.uni-goettingen.de)

XLAB Göttingen experimental laboratory for young people

The method of X-ray structure analysis

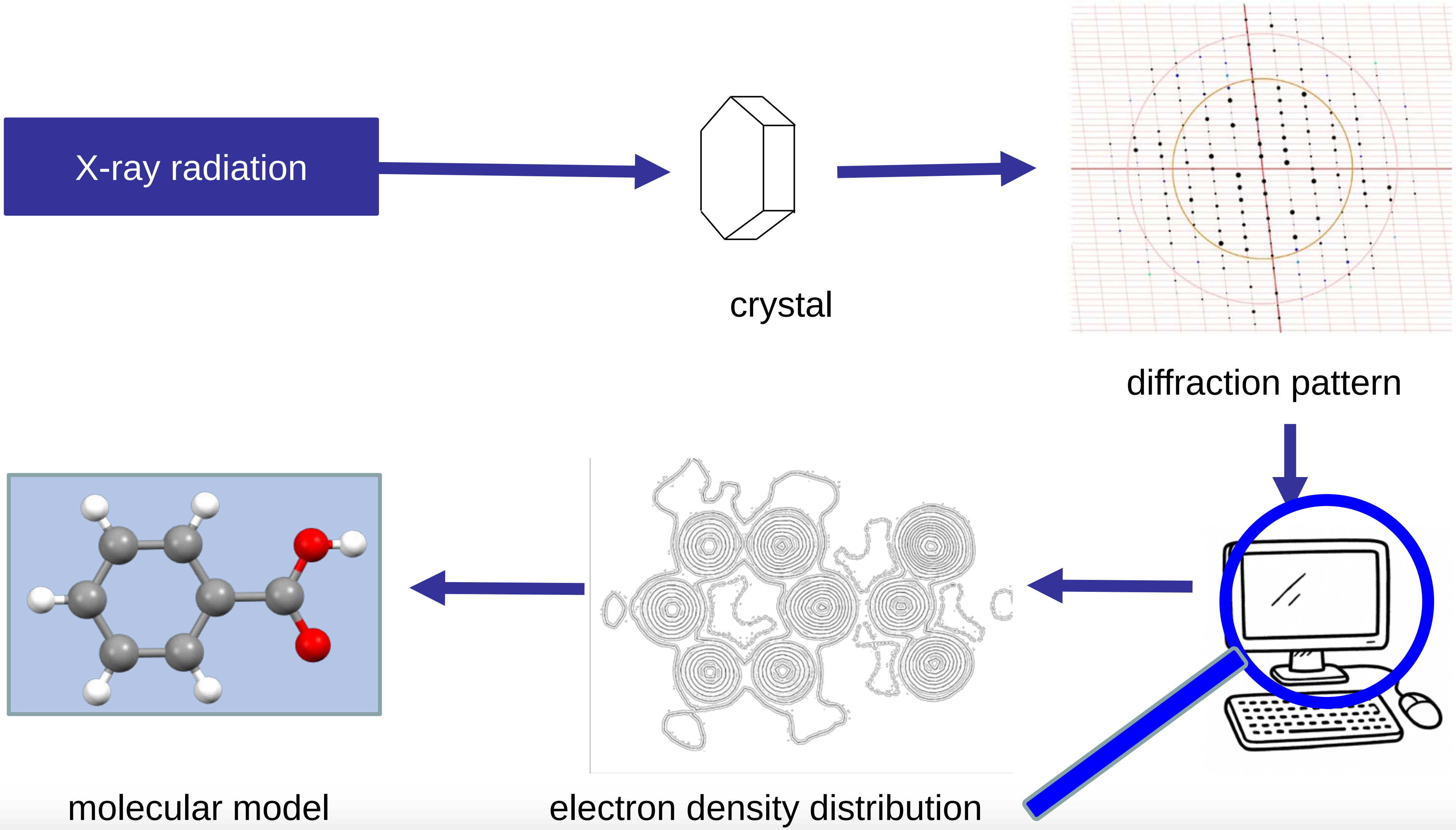

Structure determination - diffraction

from the positions of the diffraction maxima …

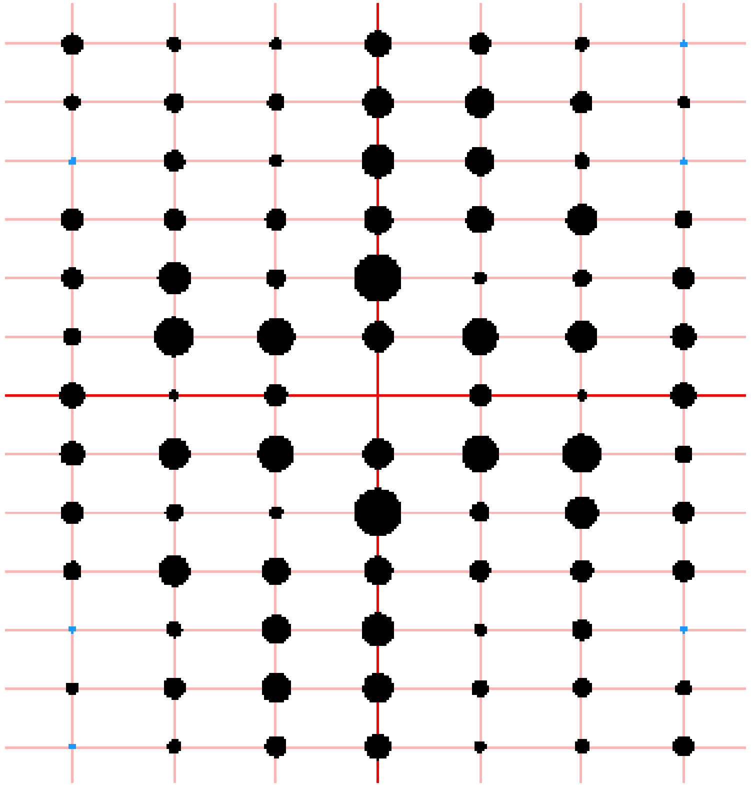

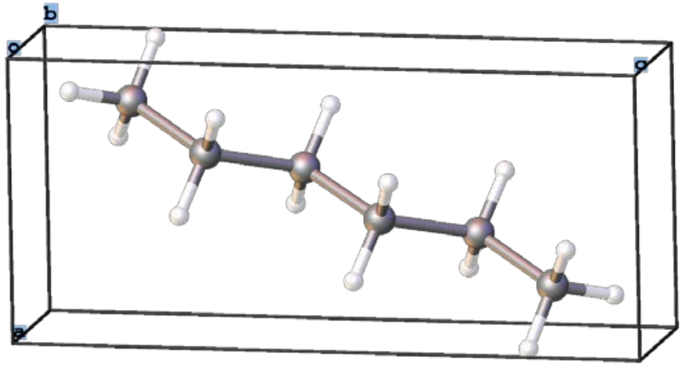

… the dimensions of the unit cell (cell constants) can be obtained

- Unit cell lengths *a*, *b*, *c*
- Angles *α*, *β*, *γ*

R. Herbst-Irmer, Uni-Göttingen

Structure determination - diffraction

The diffraction maxima (" reflections") differ in their intensity.

The intensity of the diffraction maxima contains information about the contents of the unit cell.

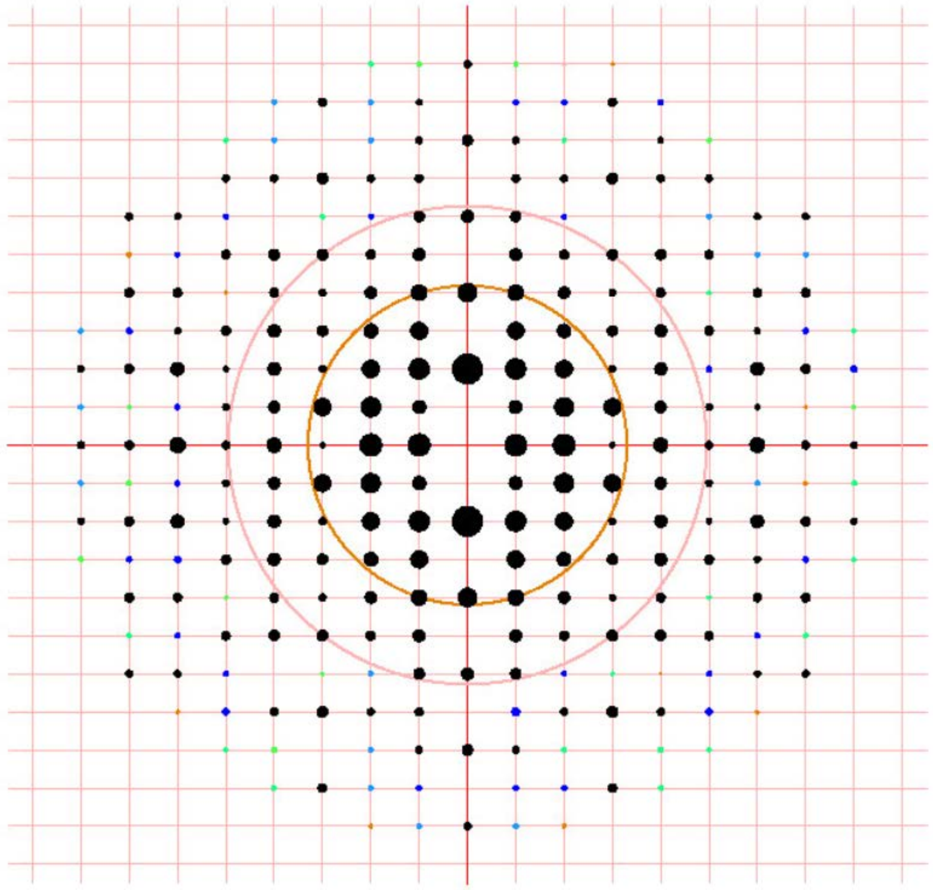

R. Herbst-Irmer, Uni-Göttingen

Structure determination - diffraction

Symmetry in the diffraction pattern and systematic “absences" of diffraction maxima are caused by symmetry in the unit cell.

Example: Glycin (GLYCIN):

2 molecules per unit cell

2-fold screw axis

(space group $P2_1$)

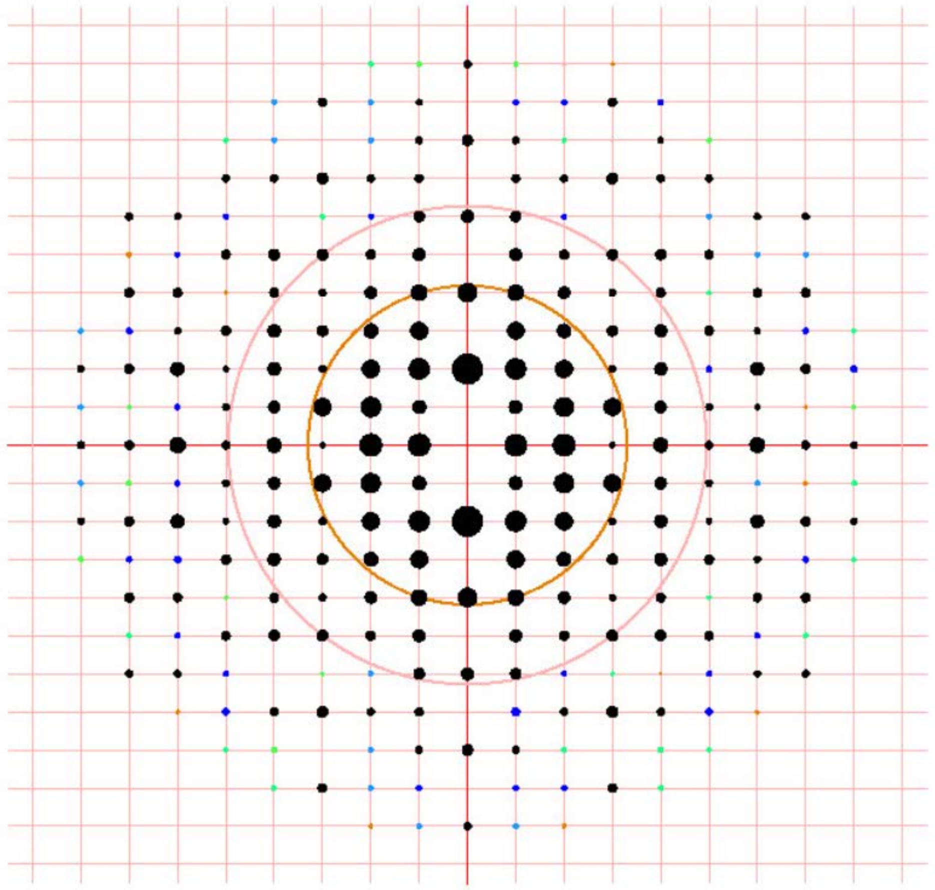

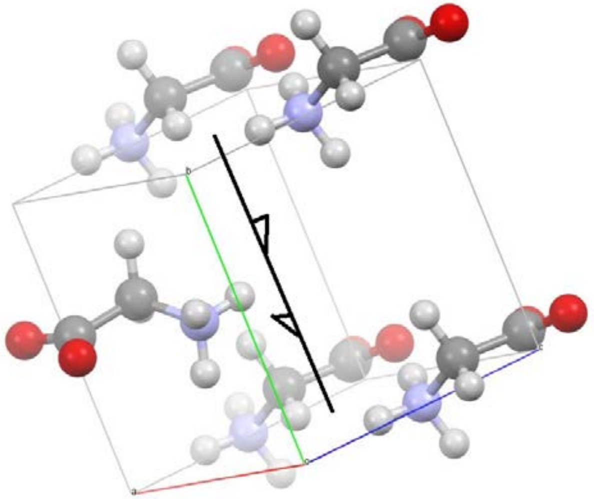

R. Herbst-Irmer, Uni-Göttingen

Diffraction maxima in the 0*kl* lattice plane (glycine structure)

Structure determination - diffraction

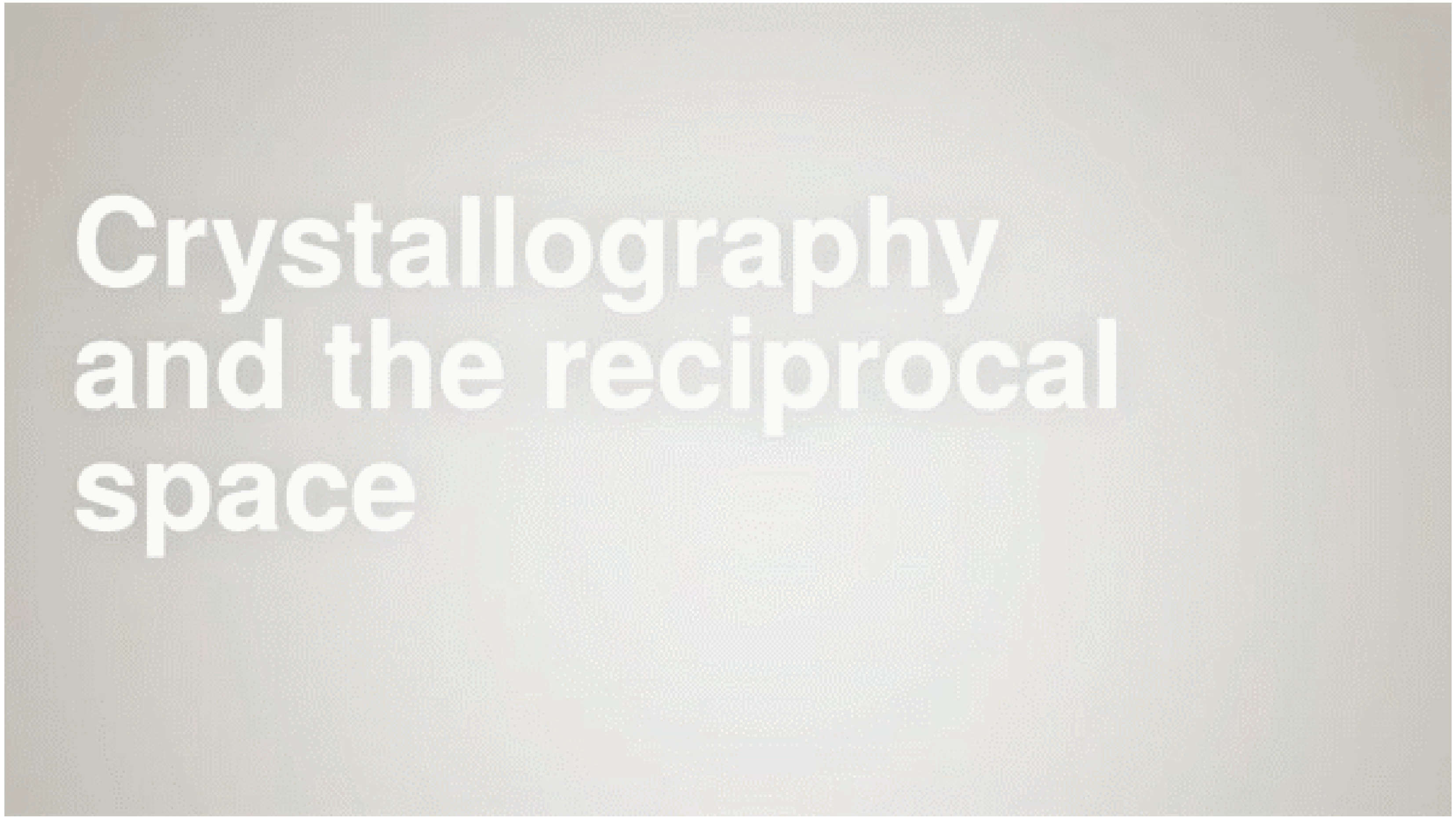

http://www.xtal.iqfr.csic.es/Cristalografia/index-en.html

Structure determination - diffraction

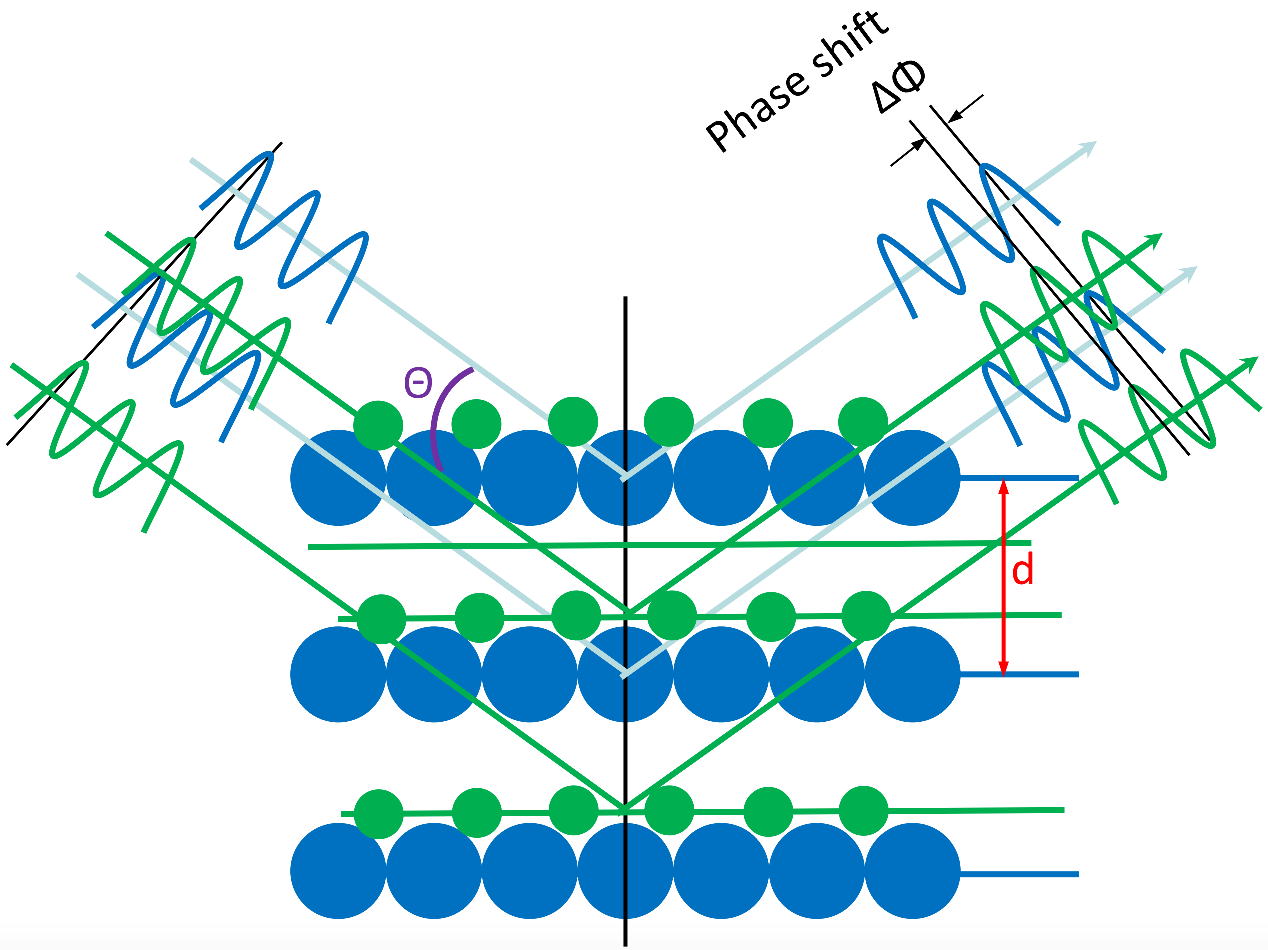

Bragg equation

Conditions for constructive interference :

$n \lambda = 2 d \sin \theta$ $\quad n \in \mathbb{Z}$

Structure determination - diffraction

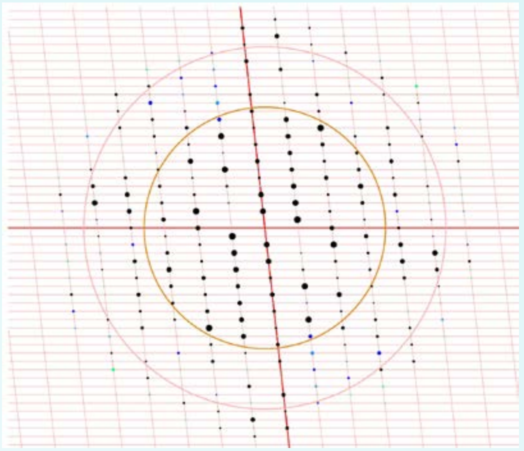

Fourier transformation

Fourier transformation

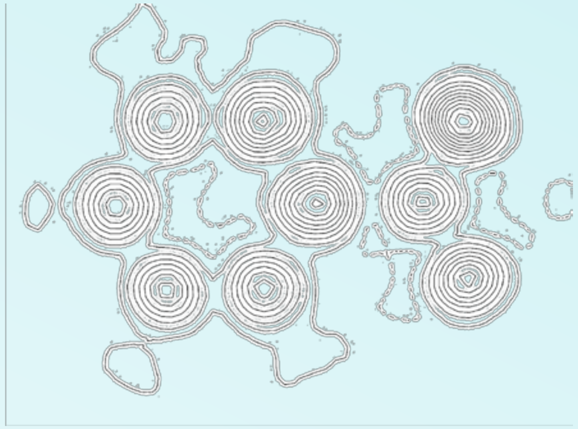

amplitudes + phases of the diffraction maxima (structure factors)

$|F_{obs}|^2 \sim I$

Electron density distribution in the unit cell

Structure determination - solution

Structure solution by direct methods:

- Multiple random sets of start phases
- Using probability relationships between phases and intensities
- „Brute force“ and „trial & error“ often work best
- Chemically reasonable result

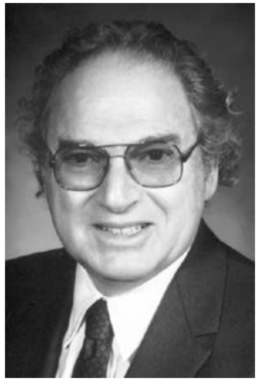

Herbert A. Hauptman (1917-2011)

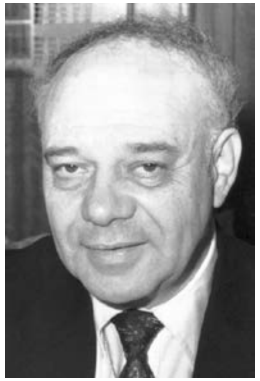

Jerome Karle (1918-2013)

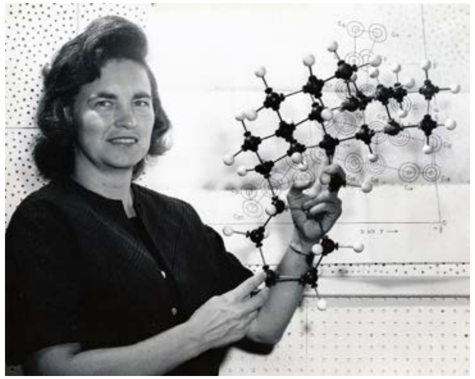

Isabella Karle (1921-2017)

Nobel Prize for Chemistry 1985

dimensions of the unit cell, symmetry information and intensities of the individual diffraction maxima

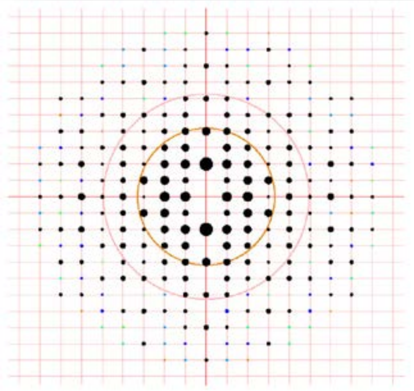

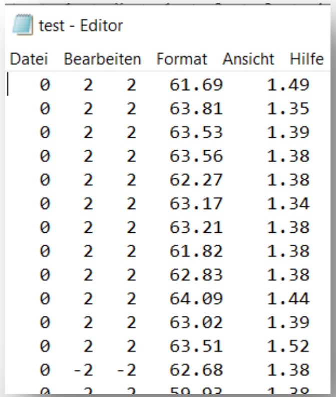

test - Editor

Datei Bearbeiten Format Ansicht Hilfe

0	2	2	61.69	1.49
0	2	2	63.81	1.35
0	2	2	63.53	1.39
0	2	2	63.56	1.38
0	2	2	62.27	1.38
0	2	2	63.17	1.34
0	2	2	63.21	1.38
0	2	2	61.82	1.38
0	2	2	62.83	1.38
0	2	2	64.09	1.44
0	2	2	63.02	1.39
0	2	2	63.51	1.52
0	-2	-2	62.68	1.38
[illegible]	[illegible]	[illegible]	[illegible]	[illegible]

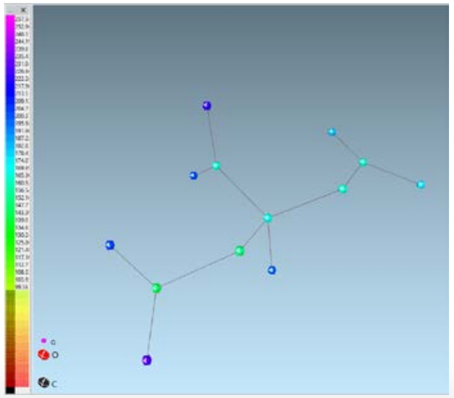

Structure solution with initial electron density peaks

Structure determination - refinement

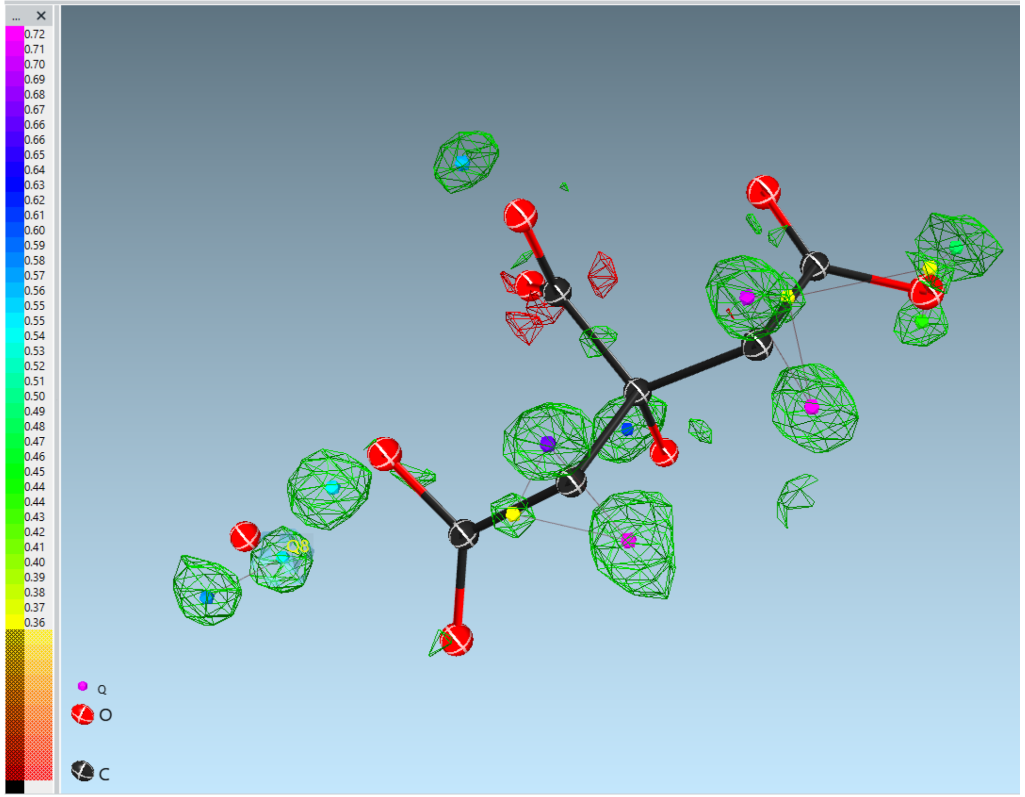

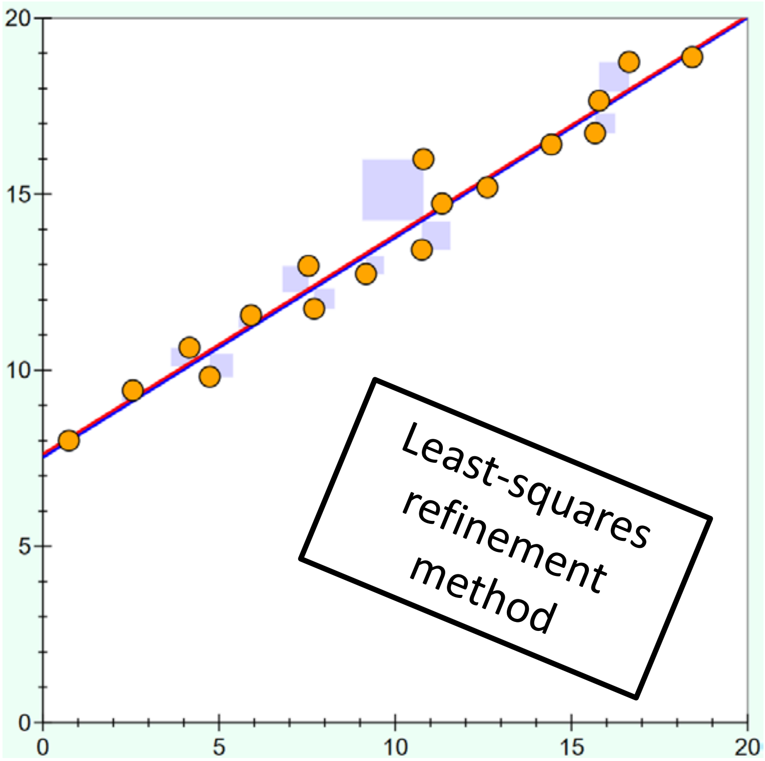

Refinement of the rough model after assignment of the electron density peaks according to atom types against the measured data

$$\Delta = ||F_{obs}| - |F_{calc}||$$

https://phet.colorado.edu/sims/html/least-squares-regression/latest/least-squares-regression_de.html

Structure determination

From the measurement data to the structural image - do it yourself!

dimensions of the unit cell, symmetry information and intensities of the individual diffraction maxima

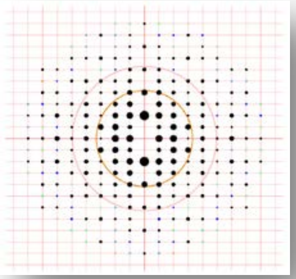

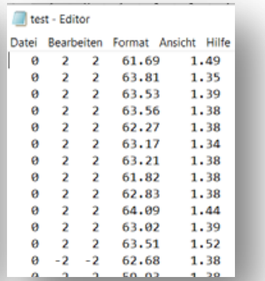

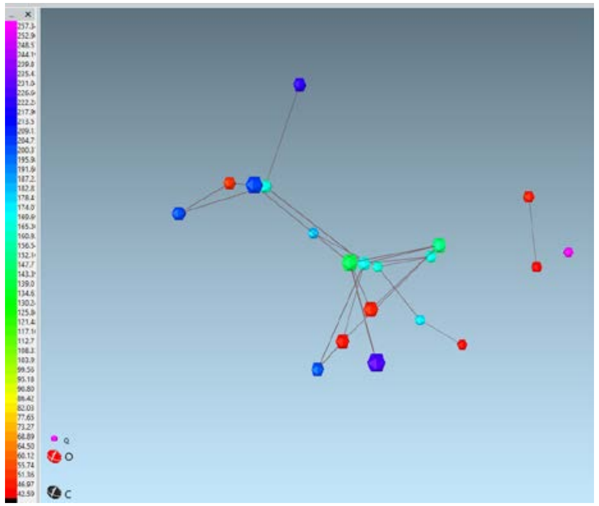

ShelXle

Structural solution with initial electron density peaks

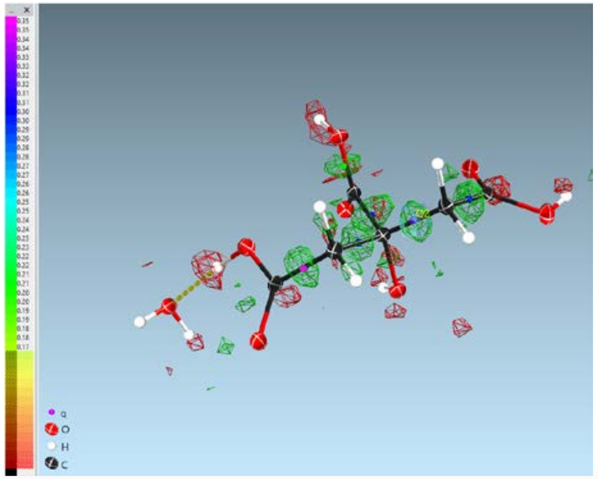

Assignment of atom types and refinement of the atom positions against the observed intensity data

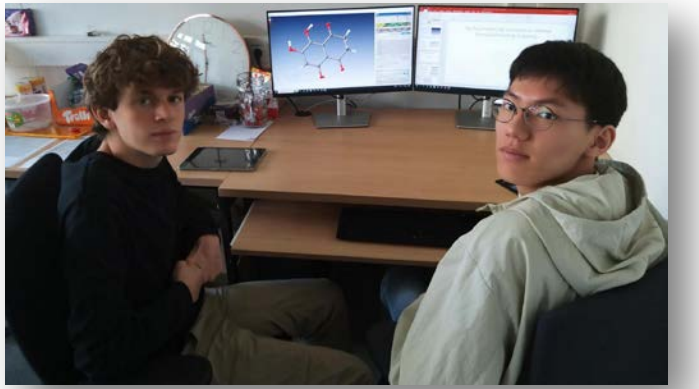

Supplement: Supplementary file 10 [file j-58-01802-sup10.pdf]
