# Supplementary material for: Crystallography in school
Source: J Appl Crystallogr. 2025 Sep 12;58(Pt 5):1802–9. doi: 10.1107/S1600576725007459 (PMC12502877; doi:10.1107/S1600576725007459)
Supplement: Supplementary file 3 [file j-58-01802-sup3.zip › Tutorials/German/Aromatischer Zustand des Benzols.pdf]

GEORG-AUGUST-UNIVERSITÄT GÖTTINGEN IN PUBLICA COMMODA SEIT 1737

XLAB Göttinger Experimentallabor für junge Leute

Die Struktur des Benzol-Moleküls

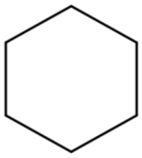

Cyclohexan

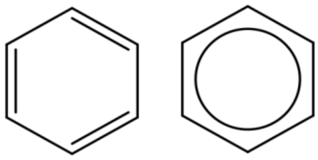

Benzol

1. In der Abbildung sehen Sie Skelettformeln von Cyclohexan und Benzol. Nutzen Sie die Refcodes BENZEN und CYCHEX, um vollständige Strukturformeln der beiden Moleküle zu formulieren. Beschreiben Sie die sichtbaren Unterschiede zwischen den beiden Molekülen.

2. Erklären Sie, weshalb die zweidimensionalen Formeln den Zustand der Moleküle nur unzulänglich wiedergeben.

3. Messen Sie die Bindungslängen der C-C-Einfachbindungen in Alkanen wie ETHANE01 und HEXANE01 und C=C-Doppelbindungen in Alkenen wie ETHLEN10 und COVJON. Anschließend messen Sie die Bindungslängen und Winkel zwischen den Kohlenstoff-Atomen innerhalb des aromatischen Rings in BENZEN und vergleichen Sie die gemessenen Werte mit den Werten für die C-C-Einfach- und C=C-Doppelbindung. Welche Schlüsse lassen sich daraus für die Bindungen im Benzol ziehen?

4. Der Torsionswinkel ist ein Maß für die Planarität („Flachheit“) von ausgewählten Atomen in einem Molekül. Messen Sie den Torsionswinkel für die Kohlenstoff-Atome im Benzol und vergleichen Sie mit Ihren Erwartungen. Beachten Sie: Die CSD nutzt reale Kristallstrukturen. Aufgrund der Packung der Moleküle im Kristall können geringfügig unterschiedliche Werte vorkommen.

5. Nutzen Sie alle bisher erhaltenen Information aus den bisherigen Aufgaben, zeichnen Sie eine Formel für Benzol und erklären die Bindungszustände in dem Molekül.

6. Erklären Sie, warum Benzol im Vergleich zu den Alkenen deutlich weniger reaktiv ist.

7. Untersuchen Sie jetzt die Struktur einer ähnlich aufgebauten Verbindung, des Cyclooctatetraens (ZZSAE01). Handelt es sich auch um einen Aromaten? Informieren Sie sich über die „Hückel-Regel“.

E. Irmer, XLAB – Göttinger Experimentallabor für junge Leute (2023)

Die Struktur des Benzol-Moleküls - Lösungen:

1. Cyclohexan C_6H_{12} ist nicht eben und kommt in einer Sessel-Konformation vor. Benzol C_6H_6 ist ein ebenes Molekül, wie die Formel es darstellt.

2. Die 2D-Darstellung impliziert, dass beide Moleküle eben (planar) sind. Die 3D-Struktur zeigt die tatsächliche räumliche Gestalt der Moleküle. Benzol ist planar, Cyclohexan nicht.

3. Die Bindungslängen für die C-C-Einfachbindung im Ethan beträgt 1,53 Å(153 pm), im Hexan 1,52 Å (152 pm). Die C=C-Bindungslänge im Ethen beträgt 1,31 Å (131 pm), im Cyclohexen 1,31 – 1,33 Å 131-133 pm). Benzol besitzt eine C-C-Bindungslänge von 1,39 Å (139 pm) und einen Bindungswinkel von 119,5°. Die Bindungen sind länger als die C=C-Doppelbindung, aber kürzer als die C-C-Einfachbindung. Man sieht, dass eine Beschreibung mit abwechselnden Einfach- und Doppelbindungen das Molekül nicht richtig wiedergibt. Die Bindungslängen liegen zwischen Einfach- und Doppelbindungen, der gemessene Bindungswinkel ist sehr nahe am theoretischen Wert von 120° (der Innenwinkel in einem regelmäßigen Sechseck).

4. Der Torsionswinkel beträgt 1,4°. Benzol ist ein planares Molekül, der Torsionswinkel sollte idealerweise 0° betragen.

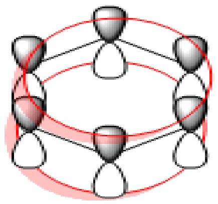

5. Jedes Kohlenstoff-Atom besitzt ein p-Orbital, welches mit einem einzelnen Elektron besetzt ist. Diese Orbitale überlappen und die Elektronen werden über alle sechs Kohlenstoff-Atome delokalisiert. Die π-Bindung erstreckt sich über das gesamte Molekül.
6. Die Delokalisierung der π-Elektronen stabilisiert das Benzol-Molekül. Es finden keine Additionsreaktionen statt wie bei den Alkenen.
7. Beim Cyclooctatetraen handelt es sich nicht um ein planares Molekül, sondern dieses Molekül liegt in einer Wannenform vor. Die C-C-Bindungsabstände unterscheiden sich mit 1,33 Å (133 pm) für die Doppelbindung und 1,47 Å (147 pm) für die Einfachbindung deutlich. Die Hückel-Regel besagt, dass (4n+2) π-Elektronen eine Bedingung für ein ringförmiges aromatisches System darstellt. Benzol besitzt 6 π-Elektronen aus drei Doppelbindungen, das nächsthöhere aromatische System müsste 10 π-Elektronen aufweisen. Cyclooctatetraen hat aber nur 8 π-Elektronen im Ring und kann deshalb kein aromatisches System bilden.

Verändert nach: https://www.ncl.ac.uk/nes/outreach/chemistry/resources/ccdc/#d.en.606483

E. Irmer, XLAB – Göttinger Experimentallabor für junge Leute (2019)
