# Supplementary material for: Crystallography in school
Source: J Appl Crystallogr. 2025 Sep 12;58(Pt 5):1802–9. doi: 10.1107/S1600576725007459 (PMC12502877; doi:10.1107/S1600576725007459)
Supplement: Supplementary file 3 [file j-58-01802-sup3.zip › Tutorials/German/Mercury Eine Einführung.pdf]

Mercury – Eine Einführung

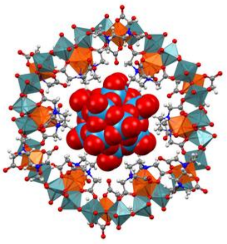

Zugriff auf den CSD Teaching Subset:

Starten Sie das Programm „Mercury“ und öffnen Sie den Teaching Subset durch „CSD-Community“ – „Open Teaching Database“

Rechts erscheint ein Bereich „Structure Navigator“ mit den Refcodes der im Teaching Subset hinterlegten Kristallstrukturen

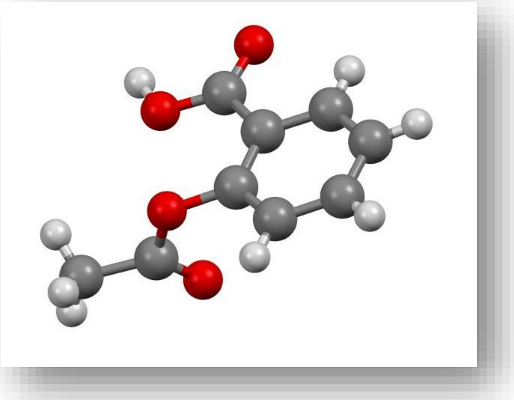

Laden Sie die Struktur von **Acetylsalicylsäure** (ACSALA01): Dazu scrollen Sie entweder zu dem Eintrag oder geben die ersten Buchstaben in das Suchfeld oben im „Structure Navigator“ ein und klicken auf den Refcode. Im linken Fenster erscheint ein Molekülmodell der Struktur. In welcher Weise das Molekül dargestellt wird, hängt von den vorgewählten Einstellungen ab.

Grundlegende Bewegungsmöglichkeiten:

Linke Maustaste gedrückt halten und bewegen: Drehen in alle drei Raumrichtungen

Mittlere Maustaste gedrückt halten und bewegen: Verschieben in zwei Richtungen

Rechte Maustaste gedrückt halten und bewegen: Zoomen

Eine kontrollierte Bewegung des Moleküls ist auch über die Menüs „x-, x+, y-, y+,…“ usw. möglich.

Verschiedene Darstellungsmöglichkeiten:

Über den Menüpunkt „Styles“ können verschiedene Darstellungsweisen gewählt werden:

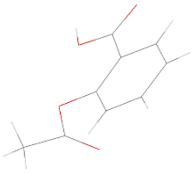

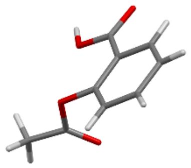

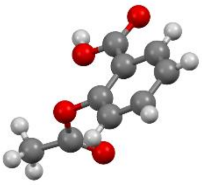

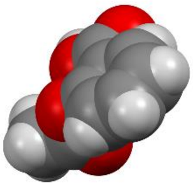

Wireframe	Drahtmodell (nur Bindungen)
Capped Sticks	Stäbchenmodell (nur Bindungen)
Ball and Stick	Kugel-Stab-Modell (Atome als Kugeln, Bindungen als Stäbchen)
Spacefill	Kalottenmodell
Ellipsoid	zeigt bei manchen Strukturen an, in welche Richtung die Atome im Kristall schwingen (die PF_6^--Ionen in FA-MZEZ)
Polyhedral	Zeigt Koordinationspolyeder

Farbe der Atome:

Wählen Sie „Colour“ „by Element“, so werden die Atome in dem typischen CPK-Farbschema dargestellt:

Dunkelgrau	Kohlenstoff
Hellgrau	Wasserstoff
Rot	Sauerstoff
Blau	Stickstoff

Die Atom-Namen können unter „Display Options“ im unteren Bildbereich angezeigt werden „Label atoms“. Ebenso können H-Atome ein- oder ausgeblendet werden: „Show hydrogens“

Messungen der Geometrie

Es können Abstände, Winkel und Torsionswinkel (Winkel zwischen zwei Ebenen) bestimmt werden.

Für **Bindungsabstände** ist es am Einfachsten, eine Liste der Bindungen aufzurufen. Dazu wählt man im unteren Bildschirmteil (Display Options) unter „More info“ den Punkt „Bond list“ aus. Hier werden alle Bindungslängen ausgegeben, teilweise mit experimentell bestimmten Standardabweichungen in Klammern). Der Bindungstyp (Type) wurde von den Autoren entsprechend der vorgegebenen Strukturformel gesetzt und in der Molekülgrafik ebenfalls angezeigt. Unter „Display“ „Styles“ kann durch Entfernen des Hakens bei „Display Bond Types“ und „Display Aromatic Rings“ der Bindungstyp ausgeblendet werden.

Um eine einzelne **Bindungslänge** zu bestimmen, wählt man im oberen Bildschirmteil unter „Picking Mode“ den Punkt „Measure Distances“ aus und klickt auf die beiden Atome, deren Abstand ich erhalten möchte.

- *Untersuchen Sie die Bindungsabstände der C-C-Bindungen im Benzolring und vergleichen Sie die C-O-Einfachbindungslängen mit der C=O-Doppelbindungslänge.*

Bindungswinkel erhält man genauso, indem man unter „Picking Mode“ stattdessen „Measure Angles“ auswählt und drei Atome anklickt. Es wird der Winkel um das mittlere Atom ausgegeben.

- *Untersuchen Sie die Winkel im Benzolring.*

Entsprechend lassen sich **Torsionswinkel** durch „Measure Torsions“ bestimmen. Hierbei werden vier Atome angeklickt. Überprüfe die Planarität der Atome um die C=O-Gruppe.

- *Überprüfen Sie die Planarität der Benzolring-Atome und der Atome rund um die C=O-Gruppe.*
- *Zeichnen Sie eine vollständige Elektronenpaarformel (Lewisformel) des ASS-Moleküls.*

Kristallpackung

Unter „Display Options“ im unteren Bildschirmbereich kann die **Elementarzelle** ein- oder ausgeblendet werden „Show cell axes“.

Durch Anklicken von „Packing“ werden die in der Elementarzelle enthaltenen Moleküle eingeblendet, beim ASS sind es vier Moleküle (evtl. werden mehr Moleküle dargestellt, wenn die Packungs-Einstellungen bereits verändert wurden).

- *Finden Sie heraus, wie viele ASS-Moleküle in der Elementarzelle vorhanden sind.*

Wählen Sie in der oberen Menüleiste unter „Calculate“ - „Packing and Slicing“ aus, wie viele Elementarzellen dargestellt werden. Vergrößern Sie unter „a:“ den rechten Wert durch mehrfaches Klicken des Pfeils nach oben oder durch Klicken auf „+0.5“. Der **Packungsausschnitt** wird entsprechend gewählt. Vorsicht bei sehr großen Ausschnitten: Die Rechenleistung kann überschritten werden. Zurücksetzen ist jederzeit über „Reset“ möglich.

Weist das Molekül eine interne Symmetrie auf, kann es sein, dass die Elementarzelle nicht das gesamte Molekül enthält, sondern nur die **asymmetrische Einheit**. Dies kann durch Anklicken von „Asymmetric Unit“ im unteren Bildschirmteil überprüft werden.

- *Welche Molekülteile sind in der Benzol-Struktur (BENZEN02) durch Symmetrie verbunden.*

Gerade beim Betrachten von Kristallpackungen ist es hilfreich, den Kristallausschnitt entlang bestimmter Zellachsen zu betrachten. Dazu finden sich in der oberen Bildschirmhälfte die Auswahlbuttons „a“, „b“ und „c“.

Zwischenmolekulare Kontakte

Es können alle Abstände angezeigt werden, die unterhalb der Summe der van-der-Waals-Radien der Atome liegen. Dazu klickt man unter „Display Options“ auf „Short Contacts“. Mit „H-Bonds“ werden nur solche Abstände angezeigt, bei denen O-, S- und N-Atome beteiligt sind. Beachten Sie dazu das Tutorial „Wasserstoff-Brücken“.

- *Finden Sie heraus, ob es H-Brücken zwischen den ASS-Molekülen gibt. Evtl. müssen Sie zuerst das Limit bei der Kristallpackung auf 1 setzen und dann selektiv einzelne Moleküle im „Picking Mode“ mithilfe von „Expand contacts“ durch Anklicken der fraglichen Atome anzeigen lassen.*

Autor: E. Irmer, XLAB – Göttinger Experimentallabor für junge Leute (2019)
