# Supplementary material for: Crystallography in school
Source: J Appl Crystallogr. 2025 Sep 12;58(Pt 5):1802–9. doi: 10.1107/S1600576725007459 (PMC12502877; doi:10.1107/S1600576725007459)
Supplement: Supplementary file 3 [file j-58-01802-sup3.zip › Tutorials/German/VSEPR.docx]

**VSEPR-Modell**

1. Rufen Sie in Mercury den RefCode [TBUCBD10](../../CIF/Others/TBUCBD10.cif) auf. Finden Sie in der Struktur die tertiären Butylgruppen. Bestimmen Sie die Winkel um das tertiäre Kohlenstoff-Atom und vergleichen Sie diese mit dem Tetraeder-Winkel.
2. Rufen Sie die Struktur des Tetramethylethen (2,3-Dimethylbut-2-en) ([PAPVAD](../../CIF/Alkenes/PAPVAD.cif)) auf und bestimmen Sie die Winkel innerhalb des Moleküls. Erklären Sie, warum ein Winkel deutlich kleiner ist als die beiden anderen. Überprüfen Sie mithilfe des Torsionswinkel, ob das Molekül planar ist.
3. Welche Geometrie erwarten Sie für das But-2-in? Überprüfen Sie Ihre Vermutung anhand der Kristallstruktur ([JUFDUJ](../../CIF/Alkynes/JUFDUJ.cif)).
4. Als nächstes untersuchen Sie bitte die Kristallstruktur des Trimethylamin-Moleküls ([CEKGUU01](../../CIF/Amines/CEKGUU01.cif)), indem Sie die Winkel um das N-Atom bestimmen. Vergleichen Sie mit der Struktur des Tetramethylammonium-Kations ([YALROS](../../CIF/Others/YALROS.cif)) und mit der des Ammoniaks ([KATLAT](../../CIF/Others/KATLAT.cif); in dieser Struktur wurde die Lage der H-Atome relativ genau bestimmt).
5. Bei mehr als vier Bindungspartnern um ein Zentralteilchen herum ergeben sich andere geometrische Körper. Untersuche das PF_6_^-^-Anion ([WINFAA](../../CIF/Others/WINFAA.cif)) und den Pentacarbonyleisen-Komplex ([FOJBOV01](../../CIF/Others/FOJBOV01.cif)) und beschreibe die Geometrien möglichst genau.
6. Betrachte die Struktur der folgenden Verbindung: [RIZMIW](../../CIF/Others/RIZMIW.cif). Zeichne eine Strukturformel mit allen freien Elektronenpaaren für die Verbindung. Erkläre, von welcher Geometrie sich diese Struktur ableitet.
7. Kehren Sie noch einmal zurück zur Struktur [TBUCBD10](../../CIF/Others/TBUCBD10.cif) aus Aufg. 1). Konzentrieren Sie sich jetzt auf den Cyclobutadien-Ring. Bestimmen Sie die Bindungswinkel und die Bindungslängen innerhalb des Rings. Wie passen dies zu den nach dem VSEPR-Modell erwarteten Bindungslängen und Winkeln? Man hat diese Struktur auch noch bei -150°C gemessen. Jetzt beobachtet man C-C-Bindungslängen von 1,44 Å und 1,53 Å. Versuche zu erklären, warum bei tiefer Temperatur die Unterschiede zwischen Einfach- und Doppelbindung größer werden.

*E. Irmer, XLAB – Göttinger Experimentallabor für junge Leute (2023)*

**Lösungen:**

1. Im Molekül des Tetra-tert-butyl-cyclobutadien kommen vier tert-Butylgruppen vor, davon sind zwei symmetrieunabhängig („Display Options“ – „Asymmetric Unit“).

Die C-C-C-Winkel um das nicht symmetrische C-Atom betragen 104,41 (104,16)°, 109,32 (109,29)°, 109,57 (109,53)° und 110,63 (110,63)°. Es gibt also durchaus Abweichungen von der idealen Tetraeder-Geometrie.

Der Winkel, der das C-Atom des Ringsystems enthält, ist ein wenig auf 110,6° aufgeweitet, hier werden sterische Effekte eine Rolle spielen.

Für Fortgeschrittene: In der Packungs-Darstellung erkennt man, dass die H-Atome an den terminalen C-Atomen, die an dem kleinen Winkel von 104° beteiligt sind, einen engen Kontakt zu denen von benachbarten Atomen haben. Hier werden zwischenmolekulare Kräfte (London-Kräfte) eine Rolle spielen.

2. Auch das Molekül des Tetramethylethens ist symmetrisch aufgebaut (D_2h_), ein Inversionszentrum befindet sich in der Mitte der C=C-Doppelbindung. Die Winkel betragen 112,15° und 123,86° bzw. 123,99°. Hier zeigt sich die aufweitende Wirkung durch die stärkere Abstoßung des Doppelbindungs-Elektronenpaars.

Das Molekül ist annähernd planar: Der Torsionswinkel beträgt 0,26°.

3. Für das But-2-in erwartet man nach dem VSEPR-Molekül eine lineare Geometrie. Die C-C-C-Winkel im But-2-in betragen 179,15° bzw. 179,34°. Innerhalb des Moleküls finden sich keine Symmetrieelemente (ein Molekül pro asymmetrischer Einheit).

Bereits ohne Packungsdarstellung fällt auf, dass das co-kristallisierte HCl-Molekül mit seinem H-Atom genau auf die C-C-Dreifachbindung ausgerichtet ist. Man beobachtet hier eine Wasserstoffbrücke vom Typ X-H ··· π. Das Cl-Atom ist genau auf die positiv teilgeladenen endständigen C-Atome ausgerichtet.

4. Die drei Winkel um das N-Atom betragen alle drei 110,65°. Die exakt gleich Werte sind durch die C_3_-Symmetrie des Moleküls bedingt (eine dreizählige Drehachse durch das N-Atom). Damit findet man eine geringfügige Aufweitung des Tetraederwinkels. Dies überrascht, denn man sollte vermuten, dass das freie Elektronenpaar am Stickstoff-Atom zu einer Winkelverengung führt.

In der Struktur des Tetramethylammonium-Ions ([YALROS](../../CIF/Others/YALROS.cif)) befinden sich zwei halbe Ionen in der asymmetrischen Einheit. In den beiden Ionen betragen die C-N-C-Winkel 109,29° und 109,70° bzw. 109,17° und 109,51°. Hier haben wir also einen nahezu idealen Tetraeder vorliegen.

In der Ammoniak-Struktur ([KATLAT](../../CIF/Others/KATLAT.cif)) betragen die H-N-H-Winkel 101°, 103° und 102° (Die Standardabweichung der Winkel lässt sich unter „More Info“ – „Angles List“ abrufen: sie beträgt 2°). Damit liegen die Werte deutlich unter denen der C-N-C-Winkel im Trimethylamin. Das steht in Übereinstimmung mit der o.g. Vermutung, dass das freie Elektronenpaar zur Winkelverengung führt.

Daraus lässt sich für das Trimethylamin schließen, dass die sterische Wirkung der Methylgruppen die Winkelverengung durch das freie Elektronenpaar (sogar über‑)kompensiert.

5. Das Ion bildet eine oktaedrische Anordnung, alle Winkel liegen dicht an 90°, auch die Bindungslängen sind nahezu identisch mit 1,570-1,590 Å.

Pentacarbonyleisen weist einen trigonal-bipyramidalen Aufbau auf. Die Kohlenstoffatome der axialen CO-Moleküle bilden einen Winkel von 176,38° aus und liegen damit fast linear zueinander. Die äquatorialen Winkel liegen mit zweimal 121.96° und einmal 116,07° in der Nähe des trigonal-planaren Winkels von 120°. Die drei äquatorialen C-Atome liegen mit dem Eisen-Atom exakt in einer Ebene (Torsionswinkel = 180,0°). Die Fe-C-Abstände unterscheiden sich nicht signifikant, es handelt sich um einen regelmäßigen Körper.

6. Wie man über die Anzeige der Label zeigen kann, handelt es sich um ein Dibromdimethylselen-Molekül. Die beiden Brom-Atome bilden mit einem Winkel um das Selen-Atom von 177,67° eine nahezu lineare Anordnung. Die beiden Methyl-C-Atome sind um das Selen-Atom mit einem Winkel von 98° gebunden. Ein einfaches Abzählen der Valenzelektronen (Se=6, 2*Br=14, 2*C=8,6*H=6) ergibt, dass in der Formel 17 Elektronenpaare vorkommen müssen. Am Se-Atom muss sich demnach ein freies Elektronenpaar befinden. Entsprechend leitet sich die Struktur von der trigonal-bipyramidalen Grundgeometrie ab. Eine äquatoriale Position wird von dem freien Elektronenpaar eingenommen. Die stärker abstoßende Wirkung des freien Elektronenpaars sorgt für die Verengung des C-Se-C-Winkels auf 98,01°.
7. Im Cyclobutadien-Ring sind zwei C-C-Doppel- und zwei C-C-Einfachbindungen enthalten. Die bevorzugte Geometrie an der C=C-Doppelbindung ist trigonal planar mit einem Winkel von 120° (etwas gestaucht durch die Doppelbindung). Andererseits zwingt die Ringstruktur die vier C-Atome in einen Winkel von 90°. Die Struktur versucht dies durch Ausbrechen von der Planarität (Torsionswinkel 7,01°) zu umgehen.

Die nahezu gleichen C-C-Bindungslängen stehen im Widerspruch zur alternierenden Einfach/Doppelbindung. Ein aromatisches System ist aufgrund der Hückel-Regel nicht anzunehmen. Die Beobachtung, dass bei tieferer Temperatur die Unterschiede zwischen den Bindungen größer wird, spricht dafür, dass sich bei höherer Temperatur durch Molekülschwingungen die Bindungslängen angleichen (die Atompositionen „verschmieren“). Die Tieftemperaturstruktur ist nicht im Teaching Subset enthalten, sie findet sich aber unter dem RefCode [TBUCBD22](../../CIF/Others/TBUCBD10.cif).

*E. Irmer, XLAB – Göttinger Experimentallabor für junge Leute (2023)*
