# Supplementary material for: Crystallography in school
Source: J Appl Crystallogr. 2025 Sep 12;58(Pt 5):1802–9. doi: 10.1107/S1600576725007459 (PMC12502877; doi:10.1107/S1600576725007459)
Supplement: Supplementary file 3 [file j-58-01802-sup3.zip › Tutorials/German/Wasserstoffbrücken.docx]

**Wasserstoffbrücken**

Die gebräuchlichste Definition einer Wasserstoff-Brücke (H-Brücke) fordert die Anwesenheit von drei Atomen, hier X, H und A genannt. X und A können unterschiedliche Elemente sein, während H immer ein Wasserstoff-Atom darstellt. Gemäß dieser Konvention können wir eine Wasserstoffbrücke mit einer gepunkteten Linie darstellen:

X-H^…^A

- X ist ein Nichtwasserstoff-Atom und muss eine höhere Elektronegativität aufweisen, so dass das H-Atom eine positive Teilladung erhält. X-H ist der Wasserstoffbrücken-Donor.
- A ist generell ein Atom mit einer hohen Elektronendichte, normalerweise ein Element mit einem oder mehreren freien Elektronenpaaren. A ist der H-Brücken-Akzeptor.
- Es gibt zwei nützliche Kriterien, um die Gegenwart und die Stärke einer H-Brücke abzuschätzen.
  - Der Abstand zwischen dem H-Atom und dem Akzeptor-Atom A sollte kürzer als die Summe der van-der-Waals-Radien der beiden Atome sein.
  - Die zweite Methode betrachtet den Winkel X-H^…^A um das H-Atom herum. Je dichter er an 180° liegt, desto stärker ist die H-Brücke.

Wir betrachten die Kristallstruktur der Ameisensäure ([FORMAC01](../../CIF/Carboxylic_acids/FORMAC01.cif)) und verfahren in zwei Schritten:

1) Zuerst identifizieren wir die Atome, die für eine Wasserstoffbrücke in Frage kommen könnten. Ordnen Sie den Bestandteilen der H-Brücke X-H^…^A die Atom-Label im Ameisensäure-Molekül aus der Kristallstruktur zu.

   *Es ergeben sich zwei Möglichkeiten: X-H^…^A: O1-H1^…^O2‘ oder C1-H2^…^O2“*

2) Überprüfen Sie die möglichen H-Brücken mithilfe der beiden Kriterien, ob es um Wasserstoffbrücken handelt und welche Stärke sie besitzen.
   Die van-der Waals-Radien nach Pauling betragen für das H-Atom 1,2 Å, für das O-Atom 1,4 Å. Jede X-H^…^A-Wechselwirkung mit einem H^…^A-Abstand kürzer als 2,6 Å kann als nennenswerte H-Brücke bezeichnet werden.

   *Die Wechselwirkung O1-H1^…^O2‘ ist mit einem H^…^A-Abstand* *von 1,764 Å eine starke H-Brücke. Dagegen ist die Wechselwirkung C1-H2^…^O2“ mit einem Abstand von 2,664 Å (wenn auch nur geringfügig) länger als die Summe der van-der-Waals-Radien. Auch ein Vergleich der beiden X-H^…^A-Winkel bestätigt diesen Befund. Im ersten Fall ist er mit 174,82° sehr dicht an 180°, während er im zweiten Fall mit 121,59° weit davon entfernt ist.*

Nun können wir dieses Verfahren auf weitere Kristallstrukturen anwenden und die Gegenwart und Stärke von Wasserstoffbrücken an den folgenden Beispielen überprüfen.

a) [ETDAMS](../../CIF/Others/ETDAMS.cif)
b) [NAPHTA04](../../CIF/Others/NAPHTA04.cif)
c) [UREAXX](../../CIF/Amines/UREAXX.cif)
d) [HIBFON](../../CIF/Others/HIBFON.cif) (nicht im Teaching Subset)
e) [FOQBAO](../../CIF/Others/FOQBAO.cif)
f) [SOCLIF](../../CIF/Others/SOCLIF.cif)
g) [ABEGIY](../../CIF/Others/ABEGIY.cif)

**Für Fortgeschrittene:**

1) Betrachte die Kristallstruktur von Phenol ([PHENOL03](../../CIF/Aromatics/PHENOL03.cif)). Zeige, dass in dieser Struktur ein und dasselbe Atom als Donor (X) und als Akzeptor (A) vorkommt.
   Untersuche, ob dies auch in den folgenden Fällen passiert:
   a) [XIBTUY](../../CIF/Others/XIBTUY.cif)
   b) [ABEKUN](../../CIF/Others/ABEKUN.cif)
   c) [NIYQUH](../../CIF/Others/NIYQUH.cif)
   d) [DEMTEU](../../CIF/Others/DEMTEU.cif)
   e) [FIGYID](../../CIF/Others/FIGYID.cif)
2) Der Sonderfall der intramolekularen Wasserstoffbrücken: Betrachte die Kristallstruktur mit dem Refcode [KEFRAP](../../CIF/Others/KEFRAP.cif). Der Abstand H^…^A entspricht den Kriterien für eine H-Brücke der Winkel jedoch ist weit von 180° entfernt. Dennoch drehen sich die beiden funktionellen Gruppen so, dass die Wechselwirkung zustande kommt.
   Untersuche bei den folgenden Beispielen, ob eine intramolekulare H-Brücke ausgebildet wird:
   a) [FRUCTO11](../../CIF/Carbohydrates/FRUCTO11.cif)
   b) [GUANPH](../../CIF/Others/GUANPH.cif)
   c) [INDIGO03](../../CIF/Others/INDIGO03.cif)
   d) [FUJCUI](../../CIF/Others/FUJCUI.cif)
   e) [ABALEV](../../CIF/Alkanols/ABALEV.cif)

*Verändert nach: Stefano Canossa, Università degli Studi di Parma, Dipartimento di Chimica, https://www.ccdc.cam.ac.uk/Community/educationalresources/teaching-modules/Teaching%20Tutorial%20-%20Hydrogen%20Bond.pdf*

*Mit freundlicher Genehmigung von Stefano Canossa, TU Delft*

*E. Irmer, XLAB – Göttinger Experimentallabor für junge Leute (2023)*
