# Supplementary material for: Crystallography in school
Source: J Appl Crystallogr. 2025 Sep 12;58(Pt 5):1802–9. doi: 10.1107/S1600576725007459 (PMC12502877; doi:10.1107/S1600576725007459)
Supplement: Supplementary file 3 [file j-58-01802-sup3.zip › Tutorials/German/Wasserstoffbrücken.pdf]

Wasserstoffbrücken

Die gebräuchlichste Definition einer Wasserstoff-Brücke (H-Brücke) fordert die Anwesenheit von drei Atomen, hier X, H und A genannt. X und A können unterschiedliche Elemente sein, während H immer ein Wasserstoff-Atom darstellt. Gemäß dieser Konvention können wir eine Wasserstoffbrücke mit einer gepunkteten Linie darstellen:

X-H···A

- X ist ein Nichtwasserstoff-Atom und muss eine höhere Elektronegativität aufweisen, so dass das H-Atom eine positive Teilladung erhält. X-H ist der Wasserstoffbrücken-Donor.
- A ist generell ein Atom mit einer hohen Elektronendichte, normalerweise ein Element mit einem oder mehreren freien Elektronenpaaren. A ist der H-Brücken-Akzeptor.
- Es gibt zwei nützliche Kriterien, um die Gegenwart und die Stärke einer H-Brücke abzuschätzen.
    - Der Abstand zwischen dem H-Atom und dem Akzeptor-Atom A sollte kürzer als die Summe der van-der-Waals-Radien der beiden Atome sein.
    - Die zweite Methode betrachtet den Winkel X-H···A um das H-Atom herum. Je dichter er an 180° liegt, desto stärker ist die H-Brücke.

Wir betrachten die Kristallstruktur der Ameisensäure (FORMAC01) und verfahren in zwei Schritten:

1) Zuerst identifizieren wir die Atome, die für eine Wasserstoffbrücke in Frage kommen könnten. Ordnen Sie den Bestandteilen der H-Brücke X-H···A die Atom-Label im Ameisensäure-Molekül aus der Kristallstruktur zu.

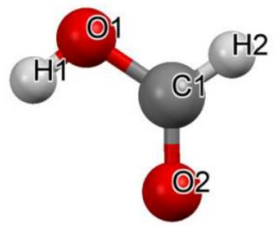

   Es ergeben sich zwei Möglichkeiten: X-H···A: O1-H1···O2ʻ oder C1-H2···O2“

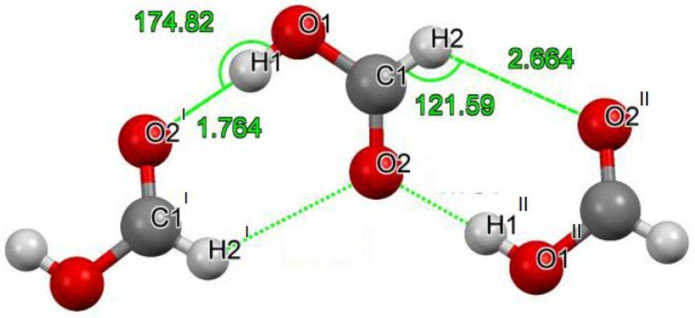

2) Überprüfen Sie die möglichen H-Brücken mithilfe der beiden Kriterien, ob es um Wasserstoffbrücken handelt und welche Stärke sie besitzen.

   Die van-der Waals-Radien nach Pauling betragen für das H-Atom 1,2 Å, für das O-Atom 1,4 Å. Jede X-H···A-Wechselwirkung mit einem H···A-Abstand kürzer als 2,6 Å kann als nennenswerte H-Brücke bezeichnet werden.

   Die Wechselwirkung O1-H1···O2′ ist mit einem H···A-Abstand von 1,764 Å eine starke H-Brücke. Dagegen ist die Wechselwirkung C1-H2···O2" mit einem Abstand von 2,664 Å (wenn auch nur geringfügig) länger als die Summe der van-der-Waals-Radien. Auch ein Vergleich der beiden X-H···A-Winkel bestätigt diesen Befund. Im ersten Fall ist er mit 174,82° sehr dicht an 180°, während er im zweiten Fall mit 121,59° weit davon entfernt ist.

Nun können wir dieses Verfahren auf weitere Kristallstrukturen anwenden und die Gegenwart und Stärke von Wasserstoffbrücken an den folgenden Beispielen überprüfen.

a) ETDAMS
b) NAPHTA04
c) UREAXX
d) HIBFON (nicht im Teaching Subset)
e) FOQBAO
f) SOCLIF
g) ABEGIY

Für Fortgeschrittene:

1) Betrachte die Kristallstruktur von Phenol (PHENOL03). Zeige, dass in dieser Struktur ein und dasselbe Atom als Donor (X) und als Akzeptor (A) vorkommt.

   Untersuche, ob dies auch in den folgenden Fällen passiert:

   a) XIBTUY
   b) ABEKUN
   c) NIYQUH
   d) DEMTEU
   e) FIGYID

2) Der Sonderfall der intramolekularen Wasserstoffbrücken: Betrachte die Kristallstruktur mit dem Refcode KEFRAP. Der Abstand H···A entspricht den Kriterien für eine H-Brücke der Winkel jedoch ist weit von 180° entfernt. Dennoch drehen sich die beiden funktionellen Gruppen so, dass die Wechselwirkung zustande kommt.

   Untersuche bei den folgenden Beispielen, ob eine intramolekulare H-Brücke ausgebildet wird:

   a) FRUCTO11
   b) GUANPH
   c) INDIGO03
   d) FUJCUI
   e) ABALEV

Verändert nach: Stefano Canossa, Università degli Studi di Parma, Dipartimento di Chimica, https://www.ccdc.cam.ac.uk/Community/educationalresources/teaching-modules/Teaching%20Tutorial%20-%20Hydrogen%20Bond.pdf

Mit freundlicher Genehmigung von Stefano Canossa, TU Delft

E. Irmer, XLAB – Göttinger Experimentallabor für junge Leute (2023)
