# Supplementary material for: Crystallography in school
Source: J Appl Crystallogr. 2025 Sep 12;58(Pt 5):1802–9. doi: 10.1107/S1600576725007459 (PMC12502877; doi:10.1107/S1600576725007459)
Supplement: Supplementary file 3 [file j-58-01802-sup3.zip › Tutorials/Mercury An Introduction Tutorial.docx]

**Mercury – An Introduction Tutorial**

**Access to the CSD Teaching Subset:**

Start the programme "Mercury" and open the Teaching Subset through "CSD Community" - "Open Teaching Database".

On the right you will see the "Structure Navigator" with the refcodes of the crystal structures in the teaching subset.

Load the structure of **acetylsalicylic acid, ASS or Aspirin^©^** ([ACSALA01](../CIF/Natural_products/acsala01.cif)): To do this, either scroll to the entry or enter the first letters in the search field at the top of the "Structure Navigator" and click on the refcode. A molecular model of the structure appears in the left-hand window. The way in which the molecule is displayed depends on the pre-selected settings.

**Basic movement options:**

Hold down the left mouse button and move:	Rotate in all three spatial directions

Keep middle mouse button pressed and move:	Move in two directions

Keep right mouse button pressed and move:	Zooming

Controlled movement of the molecule is also possible via the menus "x-, x+, y-, y+,..." etc.

**Various display options:**

Different **display styl**es can be selected via the menu item "Styles":

| Wireframe | Wireframe (bonds only) |
| --- | --- |
| Capped Sticks | Rod model (bonds only) |
| Ball and Stick | ball-and-stick model (atoms as balls, bonds as sticks) |
| Spacefill | spherical model, shows the space filling of the atoms |
| Ellipsoid | shows in some structures in which direction the atoms in the crystal are vibrating (the PF_6_^-^ ions in [FAMZEZ](../CIF/Others/FAMZEZ.cif)) |
| Polyhedral | Shows coordination polyhedra |

**Colour of the atoms:**

If you select "Colour" "by Element", the atoms are displayed in the typical CPK colour scheme:

Dark grey	Carbon

Light grey	Hydrogen

Red	Oxygen

Blue	Nitrogen

The atom names can be displayed under "Display Options" in the lower part of the screen: "Label atoms". H-atoms can also be shown or hidden: "Show hydrogens".

**Measurements of the geometry**

Distances, angles and torsion angles (angle between two planes) can be determined.

For **bond distances**, it is easiest to call up a list of bonds. To do this, select "Bond list" in the lower part of the screen (Display Options) under "More info". Here all bond lengths are displayed, partly with experimentally determined standard deviations in brackets). The bond type was set by the authors according to the given structural formula and is also displayed in the molecule graphic. Under "Display" "Styles", the bond type can be hidden by unchecking "Display Bond Types" and "Display Aromatic Rings".

To determine a single bond length, select "Measure Distances" under "Picking Mode" in the upper part of the screen and click on the two atoms whose distance you want to obtain.

*Examine the bond distances of the C-C bonds in the benzene ring and compare the C-O single bond lengths with the C=O double bond lengths.*

**Bond angles** are obtained in the same way by selecting "Measure Angles" under "Picking Mode" instead and clicking on three atoms. The angle around the middle atom is output.

*Examine the angles in the benzene ring.*

**Torsion angles** can be determined accordingly by "Measure Torsions". Here, four atoms are clicked.

*Check the planarity of the benzene ring atoms and of the atoms around the C=O group.*

**Crystal packing**

Under "Display Options" in the lower part of the screen, the unit cell can be shown or hidden "Show cell axes".

By clicking on "Packing", the molecules contained in the unit cell are displayed (more molecules may be displayed if the packing settings have already been changed).

*Find out how many ASS molecules are in the unit cell.*

Select in the upper menu bar under "Calculate" - "Packing and Slicing" how many unit cells are to be displayed. Enlarge the right value under "a:" by clicking the arrow up several times or by clicking "+0.5". The **packing section** is selected accordingly. Be careful with very large sections: The computing power may be exceeded. Reset is possible at any time via "Reset".

If the molecule has an internal symmetry, the unit cell may not contain the entire molecule, but only the **asymmetric unit**. This can be checked by clicking on "Asymmetric Unit" in the lower part of the screen.

*Which parts of the molecule are connected by symmetry in the benzene structure (*[*BENZEN02*](../CIF/Aromatics/BENZEN02.cif)*)?*

Especially when looking at the crystal packing, it is helpful to look at the crystal section along certain cell axes. For this purpose, the selection buttons "a", "b" and "c" can be found in the upper half of the screen.

**Intermolecular contacts**

All distances that lie below the sum of the van der Waals radii of the atoms can be displayed. To do this, click on "Short Contacts" under "Display Options". With "H-Bonds" only those distances are displayed where O, S and N atoms are involved. For details refer to the tutorial "Hydrogen bonds".

*Find out if there are hydrogen bonds between ASS molecules. You will probably first have to limit the packing to one unit cell and then selectively add individual further molecules after selecting "Expand contacts" in "Picking Mode" by clicking on the atoms in question.*

*Author: E. Irmer, XLAB - Göttingen Experimental Laboratory for Young People (2019)*
