# Supplementary material for: Crystallography in school
Source: J Appl Crystallogr. 2025 Sep 12;58(Pt 5):1802–9. doi: 10.1107/S1600576725007459 (PMC12502877; doi:10.1107/S1600576725007459)
Supplement: Supplementary file 3 [file j-58-01802-sup3.zip › Installation notes CSD_Teaching_Materials_EI.pdf]

CSD Teaching Materials

Installation notes

Unzip the ‘CSD_Teaching_Materials_EI.zip’ file to a desired location on your computer. Please do not change the folder structure afterwards, as otherwise the links between the list of teaching subset structures and the files in the other folders will no longer work.

The structure can only be called up directly in Mercury if the file extension ‘cif’ is linked to the Mercury programme. This link is normally created when Mercury is installed. Otherwise, the link must be set yourself: Right-click on a *.cif file, select ‘Open with...’, search for the “Mercury” programme under ‘More apps’ and tick the box ‘Always use this app to open .cif files’.
